# Machine Learning Techniques for Antimicrobial Resistance Prediction of *Pseudomonas Aeruginosa* from Whole Genome Sequence Data

**DOI:** 10.1155/2023/5236168

**Published:** 2023-03-01

**Authors:** Sohail M. Noman, Muhammad Zeeshan, Jehangir Arshad, Melkamu Deressa Amentie, Muhammad Shafiq, Yumeng Yuan, Mi Zeng, Xin Li, Qingdong Xie, Xiaoyang Jiao

**Affiliations:** ^1^Department of Cell Biology and Genetics, Shantou University Medical College, Shantou, Guangdong 515041, China; ^2^Department of Medicine and Surgery, Al-Nafees Medical College and Hospital, Isra University, Islamabad 44000, Pakistan; ^3^Department of Electrical and Computer Engineering, Comsats University Islamabad, Lahore Campus 44000, Lahore, Pakistan; ^4^Department of Information Technology, Assosa University, Assosa 5220, Ethiopia

## Abstract

**Aim:**

Due to the growing availability of genomic datasets, machine learning models have shown impressive diagnostic potential in identifying emerging and reemerging pathogens. This study aims to use machine learning techniques to develop and compare a model for predicting bacterial resistance to a panel of 12 classes of antibiotics using whole genome sequence (WGS) data of Pseudomonas *aeruginosa*.

**Method:**

A machine learning technique called Random Forest (RF) and BioWeka was used for classification accuracy assessment and logistic regression (LR) for statistical analysis.

**Results:**

Our results show 44.66% of isolates were resistant to twelve antimicrobial agents and 55.33% were sensitive. The mean classification accuracy was obtained ≥98% for BioWeka and ≥96 for RF on these families of antimicrobials. Where ampicillin was 99.31% and 94.00%, amoxicillin was 99.02% and 95.21%, meropenem was 98.27% and 96.63%, cefepime was 99.73% and 98.34%, fosfomycin was 96.44% and 99.23%, ceftazidime was 98.63% and 94.31%, chloramphenicol was 98.71% and 96.00%, erythromycin was 95.76% and 97.63%, tetracycline was 99.27% and 98.25%, gentamycin was 98.00% and 97.30%, butirosin was 99.57% and 98.03%, and ciprofloxacin was 96.17% and 98.97% with 10-fold-cross validation. In addition, out of twelve, eight drugs have found no false-positive and false-negative bacterial strains.

**Conclusion:**

The ability to accurately detect antibiotic resistance could help clinicians make educated decisions about empiric therapy based on the local antibiotic resistance pattern. Moreover, infection prevention may have major consequences if such prescribing practices become widespread for human health.

## 1. Introduction

Antimicrobial resistance (AMR) is one of the leading public health concerns of the 21st century, which hinders the ability to effectively treat and prevent a wide variety of bacterial, viral, and fungal infections [1]. AMR occurs when microorganisms (bacteria, viruses, fungi, and parasites) evolve and lose their sensitivity to existing treatments, making infections more challenging to treat and raising the risk of disease transmission, severe illness, and death [2]. The rapid global spread of multi- and pan-resistant bacteria, also known as “superbugs,” is particularly concerning because these bacteria cause infections that cannot be treated with current antimicrobial medicines like antibiotics [3]. At least 1.27 million people died from AMR-related cases in 2019, according to the CDC (https://www.cdc.gov/drugresistance/biggest-threats.html). Over 2.8 million people in the United States year contract AMR, and over 35,000 people die directly [4]. The most common multidrug-resistant bacteria globally are *Escherichia coli*, *Enterococcus faecium*, *Streptococcus*, *Klebsiella*, and *Pseudomonas aeruginosa*, and they are responsible for an estimated 250,000 annual infections and deaths [5]. For instance, the WHO priority pathogen list calls for new antibacterials to treat infections caused by *Pseudomonas aeruginosa* and carbapenem-resistant bacteria (CRE) [6]. There are currently 32 antibiotics in clinical development that target WHO priority pathogens, but only six of them can be considered truly innovative [7].

Various researchers have talked about the resistance prediction of antimicrobials [8]. This lack of treatment options often requires broad-spectrum antibiotics, which may be less effective or safe. Resistance also affects empirical treatment, in which a clinician chooses an antibiotic for an infection without obtaining microbiological results. This can lead to an underestimation of the risk associated with specific infections and the use of inappropriate antibiotics. A meta-analysis found that patients with *Enterobacteriaceae* resistance are five times more likely to delay receiving an effective therapy than patients infected by a susceptible strain [9, 10]. This may reduce the long-term effectiveness of antibiotics, delay access to effective treatments, increase treatment failure with complications, and increase fatality rates. Infections caused by resistant Gram-positive and Gram-negative bacteria increase hospital stays, surgery needs, and mortality [11].

Another study by Yamani et al., calculated the health burden of antibiotic-resistant bacteria (ARB) in European Union/European Economic Area (EU/EEA) countries in disability-adjusted life-years [12]. Their models were populated with estimated incidence from the European Antimicrobial Resistance Surveillance Network (EARS-Net) and the European Centre for Disease Prevention and Control (ECDC) point prevalence surveys of healthcare-associated infections and antimicrobial use in European acute care hospitals [13, 14]. Systematic reviews of published literature showed attributable case fatality and length of stay for antibiotic-resistant infections [15, 16]. In 2014, 671689 infections occurred in EU/EEA countries [13]. This ratio increased globally between 2015 and 2022 [5, 10, 12]. Different ARB contribute variably to the global burden, so prevention and control strategies should be tailored to each country's needs. All countries must implement effective AMR strategies to combat antibiotic overuse and misuse [17]. All systemic antibiotics globally require a doctor's prescription. Most prescriptions are written in primary care, not secondary or tertiary [6].

In 2018, 74% of all antibiotics prescribed by the National Health Service (NHS) in England were for general practitioners (GPs) patients [18]. GPs are the most frequent antibiotic prescribers, so they focus on primary care literature. Nurse practitioners and community pharmacists play a key role. In the last 10 years, nurses' roles have expanded to include prescribing in many countries and are on the policy agenda in many more [19]. Nurse prescribing was introduced to better utilize the skills and knowledge of health professionals, improve medication access, and reduce the workload of doctors. In China, the number of nurses qualified to prescribe has steadily risen over the last 5 years, and 31,000 nurses now have the same prescribing ability as doctors [20]. Pharmacists in China can register as independent prescribers, often specializing in diabetes prescriptions. More pharmacists work in secondary care than primary. Lastly, dentists are considered antibiotic prescribers because they write fewer prescriptions than general practitioners. Further, most antibiotic prescriptions are for respiratory, urinary, skin, or tooth infections [21]. In addition, most antibiotics are given for acute respiratory tract infections (RTIs) [13]. Some RTIs, such as community-acquired bacterial pneumonia, are treatable with antibiotics, but most acute RTIs are viral and self-limiting.

*P. aeruginosa* has high baseline antibiotic resistance and can acquire new resistance mechanisms through chromosomal mutations or horizontal gene transfer (HGT), increasing the risk of ineffective antibiotic treatment [22]. Mutations can cause a failed therapeutic outcome during treatment, while resistance increases mortality, hospital stays, and costs. When microorganisms become resistant to antimicrobials, standard treatments are often ineffective. Disc diffusion and minimum inhibitory concentration (MIC) are the most common antimicrobial susceptibility tests [23]. Identification of resistance-specific markers by PCR or microarray hybridization is useful for epidemiological purposes and the validation of phenotypic results. As DNA sequencing throughput and costs increase, whole-genome sequencing (WGS) becomes a viable option for routine resistance profile surveillance and identifying emerging resistances [24]. Pathogenic *P. aeruginosa* alters genome sequences and protein expression to resist. Resistance disrupts biochemical pathways and protein channels [25]. Antibiotic resistance and susceptibility must be linked to specific resistance genes; all genes in an isolate are added to predict susceptibility [26]. ResFinder, CARD, and Resfams predict genotypes from phenotypes [27]. More and more often, computational tools like machine-learning algorithms are used to build models correlating genomic variations with phenotypes [28]. Both a stimulus and an outcome are present in every supervised learning example. The algorithm will succeed only if it learns a model that faithfully transforms any input into the desired output.

Considering the above, the fundamental objective of this study was to develop an accurate phenotype prediction model against antimicrobials. For this purpose, machine learning approaches called bio-Weka [29], and random forest (RF), and logistic regression (LR) [30–32] were used on the data mining platform called Weka (v3.9.2) (an open source java-based software) [33–35] for acquiring classification accuracy assumptions to accurately predict the phenotypes against a panel of twelve antimicrobial agents, including ampicillin, amoxicillin, meropenem, cefepime, fosfomycin, ceftazidime, chloramphenicol, erythromycin, tetracycline, gentamycin, butirosin, and ciprofloxacin from whole genome sequence data of *P. aeruginosa*. Significantly, this study can further enhance the antimicrobial predictions of various bacterial agents in clinical trials.

## 2. Methods

### 2.1. Data Collection

The WGS reads of *Pseudomonas aeruginosa* and binary resistance phenotypes of antimicrobial agents utilized in this study were obtained by accession numbers provided in various studies, consisting of different countries, including China and 65 others (developed and under development), and downloaded from the open access repository called GenBank at NCBI (https://www.ncbi.nlm.nih.gov/genbank/), which is the NIH genetic DNA sequences database. All the descriptive information about the raw data is present in the Supplementary file. The metadata consists of various attributes, including genome name, NCBI taxon id, genome status, associated strains, GenBank accession numbers, country name, number of contigs, genome lengths, isolation sources, resistance genes, twelve antibiotics, and many more.

### 2.2. Model Framework and Parameters

**Figure 1:**
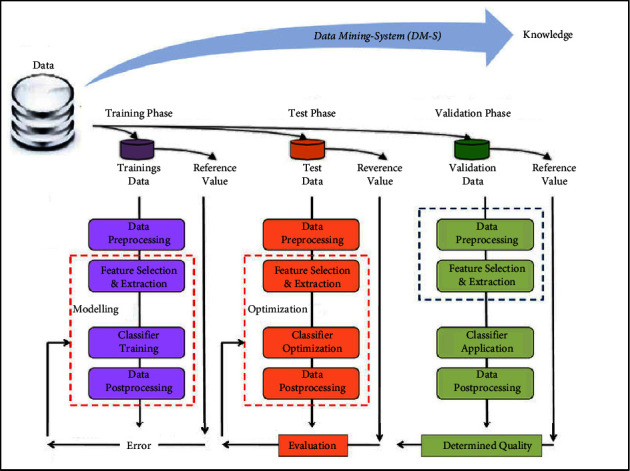
The data mining assessment framework used in this study.

In this study, antimicrobial resistance of *P. aeruginosa* was predicted using a data mining assessment framework by machine learning algorithms, as shown in Figure 1. There were a total of six stages involved in reaching these conclusions, including the following: objective; data collection and preparation; machine learning techniques on a data mining platform; model building; evaluation and assessment; and implications. Initially, we collected the data and did some preliminary preprocessing to pick the right attributes. Afterward, this data was used for analysis and assessment. Secondly, Weka (v3.9.2), “a java-based machine learning and data mining platform,” was used to measure and evaluate classifications with the most recent bio-Weka and RF plugins. In addition, the results of machine learning classifiers were used in logistic regression (LR) to evaluate the resistance phenotype assessment to twelve different antibiotic drugs, namely, ampicillin, amoxicillin, meropenem, cefepime, fosfomycin, ceftazidime, chloramphenicol, erythromycin, tetracycline, gentamycin, butirosin, and ciprofloxacin.

Furthermore, the data was divided into two sets (training set and testing set) by a ratio of 60 : 40. Overfitting was prevented by using 10-fold cross-validation, and training data were used further as efficiently as possible to determine the optimal hyperparameter settings. The training model's evaluation results were based on an average of the hyperparameter values that fared best in the 10-fold scross-validation procedure. Sensitivity, specificity, accuracy, and precision were used to assess the model performance of bio-Weka and RF by equations (1)–(4). The number of strains that turned out to be resistant was the true positive (TP), the number of strains that turned out to be sensitive was the true negative (TN), and the number of strains that turned out to be resistant when they should have been sensitive was the false positive (FP), and the number of strains that should have been sensitive when they should have been resistant was the false negative (FN) [36].

(1) $$\begin{array}{c} Sensitivity=\frac{TP}{\left(TP+FN\right)}\text{,} \end{array}$$

(2) $$\begin{array}{c} Specificity=\frac{TN}{\left(TN+FP\right)}\text{,} \end{array}$$

(3) $$\begin{array}{c} Accuracy=\frac{\left(TP+TN\right)}{\left(TP+FN+TN+FP\right)}\text{,} \end{array}$$

(4) $$\begin{array}{c} Precision=\frac{TP}{\left(TP+FP\right)}\text{.} \end{array}$$

### 2.3. BioWeka and Random Forest Prediction of Phenotypes Resistance

Weka's datasets are used and stored in a unique file format known as attribute relation file format (ARFF). Due to the wide variety of file types used for biological data, it implements a format-conversion input layer that can transform common file types into the ARFF format. Weka filters any classes that can be applied to a dataset to alter it, and bio-Weka has filters for working with biological sequences. It enabled us to compare and match sequences with BLAST and other sequence alignment tools. In addition, alignment-based classification was performed using auto alignment score evaluation schemes.

A java-based machine learning algorithm called bio-Weka and RF was used to perform the predictive modeling. The DSK (k-mer counting software) [37, 38] was used to generate K-mer profiles (abundance profiles of all unique words of length *k* in each genome) from the assembled contigs, with *k* = 31. This is a common length for analyzing bacterial genomes [39]. In order to create the dataset, the 31-mer profiles of all strains were combined using the combine kmers tool in SEER [40]. The combined 31-mer counts were converted into presence/absence matrices to be used for model training and prediction. 10-fold cross-validation was used to select the best conjunctive and/or disjunctive model with a maximum of ten rules for binary classification analysis (using S/NS phenotypes based on the two different breakpoints for each drug) [41, 42], which involved testing the suggested broad range of values for the trade-off hyperparameter to determine the optimal rule scoring function (https://aldro61.github.io/kover/doclearning.html). In addition, classification (BW-mC) and regression (BW-R) models were constructed from log2 (MIC) data in bio-Weka and RF for the purpose of comparing the performance of binary classifiers to MIC prediction [29, 43].

Furthermore, the RF method uses a majority voting strategy (MVS) to classify samples based on the results of an ensemble of decision tree (DT) [44]. In other words, the RF method relies on the class indicated by the vast majority of the DT. Having a diverse ensemble of trees is essential for boosting RF performance with respect to a single DT. One way to achieve it is by using bootstrapping with replacement to generate the training set for developing each DT's unique feature set. However, features considered for splitting each node are not chosen from the full feature set but rather from a subset of features [45]. In addition, be aware that RF is more akin to an unintelligible black box model. In RF, as in individual DT, the CART algorithm is taken into account.

Multiple metrics were used to evaluate the model's efficacy, including sensitivity, specificity, accuracy, precision, and the overall bACC (the average of the sensitivity and specificity) [46]. Since the bACC represents false positive and false negative rates equally, regardless of the imbalance in the dataset, it was chosen as the overall measure of model performance. Two measures of MIC prediction accuracy were evaluated: firstly, the proportion of isolates for which the predicted MIC was identical to the phenotypic MIC (rounded to the nearest doubling dilution in the case of regression), and secondly, the proportion of isolates for which the predicted MIC was within one doubling dilution of the phenotypic MIC (1-tier accuracy). The MIC testing criteria for exact match rates and 1-tier accuracies have been removed to include predictions within 0.5 doubling dilutions or 1.5 doubling dilutions of the phenotypic MIC, respectively, to account for MIC variation [47]. Each analysis had 10 replicates, and the mean and 95% confidence intervals were calculated for all metrics. Mean bACC was compared between replicate sets using two-tailed unpaired *t*-tests with logistic regression (LR) correction for unequal variance (*α* = 0.05) to assess differential model performance across datasets or methods. In addition, *P* values were calculated using the results of these unpaired *t*-tests.

### 2.4. Regression Statistics

Kappa statistics are reliable because they can be tested repeatedly [48, 49], ensuring that researchers have access to accurate, comprehensive data regarding research samples. It evaluates the predicted classification accuracy against a random classification [50]. We used a kappa statistic that relies on binary values, where 0 is considered as a null value and 1 represents the predicted outcome of the evaluation as in equation (5)–(7) [51]. It also serves as an indicator of the reliability of the evaluation. Not only that, but the LR variables help resolve the two-way binary classifications. When applied to the field of binary numbers, it makes predictions in the form of continuous values that allow for the preservation of sensitivity [36]. If the value is greater than the threshold (value > threshold), then the value assigned is 1; otherwise, the value measured is 0 as determined by the equations (8)–(11) [52].

(5) $$\begin{array}{c} K=\frac{\left[P\left(A\right)-P\left(E\right)\right]}{\left[1-P\left(E\right)\right]}\text{,} \end{array}$$

(6) $$\begin{array}{c} P\left(A\right)=\left[\frac{\left(TP+TN\right)}{N}\right]\text{,} \end{array}$$

(7) $$\begin{array}{c} P\left(E\right)=\left[\left(TP+FN\right)*\left(TP+FP\right)*\frac{\left(TN+FN\right)}{N^{2}}\right.\text{,} \end{array}$$

(8) $$\begin{array}{c} P=\alpha +\beta_{1}X_{1}+\beta_{2}X_{2}+\cdots +\beta_{m}X_{m}\text{,} \end{array}$$

(9) $$\begin{array}{c} \sigma \left(x\right)\frac{1}{1+e^{-x}}\in \left[0,1\right]\text{,} \end{array}$$

(10) $$\begin{array}{c} \mathrm{Pr}\;\left(Y=+1\left|X\right.\right)∼\beta .X\text{,} \end{array}$$

(11) $$\begin{array}{c} \mathrm{Pr}\;\left(Y=-1\left|X\right.\right) \end{array}$$

## 3. Results

**Figure 2:**
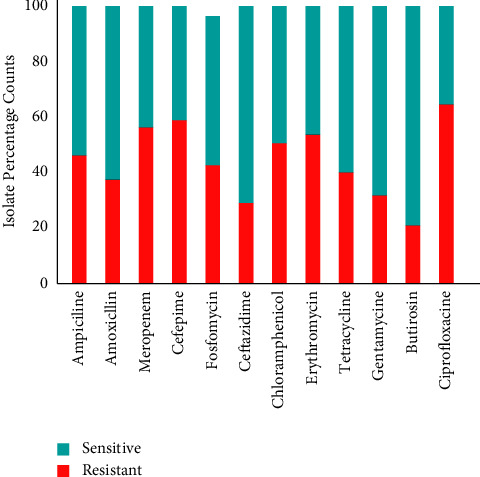
Number of resistant and sensitive isolate counts.

A total of 1200 isolates of *P. aeruginosa* were included in this study, out of which 44.66% were resistant to 12 antimicrobial agents and 55.33% were sensitive, as shown in Figure 2. Of which 44.66% resistant isolates, 44 were resistant to ampicillin, 37 to amoxicillin, 58 to meropenem, 60 to cefepime, 45 to fosfomycin, 30 to ceftazidime, 52 to chloramphenicol, 58 to erythromycin, 39 to tetracycline, 30 to gentamycin, 20 to butirosin, and 63 to ciprofloxacin. In addition, of 55.33% of sensitive isolates, 56 were sensitive to ampicillin, 63 to amoxicillin, 42 to meropenem, 40 to cefepime, 55 to fosfomycin, 70 to ceftazidime, 48 to chloramphenicol, 42 to erythromycin, 61 to tetracycline, 70 to gentamycin, 80 to butirosin, and 37 to ciprofloxacin, respectively. The most resistant genes to these twelve antimicrobial drugs were included *blaOXA-396*, *blaPAO*, *aph(3′)-IIb*, *catB5*, *qacE*, *blaOXA-488*, *aac(6′)-Ib-cr*, *aph(3′)-Iia*, *aph(6)-Ic*, *aac(6′)-Ib3*, *fosA*, *sul1*, *catB7*, *blaPAO*, *aac(3)-Ia*, *aac(6′)-Il*, *aph(3′)-Iib*, *sul1catB7*, *blaPAO*, *blaOXA-396*, *blaOXA494*, *qacE*, *crpP*, *catB7*, *blaPAO*, and *blaOXA-488*. Furthermore, from the analysis total of 19,371,434, k-mers were obtained of length 31. Which were compared from the ResFinder k-mer genes database, and a range of (1,302,507) k-mers of *fosA*, *catB7*, *crpP*, *aac(6′)-Ib-cr*, *fosA*, *tet(G)*, *aadA6*, *aph(3′)-Iib*, *sul1*, *aph(3′)-XV*, *aac(6′)-Ib3*, *bla*_OXA-488_, *bla*_GES-13_, *bla*_GES-7_, *bla*_GES-5_, *bla*_GES-6_, *bla*_PAO_, *qacE*, *crpT*, *aph(3′)-Iib*, *aadA13*, *bla*_OXA-50_, and *qacE* genes were detected in genome of 360 stains.

**Figure 3:**
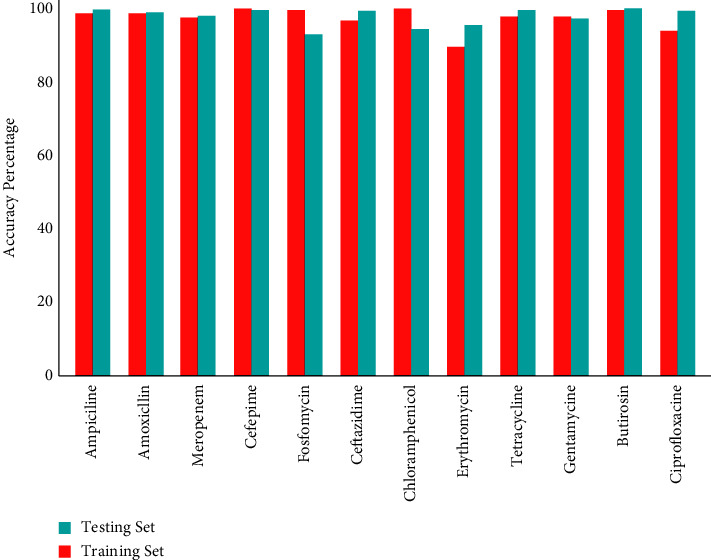
BioWeka classification accuracy percentage of the training set and testing set of twelve antimicrobial drugs.

The accuracy percentage obtained from the results of BioWeka was more than 98% (as a mean percentage) including the training set and testing set, as shown in Figure 3 for all twelve antimicrobial drugs, namely, ampicillin, amoxicillin, meropenem, cefepime, fosfomycin, ceftazidime, chloramphenicol, erythromycin, tetracycline, gentamycin, butirosin, and ciprofloxacin with the confidence factor of 0.25% by 10-fold-cross validation. After the loop tests, the final mean accuracy for ampicillin was (99.31%), amoxicillin was (99.02%), meropenem was (98.27%), cefepime was (99.73%), fosfomycin was (96.44%), ceftazidime was (98.63%), chloramphenicol was (98.71%), erythromycin was (95.76%), tetracycline was (99.27%), gentamycin was (98.00%), butirosin was (99.57%), and ciprofloxacin was (96.17%).

**Figure 4:**
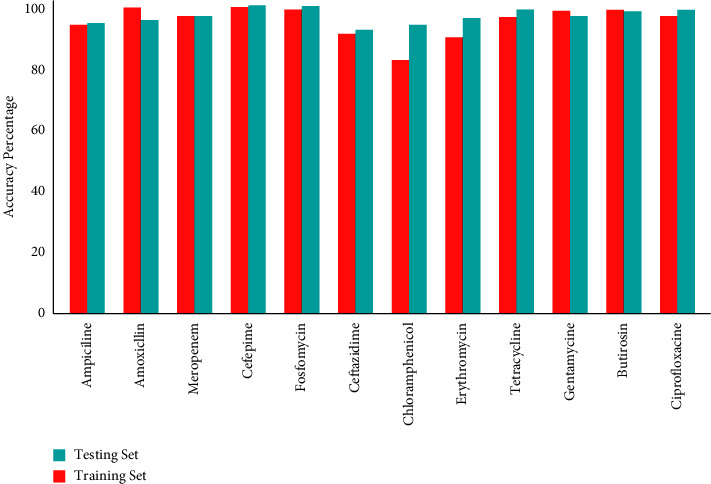
Random forest classification accuracy percentage of the training set and testing set of twelve antimicrobial drugs.

**Figure 5:**
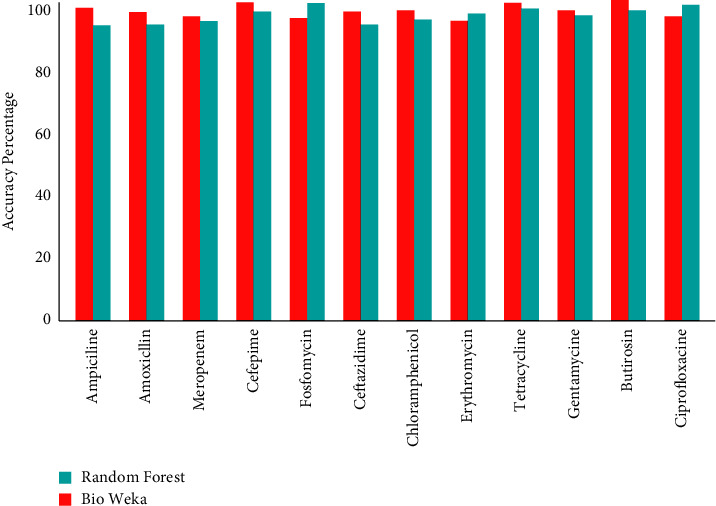
Mean accuracy percentage of random forest and BioWeka in comparison of twelve antimicrobial drugs.

In addition, Figure 4 shows the resulted classification accuracy percentage of RF algorithm in contrast to twelve antimicrobial drugs. The mean classification percentage was calculated more than 96% including the training set and testing set, as shown in Figure 5. After the loop testing, the final accuracy by RF for ampicillin was (94.00%), amoxicillin was (95.21%), meropenem was (96.63%), cefepime was (98.34%), fosfomycin was (99.23%), ceftazidime was (94.31%), chloramphenicol was (96.00%), erythromycin was (97.63%), tetracycline was (98.25%), gentamycin was (97.30%), butirosin was (98.03%), and ciprofloxacin was (98.97%). Furthermore, the standard deviation and average percentages of sensitivity, accuracy, precision, and specificity measured on the testing dataset are shown in Table 1. Our results of the testing dataset show that the antimicrobial drugs, namely ampicillin, amoxicillin, meropenem, cefepime, ceftazidime, tetracycline, butirosin, and ciprofloxacin, have no false-positive and false-negative bacterial strains.

## 4. Discussion

A number of studies have highlighted the increasing global prevalence of antimicrobial resistance [12–16, 21, 24, 27, 53–57]. This is related to the challenges of treating bacterial infections, the consequences of which can be severe. *P. aeruginosa* is one of the most common bacterial species, and its families are responsible for some of the most dangerous infections ever seen in humans. There is a correlation between the resistance of these bacteria to multiple antibiotic classes and the severity of the infection, which complicates treatment. Antibiotic resistance among these microorganisms has been rising steadily over the years, and it is now common to find clinical samples resistant to multiple drugs. The development of antibiotic resistance causes doctors to delay administering the most effective treatment methods and prescribe a larger dosage of antibiotics than is necessary. This is particularly important in the intensive care unit, where patients' health conditions necessitate longer courses of antibiotics. The extensive use of expensive medical interventions, increased mortality rates, and lengthened hospital stays are all consequences of antimicrobial resistance [58]. Another topic of great interest is the need to prevent the spread of bacteria resistant to antibiotics and to identify them in advance so that patients can be isolated as soon as possible. Since this is the case, novel approaches must be proposed for detecting antimicrobial resistance and taking appropriate action without delay. In addition, gaining insight into the factors that contribute to the spread of nosocomial infections is possible by identifying relevant features.

In this paper, we propose a data mining strategy based on two machine learning techniques, namely, bio-Weka and RF with a statistical approach for detecting the antimicrobial resistance of *P. aeruginosa* with different families of drugs. BioWeka and RF has shown that machine learning-based feature selection works with highly resulted accuracy as in Table 2. Consideration of antimicrobial drug resistance and susceptibility within data mining models and methods has been demonstrated to be useful in accelerating the workflow of clinical centers. Benefits for the individual, the healthcare system, and society may result from the early identification of patients at high risk of being resistant to one or more families of antibiotics. In addition, benefits include potential use in selecting the best antimicrobial treatment immediately.

Furthermore, the best performance achieved when testing this model strategy for resistance identification of antimicrobial drugs was a ROC area of 0.91 with a mean accuracy of more than 97% with all twelve drugs, indicating that our model can distinguish between the different classes of antibiotic susceptibility based solely on the type of the examined sample, the Gram stain classification of the pathogen, and prior antibiotic susceptibility testing results. We can foresee the sensitivity results from the various researchers using the model presented in this study. The ability to accurately detect antibiotic resistance could help clinicians make educated decisions about empiric therapy based on the local antibiotic resistance pattern. There may be major consequences for infection prevention if such prescribing practices become widespread.

The model proposed in this study has only the limitation with the process of filtering by 60 : 40 ratio with 10- fold cross-validation. If the ratios change then the accuracy and sensitivity of model might get affected. In addition, once the patient's clinical characteristics are added to the antimicrobial susceptibility dataset, the prediction performance of our model will significantly increase in terms of resistance prediction accuracy to different drugs. However, still, any such inclusion must incur the cost of retrieving the relevant data, which may be an exercise that involves a number of healthcare units, thereby increasing communication costs and complicating the need to align protocols that may operate across departments. After incurring such information, it is important to evaluate how well the additional knowledge acquired in terms of the improved accuracy metrics of the model can be incorporated into the practice of the hospital physicians, who may need to reevaluate their decision-making processes in the context of supporting or contradicting recommendations from a decision support system. To sum up, we think of this study as a node on a spectrum of cost-effectiveness studies that data mining approaches and machine learning techniques will spark in the healthcare industry.

## Figures and Tables

**Table 1:** Classification ratio of antimicrobial drugs against BioWeka and RF with phenotypes correlations.

Algorithm against drugs		Accuracy	Sensitivity	Specificity	Precision	F1 score	Kappa stats	Phenotype correlation
BioWeka classifications	Ampicillin	99.3 ± 0.0	86.0 ± 1.3	74.0 ± 2.3	1.0 ± 0.0	76.0 ± 3.2	91.0 ± 1.0	*p* < 2.1*e* − 1
	Amoxicillin	99.0 ± 0.0	62.0 ± 1.2	88.3 ± 1.2	1.0 ± 0.0	77.0 ± 1.0	91.2 ± 1.0	*p* < 2.1*e* − 1
	Meropenem	98.2 ± 0.0	88.0 ± 2.7	91.0 ± 1.0	1.0 ± 0.0	86.0 ± 2.5	89.3 ± 1.0	*p* < 2.1*e* − 1
	Cefepime	99.7 ± 0.0	89.0 ± 1.0	89.0 ± 1.0	1.0 ± 0.0	77.0 ± 1.0	94.8 ± 1.0	*p* < 2.1*e* − 1
	Fosfomycin	96.4 ± 0.0	77.0 ± 3.5	78.0 ± 2.1	1.0 ± 0.0	89.0 ± 1.0	97.6 ± 1.0	*p* < 2.1*e* − 1
	Ceftazidime	98.6 ± 0.0	85.0 ± 14.2	86.0 ± 3.7	1.0 ± 0.0	88.6 ± 2.0	91.3 ± 1.0	*p* < 2.1*e* − 1
	Chloramphenicol	98.7 ± 0.0	89.0 ± 2.1	78.0 ± 3.7	1.0 ± 0.0	91.9 ± 3.8	92.4 ± 1.0	*p* < 2.1*e* − 1
	Erythromycin	95.7 ± 0.0	91.0 ± 12.3	86.0 ± 3.2	1.0 ± 0.0	87.0 ± 1.0	89.9 ± 1.0	*p* < 2.1*e* − 1
	Tetracycline	99.2 ± 0.0	79.0 ± 1.7	89.0 ± 2.7	1.0 ± 0.0	79.0 ± 2.4	88.0 ± 1.0	*p* < 2.1*e* − 1
	Gentamycin	98.0 ± 0.0	92.0 ± 2.5	77.0 ± 2.1	1.0 ± 0.0	81.0 ± 1.0	88.0 ± 1.0	*p* < 2.1*e* − 1
	Butriosin	99.5 ± 0.0	88.0 ± 3.8	79.0 ± 12.1	1.0 ± 0.0	81.3 ± 2.7	87.6 ± 1.0	*p* < 2.1*e* − 1
	Ciprofloxacin	96.1 ± 0.0	87.0 ± 2.4	91.0 ± 1.0	1.0 ± 0.0	85.0 ± 1.0	83.8 ± 1.0	*p* < 2.1*e* − 1

Random forest classification	Ampicillin	94.0 ± 0.0	81.5 ± 2.1	88.4 ± 1.0	1.0 ± 0.0	84.9 ± 1.0	81.1 ± 1.0	*p* < 2.1*e* − 1
	Amoxicillin	95.2 ± 0.0	88.4 ± 2.5	81.2 ± 2.1	1.0 ± 0.0	88.6 ± 1.0	84.3 ± 1.0	*p* < 2.1*e* − 1
	Meropenem	96.6 ± 0.0	84.3 ± 3.6	73.9 ± 2.6	1.0 ± 0.0	87.1 ± 1.0	88.9 ± 1.0	*p* < 2.1*e* − 1
	Cefepime	98.3 ± 0.0	90.7 ± 2.2	77.0 ± 4.7	1.0 ± 0.0	82.5 ± 1.0	91.7 ± 1.0	*p* < 2.1*e* − 1
	Fosfomycin	99.2 ± 0.0	88.6 ± 2.3	76.8 ± 5.4	1.0 ± 0.0	77.7 ± 1.4	91.0 ± 1.0	*p* < 2.1*e* − 1
	Ceftazidime	94.3 ± 0.0	83.6 ± 2.1	83.7 ± 3.6	1.0 ± 0.0	79.0 ± 1.0	87.6 ± 1.0	*p* < 2.1*e* − 1
	Chloramphenicol	96.0 ± 0.0	89.7 ± 2.8	85.3 ± 2.9	1.0 ± 0.0	80.3 ± 2.7	84.9 ± 1.0	*p* < 2.1*e* − 1
	Erythromycin	97.6 ± 0.0	81.4 ± 4.6	82.6 ± 2.1	1.0 ± 0.0	78.7 ± 2.5	88.2 ± 1.0	*p* < 2.1*e* − 1
	Tetracycline	98.2 ± 0.0	83.9 ± 3.7	87.8 ± 3.1	1.0 ± 0.0	82.6 ± 1.0	91.9 ± 1.0	*p* < 2.1*e* − 1
	Gentamycin	97.3 ± 0.0	92.4 ± 2.6	79.6 ± 2.5	1.0 ± 0.0	89.4 ± 1.0	91.0 ± 1.0	*p* < 2.1*e* − 1
	Butriosin	98.0 ± 0.0	90.3 ± 3.1	81.9 ± 1.7	1.0 ± 0.0	86.3 ± 3.1	97.6 ± 1.0	*p* < 2.1*e* − 1
	Ciprofloxacin	98.9 ± 0.0	82.5 ± 3.5	88.6 ± 1.0	1.0 ± 0.0	81.2 ± 1.0	94.3 ± 1.0	*p* < 2.1*e* − 1

**Table 2:** Our machine learning resulted model accuracy percentage comparison with recent studies.

Methods	Accuracy (%)	References
BioWeka	≥98	This paper
Random forest	≥96	
Support vector machine (SVM)	≥95	[59]
Set covering machine (SCM)	≥96	[59]
Logistic regression (LR)	≥93	[44]
Decision tree (DT)	≥95	[44]
Random forest (RF)	≥97	[44]
Multi-layer perceptron (MLP)	≥91	[44]

## Data Availability

All data used in this study can be found in the Supplementary file associated with this article, or it can also be made available upon request to the first author or corresponding author.

## Supplementary Materials

Supplementary Materials All descriptive information about the raw data is present in the Supplementary file.
