# Supplementary material for: Machine Learning Techniques for Antimicrobial Resistance Prediction of Pseudomonas Aeruginosa from Whole Genome Sequence Data
Source: Comput Intell Neurosci. 2023 Mar 1;2023:5236168. doi: 10.1155/2023/5236168 (PMC9995192; doi:10.1155/2023/5236168)
Supplement: Supplementary Materials — All descriptive information about the raw data is present in the Supplementary file. [file 5236168.f1.docx]

Machine Learning Techniques for Antimicrobial Resistance Prediction of *Pseudomonas Aeruginosa* from Whole Genome Sequence Data

Sohail M. Noman ^1^, Muhammad Zeeshan ^2^, Jehangir Arshad ^3^, Melkamu Deressa Amentie ^4^, Muhammad Shafiq ^1^, Yumeng Yuan ^1^, Mi Zeng ^1^, Xin Li ^1^, Qingdong Xie ^1^, Xiaoyang Jiao ^1,^*

Genome Name Organism Name NCBI Taxon ID Genome Status Strain GenBank Accessions Contigs Genome Length Isolation Source Isolation Country Host Name AntiMicrobial Resistance AntiMicrobial Resistance Evidence Resistant Gene Fine Consistency Checkm Completeness Checkm Contamination Genome Quality Ampiciline amoxicillin meropenem cefepime fosfomycin ceftazidime chloramphenicol erythromycin tetracycline gentamycine butirosin ciprofloxacine

Pseudomonas aeruginosa PAK 1009714 Complete PAK LR657304 1 6395872 NA Resistant Computational Prediction "fosA, catB7, blaOXA-396, blaOXA-494, blaPAO, aph(3')-Iib" 99 Good 1 1 1 1 1 1 1 0 0 0 0 0

Pseudomonas aeruginosa CF27 Pseudomonas aeruginosa CF27 1402490 WGS CF27 AXRM00000000 23 6500574 Cystic fibrosis United States "Human, Homo sapiens" Resistant;Susceptible AMR Panel "blaOXA-50,blaPAO,fosA,aph(3')-Iib,catB7" 99.2 100 0.3 Good 1 1 0 1 1 1 1 0 0 0 0 0

Pseudomonas aeruginosa CF5 Pseudomonas aeruginosa CF5 1402491 WGS CF5 AXRL00000000 18 6330289 Cystic fibrosis United States "Human, Homo sapiens" Resistant;Susceptible;Intermediate AMR Panel "blaOXA-396,blaPAO,aph(3')-IIb,fosA,catB7" 98.9 99.7 0.6 Good 1 1 1 1 1 1 1 0 0 0 0 0

Pseudomonas aeruginosa X24509 Pseudomonas aeruginosa X24509 1402492 WGS X24509 AXRK00000000 23 6421104 UTI United States "Human, Homo sapiens" Resistant;Susceptible AMR Panel "blaPAO, blaOXA-486, catB7, aph(3')-IIb,crpP, fosA" 99.4 100 0.3 Good 1 1 1 1 1 1 1 0 0 0 0 1

Pseudomonas aeruginosa UDL Pseudomonas aeruginosa UDL 1402493 WGS UDL AXRJ00000000 18 6288577 UTI United States "Human, Homo sapiens" Susceptible;Intermediate AMR Panel "aph(3')-Iib,blaPAO,blaOXA-485,fosA,catB7,blaOXA-488" 99.7 100 Good 1 1 0 1 1 1 1 0 0 0 0 0

Pseudomonas aeruginosa S54485 Pseudomonas aeruginosa S54485 1402494 WGS S54485 AXRI00000000 19 7002867 UTI United States "Human, Homo sapiens" Resistant;Susceptible AMR Panel "fosA,blaPAO,blaOXA-488catB7,crpP,aph(3')-Iib" 99 100 Good 1 1 0 1 1 1 1 0 0 1 0 1

Pseudomonas aeruginosa U2504 Pseudomonas aeruginosa U2504 1402496 WGS U2504 AXRG00000000 28 7049946 UTI United States "Human, Homo sapiens" Resistant;Susceptible AMR Panel "qacE,blaOXA-488,aac(6')-Ib-cr,aph(3')-Iia,aph(6)-Ic,aac(6')-Ib3,fosA,sul1,catB7,blaPAO," 99.2 100 Good 1 1 0 1 1 1 1 0 0 1 0 1

Pseudomonas aeruginosa 6077 Pseudomonas aeruginosa 6077 1402498 WGS 6077 AXRE00000000 45 6971023 Cornea/ocular infection United States "Human, Homo sapiens" Resistant;Susceptible AMR Panel "aac(6')-Ib-cr,aac(6')-Ib3,crpP,aph(3')-IIb,aadA6,aac(6')-Ib3,tet(G),fosA,sul1,blaOXA-488,catB7,blaOXA-2,blaPAO,qacE" 98.8 100 Good 1 1 0 1 1 1 1 0 1 1 0 1

Pseudomonas aeruginosa S35004 Pseudomonas aeruginosa S35004 1402499 WGS S35004 AXRD00000000 32 6974149 Blood United States "Human, Homo sapiens" Resistant;Susceptible;Intermediate AMR Panel "aph(3')-IIb,crpP,blaOXA-396catB7,blaPAO,fosA" 98.4 100 1.2 Good 1 1 1 1 1 1 1 0 0 0 0 1

Pseudomonas aeruginosa X13273 Pseudomonas aeruginosa X13273 1402500 WGS X13273 AXRC00000000 23 7037692 Blood United States "Human, Homo sapiens" Resistant;Susceptible AMR Panel "crpP,aph(3')-Iib,catB7,blaOXA-488,blaPAO,fosA" 98.6 99.7 Good 1 1 0 1 1 1 1 0 0 1 0 1

Pseudomonas aeruginosa BWHPSA001 Pseudomonas aeruginosa BWHPSA001 1402501 WGS BWHPSA001 AXRB00000000 34 6414610 Urine United States "Human, Homo sapiens" Resistant;Susceptible;Intermediate AMR Panel "crpP,fosA,catB7,blaPAO,blaOXA-488,blaOXA-485,aph(3')-Iib" 99 100 Good 1 1 0 1 1 1 1 0 0 0 0 1

Pseudomonas aeruginosa BWHPSA002 Pseudomonas aeruginosa BWHPSA002 1402502 WGS BWHPSA002 AXRA00000000 21 6920040 Blood United States "Human, Homo sapiens" Resistant;Susceptible AMR Panel "crpP,aph(3')-Iib,catB7,blaPAO,blaOXA-396,fosA" 99.1 99.7 Good 1 1 1 1 1 1 1 0 0 0 0 1

Pseudomonas aeruginosa BWHPSA003 Pseudomonas aeruginosa BWHPSA003 1402503 WGS BWHPSA003 AXQZ00000000 35 6830561 Sputum United States "Human, Homo sapiens" Resistant;Susceptible;Intermediate AMR Panel "crpP,fosA,catB7,blaOXA-396,aph(3')-Iib,blaPAO," 98.4 99.7 Good 1 1 1 1 1 1 1 0 0 1 0 1

Pseudomonas aeruginosa BWHPSA004 Pseudomonas aeruginosa BWHPSA004 1402504 WGS BWHPSA004 AXQY00000000 16 6273839 "toenail, subungal debris" United States "Human, Homo sapiens" Susceptible;Intermediate AMR Panel "catB7,aph(3')-Iib,blaOXA-396,blaOXA-494,blaPAO,fosA" 99.6 100 Good 1 1 1 1 1 1 1 0 0 0 0 0

Pseudomonas aeruginosa BWHPSA006 Pseudomonas aeruginosa BWHPSA006 1402506 WGS BWHPSA006 AXQW00000000 20 6930020 BAL United States "Human, Homo sapiens" Resistant;Susceptible;Intermediate AMR Panel "crpP,fosA,catB7,blaPAO,blaOXA-488,aph(3')-Iib" 98.4 100 Good 1 1 0 1 1 1 1 0 0 1 0 1

Pseudomonas aeruginosa BWHPSA007 Pseudomonas aeruginosa BWHPSA007 1402507 WGS BWHPSA007 AXQV00000000 31 6753715 Abscess (abdominal) United States "Human, Homo sapiens" Resistant;Susceptible;Intermediate AMR Panel "fosA,aac(3)-Ia,aac(6')-Il,aph(3')-Iib,sul1catB7,blaPAO,blaOXA-396,blaOXA494,qacE" 98.7 100 1.2 Good 1 1 1 1 1 1 1 0 0 1 0 0

Pseudomonas aeruginosa BWHPSA008 Pseudomonas aeruginosa BWHPSA008 1402508 WGS BWHPSA008 AXQU00000000 21 6473493 shin wound United States "Human, Homo sapiens" Resistant;Susceptible;Intermediate AMR Panel "crpP,fosA,catB7,blaOXA-396,blaOXA-50,blaPAO,blaOXA-494,aph(3')-Iib" 99.1 100 Good 1 1 1 1 1 1 1 0 0 0 0 1

Pseudomonas aeruginosa BWHPSA012 Pseudomonas aeruginosa BWHPSA012 1402512 WGS BWHPSA012 AXQQ00000000 10 6476343 Sputum United States "Human, Homo sapiens" Resistant;Susceptible AMR Panel "crpP,fosA,catB7,blaOXA-486,blaPAO,aph(3')-Iib" 99.5 100 Good 1 1 0 1 1 1 1 0 0 0 0 1

Pseudomonas aeruginosa BWHPSA015 Pseudomonas aeruginosa BWHPSA015 1402515 WGS BWHPSA015 AXQN00000000 19 6414708 Urine United States "Human, Homo sapiens" Resistant;Susceptible;Intermediate AMR Panel "crpP,fosA,catB7,blaPAO,blaOXA-486,aph(3')-Iib" 98.9 100 Good 1 1 0 1 1 1 1 0 0 0 0 1

Pseudomonas aeruginosa BWHPSA017 Pseudomonas aeruginosa BWHPSA017 1402517 WGS BWHPSA017 AXQL00000000 19 6904844 Urine United States "Human, Homo sapiens" Resistant;Susceptible AMR Panel "crpP,aph(3')-Iib,catB7,blaPAO,blaOXA-396,fosA" 98.8 100 Good 1 1 1 1 1 1 1 0 0 1 0 1

Pseudomonas aeruginosa BWHPSA018 Pseudomonas aeruginosa BWHPSA018 1402518 WGS BWHPSA018 AXQK00000000 25 6604024 Wound United States "Human, Homo sapiens" Resistant;Susceptible;Intermediate AMR Panel "crpP,aph(3')-Iib,catB7,blaOXA-494,blaPAO,blaOXA-396,fosA" 99.3 100 0.3 Good 1 1 1 1 1 1 1 0 0 0 0 1

Pseudomonas aeruginosa BWHPSA019 Pseudomonas aeruginosa BWHPSA019 1402519 WGS BWHPSA019 AXQJ00000000 16 6408102 Wound United States "Human, Homo sapiens" Resistant;Susceptible AMR Panel "catB7,aph(3')-Iib,blaPAO,blaOXA-486,fosA" 99.6 100 Good 1 1 0 1 1 1 1 0 0 1 0 0

Pseudomonas aeruginosa BWHPSA021 Pseudomonas aeruginosa BWHPSA021 1402521 WGS BWHPSA021 AXQH00000000 13 6482332 Sputum United States "Human, Homo sapiens" Resistant;Susceptible AMR Panel "crpP,aph(3')-Iib,catB7,blaPAO,blaOXA-486,fosA" 99.3 100 Good 1 1 0 1 1 1 1 0 0 0 0 1

Pseudomonas aeruginosa BWHPSA025 Pseudomonas aeruginosa BWHPSA025 1402525 WGS BWHPSA025 AXQD00000000 14 6323768 Lung biopsy United States "Human, Homo sapiens" Susceptible;Intermediate AMR Panel "catB7,aph(3')-Iib,blaOXA-396,blaOXA-494,blaPAO,fosA" 99.4 100 0.3 Good 1 1 1 1 1 1 1 0 0 0 0 0

Pseudomonas aeruginosa BWHPSA026 Pseudomonas aeruginosa BWHPSA026 1402526 WGS BWHPSA026 AXQC00000000 23 6794049 QC org:ATCC 27853 United States "Human, Homo sapiens" Resistant;Susceptible;Intermediate AMR Panel "crpP,aph(3')-Iib,catB7,blaPAO,blaOXA-488,fosA" 98.7 100 Good 1 1 1 1 1 1 1 0 0 0 0 1

Pseudomonas aeruginosa BWHPSA041 Pseudomonas aeruginosa BWHPSA041 1402533 WGS BWHPSA041 AZZM00000000 27 5598610 Wound United States "Human, Homo sapiens" Resistant;Susceptible AMR Panel "fosA,catB7,blaOXA-494,blaOXA-396,blaPAO,aph(3')-Iib" 92.8 81.4 Good 1 1 1 1 1 1 1 0 0 0 0 0

Pseudomonas aeruginosa BWHPSA042 Pseudomonas aeruginosa BWHPSA042 1402534 WGS BWHPSA042 AZZL00000000 11 5886599 Sputum United States "Human, Homo sapiens" Resistant;Susceptible AMR Panel "fosA,catB7,blaPAO,blaOXA-50,aph(3')-Iib" 98.4 98.5 0.3 Good 1 1 0 1 1 1 1 0 0 0 0 0

Pseudomonas aeruginosa BWHPSA044 Pseudomonas aeruginosa BWHPSA044 1402536 WGS BWHPSA044 AZZJ00000000 12 6896541 urine United States "Human, Homo sapiens" Resistant;Susceptible AMR Panel "crpP,aph(3')-Iib,catB7,blaPAO,blaOXA-488,fosA" 99 100 Good 1 1 0 1 1 1 1 0 0 0 0 1

Pseudomonas aeruginosa BWHPSA048 Pseudomonas aeruginosa BWHPSA048 1402540 WGS BWHPSA048 AZZF00000000 13 5939118 urine United States "Human, Homo sapiens" Resistant;Susceptible AMR Panel "fosA,catB7,blaOXA-50,blaPAO,aph(3')-Iib" 98.4 97.3 0.3 Good 1 1 0 1 1 1 1 0 0 0 0 0

Pseudomonas aeruginosa BL03 Pseudomonas aeruginosa BL03 1402544 WGS BL03 AXPX00000000 22 7005139 Corneal Scaping United States "Human, Homo sapiens" Resistant;Susceptible AMR Panel "crpP,aph(3')-Iib,catB7,blaOXA-488,blaPAO,fosA" 97.9 100 1.5 Good 1 1 0 1 1 1 1 0 0 1 0 1

Pseudomonas aeruginosa BL05 Pseudomonas aeruginosa BL05 1402546 WGS BL05 AXPV00000000 15 6359317 Corneal Scraping United States "Human, Homo sapiens" Susceptible;Intermediate AMR Panel "catB7,aph(3')-Iib,blaPAO,blaOXA-50,fosA" 99.3 100 Good 1 1 0 1 1 1 1 0 0 0 0 0

Pseudomonas aeruginosa BL07 Pseudomonas aeruginosa BL07 1402548 WGS BL07 AXPT00000000 29 6490977 Conjunctiva United States "Human, Homo sapiens" Resistant;Susceptible;Intermediate AMR Panel "crpP,crpP,aph(3')-Iib,catB7,blaOXA-494,blaPAO,blaOXA-396,fosA" 99.3 100 0.3 Good 1 1 1 1 1 1 1 0 0 0 0 1

Pseudomonas aeruginosa BL12 Pseudomonas aeruginosa BL12 1402553 WGS BL12 AXPO00000000 42 7420344 Eye United States "Human, Homo sapiens" Resistant;Susceptible AMR Panel "crpP,fosA,catB7,blaPAO,blaOXA-488,aph(3')-Iib" 97.9 100 1.8 Good 1 1 0 1 1 1 1 0 0 0 0 1

Pseudomonas aeruginosa BL14 Pseudomonas aeruginosa BL14 1402555 WGS BL14 AXPM00000000 24 7101427 General Eye United States "Human, Homo sapiens" Susceptible;Intermediate AMR Panel "fosA,catB7,blaPAO,blaOXA-488,aph(3')-Iib" 98.5 100 0.9 Good 1 1 0 1 1 1 1 0 0 1 0 0

Pseudomonas aeruginosa BL17 Pseudomonas aeruginosa BL17 1402558 WGS BL17 AXPJ00000000 18 6865653 Eye United States "Human, Homo sapiens" Resistant;Susceptible;Intermediate AMR Panel "fosA,ant(2'')-Ia,aph(3')-Iib,aadA6,sul1,catB7,blaPAO,blaOXA-488,qacE," 99.2 100 Good 1 1 1 1 1 1 1 0 0 1 0 0

Pseudomonas aeruginosa BL18 Pseudomonas aeruginosa BL18 1402559 WGS BL18 AXPI00000000 18 6486858 General Eye United States "Human, Homo sapiens" Susceptible;Intermediate AMR Panel "crpP,fosA,catB7,blaOXA-395,blaPAO,aph(3')-Iib" 98.9 100 Good 1 1 1 0 1 1 1 0 0 0 0 1

Pseudomonas aeruginosa BWH049 1418235 WGS BWH049 JIFB00000000 22 6610309 urine United States "Human, Homo sapiens" Resistant;Susceptible AMR Panel "catB7,aph(3')-Iib,blaOXA-395,blaPAO,fosA" 99 100 Good 1 1 1 1 1 1 1 0 0 0 0 0

Pseudomonas aeruginosa BWH050 1418236 WGS BWH050 JIFA00000000 16 6560806 blood United States "Human, Homo sapiens" Resistant;Susceptible AMR Panel "fosA,catB7,blaOXA-488,blaPAO,aph(3')-Iib" 99.2 99.4 Good 1 1 0 1 1 1 1 0 0 1 0 0

Pseudomonas aeruginosa BWH053 1418239 WGS BWH053 JIEX00000000 25 6840259 blood United States "Human, Homo sapiens" Resistant;Susceptible AMR Panel "fosA,catB7,blaPAO,blaOXA-395,aph(3')-Iib" 99.2 100 Good 1 1 1 1 1 1 1 0 0 1 0 0

Pseudomonas aeruginosa BWH054 1418240 WGS BWH054 JIEW00000000 45 7167262 urine United States "Human, Homo sapiens" Resistant;Susceptible AMR Panel "crpP,fosA,catB7,blaPAO,blaOXA-395,aph(3')-Iib" 98.3 100 0.6 Good 1 1 1 1 1 1 1 0 0 1 0 1

Pseudomonas aeruginosa BWH056 1418242 WGS BWH056 JIEU00000000 34 6911218 blood United States "Human, Homo sapiens" Resistant;Susceptible AMR Panel "crpP,fosA,catB7,blaPAO,blaOXA-486,aph(3')-Iib" 98.7 100 0.6 Good 1 1 0 1 1 1 1 0 0 0 0 1

Pseudomonas aeruginosa BWH057 1418243 WGS BWH057 JIET00000000 34 6900307 blood United States "Human, Homo sapiens" Resistant;Susceptible AMR Panel "crpP,aph(3')-Iib,catB7,blaOXA-486,blaPAO,fosA" 98.6 100 0.6 Good 1 1 0 1 0 1 1 0 0 0 0 1

Pseudomonas aeruginosa BWH060 1418246 WGS BWH060 JIEQ00000000 18 6763011 urine United States "Human, Homo sapiens" Resistant;Susceptible;Intermediate AMR Panel "catB7,aph(3')-Iib,blaOXA-395,blaPAO,fosA" 99.1 100 Good 1 1 1 1 1 1 1 0 0 1 0 0

Pseudomonas aeruginosa PAO1-GFP 1418248 WGS PAO1-GFP JIEO00000000 22 6253573 lab strain "Human, Homo sapiens" Susceptible;Intermediate AMR Panel "catB7,aph(3')-Iib,blaOXA-50,blaPAO,fosA" 99.9 100 Good 1 1 0 1 1 1 1 0 0 0 0 0

Pseudomonas aeruginosa PA99 1437873 Complete PA99 CP042967 1 6946480 sputum Thailand "Human, Homo sapiens" Resistant Computational Prediction "fosA,msr€,aac(6')-Ib-cr,ere(A),mph€,sul1,dfrA1,ant(2'')-Ia,aac(6')-Ib3,aph(3')-Iib,aph(6)-Id,aph(3'')-Ib,aac(6')-Ib-cr,tet(G),tet(A),catB7,floR,blaIMP-1,blaIMP-1,blaOXA-21,blaPAO,blaOXA-396,qacE,qacE" 89.7 Good 1 1 1 1 1 1 0 1 0 1 0 1

Pseudomonas aeruginosa 3578 1447533 WGS 3578 JIEI00000000 115 6736273 Environmental sample "Human, Homo sapiens" Susceptible;Intermediate AMR Panel "fosA,catB7,blaPAO,blaOXA-486,aph(3')-Iib" 99 100 Good 1 1 0 1 1 1 1 0 0 0 0 0

Pseudomonas aeruginosa strain KCJ3K1 287 WGS KCJ3K1 VZIK01000000 312 6724970 USA "Human, Homo sapiens" Resistant Computational Prediction "crpP,catB7,aph(3')-Iib,blaOXA-395,blaPAO,FosA," 97.8 Good 1 1 1 1 1 1 1 0 0 0 0 1

Pseudomonas aeruginosa strain KCJ3K27 287 WGS KCJ3K27 VZIJ01000000 312 6612183 USA "Human, Homo sapiens" Resistant Computational Prediction "crpP,aph(3')-Iib,catB7,blaPAO,blaOXA-486,fosA" 98.2 Good 1 1 0 1 1 1 1 0 0 0 0 1

Pseudomonas aeruginosa strain KCJ3K10 287 WGS KCJ3K10 VZIM01000000 340 6932990 USA "Human, Homo sapiens" Resistant Computational Prediction "crpP,aph(3')-Iib,catB7,blaPAO,blaOXA-395,fosA" 97.9 Good 1 1 1 1 1 1 1 0 0 0 0 1

Pseudomonas aeruginosa AZPAE14932 287 WGS AZPAE14932 JTRJ00000000 177 6638040 respiratory tract infection Germany "Human, Homo sapiens" Resistant AMR Panel "crpP,fosA,catB7,blaPAO,blaOXA-488,aph(3')-Iib" 98.5 99.5 0.6 Good 1 1 0 1 1 1 1 0 0 1 0 1

Pseudomonas aeruginosa strain KCJ3K20 287 WGS KCJ3K20 VZIN01000000 404 6992869 USA "Human, Homo sapiens" Resistant Computational Prediction "crpP,aac(6')-Ib-cr,aph(3')-Iib,aadA7,aac(6')-Ib3,fosA,sul1,catB7,blaPAO,qacE,blaOXA-396" 97 Good 1 1 1 1 1 1 1 0 0 1 0 1

Pseudomonas aeruginosa strain KCJ3K57 287 WGS KCJ3K57 VZIP01000000 976 7121352 USA "Human, Homo sapiens" Resistant Computational Prediction "crpP,qnrVC1,aph(3')-VI,aadA1,ant(2'')-Ia,aph(3')-Iib,aadA10,,aadA3,fosA,dfrA1,sul1,catB7,blaVIM-1,blaPAO,blaOXA-488,qacE" 92.5 Good 1 1 1 1 1 1 1 0 0 1 0 1

Pseudomonas aeruginosa strain KCJ3K73 287 WGS KCJ3K73 VZIS01000000 197 6877823 USA "Human, Homo sapiens" Resistant Computational Prediction "crpP,fosA,aac(3)-Ib,aph(3')-Iib,sul1,blaOXA-50,catB7,blaPAO,qacE" 98.7 Good 1 1 0 1 1 1 1 0 0 1 0 1

Pseudomonas aeruginosa strain KCJ3K67 287 WGS KCJ3K67 VZIR01000000 533 6581083 USA "Human, Homo sapiens" Resistant Computational Prediction crpP 95.6 Good 0 0 0 0 0 0 0 0 0 0 0 1

Pseudomonas aeruginosa strain KCJ3K105 287 WGS KCJ3K105 VZIV01000000 286 6496217 USA "Human, Homo sapiens" Resistant Computational Prediction "crpP,aph(3')-Iib,catB7,blaOXA-485,blaPAO,fosA" 98.2 Good 1 1 0 1 1 1 1 0 0 0 0 1

Pseudomonas aeruginosa strain KCJ3K296 287 WGS KCJ3K296 VZIY01000000 209 6402916 USA "Human, Homo sapiens" Resistant Computational Prediction "catB7,aph(3')-Iib,blaOXA-486,blaPAO,fosA" 98.5 Good 1 1 0 1 1 1 1 0 0 0 0 0

Pseudomonas aeruginosa AZPAE14931 287 WGS AZPAE14931 JTRK00000000 61 6359186 respiratory tract infection Germany "Human, Homo sapiens" Resistant;Intermediate;Susceptible AMR Panel "catB7,aph(3')-Iib,blaOXA-50,blaPAO,fosA" 99.6 100 Good 1 1 0 1 1 1 1 0 0 0 0 0

Pseudomonas aeruginosa strain GO76 287 WGS GO76 WCHX01000000 185 6590937 USA Resistant Computational Prediction "crpP,fosA,catB7,blaPAO,aph(3')-Iib,blaOXA-395" 97.5 Good 1 1 1 1 1 1 1 0 0 0 0 1

Pseudomonas aeruginosa strain GO35 287 WGS GO35 WCHT01000000 74 6911823 USA Resistant Computational Prediction "crpP,fosA,catB7,blaPAO,aph(3')-Iib,blaOXA-396" 98.8 Good 1 1 1 1 1 1 1 0 0 0 0 1

Pseudomonas aeruginosa strain PSA-15 287 WGS PSA-15 FNLX01000000 129 6902016 Resistant Computational Prediction "crpP,aph(3')-Iib,catB7,blaPAO,blaOXA-488,fosA" 97.3 Good 1 1 0 1 1 1 1 0 0 0 0 1

Pseudomonas aeruginosa strain PSA-5 287 WGS PSA-5 FNLV01000000 66 6867575 Resistant Computational Prediction "crpP,aph(3')-Iib,catB7,blaPAO,blaOXA-488,fosA" 98.6 Good 1 1 0 1 1 1 1 0 0 0 0 1

Pseudomonas aeruginosa AZPAE14929 287 WGS AZPAE14929 JTRM00000000 91 6750744 urinary tract infection Germany "Human, Homo sapiens" Resistant AMR Panel "crpP,fosA,aph(3')-Iib,aadA2,aac(6')-Il,aac(3)-Id,dfrB5,tet(G),catB7,blaOXA-4,blaOXA-486,blaPAO,blaVIM-2,qacE" 99.2 99.4 Good 1 1 1 1 1 1 1 0 1 1 0 1

Pseudomonas aeruginosa strain PSA-6 287 WGS PSA-6 FNMA01000000 78 6873130 Resistant Computational Prediction "crpP,fosA,catB7,blaPAO,blaOXA-488,aph(3')-Iib" 98.4 Good 1 1 0 1 1 1 1 0 0 0 0 1

Pseudomonas aeruginosa strain PSA-13 287 WGS PSA-13 FNME01000000 76 6874720 Resistant Computational Prediction "crpP,fosA,catB7,blaPAO,blaOXA-488,aph(3')-Iib" 98.7 Good 1 1 0 1 1 1 1 0 0 0 0 1

Pseudomonas aeruginosa strain PSA-14 287 WGS PSA-14 FNMS01000000 77 6871711 Resistant Computational Prediction "crpP,fosA,catB7,blaPAO,blaOXA-488,aph(3')-Iib" 98.5 Good 1 1 0 1 1 1 1 0 0 0 0 1

Pseudomonas aeruginosa strain BS2366 287 WGS BS2366 "CABIPB010000001,CABIPB010000002,CABIPB010000003,CABIPB010000004,CABIPB010000005,CABIPB010000006,CABIPB010000007" 96 6926835 ENVO:01000990 Indonesia Resistant Computational Prediction "catB7,aph(3')-Iib,blaPAO-494,blaPAO-396,blaPAO,fosA" 98.6 Good 1 1 1 1 1 1 1 0 0 0 0 0

Pseudomonas aeruginosa strain BS2371 287 WGS BS2371 "CABIOW010000001,CABIOW010000002,CABIOW010000003,CABIOW010000004,CABIOW010000005,CABIOW010000006,CABIOW010000007" 104 6821546 ENVO:01000990 Indonesia Resistant Computational Prediction "fosA,aac(6')-Ib-cr,aph(3')-Iib,aadA2b,aac(3)-Ic,aadA6,aac(6')-Ib3,sul1,catB7,blaPAO,blaIMP-7,blaOXA-488,qacE" 98.9 Good 1 1 1 1 1 1 1 0 0 1 0 0

Pseudomonas aeruginosa strain BS2370 287 WGS BS2370 "CABIOX010000001,CABIOX010000002,CABIOX010000003,CABIOX010000004,CABIOX010000005,CABIOX010000006,CABIOX010000007" 117 6827798 ENVO:01000486 Indonesia Resistant Computational Prediction "fosA,aac(6')-Ib-cr,aac(3)-Ic,aadA2b,aac(6')-Ib3aadA6,aph(3')-Iib,sul1,catB7,blaPAO,blaIMP-7,blaOXA-488,qacE" 98.9 Good 1 1 1 1 1 1 1 0 0 1 0 1

Pseudomonas aeruginosa strain BS2369 287 WGS BS2369 "CABIOZ010000001,CABIOZ010000002,CABIOZ010000003,CABIOZ010000004,CABIOZ010000005,CABIOZ010000006,CABIOZ010000007" 111 6824970 ENVO:01000486 Indonesia Resistant Computational Prediction "fosA,aac(6')-Ib-cr,aph(3')-Iib,aac(3)-Ic,aac(6')-Ib3,aadA6,aadA2b,sul1,catB7,blaIMP-7,blaPAO,blaOXA-488,qacE" 98.8 Good 1 1 1 1 1 1 1 0 0 1 0 1

Pseudomonas aeruginosa strain BS2372 287 WGS BS2372 "CABIOT010000001,CABIOT010000002,CABIOT010000003,CABIOT010000004,CABIOT010000005,CABIOT010000006,CABIOT010000007" 113 6817226 ENVO:01000584 Indonesia Resistant Computational Prediction "fosA,aac(6')-Ib-cr,aph(3')-Iib,aac(3)-Ic,aac(6')-Ib3,aadA6,aadA2b,sul1,catB7,blaIMP-7,blaPAO,blaOXA-488,qacE" 98.9 Good 1 1 1 1 1 1 1 0 0 1 0 1

Pseudomonas aeruginosa strain BS2379 287 WGS BS2379 "CABIPE010000001,CABIPE010000002,CABIPE010000003,CABIPE010000004,CABIPE010000005,CABIPE010000006,CABIPE010000007" 100 6927576 ENVO:00003096 Indonesia Resistant Computational Prediction "crpP,aph(3'')-Ib,aac(3)-Id,fosA,aph(6)-Id,aph(3')-Iib,aph(3'')-Ib,,aph(3'')-Ib,aac(6')-Il,aph(3'')-Ib,dfrB5,sul2,catB7,blaPAO,blaOXA-488,blaVIM-2" 98.6 Good 1 1 1 1 1 1 1 0 0 1 0 1

Pseudomonas aeruginosa strain BS3413 287 WGS BS3413 "CABITO010000001,CABITO010000002,CABITO010000003,CABITO010000004,CABITO010000005,CABITO010000006,CABITO010000007" 74 7049171 ENVO:01000486 Indonesia Resistant Computational Prediction "crpP,aac(6')-Ib-cr,fosA,aph(3')-Ia,aac(6')-Ib3,ant(2'')-Ia,aph(3')-Iib,sul1,catB7,blaLCR-1,blaOXA-5-,blaPAO,qacE" 97.9 Good 1 1 0 0 1 1 1 0 0 1 0 1

Pseudomonas aeruginosa strain BS3412 287 WGS BS3412 "CABITQ010000001,CABITQ010000002,CABITQ010000003,CABITQ010000004,CABITQ010000005,CABITQ010000006,CABITQ010000007" 72 7048988 ENVO:01000486 Indonesia Resistant Computational Prediction "crpP,aac(6')-Ib-cr,aac(6')-Ib3,aph(3')-Iib,ant(2'')-Ia,aph(3')-Ia,fosA,sul1,catB7,blaOXA-50,blaPAO,blaLCR-1,qacE " Good 1 1 0 1 1 1 1 0 0 1 0 1

Pseudomonas aeruginosa strain BS3414 287 WGS BS3414 "CABITR010000001,CABITR010000002,CABITR010000003,CABITR010000004,CABITR010000005,CABITR010000006,CABITR010000007" 97 6873700 ENVO:01000486 Indonesia Resistant Computational Prediction "crpP,fosA,catB7,blaPAO,blaOXA-488,aph(3')-Iib" 99 Good 1 1 0 1 1 1 1 0 0 0 0 1

Pseudomonas aeruginosa AZPAE14926 287 WGS AZPAE14926 JTRP00000000 77 6886320 urinary tract infection Brazil "Human, Homo sapiens" Resistant;Susceptible AMR Panel "crpP,aac(6')-Ib-cr,aph(6)-Id,aph(3')-Iib,,aph(3'')-Ib,aac(6')-Ib3,aph(3')-XV,aph(3')-XV,fosA,sul1,sul2,catB7,blaPAO,blaOXA-486,qacE" 98.5 99.4 Good 1 1 0 1 1 1 1 0 0 0 0 1

Pseudomonas aeruginosa AZPAE14923 287 WGS AZPAE14923 JTRS00000000 104 6724711 respiratory tract infection Brazil "Human, Homo sapiens" Resistant AMR Panel "crpP,aac(6')-Ib-cr,aph(3')-Iib,aac(6')-Ib3,rmtD,aadA7,fosA,sul1,catB7,cmx,blaOXA-494,blaPAO,blaOXA-56,blaOXA-396,blaSPM-1,qacE" 99.1 99.7 Good 1 1 1 1 1 1 1 0 0 1 0 1

Pseudomonas aeruginosa AZPAE14922 287 WGS AZPAE14922 JTRT00000000 138 6839077 respiratory tract infection France "Human, Homo sapiens" Resistant AMR Panel "crpP,aac(6')-Il,aph(3')-IIbaadA2,fosA,aac(3)-Id,dfrB5,tet(G),catB7,cmlA1,blaVIM-2,blaOXA-4,blaPAO,blaOXA-486,qacE" 98.7 98.8 Good 1 1 1 1 1 1 1 0 1 0 0 1

Pseudomonas aeruginosa AZPAE14920 287 WGS AZPAE14920 JTRV00000000 104 6882761 urinary tract infection Spain "Human, Homo sapiens" Resistant;Susceptible AMR Panel "crpP,fosA,catB7,blaPAO,blaOXA-488,aph(3')-Iib" 98.6 100 Good 1 1 0 1 1 1 1 0 0 0 0 1

Pseudomonas aeruginosa strain PA28 287 WGS PA28 VMNO01000000 434 7037911 Lebanon "Human, Homo sapiens" Resistant Computational Prediction "crpP,aac(3)-Id,aph(6)-Id,aph(3'')-Ib,aph(3'')-Ib,aadA2,aac(6')-Il,fosA,dfrB5,aadA2,cmlA1,tet(G),catB7,blaVIM-2,blaOXA-4,blaPAO,qacE" 96.1 Good 1 1 1 1 1 1 1 0 1 1 0 1

Pseudomonas aeruginosa strain PA16 287 WGS PA16 VMNN01000000 414 7030390 the same hospital Lebanon "Human, Homo sapiens" Resistant Computational Prediction "crpP,aph(3')-Iib,aph(3'')-Ib,aadA2,aac(6')-Il,aac(3)-Id,aph(6)-Id,dfrB5,fosA,cmlA1,tet(G),catB7,blaOXA-4,blaOXA-486,blaVIM-2,blaPAO,qacE" 96.7 Good 1 1 1 1 1 1 1 0 1 1 0 1

Pseudomonas aeruginosa strain PA09 287 WGS PA09 VMNM01000000 464 6818344 the same hospital Lebanon "Human, Homo sapiens" Resistant Computational Prediction "crpP,fosA,aph(3')-Iib,tet(G),catB7,,cmlA1,blaPAO,blaOXA-4,blaOXA-486,qacE" 94.7 Good 1 1 0 1 1 1 1 0 1 0 0 1

Pseudomonas aeruginosa strain PA138 287 WGS PA138 VMNQ01000000 216 7199106 Lebanon "Human, Homo sapiens" Resistant Computational Prediction "crpP,aac(6')-Ib-cr,aac(6')-Ib-Hangzhou,aph(3')-Iib,fosA,sul1,catB7,blaPAO,blaOXA-395,qacE" 97.3 Good 1 1 1 1 1 1 1 0 0 0 0 1

Pseudomonas aeruginosa strain PA152 287 WGS PA152 VMNS01000000 795 6916524 Lebanon "Human, Homo sapiens" Resistant Computational Prediction "aadA13,aph(3')-Iib,aph(3'')-Ib,fosA,sul1,aph(6)-Id,ant(2'')-Ia,tet(A),catB7,blaPAO,blaGES-1,blaOXA-396,blaIMP-15qacE," 93.1 Good 1 1 1 1 1 1 1 0 1 1 0 0

Pseudomonas aeruginosa strain PA43 287 WGS PA43 VMNU01000000 189 6647372 Lebanon "Human, Homo sapiens" Resistant Computational Prediction "crpP,aph(3')-Iib,catB7,blaPAO,blaOXA-488,fosA," 99 Good 1 1 0 1 1 1 1 0 0 1 0 1

Pseudomonas aeruginosa strain PA45 287 WGS PA45 VMNV01000000 120 7009409 Lebanon "Human, Homo sapiens" Resistant Computational Prediction "crpP,aph(3')-Iib,aph(6)-Id,aac(3)-Id,aph(3'')-Ib,aac(6')-Il,aadA2,fosA,dfrB5,tet(G),catB7,,cmlA1,blaVIM-2,blaPAO,blaOXA-486,blaOXA-4,qacE," 98.6 Good 1 1 1 1 1 1 1 0 1 1 0 1

Pseudomonas aeruginosa strain PA59 287 Complete PA59 "CP024630,CP024631" 2 6972990 lung China Mink Resistant Computational Prediction "crpP,aac(3)-Iid,fosA,aph(3')-Iib,aac(6')-Iia,aadA4,tet(G),sul1,catB7,blaOXA-494,blaPAO,blaOXA-396,blaOXA-50,qacE" 98.6 Good 1 1 1 1 1 1 1 0 1 1 0 1

Pseudomonas aeruginosa AZPAE14917 287 WGS AZPAE14917 JTRY00000000 65 6308307 itra-abdominal tract infection Spain "Human, Homo sapiens" Resistant;Susceptible AMR Panel "fosA,catB7,blaPAO,blaOXA-396,blaOXA-494,aph(3')-Iib" 99.6 99.7 Good 1 1 1 1 1 1 1 0 0 0 0 0

Pseudomonas aeruginosa strain AG1 287 Complete AG1 CP045739 1 7190208 Sputum-lungs Costa Rica "Human, Homo sapiens" Resistant Computational Prediction "crpP,aac(6')-Ib-cr,fosA,aac(6')-29b,aac(6')-29a,aph(3')-Iib,aac(6')-Ib-Hangzhou,sul1,catB7,blaIMP-18,blaPAO,blaVIM-2,blaOXA-2,blaOXA-395,qacE" 98.8 Good 1 1 1 1 1 1 1 0 0 0 0 1

Pseudomonas aeruginosa strain ST773 287 Complete ST773 CP041945 1 6835731 urine USA "Human, Homo sapiens" Resistant Computational Prediction "aadA10,qnrVC1,aph(3')-Iib,rmtb,fosA,tet(G),sul1,catB7,blaNDM-1,blaPAO,blaOXA-395,qacE" 99.1 Good 1 1 1 1 1 1 1 0 1 1 0 1

Pseudomonas aeruginosa AZPAE14916 287 WGS AZPAE14916 JTRZ00000000 93 6242056 respiratory tract infection Spain "Human, Homo sapiens" Resistant;Intermediate;Susceptible AMR Panel "catB7,aph(3')-Iib,blaOXA-396,blaOXA-494,blaPAO,fosA" 98.9 99.7 Good 1 1 1 1 1 1 1 0 0 0 0 0

Pseudomonas aeruginosa AZPAE14915 287 WGS AZPAE14915 JTSA00000000 122 6880572 urinary tract infection Spain "Human, Homo sapiens" Susceptible;Resistant;Intermediate AMR Panel "crpP,fosA,catB7,blaPAO,blaOXA-488,aph(3')-Iib" 99.3 100 Good 1 1 1 1 1 1 1 0 0 0 0 1

Pseudomonas aeruginosa AZPAE14914 287 WGS AZPAE14914 JTSB00000000 105 6858678 urinary tract infection Spain "Human, Homo sapiens" Susceptible;Resistant AMR Panel "crpP,fosA,aac(6')-Ib-cr,blaPAO,aac(6')-Ib3,aph(3'')-Ib,aph(3')-XV,aph(6)-Id,aph(3')-Iib,sul1,sul2,catB7,qacE,blaOXA-486" 98.4 99.4 Good 1 1 0 0 1 1 1 0 0 0 0 1

Pseudomonas aeruginosa AZPAE14909 287 WGS AZPAE14909 JTSG00000000 126 7056926 itra-abdominal tract infection Spain "Human, Homo sapiens" Resistant AMR Panel "crpP,fosA,catB7,blaPAO,aph(3')-Iib,ant(2'')-Ia,blaOXA-50,qacE" 98.8 99.1 Good 1 1 1 1 1 1 1 0 0 1 0 1

Pseudomonas aeruginosa AZPAE14907 287 WGS AZPAE14907 JTSI00000000 87 6392112 itra-abdominal tract infection China "Human, Homo sapiens" Intermediate;Susceptible AMR Panel "fosA,catB7,blaPAO,blaOXA-488,aph(3')-Iib" 98.8 100 Good 1 1 0 1 1 1 1 0 0 0 0 0

Pseudomonas aeruginosa AZPAE14906 287 WGS AZPAE14906 JTSJ00000000 86 6690011 respiratory tract infection Germany "Human, Homo sapiens" Resistant AMR Panel "crpP,fosA,aph(3')-Iib,catB7,blaOXA-488,blaPAO," 98.7 100 Good 1 1 0 1 1 1 1 0 0 1 0 1

Pseudomonas aeruginosa strain PA219 287 WGS PA219 WOAF01000000 163 7456853 eye India "Human, Homo sapiens" Resistant Computational Prediction "crpP,aac(6')-Ib-cr,qnrVC1,fosA,tet(G),aph(3')-Iib,aadA1,aph(6)-Id,rmtb,aac(6')-Ib3,sul1,blaPAU-1,catB7,blaVIM-2,blaOXA-488,blaTEM-1B,blaPAO,blaOXA-10,mph(A),mph€,msr€,qacE" 95.6 Good 1 1 1 1 1 1 1 1 1 1 0 1

Pseudomonas aeruginosa strain PA202 287 WGS PA202 WOAK01000000 352 7192476 eye India "Human, Homo sapiens" Resistant Computational Prediction "crpP,aac(6')-Ib-cr,qnrVC1,fosA,tet(G),sul1,aadA10,aadA1,aph(6)-Id,aac(3)-Iid,aph(3'')-Ib,,aac(6')-Ib3,aac(6')-Ib-cr,aph(3')-Iib,catB7,blaLCR-1,blaPAO,blaOXA-395,qacE" 94.9 Good 1 1 1 1 1 1 1 0 1 1 0 1

Pseudomonas aeruginosa strain PA217 287 WGS PA217 WOAH01000000 127 6886566 eye India "Human, Homo sapiens" Resistant Computational Prediction "crpP,aph(6)-Id,aph(3'')-Ib,fosA,aph(3')-Iib,sul1,tet(G),catB7,blaPAO,blaOXA-488" 95.9 Good 1 1 0 1 1 1 1 0 1 0 0 1

Pseudomonas aeruginosa strain PA220 287 WGS PA220 WOAE01000000 87 6653669 eye India "Human, Homo sapiens" Resistant Computational Prediction "crpP,fosA,aph(3')-Iib,aph(3'')-Ib,aph(6)-Idsul1,catB7,blaPAO,blaOXA-395" 95.3 Good 1 1 1 1 1 1 1 0 0 0 0 1

Pseudomonas aeruginosa strain PA198 287 WGS PA198 WOAL01000000 118 7101775 eye India "Human, Homo sapiens" Resistant Computational Prediction "crpP,qnrVC1,aph(6)-Id,rmtD2,aph(3'')-Ib,aph(3')-VI,aph(3')-IIbfosA,sul1,tet(G),catB7,blaPME-1,blaOXA-488,blaPAO," 95.4 Good 1 1 0 1 1 1 1 0 1 1 0 1

Pseudomonas aeruginosa strain PA834 287 WGS PA834 VRZB01000000 487 6722504 blood Brazil "Human, Homo sapiens" Resistant Computational Prediction "fosA,aadA1b,aac(6')-Ilaph(3')-Iib,sul1,catB7,blaVIM-2,blaPAO,blaOXA-50,qacE" 91.6 Good 1 1 1 1 1 1 1 0 0 1 0 0

Pseudomonas aeruginosa AZPAE14900 287 WGS AZPAE14900 JTSP00000000 168 6851898 itra-abdominal tract infection India "Human, Homo sapiens" Resistant;Susceptible AMR Panel "crpP,msr€,dfrA1,dfrB2,sul1,fosA,mph€,aadA1,ant(2'')-Ia,aph(3')-Iib,aac(6')-Il,aph(3')-Via,tet(A),tet(G),floR,catB7,blaVIM-5,blaOXA-10,blaPAO,blaVEB-1,blaOXA-50" 98.7 99.7 Good 1 1 1 1 1 1 1 1 1 1 0 1

Pseudomonas aeruginosa strain PA221 287 WGS PA221 WOAD01000000 281 7205091 eye India "Human, Homo sapiens" Resistant Computational Prediction "crpP,aac(6')-Ib-cr,qnrVC1,aac(3)-Iid,aph(3')-Iib,rmtD2,aadA10,aac(6')-Ib3,aph(6)-Id,aadA1,aph(3'')-Ib,tet(G),sul1,blaPAO,catB7,blaOXA-395,blaLCR-1,qacE,fosA" 94.5 Good 1 1 1 1 1 1 1 0 1 1 0 1

Pseudomonas aeruginosa strain PA120 287 WGS PA120 VMNY01000000 122 6624004 Patients Lebanon "Human, Homo sapiens" Resistant Computational Prediction "fosA,catB7,blaOXA-395,blaPAO,aph(3')-Iib" 95.9 Good 1 1 1 1 1 1 1 0 0 0 0 0

Pseudomonas aeruginosa strain P28 287 WGS P28 VTFC01000000 201 6801376 Dammam Saudi Arabia "Human, Homo sapiens" Resistant Computational Prediction "crpP,aadA6,aac(6')-Il,fosA,sul1,aph(3')-Iib,tet(A),blaPAO,blaVEB-1,qacE,blaOXA-488" 94.7 Good 1 1 0 1 1 1 0 0 1 1 0 1

Pseudomonas aeruginosa strain P30 287 WGS P30 VTFB01000000 199 6778388 Dammam Saudi Arabia "Human, Homo sapiens" Resistant Computational Prediction "crpP,fosA,aph(3')-Iib,aac(6')-Ilaac(3)-Ic,aadA6,tet(A),sul1,cmlA1,blaVEB-1,blaPAO,blaOXA-488,qacE" 95.4 Good 1 1 1 1 1 1 1 0 1 1 0 1

Pseudomonas aeruginosa strain P13 287 WGS P13 VTFE01000000 151 6741877 Dammam Saudi Arabia "Human, Homo sapiens" Resistant Computational Prediction "crpP,fosA,aph(3')-Iib,aac(6')-Il,aadA1,ant(2'')-Ia,sul1,dfrB2,tet(A),catB7,blaVEB-1,blaPAO,blaOXA-10,qacE,blaOXA-50," 95.2 Good 1 1 0 1 1 1 1 0 1 1 0 1

Pseudomonas aeruginosa strain P2 287 WGS P2 VTFD01000000 154 6641132 Dammam Saudi Arabia "Human, Homo sapiens" Resistant Computational Prediction "crpP,fosA,catB7,aph(3')-Iib,blaPAO,blaOXA-395" 95.5 Good 1 1 1 1 1 1 1 0 0 0 0 1

Pseudomonas aeruginosa strain PA-50010278 287 WGS PA-50010278 WSYN01000000 84 6821270 surgical site USA "Human, Homo sapiens" Resistant Computational Prediction "rmtB,aadA10,qnrVC1,aph(3')-Iib,fosA,tet(G),sul1,catB7,blaNDM-1,blaPAO,blaOXA-395,qacE" 96.2 Good 1 1 1 1 1 1 1 0 1 1 0 1

Pseudomonas aeruginosa AZPAE14892 287 WGS AZPAE14892 JTSW00000000 124 6609151 respiratory tract infection France "Human, Homo sapiens" Resistant AMR Panel "catB7,fosA,aph(3')-Iib,blaOXA-50,blaPAO," 98.4 99.1 0.3 Good 1 1 0 1 1 1 1 0 0 0 0 0

Pseudomonas aeruginosa strain UMB0501 287 WGS UMB0501 WUDN01000000 158 5999852 urine USA "Human, Homo sapiens" Resistant Computational Prediction "catB7,fosA,aph(3')-Iib,blaOXA-395,blaPAO," 95 Good 1 1 1 1 1 1 1 0 0 0 0 0

Pseudomonas aeruginosa strain UMB0802 287 WGS UMB0802 WUDL01000000 181 6841797 kidney stone USA "Human, Homo sapiens" Resistant Computational Prediction "crpP,aph(3')-Iib,aadA6,blaOXA-494,fosA,sul1,catB7,blaPAO,blaOXA-396,qacE" 95.3 Good 1 1 1 1 1 1 1 0 0 0 0 1

Pseudomonas aeruginosa strain UMB1204 287 WGS UMB1204 WUDK01000000 163 6270797 urine USA "Human, Homo sapiens" Resistant Computational Prediction "catB7,aph(3')-Iib,blaOXA-50,blaPAO,fosA" 95.8 Good 1 1 0 1 1 1 1 0 0 0 0 0

Pseudomonas aeruginosa AZPAE14890 287 WGS AZPAE14890 JTSY00000000 112 7016044 urinary tract infection France "Human, Homo sapiens" Resistant AMR Panel "crpP,aph(3')-Iib,catB7,blaPAO,blaOXA-488,fosA" 98.9 99.7 Good 1 1 0 1 1 1 1 0 0 0 0 1

Pseudomonas aeruginosa strain UMB0801 287 WGS UMB0801 WUDM01000000 231 6841150 urine USA "Human, Homo sapiens" Resistant Computational Prediction "crpP,aadA6,aph(3')-Iib,fosA,sul1,catB7,blaOXA-494,blaPAO,qacE,blaOXA-396," 94.5 Good 1 1 1 1 1 1 1 0 0 0 0 1

Pseudomonas aeruginosa strain VNMU144 287 WGS VNMU144 WXZV01000000 114 6864073 wound Ukraine "Human, Homo sapiens" Resistant Computational Prediction "crpP,qnrVC1,aac(6')-Ib-cr,aph(6)-Id,aph(3'')-Ib,aac(6')-Ib3,aph(3')-Iib,fosA,ant(2'')-Ia,dfrA1,sul1,catB7,blaPAO,blaOXA-10,blaOXA-488,blaIMP-34,qacE" 96.1 Good 1 1 1 1 1 1 1 0 0 1 0 1

Pseudomonas aeruginosa strain VNMU149 287 WGS VNMU149 WXZS01000000 116 6455613 wound Ukraine "Human, Homo sapiens" Resistant Computational Prediction "crpP,aac(6')-Ib-cr,aph(3'')-Ib,aac(6')-Ib3,aph(6)-Id,aph(3')-Iib,aac(6')-Il,aac(6')-Ib-cr,fosA,sul1,catB7,cmx,blaOXA-494,blaPAO" Good 1 1 1 1 1 1 1 0 0 0 0 1

Pseudomonas aeruginosa strain VNMU145 287 WGS VNMU145 WXZU01000000 107 6784500 wound Ukraine "Human, Homo sapiens" Resistant Computational Prediction "crpP,fosA,aph(3'')-Ib,aph(3')-Ia,aac(6')-Il,aph(6)-Id,aph(3')-Iib,sul1,tet(A),catB7,blaVEB-1,blaPAO,blaOXA-50,qacE" 95.6 Good 1 1 0 1 1 1 1 0 1 0 0 1

Pseudomonas aeruginosa strain VNMU143 287 WGS VNMU143 WXZW01000000 139 6945863 wound Ukraine "Human, Homo sapiens" Resistant Computational Prediction "crpP,qnvVC1,aac(6')-Ib-cr,aph(3')-Iib,aph(6)-Id,aph(3'')-Ib,ant(2'')-Ia,aac(6')-Ib3,fosA,sul1,dfrA1,catB7,blaIMP-34,blaOXA-488,blaPAO,qacE,blaOXA-10" 96 Good 1 1 1 1 1 1 1 0 0 1 0 1

Pseudomonas aeruginosa strain PA4722 287 WGS PA4722 VKOE01000000 67 6798564 Rectal Swab Brazil "Human, Homo sapiens" Resistant Computational Prediction "crpP,aac(6')-Ib-cr,fosA,rmtD,aph(3')-Iib,aac(6')-Ib3,aadA7,cmx,catB7,sul1,blaOXA-396,blaPAO,blaOXA-494,blaOXA-50,blaSPM-1,qacE" 95.9 Good 1 1 1 1 1 1 1 0 0 1 0 1

Pseudomonas aeruginosa strain INP-43 287 Complete INP-43 CP047592 1 6335031 cystic fibrosis pediatric isolate Mexico "Human, Homo sapiens" Resistant Computational Prediction "crpP,aph(3')-Iib,catB7,blaOXA-396,blaPAO,blaOXA-494,fosA" 96.4 Good 1 1 1 1 1 1 1 0 0 0 0 1

Pseudomonas aeruginosa strain BCW_7430 287 WGS BCW_7430 SDSQ01000000 534 6383517 Blowhole USA "Bottlenose dolphin, Tursiops truncatus" Resistant Computational Prediction "catB7,aph(3')-Iib,blaOXA-395,blaPAO,fosA" 91.8 Good 1 1 1 1 1 1 1 0 0 1 0 0

Pseudomonas aeruginosa strain BCW_7427 287 WGS BCW_7427 SDXM01000000 81 6360760 BAL USA "Bottlenose dolphin, Tursiops truncatus" Resistant Computational Prediction "catB7,aph(3')-Iib,blaOXA-395,blaPAO,fosA," 95.9 Good 1 1 1 1 1 1 1 0 0 1 0 0

Pseudomonas aeruginosa AZPAE14887 287 WGS AZPAE14887 JTTB00000000 114 6903570 itra-abdominal tract infection Croatia "Human, Homo sapiens" Resistant AMR Panel "crpP,aac(6')-Ib-cr,aac(6')-Ib3,aadA6,aac(6')-Il,aph(3')-Iib,aac(6')-31,fosA,sul1,catB7,blaPAO,blaOXA-488,blaOXA-2,qacE" 99.1 99.7 0.3 Good 1 1 0 1 1 1 1 0 0 1 0 1

Pseudomonas aeruginosa AZPAE14886 287 WGS AZPAE14886 JTTC00000000 135 7057811 urinary tract infection Croatia "Human, Homo sapiens" Resistant AMR Panel "crpP,fosA,blaPAO,aac(6')-Ib-cr,aac(6')-Ib3,aadA2b,sul1,catB7,aph(3')-Iib,blaOXA-395,qacE,blaCARB-2" 98.7 100 Good 1 1 1 1 1 1 1 0 0 0 0 1

Pseudomonas aeruginosa AZPAE14884 287 WGS AZPAE14884 JTTE00000000 44 6434371 respiratory tract infection Croatia "Human, Homo sapiens" Resistant;Susceptible AMR Panel "crpP,fosA,catB7,blaPAO,aph(3')-Iib,blaOXA-395" 99.1 99.7 Good 1 1 0 1 1 1 1 0 0 0 0 1

Pseudomonas aeruginosa AZPAE14882 287 WGS AZPAE14882 JTTG00000000 73 6455645 respiratory tract infection Spain "Human, Homo sapiens" Resistant;Susceptible AMR Panel "catB7,aph(3')-Iib,fosA,blaPAO,blaOXA-486" 99.1 100 Good 1 1 0 1 1 1 1 0 0 0 0 0

Pseudomonas aeruginosa AZPAE14879 287 WGS AZPAE14879 JTTJ00000000 50 6226729 respiratory tract infection United States "Human, Homo sapiens" Resistant AMR Panel "fosA,catB7,blaPAO,blaOXA-396,aph(3')-Iib" 99.4 100 Good 1 1 1 1 1 1 1 0 0 0 0 0

Pseudomonas aeruginosa AZPAE14878 287 WGS AZPAE14878 JTTK00000000 151 6953921 itra-abdominal tract infection United States "Human, Homo sapiens" Resistant AMR Panel "crpP,fosA,aph(3')-Iib,tet(G),catB7,,blaPAO,blaOXA-488" 98.1 99.4 0.6 Good 1 1 0 1 1 1 1 0 1 1 0 1

Pseudomonas aeruginosa AZPAE14877 287 WGS AZPAE14877 JTTL00000000 64 6300065 respiratory tract infection United States "Human, Homo sapiens" Susceptible;Intermediate AMR Panel "catB7,aph(3')-Iib,fosA,blaPAO,blaOXA-50" 99.1 100 0.3 Good 1 1 0 1 1 1 1 0 0 0 0 0

Pseudomonas aeruginosa strain CF39S 287 Complete CF39S "CP045916,CP045917" 2 7266549 lung USA "Human, Homo sapiens" Resistant Computational Prediction "crpP,aac(6')-Ib-cr,tet(G),fosA,aph(3'')-Ib,aac(6')-Ib3,aph(6)-Id,aph(3')-Iib,aac(6')-Ib-cr,sul1,catB7,blaOXA-50,blaPAO" 95 Good 1 1 0 1 1 1 1 0 1 0 0 1

Pseudomonas aeruginosa strain ST235 287 WGS ST235 VLOE01000000 157 6948122 hospital Italy "Human, Homo sapiens" Resistant Computational Prediction "aadA6,aac(6')-Il,aph(3')-Iib,aac(6')-31,aph(3')-Iib,Gar,fosA,sul1,catB7,catB3,blaVIM-1,blaPAO,blaOXA-2,blaOXA-488,qacE" 95.7 Good 1 1 1 1 1 1 1 0 0 0 0 0

Pseudomonas aeruginosa strain CPa12-1 287 WGS CPa12-1 VCOU01000000 46 6745488 sputum China "Human, Homo sapiens" Resistant Computational Prediction "crpP,aac(6')-Ib-cr,aph(3')-Ia,aph(3')-Iib,aac(6')-Ib3,aadA2b,tet©,fosA,sul1,dfrA1,catB7,cmlA1,blaOXA-395,blaPAO,qacE," 96.2 Good 1 1 1 1 1 1 1 0 1 0 0 1

Pseudomonas aeruginosa strain CPa40 287 WGS CPa40 VCOX01000000 80 7322213 sputum China "Human, Homo sapiens" Resistant Computational Prediction "crpP,msr€,aac(6')-Ib-cr,aac(6')-Ib3,aph(3')-Ia,aph(3')-Iib,sul1,mph€,tet©,catB7,catB3,blaIMP-45,blaOXA-1,blaOXA-488,blaPAO,qacE,fosA" 95 Good 1 1 1 1 1 1 1 1 1 1 0 1

Pseudomonas aeruginosa strain CPa12-2 287 WGS CPa12-2 VCOV01000000 49 6685338 Bronchoalveolar lavage fluid China "Human, Homo sapiens" Resistant Computational Prediction "fosA,aac(6')-Ib-cr,aadA2b,tet©,dfrA1,sul1,aph(3')-Iib,aac(6')-Ib3,aph(3')-Ia,catB7,cmlA1blaOXA-395,blaPAO,qacE," 96.2 Good 1 1 1 1 1 1 1 1 1 0 0 1

Pseudomonas aeruginosa strain NSPa12 287 WGS NSPa12 VCOT01000000 51 6687303 nasopharynx China "Human, Homo sapiens" Resistant Computational Prediction "aadA2b,aac(6')-Ib-cr,aac(6')-Ib3,aph(3')-Ia,aph(3')-Iib,dfrA1,tet©,sul1,cmlA1,catB7,blaPAO,blaOXA-395,qacE,fosA" 96.2 Good 1 1 1 1 1 1 1 0 1 0 0 1

Pseudomonas aeruginosa strain PcyII-40 287 Complete PcyII-40 LR739069 1 6831274 clinical France "Human, Homo sapiens" Resistant Computational Prediction "fosA,catB7,blaPAO,aph(3')-Iib,blaOXA-50" 95.9 Good 1 1 0 1 1 1 1 0 0 1 0 0

Pseudomonas aeruginosa AZPAE14872 287 WGS AZPAE14872 JTTQ00000000 140 7250921 respiratory tract infection Argentina "Human, Homo sapiens" Resistant AMR Panel "crpP,fosA,blaPAO,catB7,aac(6')-Ib-cr,aph(6)-Id,aph(3'')-Ib,aph(3'')-Ib,aac(6')-Ib3,aph(3')-Iib,sul1,cmx,blaOXA-494,blaOXA-2,blaOXA-396,qacE," 98.2 99.4 0.3 Good 1 1 1 1 1 1 1 0 0 0 0 1

Pseudomonas aeruginosa strain NICED-PA-01 287 WGS NICED-PA-01 "JAACJB010000010,JAACJB010000011,JAACJB010000012,JAACJB010000013,JAACJB010000014,JAACJB010000015,JAACJB010000016" 75 6910564 stool India "Human, Homo sapiens" Resistant Computational Prediction "qnrVC1,msr€,crpP,fosA,dfrA5,ere(A),mph€,aadA10,aph(6)-Id,rmtB,aph(3'')-Ib,aph(3')-Iib,tet(G),floR,catB7,blaPAO,blaOXA-395,blaDIM-1,blaNDM-1,qacE" 95.6 Good 1 1 1 1 1 1 1 1 1 1 0 1

Pseudomonas aeruginosa strain Pa-3 287 WGS Pa-3 "JAATVZ010000100,JAATVZ010000101,JAATVZ010000102,JAATVZ010000103,JAATVZ010000104,JAATVZ010000105,JAATVZ010000106" 125 7017375 tracheal aspirate Pakistan "Human, Homo sapiens" Resistant Computational Prediction "crpP,aph(6)-Id,aadA13,aph(3')-Iib,aph(3'')-Ib,ant(2'')-Ia,aac(6')-Il,tet(X),fosA,sul1,dfrA5,catB7,ARR-8,blaPAO,blaOXA-2,blaOXA-395,blaIMP-34,ere(A),qacE" 95.6 Good 1 1 1 1 1 1 1 1 1 1 0 1

Pseudomonas aeruginosa strain 99 287 WGS 99 "CACPEP010000001,CACPEP010000002,CACPEP010000003,CACPEP010000004,CACPEP010000005,CACPEP010000006,CACPEP010000007" 83 6372765 throat swab Indonesia "Human, Homo sapiens" Resistant Computational Prediction "fosA,catB7,aph(3')-Iib,blaPAO,blaOXA-488" 96.4 Good 1 1 0 1 1 1 1 0 0 0 0 0

Pseudomonas aeruginosa strain 223 287 WGS 223 "CACPET010000001,CACPET010000002,CACPET010000003,CACPET010000004,CACPET010000005,CACPET010000006,CACPET010000007" 129 6580949 throat swab Indonesia "Human, Homo sapiens" Resistant Computational Prediction "fosA,catB7,aph(3')-Iib,blaPAO,blaOXA-488,crpP" 95.1 Good 1 1 0 1 1 1 1 0 0 1 0 1

Pseudomonas aeruginosa strain PBIO724 287 WGS PBIO724 "CADCYC010000001,CADCYC010000002,CADCYC010000003,CADCYC010000004,CADCYC010000005,CADCYC010000006,CADCYC010000007" 88 6836681 "tertiary hospital, surgery" Rwanda Musca domestica Resistant Computational Prediction "crpP,fosA,catB7,blaPAO,blaOXA-396,aph(3')-Iib" 96 Good 1 1 1 1 1 1 1 0 0 0 0 1

Pseudomonas aeruginosa strain PBIO712 287 WGS PBIO712 "CADCYH010000001,CADCYH010000002,CADCYH010000003,CADCYH010000004,CADCYH010000005,CADCYH010000006,CADCYH010000007" 111 7100306 "tertiary hospital, gynecology" Rwanda "crpP,fosA,catB7,blaPAO,blaOXA-396,aph(3')-Iib" Resistant Computational Prediction "crpP,fosA,catB7,blaPAO,blaOXA-396,aph(3')-Iib" 95.6 Good 1 1 1 1 1 1 1 0 0 0 0 1

Pseudomonas aeruginosa AZPAE14865 287 WGS AZPAE14865 JTTX00000000 133 6894960 respiratory tract infection India "Human, Homo sapiens" Resistant AMR Panel "crpP,msr€,aac(6')-Il,aph(3')-Iib,aadA1,ant(2'')-Ia,aph(3')-Via,fosA,mph€,dfrA1,dfrB2,tet(A),sul1,tet(G),floR,catB7,blaPAO,blaOXA-10,blaVIM-5,blaVEB-1,blaOXA-50," 99.1 100 Good 1 1 1 1 1 1 1 1 1 1 1 1

Pseudomonas aeruginosa AZPAE14862 287 WGS AZPAE14862 JTUA00000000 167 7067572 urinary tract infection India "Human, Homo sapiens" Susceptible;Resistant AMR Panel "crpP,aac(6')-Ib-cr,aac(6')-Ib3,aph(3')-Iib,fosA,sul1,catB7,blaIMP-13,blaPAO,blaOXA-50,qacE," 98.7 100 Good 1 1 1 1 1 1 1 0 0 0 0 1

Pseudomonas aeruginosa AZPAE14861 287 WGS AZPAE14861 JTUB00000000 110 6713012 itra-abdominal tract infection Spain "Human, Homo sapiens" Susceptible;Intermediate AMR Panel "crpP,fosA,aph(3')-Iib,sul1,catB7,blaOXA-494,blaPAO,blaOXA-396,qacE," 98.2 99.4 Good 1 1 1 1 1 1 1 0 0 0 0 1

Pseudomonas aeruginosa AZPAE14860 287 WGS AZPAE14860 JTUC00000000 93 6768731 itra-abdominal tract infection Spain "Human, Homo sapiens" Resistant AMR Panel "crpP,aac(6')-Ib-cr,aph(3'')-Ib,aac(6')-Ib-Hangzhou,aph(6)-Id,aph(3')-Iib,fosA,sul1,catB7,blaPAO,blaOXA-488,qacE," 98.8 100 Good 1 1 0 1 1 1 1 0 0 1 0 1

Pseudomonas aeruginosa AZPAE14853 287 WGS AZPAE14853 JTUI00000000 122 6829167 respiratory tract infection Brazil "Human, Homo sapiens" Resistant AMR Panel "rmtD,aac(6')-Ib-craac(6')-Ib3,aadA7,aph(3')-Iib,fosA,sul1,catB7,blaOXA-396,blapAO,blaOXA-56,blaSPM-1,blaOXA-494,qacE" 99.2 100 Good 1 1 1 1 1 1 1 0 0 1 0 1

Pseudomonas aeruginosa AZPAE14852 287 WGS AZPAE14852 JTUJ00000000 233 6860603 respiratory tract infection Brazil "Human, Homo sapiens" Resistant;Susceptible AMR Panel "crpP,aac(6')-Ib-cr,fosA,aph(3')-Iib,sul1,aac(6')-Ib3,catB7,blaPAO,blaOXA-17,blaOXA-486,qacE" 98.2 99.1 Good 1 1 0 1 1 1 1 0 0 0 0 1

Pseudomonas aeruginosa AZPAE14846 287 WGS AZPAE14846 JTUO00000000 133 6819636 respiratory tract infection France "Human, Homo sapiens" Resistant;Susceptible AMR Panel "crpP,fosA,aac(6')-Ib-cr,aph(3')-Iib,sul1,aac(6')-Ib3,blaPAO,blaOXA-395,blaCARB-2,qacE," 98.2 99.7 0.2 Good 1 1 1 1 1 1 0 0 0 0 0 1

Pseudomonas aeruginosa AZPAE14843 287 WGS AZPAE14843 JTUR00000000 125 6737478 urinary tract infection United States "Human, Homo sapiens" Resistant AMR Panel "fosA,aadA6,sul1,tet(G),catB7,blaPAO,blaOXA-488,qacE," 98.3 97.3 Good 1 1 0 1 0 1 1 0 1 0 0 0

Pseudomonas aeruginosa AZPAE14842 287 WGS AZPAE14842 JTUS00000000 80 6889407 urinary tract infection United States "Human, Homo sapiens" Resistant AMR Panel "aadA6,fosA,aph(3')-Iib,sul1,catB7,blaOXA-488,blaPAO,qacE" 98.8 100 0.6 Good 1 1 0 1 1 1 1 0 0 1 0 0

Pseudomonas aeruginosa AZPAE14840 287 WGS AZPAE14840 JTUU00000000 76 7049244 urinary tract infection China "Human, Homo sapiens" Susceptible;Intermediate;Resistant AMR Panel "crpP,fosA,aac(6')-Ib-cr,tet(G),aph(3')-Iib,aph(3'')-Ib,aph(6)-Id,aac(6')-Ib3,ant(2'')-Ia,sul1,catB7,blaOXA-11,blaPAO,blaOXA-494,blaOXA-14,blaOXA-396" 98.6 100 Good 1 1 1 1 1 1 1 0 1 1 0 1

Pseudomonas aeruginosa AZPAE14839 287 WGS AZPAE14839 JTUV00000000 76 6615080 itra-abdominal tract infection China "Human, Homo sapiens" Resistant;Susceptible AMR Panel "crpP,catB7,aph(3')-Iib,blaOXA-396,blaPAO,FosA,blaOXA-494,blaOXA-50" 99 100 Good 1 1 1 1 1 1 1 0 0 0 0 1

Pseudomonas aeruginosa AZPAE14838 287 WGS AZPAE14838 JTUW00000000 97 7113432 respiratory tract infection China "Human, Homo sapiens" Resistant;Intermediate;Susceptible AMR Panel "crpP,aac(6')-Ib-cr,fosA,sul1,tet(G),aph(3'')-Ib,aac(6')-Ib3,aph(6)-Id,ant(2'')-Ia,catB7,blaPAO,blaOXA-10,blaOXA-50," 99 100 Good 1 1 0 1 1 1 1 0 1 1 0 1

Pseudomonas aeruginosa AZPAE14836 287 WGS AZPAE14836 JTUY00000000 83 6438844 respiratory tract infection China "Human, Homo sapiens" Intermediate;Susceptible AMR Panel "crpP,fosA,catB7,blaPAO,blaOXA-494,blaOXA-50,blaOXA-396,aph(3')-Iib" 99 99.4 Good 1 1 1 1 1 1 1 0 0 0 0 1

Pseudomonas aeruginosa AZPAE14834 287 WGS AZPAE14834 JTVA00000000 149 6792091 urinary tract infection Argentina "Human, Homo sapiens" Resistant AMR Panel "crpP,aac(6')-Ib-cr,aph(3'')-Ib,fosA,aph(3'')-Ib,aph(3')-Iib,aph(6)-Id,aac(6')-Ib3,aac(6')-Il,sul1,cmx,catB7,blaOXA-494,blaPAO,blaOXA-2,blaOXA-396,qacE" 99.1 99.4 0.3 Good 1 1 1 1 1 1 1 0 0 0 0 1

Pseudomonas aeruginosa AZPAE14833 287 WGS AZPAE14833 JTVB00000000 148 6876687 urinary tract infection Argentina "Human, Homo sapiens" Resistant AMR Panel "crpP,aac(6')-Ib-cr,aac(6')-Ib3,aph(3')-IIb,aph(3'')-Ib,aac(6')-Il,aph(3'')-Ib,aph(6)-Id,fosA,sul1,cmx,catB7,blaOXA-2,blaOXA-494,blaOXA-210,blaPAO,blaOXA-396,qacE," 99 100 0.3 Good 1 1 1 1 1 1 1 0 0 0 0 1

Pseudomonas aeruginosa AZPAE14831 287 WGS AZPAE14831 JTVD00000000 149 7184792 respiratory tract infection Argentina "Human, Homo sapiens" Resistant AMR Panel "crpP,fosA,aac(6')-Ib-cr,aph(3')-Iib,aac(6')-Ib-Hangzhou,catB7,blaGES-1,blaPAO,blaOXA-494,blaOXA-396" 98.2 99.7 0.6 Good 1 1 1 1 1 1 1 0 0 0 0 1

Pseudomonas aeruginosa AZPAE14830 287 WGS AZPAE14830 JTVE00000000 150 6816534 itra-abdominal tract infection Argentina "Human, Homo sapiens" Resistant AMR Panel "fosA,aac(6')-Ib-cr,aph(3'')-Ib,ant(2'')-Ia,aph(6)-Id,aadA6,aac(6')-Ib3,aph(3')-Iib,sul1,dfrA5,catB3,catB7,cmx,blaOXA-17,blaOXA-129,blaPAO,blaOXA-488,qacE," 98.5 99.4 Good 1 0 1 1 1 1 1 0 0 1 0 1

Pseudomonas aeruginosa AZPAE14825 287 WGS AZPAE14825 JTVJ00000000 68 6426191 respiratory tract infection Germany "Human, Homo sapiens" Susceptible;Intermediate AMR Panel "fosA,catB7,blaPAO,blaOXA-395,aph(3')-Iib" 98.9 99.4 Good 1 1 1 1 1 1 1 0 0 0 0 0

Pseudomonas aeruginosa strain LYT4 287 Complete LYT4 "CP052759,CP052760" 2 6814016 tung meal China Resistant Computational Prediction "crpP,fosA,catB7,blaPAO,blaOXA-50,aph(3')-Iib" 95.9 Good 1 1 0 1 1 1 1 0 0 0 0 1

Pseudomonas aeruginosa AZPAE14822 287 WGS AZPAE14822 JTVM00000000 100 6668034 itra-abdominal tract infection Brazil "Human, Homo sapiens" Resistant AMR Panel "crpP,aac(6')-Ib-cr,fosA,aph(3')-Iib,aac(6')-Ib3,rmtD,aadA7,sul1,cmx,catB7,blaPAO,blaOXA-396,blaOXA-56,blaOXA-494,qacE," 98.7 99.7 0.3 Good 1 1 1 1 1 1 1 0 0 1 0 1

Pseudomonas aeruginosa AZPAE14821 287 WGS AZPAE14821 JTVN00000000 79 6811510 urinary tract infection Brazil "Human, Homo sapiens" Resistant AMR Panel "crpP,aac(6')-Ib3,fosA,aadA7,aac(6')-Ib-cr,rmtD,aph(3')-Iib,sul1,catB7,blaPAO,blaOXA-56,blaOXA-494,blaSPM-1,blaOXA-396,qacE" 99 100 Good 1 1 1 1 1 1 1 0 0 1 0 1

Pseudomonas aeruginosa strain GIMC5019:PA52Ts1 287 WGS GIMC5019:PA52Ts1 "CP051766,CP051767" 2 6860562 tracheal aspirate Russia "Human, Homo sapiens" Resistant Computational Prediction "crpP,aadA1,aph(3')-Iib,ant(2'')-Ia,aac(6')-Il,fosA,sul1,tet(A),tet(G),catB7,blaPAO,blaVEB-1,blaOXA-50,blaOXA-10,qacE," 95.8 Good 1 1 0 1 1 1 1 0 1 1 0 1

Pseudomonas aeruginosa strain GIMC5020:PA52Ts2 287 WGS GIMC5020:PA52Ts2 "CP051768,CP051769" 2 6814990 tracheal aspirate Russia "Human, Homo sapiens" Resistant Computational Prediction "crpP,fosA,ant(2'')-Ia,aac(6')-Il,aadA1,aph(3')-Iib,sul1,tet(A),tet(G),catB7,blaVEB-1,blaOXA-50,blapAO,blaOXA-10,qacE" 95.7 Good 1 1 0 1 1 1 1 0 1 1 0 1

Pseudomonas aeruginosa strain GIMC5021:PA52Ts17 287 WGS GIMC5021:PA52Ts17 "CP051770,CP051771" 2 6851706 tracheal aspirate Russia "Human, Homo sapiens" Resistant Computational Prediction "crpP,aadA12,aac(6')-Il,aph(3')-Iib,ant(2'')-Ia,fosA,sul1,tet(G),tet(A),catB7,blaVEB-1,blaOXA-50,blaOXA-10,blaPAO,qacE," 95.1 Good 1 1 0 1 1 1 1 0 1 1 0 1

Pseudomonas aeruginosa strain 3856 287 WGS 3856 "JABGNY010000001,JABGNY010000002,JABGNY010000011,JABGNY010000012,JABGNY010000013,JABGNY010000014,JABGNY010000015" 64 6412945 sputum United Kingdom "Human, Homo sapiens" Resistant Computational Prediction "fosA,catB7,blaPAO,blaOXA-50,aph(3')-Iib" 94.8 Good 1 1 0 1 1 1 1 0 0 0 0 0

Pseudomonas aeruginosa strain 2756 287 WGS 2756 "JABGOB010000001,JABGOB010000002,JABGOB010000011,JABGOB010000101,JABGOB010000102,JABGOB010000103,JABGOB010000104" 104 6428166 bronchoalveolar lavage United Kingdom "Human, Homo sapiens" Resistant Computational Prediction "fosA,catB7,blaPAO,blaOXA-50,aph(3')-Iib" 96.2 Good 1 1 0 1 1 1 1 0 0 0 0 0

Pseudomonas aeruginosa strain 811 287 WGS 811 "JABGNE010000001,JABGNE010000002,JABGNE010000011,JABGNE010000101,JABGNE010000102,JABGNE010000103,JABGNE010000104" 156 6729819 bronchoalveolar lavage United Kingdom "Human, Homo sapiens" Resistant Computational Prediction "crpP,aph(3')-Iib,catB7,blaOXA-485,fosA,blaPAO" 95.7 Good 1 1 0 1 1 1 1 0 0 1 0 1

Pseudomonas aeruginosa strain 179 287 WGS 179 "JABGNG010000001,JABGNG010000002,JABGNG010000011,JABGNG010000101,JABGNG010000102,JABGNG010000103,JABGNG010000104" 133 6621475 bronchoalveolar lavage United Kingdom "Human, Homo sapiens" Resistant Computational Prediction "aph(3')-Iib,catB7,blaOXA-485,fosA,blaPAO" 95.9 Good 1 1 0 1 1 1 1 0 0 1 0 0

Pseudomonas aeruginosa strain 160 287 WGS 160 "JABGNH010000001,JABGNH010000002,JABGNH010000011,JABGNH010000101,JABGNH010000102,JABGNH010000103,JABGNH010000104" 146 6632787 bronchoalveolar lavage United Kingdom "Human, Homo sapiens" Resistant Computational Prediction "aph(3')-Iib,catB7,blaOXA-485,fosA,blaPAO" 96 Good 1 1 0 1 1 1 1 0 0 1 0 0

Pseudomonas aeruginosa AZPAE14819 287 WGS AZPAE14819 JTVP00000000 98 6660543 urinary tract infection Brazil "Human, Homo sapiens" Resistant AMR Panel "crpP,aph(3')-Iib,catB7,blaOXA-486,fosA,blaPAO" 99 100 Good 1 1 0 1 1 1 1 0 0 0 0 1

Pseudomonas aeruginosa strain 3942 287 WGS 3942 "JABGNO010000001,JABGNO010000002,JABGNO010000011,JABGNO010000012,JABGNO010000013,JABGNO010000014,JABGNO010000015" 69 6259851 bronchoalveolar lavage United Kingdom "Human, Homo sapiens" Resistant Computational Prediction "crpP,aac(6')-Ib-cr,rmtD,aadA7,aph(3')-Iib,fosA,sul1,cmx,aac(6')-Ib3,catB7,blaOXA-396,blaPAO,blaOXA-56,blaSPM-1,blaOXA-494,qacE," 94 Good 1 1 1 1 1 1 1 0 0 1 0 1

Pseudomonas aeruginosa strain 3734 287 WGS 3734 "JABGNT010000001,JABGNT010000002,JABGNT010000011,JABGNT010000101,JABGNT010000102,JABGNT010000103,JABGNT010000104" 120 6281375 bronchoalveolar lavage United Kingdom "Human, Homo sapiens" Resistant Computational Prediction "crpP,catB7,aph(3')-Iib,blaOXA-486,blaPAO,fosA," 95.4 Good 1 1 0 1 1 1 1 0 0 0 0 1

Pseudomonas aeruginosa strain 3736 287 WGS 3736 "JABGNR010000001,JABGNR010000002,JABGNR010000011,JABGNR010000012,JABGNR010000013,JABGNR010000014,JABGNR010000015" 85 6320458 bronchoalveolar lavage United Kingdom "Human, Homo sapiens" Resistant Computational Prediction "fosA,aph(3')-Iib,catB7,blaPAO,blaOXA-50," 94.8 Good 1 1 0 1 1 1 1 0 0 0 0 0

Pseudomonas aeruginosa strain 3732 287 WGS 3732 "JABGNU010000001,JABGNU010000002,JABGNU010000011,JABGNU010000101,JABGNU010000102,JABGNU010000103,JABGNU010000104" 107 6179053 bronchoalveolar lavage United Kingdom "Human, Homo sapiens" Resistant Computational Prediction "crpP,catB7,aph(3')-Iib,blaOXA-486,blaPAO,fosA," 94.9 Good 1 1 0 1 1 1 1 0 0 0 0 1

Pseudomonas aeruginosa strain 3857 287 WGS 3857 "JABGNX010000001,JABGNX010000002,JABGNX010000011,JABGNX010000101,JABGNX010000102,JABGNX010000103,JABGNX010000104" 110 6429765 sputum United Kingdom "Human, Homo sapiens" Resistant Computational Prediction "fosA,aph(3')-Iib,catB7,blaPAO,blaOXA-50," 96.1 Good 1 1 0 1 1 1 1 0 0 0 0 0

Pseudomonas aeruginosa strain 2755 287 WGS 2755 "JABGOC010000001,JABGOC010000002,JABGOC010000011,JABGOC010000012,JABGOC010000013,JABGOC010000014,JABGOC010000015" 62 6413374 bronchoalveolar lavage United Kingdom "Human, Homo sapiens" Resistant Computational Prediction "fosA,aph(3')-Iib,catB7,blaPAO,blaOXA-50," 95.1 Good 1 1 0 1 1 1 1 0 0 0 0 0

Pseudomonas aeruginosa strain 3473 287 WGS 3473 "JABGNZ010000001,JABGNZ010000002,JABGNZ010000011,JABGNZ010000012,JABGNZ010000013,JABGNZ010000014,JABGNZ010000015" 65 6414001 bronchoalveolar lavage United Kingdom "Human, Homo sapiens" Resistant Computational Prediction "fosA,aph(3')-Iib,catB7,blaPAO,blaOXA-50," 95.1 Good 1 1 0 1 1 1 1 0 0 0 0 0

Pseudomonas aeruginosa strain 3472 287 WGS 3472 "JABGOA010000001,JABGOA010000002,JABGOA010000011,JABGOA010000012,JABGOA010000013,JABGOA010000014,JABGOA010000015" 67 6411878 bronchoalveolar lavage United Kingdom "Human, Homo sapiens" Resistant Computational Prediction "fosA,aph(3')-Iib,catB7,blaPAO,blaOXA-50," 95.1 Good 1 1 0 1 1 1 1 0 0 0 0 0

Pseudomonas aeruginosa strain 4007 287 WGS 4007 "JABGNW010000001,JABGNW010000002,JABGNW010000011,JABGNW010000101,JABGNW010000102,JABGNW010000103,JABGNW010000104" 106 6429547 bronchoalveolar lavage United Kingdom "Human, Homo sapiens" Resistant Computational Prediction "fosA,aph(3')-Iib,catB7,blaPAO,blaOXA-50," 96.2 Good 1 1 0 1 1 1 1 0 0 0 0 0

Pseudomonas aeruginosa strain C2-105-1 287 WGS C2-105-1 SWGJ01000000 339 6955705 Tissue Colombia "Human, Homo sapiens" Resistant Computational Prediction "crpP,aac(6')-Ib-cr,fosA,tet©,aph(3')-Iib,aac(6')-Ib3,aph(3'')-Ib,ant(2'')-Ia,aph(6)-Id,sul1,catB7,blaPAO,blaOXA-488,qacE," 94.2 Good 1 1 0 1 1 1 1 0 1 1 0 1

Pseudomonas aeruginosa strain C2-54 287 WGS C2-54 SWGK01000000 338 6789920 Tissue Colombia "Human, Homo sapiens" Resistant Computational Prediction "crpP,catB7,fosA,blaPAO,aph(3')-Iib,blaOXA-50," 94.4 Good 1 1 0 1 1 1 1 0 0 1 0 1

Pseudomonas aeruginosa strain C2-155-2 287 WGS C2-155-2 SWGG01000000 467 6877665 Rectal swab Colombia "Human, Homo sapiens" Resistant Computational Prediction "fosA,qnrVC1,aph(3')-Via,aac(6')-Il,aph(3')-Iib,ant(2'')-Ia,sul1,dfrA22,ARR-2,catB7,catB3,blaVIM-2,blaPAO,blaOXA-50," 92.2 Good 1 1 1 1 1 1 1 0 0 1 1 1

Pseudomonas aeruginosa strain C2-102-3 287 WGS C2-102-3 SWGI01000000 194 7119993 osteosynthesis material Colombia "Human, Homo sapiens" Resistant Computational Prediction "crpP,aac(6')-Ib-cr,ant(2'')-Ia,aph(3'')-Ib,aph(3')-Iib,aac(6')-Ib3,aph(6)-Id,fosA,sul1,tet©,catB7,blaPAO,blaKPC-2,blaOXA-488,qacE" 95.3 Good 1 1 1 1 1 1 1 0 1 1 0 1

Pseudomonas aeruginosa strain C2-64-3 287 WGS C2-64-3 SWGN01000000 438 7029020 Rectal swab Colombia "Human, Homo sapiens" Resistant Computational Prediction "crpP,aac(6')-Ib-cr,aac(6')-Ib3,aac(6')-29b,aph(3')-Iib,aadA1b,fosA,sul1,catB7,blaPAO,blaOXA-395,blaVIM-2,blaOXA-10,qacE," 93.9 Good 1 1 1 1 1 1 1 0 0 0 0 1

Pseudomonas aeruginosa strain C2-64-2 287 WGS C2-64-2 SWGO01000000 182 7145527 Tissue Colombia "Human, Homo sapiens" Resistant Computational Prediction "crpP,aac(6')-Ib-cr,aac(6')-29b,aadA1b,aac(6')-Ib3,fosA,aph(3')-Iib,sul1,catB7,blaVIM-2,blaPAO,blaOXA-395,blaOXA-10,qacE," 95.6 Good 1 1 1 1 1 1 1 0 0 0 0 0

Pseudomonas aeruginosa strain C2-42-1 287 WGS C2-42-1 SWGP01000000 383 7437298 Secretion Colombia "Human, Homo sapiens" Resistant Computational Prediction "qnrVC1,aac(6')-Ib-cr,crpP,fosA,dfrA22,sul1,aadA1b,aph(3')-Iib,ant(2'')-Ia,aac(6')-Ib3,aac(6')-29b,ARR-2,catB7,catB3,blaOXA-395,blaPAO,blaOXA-10,blaVIM-2,qacE," 94.1 Good 1 1 1 1 1 1 1 0 0 1 0 1

Pseudomonas aeruginosa strain C2-42 287 WGS C2-42 SWGQ01000000 189 7521464 Rectal swab Colombia "Human, Homo sapiens" Resistant Computational Prediction "qnrVC1,aac(6')-Ib-cr,fosA,sul1,dfrA22,aadA1b,aph(3')-Iib,aph(3')-Via,aac(6')-Ib3,ant(2'')-Ia,aac(6')-29a,ARR-2,catB7,catB3,blaPAO,blaOXA-10,blaVIM-2,blaOXA-395," 94.8 Good 1 1 1 1 1 1 1 0 0 1 1 1

Pseudomonas aeruginosa strain C2-30-1 287 WGS C2-30-1 SWGR01000000 168 7100866 bone Colombia "Human, Homo sapiens" Resistant Computational Prediction "crpP,aac(6')-Ib-cr,fosA,aph(3')-Iib,aac(6')-Ib3,aadA1b,aac(6')-29a,sul1,catB7,blaPAO,blaVIM-2,blaOXA-395,blaOXA-10,qacE," 95.7 Good 1 1 1 1 1 1 1 0 0 0 0 1

Pseudomonas aeruginosa strain C1-133-2 287 WGS C1-133-2 SWGS01000000 218 7197362 tracheal aspirate Colombia "Human, Homo sapiens" Resistant Computational Prediction "crpP,aac(6')-Ib-cr,aadA1b,aph(3')-Iib,aac(6')-Ib3,aac(6')-29b,fosA,sul1,catB7,blaVIM-2,blaOXA-395,blaOXA-10,blaPAO,qacE" 95.1 Good 1 1 1 1 1 1 1 0 0 0 0 1

Pseudomonas aeruginosa strain PASP657 287 WGS PASP657 "JABLTU010000100,JABLTU010000101,JABLTU010000102,JABLTU010000103,JABLTU010000104,JABLTU010000105,JABLTU010000106" 134 6836322 blood Spain "Human, Homo sapiens" Resistant Computational Prediction "fosA,aph(3')-Iib,catB7,blaPAO,blaOXA-50,crpP," 96.1 Good 1 1 0 1 1 1 1 0 0 0 0 1

Pseudomonas aeruginosa strain PASP612 287 WGS PASP612 "JABLTW010000100,JABLTW010000101,JABLTW010000102,JABLTW010000103,JABLTW010000104,JABLTW010000105,JABLTW010000106" 209 6962611 blood Spain "Human, Homo sapiens" Resistant Computational Prediction "crpP,ant(2'')-Ia,aph(3')-Iib,fosA,catB7,blaPAO,blaOXA-50,qacE" 95.6 Good 1 1 0 1 1 1 1 0 0 1 0 1

Pseudomonas aeruginosa strain PASP315 287 WGS PASP315 "JABLUJ010000100,JABLUJ010000101,JABLUJ010000102,JABLUJ010000103,JABLUJ010000104,JABLUJ010000105,JABLUJ010000106" 128 6910178 blood Spain "Human, Homo sapiens" Resistant Computational Prediction "crpP,aac(6')-Ib-cr,fosA,aph(3')-Iib,aac(6')-Ib3,sul1,blaOXA-395,blaPAO,qacE" 95.9 Good 1 1 1 1 1 1 0 0 0 0 0 1

Pseudomonas aeruginosa strain PASP499 287 WGS PASP499 "JABLTZ010000100,JABLTZ010000101,JABLTZ010000102,JABLTZ010000103,JABLTZ010000104,JABLTZ010000105,JABLTZ010000106" 174 7121993 blood Spain "Human, Homo sapiens" Resistant Computational Prediction "crpP,fosA,aph(3')-Iib,ant(2'')-Ia,blaOXA-50,catB7,blaPAO,qacE," 95.7 Good 1 1 0 1 1 1 1 0 0 1 0 1

Pseudomonas aeruginosa strain PASP388 287 WGS PASP388 "JABLUH010000100,JABLUH010000101,JABLUH010000102,JABLUH010000103,JABLUH010000104,JABLUH010000105,JABLUH010000106" 160 7012486 blood Spain "Human, Homo sapiens" Resistant Computational Prediction "crpP,fosA,catB7,blaPAO,aph(3')-Iib,blaOXA-396," 95.9 Good 1 1 1 1 1 1 1 0 0 0 0 1

Pseudomonas aeruginosa strain PASP251 287 WGS PASP251 "JABLUM010000010,JABLUM010000011,JABLUM010000012,JABLUM010000013,JABLUM010000014,JABLUM010000015,JABLUM010000016" 85 6875145 blood Spain "Human, Homo sapiens" Resistant Computational Prediction "catB7,aph(3')-Iib,fosA,blaPAO,blaOXA-50," 95.8 Good 1 1 0 1 1 1 1 0 0 0 0 0

Pseudomonas aeruginosa strain PASP198 287 WGS PASP198 "JABLUP010000010,JABLUP010000011,JABLUP010000012,JABLUP010000013,JABLUP010000014,JABLUP010000015,JABLUP010000016" 75 6279554 blood Spain "Human, Homo sapiens" Resistant Computational Prediction "catB7,aph(3')-Iib,fosA,blaPAO,blaOXA-50," 96.5 Good 1 1 0 1 1 1 1 0 0 0 0 0

Pseudomonas aeruginosa AZPAE14811 287 WGS AZPAE14811 JTVX00000000 124 6981966 respiratory tract infection India "Human, Homo sapiens" Resistant AMR Panel "crpP,aadA2,aph(3'')-Ib,aph(6)-Id,aac(6')-Il,aph(3')-VI,aph(3')-Iib,fosA,dfrA1,tet(G),catB7,cmlA1,blaOXA-486,blaPAO,blaVIM-2,blaOXA-4,qacE," 99.1 99.1 0.3 Good 1 1 1 1 1 1 1 0 1 0 0 1

Pseudomonas aeruginosa AZPAE14732 287 WGS AZPAE14732 JTWA00000000 196 6793558 respiratory tract infection United States "Human, Homo sapiens" Resistant AMR Panel "fosA,aadA1b,ant(2'')-Ia,aac(6')-Iib,aadA6,aph(3')-Iib,aac(3)-I,sul1,catB7,blaPAO,blaOXA-415,blaOXA-488,qacE" 98.1 99.4 Good 1 1 0 1 1 1 1 0 0 0 0 0

Pseudomonas aeruginosa AZPAE14731 287 WGS AZPAE14731 JTWB00000000 105 6231966 respiratory tract infection Italy "Human, Homo sapiens" Resistant;Susceptible AMR Panel "fosA,catB7,blaPAO,blaOXA-396,blaOXA-494,aph(3')-Iib" 98.9 99.4 0.3 Good 1 1 1 1 1 1 1 0 0 0 0 0

Pseudomonas aeruginosa AZPAE14730 287 WGS AZPAE14730 JTWC00000000 198 6865760 respiratory tract infection Italy "Human, Homo sapiens" Resistant AMR Panel "crpP,aac(6')-Ib-cr,aph(3')-Iib,aac(6')-Ib3,aadA6,aac(6')-31,aadA2b,aac(6')-Il,fosA,sul1,catB7,blaCARB-2,blaPAO,blaOXA-488,blaOXA-2,qacE" 98.2 100 0.3 Good 1 0 0 1 1 1 1 0 0 1 0 1

Pseudomonas aeruginosa AZPAE14729 287 WGS AZPAE14729 JTWD00000000 202 7015575 urinary tract infection Italy "Human, Homo sapiens" Resistant AMR Panel "crpP,aac(6')-29b,aph(3')-Iib,fosA,sul1,catB7,blaOXA-395,blaVIM-2,blaPAO,qacE" 98 99.7 Good 1 1 1 1 1 1 1 0 0 0 0 1

Pseudomonas aeruginosa AZPAE14728 287 WGS AZPAE14728 JTWE00000000 217 7356424 itra-abdominal tract infection United States "Human, Homo sapiens" Resistant AMR Panel "crpP,aac(6')-Ib-cr,aph(3')-Iib,aac(6')-Ib3,aadA6,aac(6')-31,aadA2b,aac(6')-Il,fosA,sul1,catB7,blaCARB-2,blaPAO,blaOXA-395,blaOXA-2,qacE" 98.6 99.7 Good 1 1 1 1 1 1 1 0 0 0 0 1

Pseudomonas aeruginosa AZPAE14727 287 WGS AZPAE14727 JTWF00000000 248 7362494 itra-abdominal tract infection United States "Human, Homo sapiens" Resistant AMR Panel "crpP,fosA,aac(6')-Ib-cr,aadA2b,aph(3')-Iib,aac(6')-Ib3,sul1,catB7,blaPAO,blaCARB-2,blaOXA-395,qacE," 98.7 99.1 Good 1 1 1 1 1 1 1 0 0 0 0 1

Pseudomonas aeruginosa AZPAE14725 287 WGS AZPAE14725 JTWH00000000 105 6802459 respiratory tract infection United States "Human, Homo sapiens" Susceptible;Intermediate AMR Panel "fosA,catB7,blaPAO,crpP,blaOXA-50,aph(3')-Iib" 98.8 100 Good 1 1 0 1 1 1 1 0 0 0 0 1

Pseudomonas aeruginosa AZPAE14724 287 WGS AZPAE14724 JTWI00000000 171 7091089 itra-abdominal tract infection Italy "Human, Homo sapiens" Resistant AMR Panel "crpP,aph(3')-Iib,aac(6')-29b,fosA,sul1,catB7,blaPAO,blaOXA-395,blaVIM-2,qacE" 98.8 100 Good 1 1 1 1 1 1 1 0 0 0 0 1

Pseudomonas aeruginosa AZPAE14722 287 WGS AZPAE14722 JTWK00000000 126 6781659 itra-abdominal tract infection Italy "Human, Homo sapiens" Resistant AMR Panel "crpP,fosA,ant(2'')-Ia,aph(3')-Iib,catB7,blaOXA-50,blaPAO,qacE" 98.6 99.1 Good 1 1 1 1 1 1 1 0 0 1 0 1

Pseudomonas aeruginosa AZPAE14721 287 WGS AZPAE14721 JTWL00000000 164 6867543 itra-abdominal tract infection Colombia "Human, Homo sapiens" Resistant AMR Panel "aadA6,aac(6')-29a,aph(3')-Iib,aac(6')-29b,aph(6)-Id,aph(3')-Iib,fosA,sul1,tet©,catB7,blaPAO,blaOXA-488,blaKPC-2,qacE" 98.5 100 Good 1 1 1 1 1 1 1 0 1 1 0 0

Pseudomonas aeruginosa AZPAE14720 287 WGS AZPAE14720 JTWM00000000 236 6813733 urinary tract infection Colombia "Human, Homo sapiens" Resistant AMR Panel "aadA6,aac(6')-29a,aph(3')-Iib,aac(6')-29b,aph(6)-Id,fosA,sul1,tet©,catB7,blaPAO,blaOXA-488,blaKPC-2,qacE,blaOXA-2,ant(2'')-Ia,aac(6')-Il," 98.2 99.1 0.3 Good 1 1 1 1 1 1 1 0 1 1 0 0

Pseudomonas aeruginosa AZPAE14719 287 WGS AZPAE14719 JTWN00000000 190 6855346 respiratory tract infection Colombia "Human, Homo sapiens" Resistant AMR Panel "aadA6,aac(6')-29a,aph(3')-Iib,aac(6')-29b,aph(6)-Id,fosA,sul1,tet©,catB7,blaPAO,blaOXA-488,blaKPC-2,qacE,blaOXA-2,ant(2'')-Ia,aac(6')-Il," 98.4 100 Good 1 1 1 1 1 1 1 0 1 1 0 0

Pseudomonas aeruginosa AZPAE14718 287 WGS AZPAE14718 JTWO00000000 231 6910404 respiratory tract infection United States "Human, Homo sapiens" Resistant AMR Panel "fosA,catB7,blaPAO,blaOXA-486,blaOXA-4,aph(3')-Iib,aadA2,aac(3)-Id,aac(6')-Il,dfrB5,tet(G),cmlA1,blaVIM-2,qacE" 97.9 99.1 Good 1 1 1 1 1 1 1 0 1 1 0 1

Pseudomonas aeruginosa AZPAE14717 287 WGS AZPAE14717 JTWP00000000 153 7039359 itra-abdominal tract infection United States "Human, Homo sapiens" Intermediate;Susceptible AMR Panel "crpP,fosA,aph(3')-Iib,catB7,blaOXA-486,blaPAO," 98.4 98.8 0.3 Good 1 1 1 1 1 1 1 0 0 0 0 1

Pseudomonas aeruginosa AZPAE14716 287 WGS AZPAE14716 JTWQ00000000 149 7038646 itra-abdominal tract infection Venezuela "Human, Homo sapiens" Resistant AMR Panel "fosA,catB7,blaPAO,blaOXA-486,blaOXA-4,aph(3')-Iib,aadA2,aac(3)-Id,aac(6')-Il,dfrB5,tet(G),cmlA1,blaVIM-2,qacE" 98.4 99.1 Good 1 1 1 1 1 1 1 0 0 1 1 1

Pseudomonas aeruginosa AZPAE14715 287 WGS AZPAE14715 JTWR00000000 129 7119339 itra-abdominal tract infection Venezuela "Human, Homo sapiens" Resistant AMR Panel "crpP,fosA,aph(3'')-Ib,aph(3')-Ia,aph(6)-Id,aph(3')-Iib,sul1,catB7,blaOXA-396,blaPAO,blaOXA-4,qacE,aadA17,aph(3')-Vib,ant(2'')-Ia,blaPER-1" 98.7 99.7 Good 1 1 1 1 1 1 1 0 0 1 1 1

Pseudomonas aeruginosa AZPAE14714 287 WGS AZPAE14714 JTWS00000000 233 7022276 itra-abdominal tract infection Venezuela "Human, Homo sapiens" Resistant AMR Panel "crpP,aph(3')-Iib,aac(6')-29a,fosA,sul1,catB7,blaPAO,blaVIM-2,blaOXA-395,qacE" 98.2 99.1 0.3 Good 1 1 1 1 1 1 1 0 0 0 0 1

Pseudomonas aeruginosa AZPAE14713 287 WGS AZPAE14713 JTWT00000000 184 7238306 itra-abdominal tract infection Venezuela "Human, Homo sapiens" Resistant AMR Panel "crpP,aph(3')-Iib,aac(6')-29b,fosA,sul1,catB7,blaPAO,blaVIM-2,blaOXA-395,qacE" 98.7 99.7 Good 1 1 1 1 1 1 1 0 0 0 0 1

Pseudomonas aeruginosa AZPAE14712 287 WGS AZPAE14712 JTWU00000000 228 7010520 itra-abdominal tract infection Venezuela "Human, Homo sapiens" Resistant AMR Panel "crpP,aac(6')-Ib-cr,fosA,sul1,tet©,aac(6')-Ib3,ant(2'')-Ia,aph(6)-Id,aph(3')-Iib,aph(3')-Via,catB7,blaPAO,blaOXA-488,blaVIM-2" 98.2 99.7 Good 1 1 1 1 1 1 1 0 1 1 1 0

Pseudomonas aeruginosa AZPAE14711 287 WGS AZPAE14711 JTWV00000000 177 7121620 itra-abdominal tract infection Venezuela "Human, Homo sapiens" Resistant AMR Panel "crpP,aph(3')-Ib,fosA,sul1,aac(6')-Ib3,ant(2'')-Ia,aph(3')-Iib,aph(3')-VIb,catB7,blaPAO,blaOXA-396,blaOXA-4,aadA17,blaPER-1" 98 99.4 Good 1 1 1 1 1 1 1 0 0 1 1 1

Pseudomonas aeruginosa AZPAE14710 287 WGS AZPAE14710 JTWW00000000 147 6748304 respiratory tract infection United States "Human, Homo sapiens" Resistant AMR Panel "aadA6,aph(3')-Iib,aph(3')-Iia,aph(6)-Ic,fosA,sul1,catB7,blaPAO,blaOXA-488,qacE" 98.3 99.7 0.4 Good 1 1 0 1 1 1 1 0 0 1 0 0

Pseudomonas aeruginosa AZPAE14708 287 WGS AZPAE14708 JTWX00000000 187 6707536 itra-abdominal tract infection Greece "Human, Homo sapiens" Resistant AMR Panel "crpP,fosA,aac(6')-Ib-cr,aadA6,aph(3')-Iib,aac(6')-Ib3,sul1,catB7,blaPAO,blaOXA-19,blaOXA-488,qacE," 97.9 99.4 Good 1 1 0 1 1 1 1 0 0 1 0 1

Pseudomonas aeruginosa AZPAE14707 287 WGS AZPAE14707 JTWY00000000 215 7081209 respiratory tract infection Greece "Human, Homo sapiens" Resistant AMR Panel "crpP,aph(3')-Iib,aac(6')-29a,fosA,sul1,catB7,blaPAO,blaVIM-2,blaOXA-395,qacE" 98 99.4 Good 1 1 1 1 1 1 1 0 0 0 0 1

Pseudomonas aeruginosa AZPAE14706 287 WGS AZPAE14706 JTWZ00000000 149 6921381 itra-abdominal tract infection Greece "Human, Homo sapiens" Resistant AMR Panel "crpP,aph(3')-IIb,fosA,sul1,ARR-7,catB7,blaPAO,blaOXA-488,blaCARB-2,blaVIM-4,blaOXA-35,qacE" 98.6 99.7 Good 1 1 1 1 1 1 1 0 0 1 1 1

Pseudomonas aeruginosa AZPAE14705 287 WGS AZPAE14705 JTXA00000000 240 6888268 urinary tract infection Greece "Human, Homo sapiens" Resistant AMR Panel "crpP,fosA,aac(6')-Ib-cr,ant(2'')-Ia,cmlA1,carB3,aadA6,aph(3')-Iib,aac(6')-Ib3,sul1,catB7,blaPAO,blaOXA-10,blaVIM-2,blaOXA-488,qacE," 98.2 99.7 Good 1 1 1 1 1 1 1 0 0 1 0 1

Pseudomonas aeruginosa AZPAE14703 287 WGS AZPAE14703 JTXC00000000 201 6892377 itra-abdominal tract infection Philippines "Human, Homo sapiens" Resistant AMR Panel "crpP,aac(6')-Ib-cr,aad1,ant(2'')-Ia,aac(6')-Ib3,aph(3')-IIb,fosA,sul1,catB3,cmlA1,catB7,blaPAO,blaOXA-488,blaVIM-2,blaOXA-10,qacE" 98.4 99.7 Good 1 1 1 1 1 1 1 0 0 1 0 1

Pseudomonas aeruginosa AZPAE14702 287 WGS AZPAE14702 JTXD00000000 160 7131510 respiratory tract infection Philippines "Human, Homo sapiens" Resistant AMR Panel "crpP,fosA,aac(6')-Ib-cr,dfrA22,sul1,mph€,msr€,aph(3')-IaaadA1,ant(2'')-Ia,aac(6')-Ib3,aph(3')-Iib,ARR-2,catB3,catB7,cmlA1,blaPAO,blaOXA-488,blaIMP-26,blaOXA-10,qacE" 98.9 100 Good 1 1 1 1 1 1 1 1 0 1 0 1

Pseudomonas aeruginosa AZPAE14701 287 WGS AZPAE14701 JTXE00000000 251 6990663 itra-abdominal tract infection Philippines "Human, Homo sapiens" Resistant AMR Panel "crpP,catB7,aph(3')-Iib,blaPAO,blaOXA-494,blaOXA-396,fosA" 99 99.7 Good 1 1 1 1 1 1 1 0 0 0 0 1

Pseudomonas aeruginosa strain DVT639 287 WGS DVT639 "JAATIF010000001,JAATIF010000002" 2 7436835 USA "Human, Homo sapiens" Resistant Computational Prediction "crpP,catB7,aph(3')-Iib,blaPAO,blaOXA-395,fosA" 95.1 Good 1 1 1 1 1 1 1 0 0 0 0 1

Pseudomonas aeruginosa AZPAE14700 287 WGS AZPAE14700 JTXF00000000 156 6754658 respiratory tract infection Philippines "Human, Homo sapiens" Resistant AMR Panel "catB7,crpP" 99.3 99.7 0.3 Good 0 0 0 0 0 0 1 0 0 0 0 1

Pseudomonas aeruginosa strain DVT411 287 WGS DVT411 "JAATIM010000001,JAATIM010000002" 2 6666342 lung USA "Human, Homo sapiens" Resistant Computational Prediction "fosA,blaPAO,aph(3')-Iib,blaOXA-50" 95.6 Good 1 1 0 1 1 1 0 0 0 0 0 0

Pseudomonas aeruginosa strain PSE6684 287 Complete PSE6684 CP053917 1 6924367 urine South Korea "Human, Homo sapiens" Resistant Computational Prediction "crpP,fosA,catB7,blaPAO,qnrVC1,aac(6')-Ib-cr,tet(G),aadA10,aac(6')-Ib-Hangzhou,aph(3')-Iib,rmtb,sul1,ARR-2,blaNDM-1,blaOXA-395,blaOXA-4,blaOXA-1,qacE" 95.9 Good 1 1 1 1 1 1 1 0 1 1 0 1

Pseudomonas aeruginosa strain UNIBA_ST235PA 287 WGS UNIBA_ST235PA "JAABOY010000010,JAABOY010000011,JAABOY010000012,JAABOY010000013,JAABOY010000014,JAABOY010000015,JAABOY010000016" 91 6941885 blood Italy "Human, Homo sapiens" Resistant Computational Prediction "crpP,aph(3')-Iib,blaPAO,aph(3')-VI,blaNDM-1,blaOXA-488" 95.7 Good 1 1 1 1 0 1 0 0 0 1 0 1

Pseudomonas aeruginosa strain V2O4Ci 287 WGS V2O4Ci "JABTCD010000001,JABTCD010000010,JABTCD010000011,JABTCD010000012,JABTCD010000013,JABTCD010000014,JABTCD010000015" 60 6740285 France "Human, Homo sapiens" Resistant Computational Prediction 96.1 Good 1 1 1 1 1 1 1 0 0 1 0 1

Pseudomonas aeruginosa AZPAE14699 287 WGS AZPAE14699 JTXG00000000 501 5743255 itra-abdominal tract infection United States "Human, Homo sapiens" Resistant;Susceptible AMR Panel "crpP,aph(3')-Iib,aac(6')-Il,tet(G)dfrB5,fosA,blaVIM-2,catB7,blaOXA-486,blaPAO," 89.8 82.5 2.8 Good 1 1 1 1 1 1 1 0 1 1 0 0

Pseudomonas aeruginosa strain 185385 287 WGS 185385 "JABTCC010000001,JABTCC010000010,JABTCC010000011,JABTCC010000012,JABTCC010000013,JABTCC010000014,JABTCC010000015" 53 6813774 France "Human, Homo sapiens" Resistant Computational Prediction "crpP,aadA6,aph(3')-Iib,fosA,sul1,catB7,qacE,blaOXA-488," 96 Good 0 0 0 0 1 0 1 0 0 0 0 1

Pseudomonas aeruginosa strain 131709 287 WGS 131709 "JABTBW010000001,JABTBW010000010,JABTBW010000100,JABTBW010000101,JABTBW010000102,JABTBW010000103,JABTBW010000104" 138 7032749 Bones France "Human, Homo sapiens" Resistant Computational Prediction "crpP,aph(3')-Iib,fosA,aac(3)-Id,aph(3'')-Ib,aac(6')-Il,aph(3')-Vib,aph(6)-Id,aac(6')-Il,dfrB5,catB7,blaVIM-2blaPER-1blaPAO" 95.1 Good 1 1 1 1 1 1 1 0 0 1 1 1

Pseudomonas aeruginosa strain 163915 287 WGS 163915 "JABTBZ010000001,JABTBZ010000010,JABTBZ010000011,JABTBZ010000012,JABTBZ010000013,JABTBZ010000014,JABTBZ010000015" 66 6868182 urine France "Human, Homo sapiens" Resistant Computational Prediction "crpP,aadA2,aac(3)-Id,aph(6)-Id,aph(3'')-Ib,aph(3')-Vib,aac(6')-Il,fosA,sul1,dfrB5,cmlA1,tet(G),catB7,blaOXA-4,blaVIM-2,blaPER-1,blaOXA-488,qacE" 94.8 Good 1 1 1 1 1 1 1 0 1 1 1 1

Pseudomonas aeruginosa strain 174581 287 WGS 174581 "JABTCB010000001,JABTCB010000010,JABTCB010000011,JABTCB010000012,JABTCB010000013,JABTCB010000014,JABTCB010000015" 76 6927367 urine France "Human, Homo sapiens" Resistant Computational Prediction "crpP,aph(3')-IIbfosA,aadA2,aac(3)-Id,aac(6')-Il,dfrB5,sul1,catB7,tet(G),cmlA1,blaVIM-2,blaPAO,blaOXA-4,blaVIM-2,blaOXA-488,qacE" 96 Good 1 1 1 1 1 1 1 0 1 1 0 1

Pseudomonas aeruginosa strain 163603 287 WGS 163603 "JABTBY010000001,JABTBY010000010,JABTBY010000011,JABTBY010000012,JABTBY010000013,JABTBY010000014,JABTBY010000015" 79 6987968 urine France "Human, Homo sapiens" Resistant Computational Prediction "crpP,fosA,aac(6')-Il,aac(3)-Id,aadA2,aph(3')-Iib,tet(G),sul1,dfrB5,catB7,cmlA1,blaVIM-2,blaPAO,blaOXA-486,blaOXA-4,qacE" 94.6 Good 1 1 1 1 1 1 1 0 1 1 0 1

Pseudomonas aeruginosa strain 152609 287 WGS 152609 "JABTBX010000001,JABTBX010000010,JABTBX010000100,JABTBX010000101,JABTBX010000102,JABTBX010000103,JABTBX010000104" 117 6993150 Bones France "Human, Homo sapiens" Resistant Computational Prediction "fosA,aph(3')-Iib,aadA2,aac(6')-Il,aac(3)-Id,aac(3)-Id,dfrB5,sul1,tet(G),cmlA1,blaOXA-4,blaVIM-2,blaOXA-486,blaPAO,qacE" 95.7 Good 1 1 1 1 1 1 1 0 1 1 0 0

Pseudomonas aeruginosa strain 174276 287 WGS 174276 "JABTCA010000001,JABTCA010000010,JABTCA010000100,JABTCA010000101,JABTCA010000102,JABTCA010000103,JABTCA010000104" 138 6964328 Rectal swab France "Human, Homo sapiens" Resistant Computational Prediction "crpP,fosA,aph(3')-Iib,aac(3)-Id,aac(6')-Il,aadA2,aac(3)-Id,dfrB5,sul1,tet(G),cmlA1,catB7,blaVIM-2,blaOXA-4,blaOXA-486,blaPAO,qacE" 95.6 Good 1 1 1 1 1 1 1 0 1 1 0 1

Pseudomonas aeruginosa strain 12866 287 WGS 12866 "JABTBV010000001,JABTBV010000010,JABTBV010000011,JABTBV010000012,JABTBV010000013,JABTBV010000014,JABTBV010000015" 87 6892119 France "Human, Homo sapiens" Resistant Computational Prediction "crpP,fosA,aac(6')-Il,aadA2,aph(3')-Iib,aac(3)-Id,aac(3)-Id,dfrB5,sul1,tet(G),cmlA1,catB7,blaOXA-486,blaVIM-2,blaPAO,blaOXA-4,qacE" 95.8 Good 1 1 1 1 1 1 1 0 1 1 0 1

Pseudomonas aeruginosa strain 09107 287 WGS 9107 "JABTBU010000001,JABTBU010000010,JABTBU010000011,JABTBU010000012,JABTBU010000013,JABTBU010000014,JABTBU010000015" 84 6954885 France "Human, Homo sapiens" Resistant Computational Prediction "crpP,fosA,aac(6')-Il,aadA2,aph(3')-Iib,aac(3)-Id,dfrB5,sul1,tet(G),cmlA1,catB7,blaOXA-486,blaVIM-2,blaPAO,blaOXA-4,qacE" 95.8 Good 1 1 1 1 1 1 1 0 1 1 0 1

Pseudomonas aeruginosa strain YT12746 287 Complete YT12746 CP045552 1 6870662 blood China "Human, Homo sapiens" Resistant Computational Prediction "crpP,aac(6')-Ib-cr,aph(3')-Iib,aadA2b,aac(6')-Iia,aac(6')-Ib3,aph(3'')-Ib,ant(2'')-Ia,fosA,sul1,catB3,catB7,blaOXA-396,blaOXA-494,blaPAO,blaPER-1,qacE,blaCARB-2" 95.9 Good 1 1 1 1 1 1 1 0 0 1 0 1

Pseudomonas aeruginosa strain ROUE09 287 WGS ROUE09 "JABUGH010000001,JABUGH010000010,JABUGH010000011,JABUGH010000012,JABUGH010000013,JABUGH010000014,JABUGH010000015" 55 6412266 France "Human, Homo sapiens" Resistant Computational Prediction "crpP,aph(3')-Iib,catB7,blaPAO,fosA,blaOXA-486" 96.1 Good 1 1 0 1 1 1 1 0 0 0 0 1

Pseudomonas aeruginosa strain POIT06 287 WGS POIT06 "JABUGJ010000001,JABUGJ010000010,JABUGJ010000011,JABUGJ010000012,JABUGJ010000013,JABUGJ010000014,JABUGJ010000015" 71 7030359 lumbar puncture France "Human, Homo sapiens" Resistant Computational Prediction "fosA,blaPAO,aph(3')-Iib" 94.8 Good 1 1 0 1 1 1 0 0 0 1 0 0

Pseudomonas aeruginosa AZPAE14697 287 WGS AZPAE14697 JTXI00000000 225 6531914 respiratory tract infection Israel "Human, Homo sapiens" Susceptible;Intermediate AMR Panel "crpP,aph(3')-Iib,fosA,blaOXA-486,blaPAO,catB7," 97.6 99.1 0.3 Good 1 1 0 1 1 1 1 0 0 0 0 1

Pseudomonas aeruginosa strain GAR02 287 WGS GAR02 "JABUGS010000001,JABUGS010000010,JABUGS010000011,JABUGS010000012,JABUGS010000013,JABUGS010000014,JABUGS010000015" 78 6626552 France "Human, Homo sapiens" Resistant Computational Prediction "crpP,aph(3')-Iib,fosA,blaOXA-494,blaOXA-50,catB7,blaOXA-396" 95.3 Good 1 1 1 0 1 0 1 0 0 0 0 1

Pseudomonas aeruginosa strain HEGP08 287 WGS HEGP08 "JABUGR010000001,JABUGR010000010,JABUGR010000011,JABUGR010000012,JABUGR010000013,JABUGR010000014,JABUGR010000015" 66 6758914 urine France "Human, Homo sapiens" Resistant Computational Prediction "crpP,fosA,catB7,aph(3')-Iib,blaPAO,blaOXA-488" 96 Good 1 1 0 1 1 1 1 0 0 0 0 1

Pseudomonas aeruginosa strain LILL04 287 WGS LILL04 "JABUGQ010000001,JABUGQ010000010,JABUGQ010000011,JABUGQ010000012,JABUGQ010000013,JABUGQ010000014,JABUGQ010000015" 58 7004240 Tracheal aspiration France "Human, Homo sapiens" Resistant Computational Prediction "crpP,fosA,catB7,aph(3')-Iib,blaPAO,blaOXA-488" 95.7 Good 1 1 0 1 1 1 1 0 0 0 0 1

Pseudomonas aeruginosa strain MONT08 287 WGS MONT08 "JABUGM010000001,JABUGM010000010,JABUGM010000011,JABUGM010000012,JABUGM010000013,JABUGM010000014,JABUGM010000015" 52 6323627 Bronchial aspiration France "Human, Homo sapiens" Resistant Computational Prediction "crpP,fosA,catB7,aph(3')-Iib,blaPAO,blaOXA-396" 96.6 Good 1 1 1 1 1 1 1 0 0 0 0 0

Pseudomonas aeruginosa strain MONT13 287 WGS MONT13 "JABUGL010000001,JABUGL010000010,JABUGL010000011,JABUGL010000012,JABUGL010000013,JABUGL010000014,JABUGL010000015" 74 7136800 Bronchial aspiration France "Human, Homo sapiens" Resistant Computational Prediction "crpP,aac(6')-Ib-cr,aac(6')-Ib3,aph(6)-Id,aph(3'')-Ib,aph(3')-Iib,aac(3)-I,fosA,sul1,blaPAO,blaTEM-2,blaOXA-488,qacE" 94.5 Good 1 1 0 1 1 1 0 0 0 0 0 1

Pseudomonas aeruginosa strain CAE12 287 WGS CAE12 "JABUGT010000001,JABUGT010000010,JABUGT010000011,JABUGT010000012,JABUGT010000013,JABUGT010000014,JABUGT010000015" 93 6915989 Expectoration France "Human, Homo sapiens" Resistant Computational Prediction "crpP,aac(6')-Ib-cr,aac(6')-Ib3,aph(3')-Iib,aadA2b,fosA,sul1,blaCARB-2,catB7,blaPAO,blaOXA-395,qacE" 95.8 Good 1 1 1 1 1 1 1 0 0 0 0 1

Pseudomonas aeruginosa strain BOR21 287 WGS BOR21 "JABUGU010000001,JABUGU010000010,JABUGU010000011,JABUGU010000012,JABUGU010000013,JABUGU010000014,JABUGU010000015" 45 6264925 Expectoration France "Human, Homo sapiens" Resistant Computational Prediction "catB7,aph(3')-Iib,blaPAO,blaOXA-396,blaOXA-494" 96.2 Good 1 1 1 1 0 1 1 0 0 0 0 0

Pseudomonas aeruginosa strain PAAK088 287 Complete PAAK088 CP054472 1 6390212 lungs Canada "Human, Homo sapiens" Resistant Computational Prediction "catB7,fosA,aph(3')-Iib,blaPAO,aph(3')-Iib" 96.1 Good 1 1 0 1 1 1 1 0 0 0 0 0

Pseudomonas aeruginosa strain PAAK095 287 Complete PAAK095 CP054473 1 6780782 lungs Canada "Human, Homo sapiens" Resistant Computational Prediction "fosA,catB7,blaPAO,blaOXA-396,aph(3')-Iib," 94.9 Good 1 1 1 0 1 1 1 0 0 0 0 0

Pseudomonas aeruginosa strain DVT729 287 Complete DVT729 CP050322 1 6546693 lung USA "Human, Homo sapiens" Resistant Computational Prediction "crpP,fosA,catB7,blaPAO,blaOXA-396,aph(3')-Iib,blaOXA-494" 94.8 Good 1 1 1 1 1 1 1 0 0 0 0 1

Pseudomonas aeruginosa AZPAE14694 287 WGS AZPAE14694 JTXK00000000 268 6964286 urinary tract infection Romania "Human, Homo sapiens" Resistant AMR Panel "crpP,fosA,catB7,blaPAO,blaOXA-396,aph(3')-Iib," 98.7 98.5 Good 1 1 1 1 1 1 1 0 0 0 0 1

Pseudomonas aeruginosa strain DVT425 287 Complete DVT425 CP050325 1 6506945 lung USA "Human, Homo sapiens" Resistant Computational Prediction "crpP,aac(6')-Ib-cr,ant(2'')-Ia,aph(3'')-Ib,aph(3')-Iib,aph(6)-Id,aph(3')-Vib,aph(3')-XV,fosA,sul1,cmlA1,cmx,catB7,blaPAO,blaOXA-2,blaOXA-488,blaPER-1,blaOXA-74,qacE" 95.8 Good 1 1 0 1 1 1 1 0 0 1 1 1

Pseudomonas aeruginosa strain DVT410 287 Complete DVT410 CP050334 1 6229931 lung USA "Human, Homo sapiens" Resistant Computational Prediction "fosA,catB7,blaPAO,blaOXA-395,aph(3')-Iib," 96.5 Good 1 1 1 1 1 1 1 0 0 0 0 0

Pseudomonas aeruginosa AZPAE14693 287 WGS AZPAE14693 JTXL00000000 50 6380431 itra-abdominal tract infection Romania "Human, Homo sapiens" Susceptible;Resistant AMR Panel "fosA,catB7,blaPAO,blaOXA-396,aph(3')-Iib,blaOXA-494,crpP" 98.9 100 Good 1 1 1 1 1 1 1 0 0 0 0 1

Pseudomonas aeruginosa strain CDN118 287 Complete CDN118 CP054591 1 6832395 bacteremia Nigeria "Human, Homo sapiens" Resistant Computational Prediction "crpP,fosA,aadA2,aac(3)-Id,aac(6')-Il,aph(3')-Iib,sul1,dfrB5,tet(G),cmlA1,catB7,blaVIM-2,blaOXA-4,blaPAO,blaOXA-486,qacE" 96 Good 1 1 1 1 1 1 1 0 1 1 0 1

Pseudomonas aeruginosa AZPAE14692 287 WGS AZPAE14692 JTXM00000000 123 7011166 itra-abdominal tract infection United States "Human, Homo sapiens" Resistant AMR Panel "crpP,aadA6,aph(3')-Iib,fosA,sul1,catB7,blaOXA-396,blapAO,qacE" 98.7 100 0.3 Good 1 1 1 1 1 1 1 0 0 1 0 1

Pseudomonas aeruginosa strain PABCH42 287 Complete PABCH42 "CP056090,CP056091" 2 7274920 endotracheal tube (ETT) or tracheostomy tube USA "Human, Homo sapiens" Resistant Computational Prediction "crpP,aph(3')-Iib,fosA,catB7,blaOXA-396,blapAO," 95.7 Good 1 1 1 1 1 1 1 0 0 0 0 1

Pseudomonas aeruginosa AZPAE14691 287 WGS AZPAE14691 JTXN00000000 76 6301926 respiratory tract infection United States "Human, Homo sapiens" Intermediate;Susceptible AMR Panel "aph(3')-Iib,fosA,catB7,blaOXA-395,blapAO," 99.3 100 Good 1 1 1 1 1 1 1 0 0 0 0 0

Pseudomonas aeruginosa AZPAE14690 287 WGS AZPAE14690 JTXO00000000 106 6730564 urinary tract infection Romania "Human, Homo sapiens" Resistant AMR Panel "fosA,aac(6')-Ib-cr,aph(6)-Id,aph(3'')-Ib,aph(3')-Iib,aph(3')-IIbaadA6,sul1,aac(6')-Ib3,cmlA1,catB7,blaOXA-488,blaPAO,blaOXA-14,qacE" 98.9 100 0.3 Good 1 1 0 1 1 1 1 0 0 1 0 1

Pseudomonas aeruginosa AZPAE14689 287 WGS AZPAE14689 JTXP00000000 216 7252889 respiratory tract infection Mexico "Human, Homo sapiens" Resistant AMR Panel "crpP,aac(6')-Ib-cr,aph(3')-Iib,aac(6')-Ib-Hangzhou,aac(6')-Il,aph(3')-Via,aadA1b,aadA6,fosA,catB7,blaPAO,blaGES-1,blaOXA-2,blaOXA-488,qacE" 98.6 98.8 0.3 Good 1 1 0 1 1 1 1 0 0 1 1 1

Pseudomonas aeruginosa AZPAE14688 287 WGS AZPAE14688 JTXQ00000000 149 7007711 Mexico "Human, Homo sapiens" Resistant;Susceptible AMR Panel "fosA,aac(6')-Ib-cr,aph(6)-Ic,aph(3')-Iib,aph(3')-Iia,aac(6')-29b,aac(6')-Ib-Hangzhou,sul1,catB7,blaPAO,blaOXA-396,blaIMP-56,qacE" 98.2 100 0.6 Good 1 1 1 1 1 1 1 0 0 0 0 1

Pseudomonas aeruginosa AZPAE14687 287 WGS AZPAE14687 JTXR00000000 147 6995963 respiratory tract infection Mexico "Human, Homo sapiens" Resistant AMR Panel "aadA1b,aac(6')-Ib-cr,aac(6')-Ib-Hangzhou,aph(3')-Iib,aph(3')-Via,aac(6')-Il,aadA6,fosA,sul1,catB7,catA1,blaPAO,blaOXA-2,blaOXA-494,blaIMP-62,blaOXA-396,qacE" 99.1 100 1.2 Good 1 1 1 1 1 1 1 0 0 1 1 1

Pseudomonas aeruginosa AZPAE14557 287 WGS AZPAE14557 JTXU00000000 82 6772788 respiratory tract infection Germany "Human, Homo sapiens" Resistant;Susceptible AMR Panel "crpP,aph(3')-Iib,catB7,blaOXA-486,blaPAO,fosA" 99 100 Good 1 1 0 1 1 1 1 0 0 0 0 1

Pseudomonas aeruginosa AZPAE14554 287 WGS AZPAE14554 JTXV00000000 119 7101679 respiratory tract infection Spain "Human, Homo sapiens" Resistant AMR Panel "crpP,aac(6')-Ib-cr,aac(6')-Ib3,aph(3')-Iib,fosA,sul1,catB7,blaOXA-395,blaOXA-46,blaPAO,qacE," 99.1 100 Good 1 1 1 1 1 1 1 0 0 0 0 0

Pseudomonas aeruginosa AZPAE14538 287 WGS AZPAE14538 JTXX00000000 78 6267671 respiratory tract infection China "Human, Homo sapiens" Resistant;Susceptible AMR Panel "catB7,aph(3')-Iib,blaPAO,blaOXA-486,fosA" 99.6 100 Good 1 1 0 1 1 1 1 0 0 0 0 0

Pseudomonas aeruginosa AZPAE14535 287 WGS AZPAE14535 JTXY00000000 105 6944986 respiratory tract infection Spain "Human, Homo sapiens" Resistant AMR Panel "catB7,aph(3')-Iib,blaPAO,blaOXA-395,fosA,crpP," 99 100 Good 1 1 1 1 1 1 1 0 0 0 0 1

Pseudomonas aeruginosa strain CDN129 287 Complete CDN129 CP056774 1 6770626 wound Nigeria "Human, Homo sapiens" Resistant Computational Prediction "crpP,fosA,v,aph(3')-Iib,aac(3)-Id,aadA2,dfrB5,sul1,tet(G),cmlA1,catB7,blaPAO,blaOXA-4,blaOXA486,blaVIM-2,qacE" 96 Good 1 1 1 1 1 1 1 0 1 1 0 1

Pseudomonas aeruginosa strain SE5331 287 Complete SE5331 CP046402 1 7056430 respiratory China "Human, Homo sapiens" Resistant Computational Prediction "crpP,aac(6')-Ib-cr,fosA,tet(G),aadA6,aph(3')-Iib,sul1,aph(3')-XV,aac(6')-Ib3,catB7,blaOXA-488,blaGES-13,blaGES-7,blaGES-5,blaGES-6,blaPAO,qacE" 95.9 Good 1 1 0 1 1 1 1 0 1 1 0 1

Pseudomonas aeruginosa strain SE5369 287 Complete SE5369 CP046403 1 7153217 Shunt fluid China "Human, Homo sapiens" Resistant Computational Prediction "catB7,aph(3')-Iib,blaPAO,blaOXA-396,fosA,crpP," 95.7 Good 1 1 1 1 1 1 1 0 0 0 0 1

Pseudomonas aeruginosa strain SE5416 287 Complete SE5416 CP046404 1 6874270 sputum China "Human, Homo sapiens" Resistant Computational Prediction "crpP,fosA,aadA1,aph(3')-Iib,sul1,tet(G)cmlA1,catB7,blaOXA-494,blaOXA-396,blaPAO,qacE" 95.8 Good 1 1 1 1 1 1 1 0 1 0 0 1

Pseudomonas aeruginosa strain SE5443 287 Complete SE5443 CP046405 1 6579572 respiratory China "Human, Homo sapiens" Resistant Computational Prediction "crpP,fosA,v,aph(3')-Iib,ant(2'')-Ia,aac(6')-Iia,sul1,catB7,blaVIM-2,blaPAO,blaOXA-486,blaCARB-2,qacE" 96.5 Good 1 1 1 1 1 1 1 0 0 1 0 1

Pseudomonas aeruginosa strain SE5458 287 Complete SE5458 CP046406 1 7157752 respiratory China "Human, Homo sapiens" Resistant Computational Prediction "crpP,fosA,v,rmtB,aac(3)-Iid,aadA1,aadA2,aph(3')-VI,aph(3')-Iib,ant(2'')-Ia,sul1,dfrA12,cmlA1,catB7,blaTEM-1B,blaVEB-3,blaOXA-488,blaOXA-10,blaPAO,qacE" 95.6 Good 1 1 0 1 1 1 1 0 0 1 0 1

Pseudomonas aeruginosa strain Pae1252-NDM1 287 WGS Pae1252-NDM1 "JABJWI010000098,JABJWI010000099,JABJWI010000100,JABJWI010000101,JABJWI010000102,JABJWI010000103,JABJWI010000104" 215 6908359 Tracheobronchial aspirates Bulgaria "Human, Homo sapiens" Resistant Computational Prediction "fosA,ant(2'')-Ia,aph(3')-Iib,aph(6)-Id,aph(3'')-Ib,aph(3')-VI,tet(A),blaGES-5,blaNDM-1,aadA6,sul1,catB7,blaPAO,blaOXA-396," 94.5 Good 1 1 1 1 1 1 1 0 1 1 0 0

Pseudomonas aeruginosa strain Pae1257-NDM1 287 WGS Pae1257-NDM1 "JABJWJ010000100,JABJWJ010000101,JABJWJ010000102,JABJWJ010000103,JABJWJ010000104,JABJWJ010000105,JABJWJ010000106" 217 6903901 Tracheobronchial aspirates Bulgaria "Human, Homo sapiens" Resistant Computational Prediction "fosA,aph(3')-Via,ant(2'')-Ia,aph(6)-Id,aph(3'')-Ib,aph(3')-Iib,sul1,tet(A),catB7,blaNDM-1,blaOXA-396,blaGES-5,blaPAO" 94.8 Good 1 1 1 1 1 1 1 0 1 0 1 0

Pseudomonas aeruginosa strain A17CT 287 Complete A17CT CP053119 1 6264758 respiratory tract Australia "Human, Homo sapiens" Resistant Computational Prediction "fosA,catB7,aph(3')-Iib,blaPAO,blaOXA-486" 96.5 Good 1 1 0 1 1 1 1 0 0 0 0 0

Pseudomonas aeruginosa strain NMI1897/13 287 WGS NMI1897/13 "JACCIO010000095,JACCIO010000096,JACCIO010000097,JACCIO010000098,JACCIO010000099,JACCIO010000100,JACCIO010000101" 138 7105814 bronchoalveolar lavage Poland "Human, Homo sapiens" Resistant Computational Prediction "crpP,aac(6')-Ib-cr,ant(4')-Iib,aadA2b,ant(2'')-Ia,aadA10,aac(6')-Ib3,aph(3')-Iib,fosA,sul1,blaPAO,blaCARB-2,blaVIM-2,blaOXA-395,blaOXA-2,qacE" 95.6 Good 1 1 1 1 1 1 0 0 0 1 0 1

Pseudomonas aeruginosa AZPAE14505 287 WGS AZPAE14505 JTYC00000000 121 6823044 respiratory tract infection France "Human, Homo sapiens" Resistant;Susceptible AMR Panel "crpP,fosA,v,ant(2'')-Ia,sul1,catB7,blaVIM-2,blaOXA-50,blaPAO,qacE" 99.3 99.7 0.6 Good 1 1 1 1 1 1 1 0 0 1 0 1

Pseudomonas aeruginosa strain NMI5352/14 287 WGS NMI5352/14 "JACCIK010000099,JACCIK010000100,JACCIK010000101,JACCIK010000102,JACCIK010000103,JACCIK010000104,JACCIK010000105" 120 7379969 blood Poland "Human, Homo sapiens" Resistant Computational Prediction "crpP,fosA,v,ant(2'')-Ia,aadA10,ant(4')-Iib,sul1,catB7,blaOXA-50,blaVIM-2,blaPAO,qacE" 95.5 Good 1 1 1 1 1 1 1 0 0 1 0 1

Pseudomonas aeruginosa strain NMI18/09 287 WGS NMI18/09 "JACCIT010000010,JACCIT010000011,JACCIT010000012,JACCIT010000013,JACCIT010000014,JACCIT010000015,JACCIT010000016" 80 6960089 blood Poland "Human, Homo sapiens" Resistant Computational Prediction "crpP,fosA,aadA10,aph(3')-Iib,ant(4')-Iib,ant(2'')-Ia,sul1,catB7,blaPAO,blaOXA-486,blaVIM-2,qacE" 95.5 Good 1 1 1 1 1 1 1 0 0 1 0 1

Pseudomonas aeruginosa strain NMI3231/13 287 WGS NMI3231/13 "JACCIN010000064,JACCIN010000010,JACCIN010000011,JACCIN010000065,JACCIN010000012,JACCIN010000066,JACCIN010000013" 124 6972381 wound Poland "Human, Homo sapiens" Resistant Computational Prediction "crpP,fosA,aadA10,aph(3')-Iib,ant(2'')-Ia,sul1,blaPAO,blaOXA-395,blaVIM-2,qacE" 95.8 Good 1 1 1 1 1 1 0 0 0 1 0 1

Pseudomonas aeruginosa strain NMI5912/09 287 WGS NMI5912/09 "JACCIS010000091,JACCIS010000092,JACCIS010000093,JACCIS010000094,JACCIS010000095,JACCIS010000096,JACCIS010000097" 125 6857690 wound Poland "Human, Homo sapiens" Resistant Computational Prediction "crpP,fosA,aadA10,aph(3')-Iib,ant(2'')-Ia,sul1,blaPAO,blaOXA-486,blaVIM-2,qacE,catB7," 95.7 Good 1 1 1 1 1 1 1 0 0 1 0 0

Pseudomonas aeruginosa strain NNPS180 287 WGS NNPS180 "JACCIH010000001,JACCIH010000010,JACCIH010000099,JACCIH010000100,JACCIH010000101,JACCIH010000102,JACCIH010000103" 140 6602334 soft tissue inflammation site Russia "Human, Homo sapiens" Resistant Computational Prediction "fosA,aac(3)-Ic,aph(3')-Iib,aac(3)-Id,aac(6')-Il,dfrB5,sul1,cmlA1,catB7,blaPao,blaOXA-488,qacE" 96 Good 1 1 0 1 1 1 1 0 0 1 1 0

Pseudomonas aeruginosa strain NNPS269 287 WGS NNPS269 "JACCIJ010000001,JACCIJ010000010,JACCIJ010000100,JACCIJ010000101,JACCIJ010000102,JACCIJ010000103,JACCIJ010000104" 190 6866147 soft tissue inflammation site Russia "Human, Homo sapiens" Resistant Computational Prediction "crpP,aac(6')-Ib-cr,aph(3')-Iib,aac(6')-Ib3,aadA6,fosA,sul1,cmlA1,catB7,blaPAO,blaOXA-488,blaOXA-14,qacE" 95.5 Good 1 1 0 1 1 1 1 0 0 1 0 1

Pseudomonas aeruginosa strain NNPS244 287 WGS NNPS244 "JACCII010000001,JACCII010000010,JACCII010000099,JACCII010000100,JACCII010000101,JACCII010000102,JACCII010000103" 141 6909183 soft tissue inflammation site Russia "Human, Homo sapiens" Resistant Computational Prediction "fosA,aph(6)-Id,aph(3'')-Ib,aph(3')-Iib,aph(3')-VI,sul1,tet(A),tet(G),catB7,blaPAO,blaOXA-396,blaVIM-2" 96 Good 1 1 1 1 1 1 1 0 1 0 0 0

Pseudomonas aeruginosa AZPAE14463 287 WGS AZPAE14463 JTYE00000000 125 7034157 urinary tract infection Colombia "Human, Homo sapiens" Resistant AMR Panel "crpP,fosA,aph(3')-Iib,aac(6')-29a,sul1,catB7,blaPAO,blaVIM-2,blaOXA-395,qacE" 99.2 100 Good 1 1 1 1 1 1 1 0 0 0 0 1

Pseudomonas aeruginosa AZPAE14453 287 WGS AZPAE14453 JTYF00000000 88 7052856 urinary tract infection United States "Human, Homo sapiens" Resistant;Susceptible AMR Panel "crpP,fosA,aph(3')-Iib,blaPAO,blaOXA-395," 99.1 100 0.3 Good 1 1 1 1 1 1 0 0 0 0 0 1

Pseudomonas aeruginosa AZPAE14443 287 WGS AZPAE14443 JTYG00000000 47 6268752 urinary tract infection United States "Human, Homo sapiens" Susceptible;Resistant AMR Panel "catB7,fosA,aph(3')-Iib,blaPAO,blaOXA-488," 99.5 100 Good 1 1 0 1 1 1 1 0 0 0 0 0

Pseudomonas aeruginosa AZPAE14442 287 WGS AZPAE14442 JTYH00000000 114 7007955 urinary tract infection Taiwan "Human, Homo sapiens" Resistant;Susceptible AMR Panel "sul1,fosA,catB2,catB3,catB7,blaOXA-395,blaOXA-10,blaPAO,aph(3')-Via,aac(6')-Iia,aph(3')-Iib" 98.4 100 Good 1 1 1 1 1 1 1 0 0 1 1 0

Pseudomonas aeruginosa AZPAE14441 287 WGS AZPAE14441 JTYI00000000 103 6810460 urinary tract infection Taiwan "Human, Homo sapiens" Resistant;Susceptible AMR Panel "fosA,aac(6')-Ib-cr,aac(6')-Ib3,aadA2b,aph(3')-Iib,tet(G),catB7,blaCARB-2,blaPAO,blaOXA-494,blaOXA-396,qacE" 99.4 100 0.6 Good 1 1 1 1 1 1 1 0 1 0 0 1

Pseudomonas aeruginosa AZPAE14422 287 WGS AZPAE14422 JTYK00000000 90 6732223 urinary tract infection United States "Human, Homo sapiens" Resistant AMR Panel "aadA6,aph(3')-Iib,fosA,sul1,tet(G),catB7,blaOXA-488,blaPAO,qacE" 98.8 100 Good 1 1 0 1 1 1 1 0 1 1 0 0

Pseudomonas aeruginosa AZPAE14403 287 WGS AZPAE14403 JTYO00000000 92 6907387 itra-abdominal tract infection France "Human, Homo sapiens" Resistant AMR Panel "crpP,fosA,v,aph(3')-Iib,aadA13,catB7,blaPAO,blaOXA-50,qacE" 99 99.7 Good 1 1 0 1 1 1 1 0 0 1 0 0

Pseudomonas aeruginosa strain PHELES4 287 WGS PHELES4 "JACEMH010000001,JACEMH010000010,JACEMH010000100,JACEMH010000101,JACEMH010000102,JACEMH010000103,JACEMH010000104" 106 6468863 lung United Kingdom "Human, Homo sapiens" Resistant Computational Prediction "catB7,blaPAO,blaOXA-50,fosA,aph(3')-Iib" 96.1 Good 1 1 0 1 1 1 1 0 0 0 0 0

Pseudomonas aeruginosa strain PHELES30 287 WGS PHELES30 "JACEMG010000001,JACEMG010000010,JACEMG010000011,JACEMG010000012,JACEMG010000013,JACEMG010000014,JACEMG010000015" 87 6453945 United Kingdom "Human, Homo sapiens" Resistant Computational Prediction "catB7,blaPAO,blaOXA-50,fosA,aph(3')-Iib" 96.1 Good 1 1 0 1 1 1 1 0 0 0 0 0

Pseudomonas aeruginosa strain PHELES3 287 WGS PHELES3 "JACEMF010000001,JACEMF010000010,JACEMF010000011,JACEMF010000012,JACEMF010000013,JACEMF010000014,JACEMF010000015" 91 6484464 lung United Kingdom "Human, Homo sapiens" Resistant Computational Prediction "catB7,blaPAO,blaOXA-50,fosA,aph(3')-Iib" 95.4 Good 1 1 0 1 1 1 1 0 0 0 0 0

Pseudomonas aeruginosa strain PHELES18 287 WGS PHELES18 "JACELS010000001,JACELS010000010,JACELS010000011,JACELS010000012,JACELS010000013,JACELS010000014,JACELS010000015" 95 6426844 lung United Kingdom "Human, Homo sapiens" Resistant Computational Prediction "catB7,blaPAO,blaOXA-50,fosA,aph(3')-Iib" 96.1 Good 1 1 0 1 1 1 1 0 0 0 0 0

Pseudomonas aeruginosa strain PHELES17 287 WGS PHELES17 "JACELR010000001,JACELR010000010,JACELR010000100,JACELR010000101,JACELR010000102,JACELR010000103,JACELR010000104" 104 6493201 lung United Kingdom "Human, Homo sapiens" Resistant Computational Prediction "catB7,blaPAO,blaOXA-50,fosA,aph(3')-Iib" 96.3 Good 1 1 0 1 1 1 1 0 0 0 0 0

Pseudomonas aeruginosa strain PHELES12 287 WGS PHELES12 "JACELM010000001,JACELM010000010,JACELM010000011,JACELM010000012,JACELM010000013,JACELM010000014,JACELM010000015" 86 6258322 lung United Kingdom "Human, Homo sapiens" Resistant Computational Prediction "catB7,blaPAO,blaOXA-50,fosA,aph(3')-Iib" 95.6 Good 1 1 0 1 1 1 1 0 0 0 0 0

Pseudomonas aeruginosa strain PHELES11 287 WGS PHELES11 "JACELL010000001,JACELL010000010,JACELL010000011,JACELL010000012,JACELL010000013,JACELL010000014,JACELL010000015" 91 6462491 lung United Kingdom "Human, Homo sapiens" Resistant Computational Prediction "catB7,blaPAO,blaOXA-50,fosA,aph(3')-Iib" 96.1 Good 1 1 0 1 1 1 1 0 0 0 0 0

Pseudomonas aeruginosa strain OSIRIS 287 WGS OSIRIS "JACELI010000001,JACELI010000010,JACELI010000011,JACELI010000012,JACELI010000013,JACELI010000014,JACELI010000015" 99 6557580 Upper Resp Tract United Kingdom "Cat, Felis catus" Resistant Computational Prediction "catB7,blaPAO,blaOXA-50,fosA,aph(3')-Iib" 95.9 Good 1 1 0 1 1 1 1 0 0 0 0 0

Pseudomonas aeruginosa strain MdLES 287 WGS MdLES "JACELG010000001,JACELG010000009,JACELG010000099,JACELG010000100,JACELG010000101,JACELG010000102,JACELG010000103" 129 6307015 lung United Kingdom "Human, Homo sapiens" Resistant Computational Prediction "catB7,blaPAO,blaOXA-50,fosA,aph(3')-Iib" 95.8 Good 1 1 0 1 1 1 1 0 0 0 0 0

Pseudomonas aeruginosa strain o59100 287 WGS o59100 "JACELH010000001,JACELH010000010,JACELH010000011,JACELH010000012,JACELH010000013,JACELH010000014,JACELH010000015" 93 6479972 lung United Kingdom "Human, Homo sapiens" Resistant Computational Prediction "catB7,blaPAO,blaOXA-50,fosA,aph(3')-Iib" 95.9 Good 1 1 0 1 1 1 1 0 0 0 0 0

Pseudomonas aeruginosa AZPAE14394 287 WGS AZPAE14394 JTYS00000000 131 6977785 itra-abdominal tract infection Spain "Human, Homo sapiens" Resistant AMR Panel "catB7,blaPAO,blaOXA-50,fosA,aph(3')-Iib" 98.7 99.7 Good 1 1 0 1 1 1 1 0 0 0 0 0

Pseudomonas aeruginosa strain LESB582 287 WGS LESB582 "JACEKO010000001,JACEKO010000010,JACEKO010000097,JACEKO010000098,JACEKO010000099,JACEKO010000100,JACEKO010000101" 108 6535614 lung United Kingdom "Human, Homo sapiens" Resistant Computational Prediction "catB7,blaPAO,blaOXA-50,fosA,aph(3')-Iib" 96.3 Good 1 1 0 1 1 1 1 0 0 0 0 0

Pseudomonas aeruginosa strain CF60P44 287 WGS CF60P44 "JACEKN010000001,JACEKN010000010,JACEKN010000100,JACEKN010000101,JACEKN010000102,JACEKN010000103,JACEKN010000104" 126 6498215 lung United Kingdom "Human, Homo sapiens" Resistant Computational Prediction "catB7,blaPAO,blaOXA-50,fosA,aph(3')-Iib" 95.9 Good 1 1 0 1 1 1 1 0 0 0 0 0

Pseudomonas aeruginosa strain 417GLY 287 WGS 417GLY "JACEKI010000001,JACEKI010000010,JACEKI010000011,JACEKI010000012,JACEKI010000013,JACEKI010000014,JACEKI010000015" 86 6455045 lung United Kingdom "Human, Homo sapiens" Resistant Computational Prediction "catB7,blaPAO,blaOXA-50,fosA,aph(3')-Iib" 96.2 Good 1 1 0 1 1 1 1 0 0 0 0 0

Pseudomonas aeruginosa strain B9 287 WGS B9 "JACEKL010000001,JACEKL010000010,JACEKL010000011,JACEKL010000012,JACEKL010000013,JACEKL010000014,JACEKL010000015" 99 6534692 lung United Kingdom "Human, Homo sapiens" Resistant Computational Prediction "catB7,blaPAO,blaOXA-50,fosA,aph(3')-Iib" 96.2 Good 1 1 0 1 1 1 1 0 0 0 0 0

Pseudomonas aeruginosa strain 120MO 287 WGS 120MO "JACEKF010000001,JACEKF010000010,JACEKF010000011,JACEKF010000012,JACEKF010000013,JACEKF010000014,JACEKF010000015" 99 6495615 lung United Kingdom "Human, Homo sapiens" Resistant Computational Prediction "catB7,blaPAO,blaOXA-50,fosA,aph(3')-Iib" 96.2 Good 1 1 0 1 1 1 1 0 0 0 0 0

Pseudomonas aeruginosa strain 89577 287 WGS 89577 "JACEKB010000001,JACEKB010000010,JACEKB010000100,JACEKB010000101,JACEKB010000102,JACEKB010000103,JACEKB010000104" 140 6586528 lung United Kingdom "Human, Homo sapiens" Resistant Computational Prediction "catB7,blaPAO,blaOXA-50,fosA,aph(3')-Iib" 95.5 Good 1 1 0 1 1 1 1 0 0 0 0 0

Pseudomonas aeruginosa strain 89568 287 WGS 89568 "JACEJZ010000001,JACEJZ010000010,JACEJZ010000100,JACEJZ010000101,JACEJZ010000102,JACEJZ010000011,JACEJZ010000012" 102 6588936 lung United Kingdom "Human, Homo sapiens" Resistant Computational Prediction "catB7,blaPAO,blaOXA-50,fosA,aph(3')-Iib" 96 Good 1 1 0 1 1 1 1 0 0 0 0 0

Pseudomonas aeruginosa strain 89347 287 WGS 89347 "JACEJY010000001,JACEJY010000010,JACEJY010000011,JACEJY010000012,JACEJY010000013,JACEJY010000014,JACEJY010000015" 98 6492275 lung United Kingdom "Human, Homo sapiens" Resistant Computational Prediction "catB7,blaPAO,blaOXA-50,fosA,aph(3')-Iib" 96 Good 1 1 0 1 1 1 1 0 0 0 0 0

Pseudomonas aeruginosa strain 89244 287 WGS 89244 "JACEJX010000001,JACEJX010000010,JACEJX010000011,JACEJX010000012,JACEJX010000013,JACEJX010000014,JACEJX010000015" 95 6493130 lung United Kingdom "Human, Homo sapiens" Resistant Computational Prediction "catB7,blaPAO,blaOXA-50,fosA,aph(3')-Iib" 96.2 Good 1 1 0 1 1 1 1 0 0 0 0 0

Pseudomonas aeruginosa strain 89221 287 WGS 89221 "JACEJV010000001,JACEJV010000010,JACEJV010000100,JACEJV010000101,JACEJV010000102,JACEJV010000103,JACEJV010000104" 110 6492022 lung United Kingdom "Human, Homo sapiens" Resistant Computational Prediction "catB7,blaPAO,blaOXA-50,fosA,aph(3')-Iib" 95.9 Good 1 1 0 1 1 1 1 0 0 0 0 0

Pseudomonas aeruginosa strain 89224 287 WGS 89224 "JACEJW010000001,JACEJW010000010,JACEJW010000011,JACEJW010000012,JACEJW010000013,JACEJW010000014,JACEJW010000015" 99 6490739 lung United Kingdom "Human, Homo sapiens" Resistant Computational Prediction "catB7,blaPAO,blaOXA-50,fosA,aph(3')-Iib" 95.9 Good 1 1 0 1 1 1 1 0 0 0 0 0

Pseudomonas aeruginosa strain 89192 287 WGS 89192 "JACEJS010000001,JACEJS010000010,JACEJS010000011,JACEJS010000012,JACEJS010000013,JACEJS010000014,JACEJS010000015" 98 6493022 lung United Kingdom "Human, Homo sapiens" Resistant Computational Prediction "catB7,blaPAO,blaOXA-50,fosA,aph(3')-Iib" 96.2 Good 1 1 0 1 1 1 1 0 0 0 0 0

Pseudomonas aeruginosa strain 89153 287 WGS 89153 "JACEJR010000001,JACEJR010000010,JACEJR010000100,JACEJR010000011,JACEJR010000012,JACEJR010000013,JACEJR010000014" 100 6492594 lung United Kingdom "Human, Homo sapiens" Resistant Computational Prediction "catB7,blaPAO,blaOXA-50,fosA,aph(3')-Iib" 96.2 Good 1 1 0 1 1 1 1 0 0 0 0 0

Pseudomonas aeruginosa strain 49070 287 WGS 49070 "JACEJH010000001,JACEJH010000010,JACEJH010000100,JACEJH010000101,JACEJH010000102,JACEJH010000103,JACEJH010000104" 109 6596014 lung United Kingdom "Human, Homo sapiens" Resistant Computational Prediction "catB7,blaPAO,blaOXA-50,fosA,aph(3')-Iib" 95.8 Good 1 1 0 1 1 1 1 0 0 0 0 0

Pseudomonas aeruginosa AZPAE14379 287 WGS AZPAE14379 JTYW00000000 44 6253630 itra-abdominal tract infection Germany "Human, Homo sapiens" Resistant;Intermediate;Susceptible AMR Panel "crpP,catB7,blaPAO,blaOXA-50,fosA,aph(3')-Iib" 99.5 100 Good 1 1 0 1 1 1 1 0 0 0 0 1

Pseudomonas aeruginosa strain 9857 287 WGS 9857 "JACEJC010000001,JACEJC010000010,JACEJC010000011,JACEJC010000012,JACEJC010000013,JACEJC010000014,JACEJC010000015" 99 6468458 lung United Kingdom "Human, Homo sapiens" Resistant Computational Prediction "catB7,blaPAO,blaOXA-50,fosA,aph(3')-Iib" 96 Good 1 1 0 1 1 1 1 0 0 0 0 0

Pseudomonas aeruginosa strain 8835 287 WGS 8835 "JACEJA010000001,JACEJA010000010,JACEJA010000011,JACEJA010000012,JACEJA010000013,JACEJA010000014,JACEJA010000015" 98 6436353 lung United Kingdom "Human, Homo sapiens" Resistant Computational Prediction "catB7,blaPAO,blaOXA-50,fosA,aph(3')-Iib" 95.9 Good 1 1 0 1 1 1 1 0 0 0 0 0

Pseudomonas aeruginosa strain PHELES8 287 WGS PHELES8 "JACEML010000001,JACEML010000010,JACEML010000011,JACEML010000012,JACEML010000013,JACEML010000014,JACEML010000015" 88 6488572 lung United Kingdom "Human, Homo sapiens" Resistant Computational Prediction "catB7,blaPAO,blaOXA-50,fosA,aph(3')-Iib" 96.2 Good 1 1 0 1 1 1 1 0 0 0 0 0

Pseudomonas aeruginosa strain PHELES7 287 WGS PHELES7 "JACEMK010000001,JACEMK010000010,JACEMK010000011,JACEMK010000012,JACEMK010000013,JACEMK010000014,JACEMK010000015" 99 6305427 lung United Kingdom "Human, Homo sapiens" Resistant Computational Prediction "catB7,blaPAO,blaOXA-50,fosA,aph(3')-Iib" 95.8 Good 1 1 0 1 1 1 1 0 0 0 0 0

Pseudomonas aeruginosa AZPAE14373 287 WGS AZPAE14373 JTYX00000000 51 6337010 itra-abdominal tract infection Germany "Human, Homo sapiens" Resistant;Susceptible AMR Panel "catB7,blaPAO,blaOXA-50,fosA,aph(3')-Iib" 99.2 99.7 0.3 Good 1 1 0 1 1 1 1 0 0 0 0 0

Pseudomonas aeruginosa strain PHELES6 287 WGS PHELES6 "JACEMJ010000001,JACEMJ010000010,JACEMJ010000100,JACEMJ010000101,JACEMJ010000102,JACEMJ010000011,JACEMJ010000012" 102 6494575 lung United Kingdom "Human, Homo sapiens" Resistant Computational Prediction "catB7,aph(3')-Iib,blaPAO,blaOXA-395,fosA," 96.1 Good 1 1 1 1 1 1 1 0 0 1 0 0

Pseudomonas aeruginosa strain PHELES5 287 WGS PHELES5 "JACEMI010000001,JACEMI010000010,JACEMI010000100,JACEMI010000101,JACEMI010000102,JACEMI010000103,JACEMI010000104" 105 6450171 lung United Kingdom "Human, Homo sapiens" Resistant Computational Prediction "catB7,blaPAO,blaOXA-50,fosA,aph(3')-Iib" 96.3 Good 1 1 0 1 1 1 1 0 0 0 0 0

Pseudomonas aeruginosa strain PHELES28 287 WGS PHELES28 "JACEMD010000001,JACEMD010000010,JACEMD010000100,JACEMD010000101,JACEMD010000011,JACEMD010000012,JACEMD010000013" 101 6493384 lung United Kingdom "Human, Homo sapiens" Resistant Computational Prediction "catB7,blaPAO,blaOXA-50,fosA,aph(3')-Iib" 96.3 Good 1 1 0 1 1 1 1 0 0 0 0 0

Pseudomonas aeruginosa strain PHELES26 287 WGS PHELES26 "JACEMB010000001,JACEMB010000010,JACEMB010000011,JACEMB010000012,JACEMB010000013,JACEMB010000014,JACEMB010000015" 91 6491742 lung United Kingdom "Human, Homo sapiens" Resistant Computational Prediction "catB7,blaPAO,blaOXA-50,fosA,aph(3')-Iib" 95.7 Good 1 1 0 1 1 1 1 0 0 0 0 0

Pseudomonas aeruginosa strain PHELES24 287 WGS PHELES24 "JACELZ010000001,JACELZ010000010,JACELZ010000011,JACELZ010000012,JACELZ010000013,JACELZ010000014,JACELZ010000015" 98 6500280 lung United Kingdom "Human, Homo sapiens" Resistant Computational Prediction "catB7,blaPAO,blaOXA-50,fosA,aph(3')-Iib" 95.8 Good 1 1 0 1 1 1 1 0 0 0 0 0

Pseudomonas aeruginosa strain PHELES23 287 WGS PHELES23 "JACELY010000001,JACELY010000010,JACELY010000011,JACELY010000012,JACELY010000013,JACELY010000014,JACELY010000015" 89 6488609 lung United Kingdom "Human, Homo sapiens" Resistant Computational Prediction "catB7,blaPAO,blaOXA-50,fosA,aph(3')-Iib" 96 Good 1 1 0 1 1 1 1 0 0 0 0 0

Pseudomonas aeruginosa strain PHELES25 287 WGS PHELES25 "JACEMA010000001,JACEMA010000010,JACEMA010000011,JACEMA010000012,JACEMA010000013,JACEMA010000014,JACEMA010000015" 94 6459936 lung United Kingdom "Human, Homo sapiens" Resistant Computational Prediction "catB7,blaPAO,blaOXA-50,fosA,aph(3')-Iib" 96.2 Good 1 1 0 1 1 1 1 0 0 0 0 0

Pseudomonas aeruginosa AZPAE14372 287 WGS AZPAE14372 JTYY00000000 84 6794294 itra-abdominal tract infection China "Human, Homo sapiens" Resistant AMR Panel "catB7,blaPAO,blaOXA-50,fosA,aph(3')-Iib" 99.2 100 Good 1 1 0 1 1 1 1 0 0 0 0 0

Pseudomonas aeruginosa strain PHELES20 287 WGS PHELES20 "JACELV010000001,JACELV010000010,JACELV010000100,JACELV010000011,JACELV010000012,JACELV010000013,JACELV010000014" 100 6494441 lung United Kingdom "Human, Homo sapiens" Resistant Computational Prediction "catB7,blaPAO,blaOXA-50,fosA,aph(3')-Iib" 96.2 Good 1 1 0 1 1 1 1 0 0 0 0 0

Pseudomonas aeruginosa strain PHELES2 287 WGS PHELES2 "JACELU010000001,JACELU010000010,JACELU010000011,JACELU010000012,JACELU010000013,JACELU010000014,JACELU010000015" 86 6345013 lung United Kingdom "Human, Homo sapiens" Resistant Computational Prediction "catB7,blaPAO,blaOXA-50,fosA,aph(3')-Iib" 96.1 Good 1 1 0 1 1 1 1 0 0 0 0 0

Pseudomonas aeruginosa strain PAL1.49 287 WGS PAL1.49 "JACEPF010000010,JACEPF010000011,JACEPF010000012,JACEPF010000013,JACEPF010000014,JACEPF010000015,JACEPF010000016" 69 6936624 bronchial France "Human, Homo sapiens" Resistant Computational Prediction "crpP,catB7,blaPAO,blaOXA-396,fosA,aph(3')-Iib" 95.8 Good 1 1 1 1 1 1 1 0 0 0 0 1

Pseudomonas aeruginosa strain PAL1.45 287 WGS PAL1.45 "JACEPJ010000010,JACEPJ010000011,JACEPJ010000012,JACEPJ010000013,JACEPJ010000014,JACEPJ010000015,JACEPJ010000016" 83 6788527 France "Human, Homo sapiens" Resistant Computational Prediction "aadA11,ant(2'')-Ia,aac(6')-Il,aph(3')-Iib,sul1,dfrB1,fosA,catB7,blaPAO,blaOXA-488,qacE," 96.1 Good 1 1 0 1 1 1 1 0 0 1 0 0

Pseudomonas aeruginosa strain PAL1.39 287 WGS PAL1.39 "JACEPO010000100,JACEPO010000101,JACEPO010000102,JACEPO010000103,JACEPO010000104,JACEPO010000105,JACEPO010000106" 126 6826300 trachea France "Human, Homo sapiens" Resistant Computational Prediction "fosA,aph(3')-Iib,aac(6')-Il,aac(6')-31,ant(2'')-Ia,dfrB1,sul1,catB7,blaPAO,blaVIM-2,blaOXA-488,qacE" 95.8 Good 1 1 1 1 1 1 1 0 0 1 0 0

Pseudomonas aeruginosa strain PAL1.38 287 WGS PAL1.38 "JACEPP010000100,JACEPP010000101,JACEPP010000102,JACEPP010000103,JACEPP010000104,JACEPP010000105,JACEPP010000106" 123 7059166 trachea France "Human, Homo sapiens" Resistant Computational Prediction "crpP,fosA,aac(6')-Ib-cr,aph(3'')-Ib,tet(A),aph(6)-Id,aac(6')-Ib3,aac(3)-I,aph(3')-Iib,sul1,catB7,blaTEM-2,blaOXA-50,blaPAO,qacE" 95.3 Good 1 1 0 1 1 1 1 0 1 1 0 1

Pseudomonas aeruginosa strain PAL1.37 287 WGS PAL1.37 "JACEPQ010000010,JACEPQ010000011,JACEPQ010000012,JACEPQ010000013,JACEPQ010000014,JACEPQ010000015,JACEPQ010000016" 84 7194401 trachea France "Human, Homo sapiens" Resistant Computational Prediction "crpP,aac(6')-Ib-cr,aac(6')-Ib3,aph(3'')-Ib,aph(6)-Id,aac(3)-I,aph(3')-Iib,fosA,sul1,catB7,blaPAO,blaTEM-2,blaOXA-494,blaOXA-396,qacE" 95 Good 1 1 1 1 1 1 1 0 0 1 0 1

Pseudomonas aeruginosa strain PAL1.28 287 WGS PAL1.28 "JACEPX010000010,JACEPX010000011,JACEPX010000012,JACEPX010000013,JACEPX010000014,JACEPX010000015,JACEPX010000016" 78 6640756 trachea France "Human, Homo sapiens" Resistant Computational Prediction "crpP,aph(3')-Iib,blaOXA-488,catB7,blaPAO,fosA," 95.7 Good 1 1 0 1 1 1 1 0 0 0 0 1

Pseudomonas aeruginosa AZPAE13880 287 WGS AZPAE13880 JTZC00000000 112 6855952 Mexico "Human, Homo sapiens" Resistant AMR Panel "crpP,catB7,aph(3')-Iib,blaPAO,fosA,blaOXA-488" 98.6 100 0.3 Good 1 1 0 1 1 1 1 0 0 0 0 1

Pseudomonas aeruginosa strain PAL1.23 287 WGS PAL1.23 "JACEQB010000100,JACEQB010000101,JACEQB010000102,JACEQB010000103,JACEQB010000104,JACEQB010000105,JACEQB010000106" 108 7094489 trachea France "Human, Homo sapiens" Resistant Computational Prediction "crpP,aadA2b,aph(3')-Iib,fosA,sul1,catB7,blaOXA-395,blaPAO,qacE" 95.8 Good 1 1 1 1 1 1 1 0 0 0 0 1

Pseudomonas aeruginosa strain PAL1.15 287 WGS PAL1.15 "JACEQH010000010,JACEQH010000011,JACEQH010000012,JACEQH010000013,JACEQH010000014,JACEQH010000015,JACEQH010000016" 91 7239501 urine France "Human, Homo sapiens" Resistant Computational Prediction "crpP,fosA,aac(6')-Ib-cr,aph(3')-Iib,aph(3'')-Ib,aac(6')-Ib3,aph(6)-Id,aac(3)-I,sul1,catB7,blaTEM-2,blaOXA-396,blaOXA-494,blaPAO,qacE" 95.2 Good 1 1 1 1 1 1 1 0 0 1 0 1

Pseudomonas aeruginosa strain PAL1.14 287 WGS PAL1.14 "JACEQI010000010,JACEQI010000011,JACEQI010000012,JACEQI010000013,JACEQI010000014,JACEQI010000015,JACEQI010000016" 64 6932288 trachea France "Human, Homo sapiens" Resistant Computational Prediction "crpP,fosA,aph(3')-Iib,ant(2'')-Ia,aph(3')-Ib,sul1,catB7,blaOXA-50,blaPAO,qacE" 95.8 Good 1 1 0 1 1 1 1 0 0 1 0 1

Pseudomonas aeruginosa AZPAE13879 287 WGS AZPAE13879 JTZD00000000 132 6948193 Argentina "Human, Homo sapiens" Resistant AMR Panel "fosA,catB7,aph(3')-Iib,blaPAO,blaOXA-488" 98.9 100 Good 1 1 0 1 1 1 1 0 0 1 0 0

Pseudomonas aeruginosa strain PAL1.6 287 WGS PAL1.6 "JACEQQ010000100,JACEQQ010000010,JACEQQ010000011,JACEQQ010000012,JACEQQ010000013,JACEQQ010000014,JACEQQ010000015" 100 7125275 sputum France "Human, Homo sapiens" Resistant Computational Prediction "crpP,aac(6')-Ib-cr,aph(3')-Iib,aac(6')-Ib3,aadA2b,sul1,fosA,blaCARB-2,blaOXA-395,blaPAO,qacE" 95.6 Good 1 1 1 1 1 1 0 0 0 0 0 1

Pseudomonas aeruginosa strain PAL1.2 287 WGS PAL1.2 "JACEQU010000010,JACEQU010000011,JACEQU010000012,JACEQU010000013,JACEQU010000014,JACEQU010000015,JACEQU010000016" 85 7201349 trachea France "Human, Homo sapiens" Resistant Computational Prediction "crpP,aph(3')-Iib,blaOXA-488,catB7,blaPAO,fosA," 95.8 Good 1 1 0 1 1 1 1 0 0 0 0 1

Pseudomonas aeruginosa AZPAE13877 287 WGS AZPAE13877 JTZE00000000 75 6788523 Romania "Human, Homo sapiens" Resistant AMR Panel "crpP,aph(3')-Iib,blaOXA-488,catB7,blaPAO,fosA," 99.3 100 Good 1 1 0 1 1 1 1 0 0 0 0 1

Pseudomonas aeruginosa strain PAL1.67 287 WGS PAL1.67 "JACEUY010000010,JACEUY010000011,JACEUY010000012,JACEUY010000013,JACEUY010000014,JACEUY010000015,JACEUY010000016" 58 6926712 sputum France "Human, Homo sapiens" Resistant Computational Prediction "crpP,aph(3')-Iib,aadA13,ant(2'')-Ia,fosA,sul1,catB7,blaPAO,blaOXA-50,qacE" 95.8 Good 1 1 0 1 1 1 1 0 0 1 0 1

Pseudomonas aeruginosa strain GIMC5034:PA52Ts32 287 Complete GIMC5034:PA52Ts32 "CP059063,CP059062" 2 6824624 tracheal aspirate Russia "Human, Homo sapiens" Resistant Computational Prediction "crpP,aadA1,aph(3')-Iib,ant(2'')-Ia,aac(6')-Il,fosA,sul1,tet(A),tet(G),catB7,blaPAO,blaVEB-1,blaOXA-50,blaOXA-10,qacE," 95.8 Good 1 1 0 1 1 1 1 0 1 1 0 1

Pseudomonas aeruginosa AZPAE13876 287 WGS AZPAE13876 JTZF00000000 153 6795623 Portugal "Human, Homo sapiens" Resistant AMR Panel "crpP,fosA,catB7,blaPAO,blaOXA-395,aph(3')-Iib" 97.9 97.9 Good 1 1 1 1 1 1 1 0 0 1 0 1

Pseudomonas aeruginosa strain 401853 287 Complete 401853 CP059995 1 7092673 China "Human, Homo sapiens" Resistant Computational Prediction "crpP,aac(6')-Ib-cr,ant(2'')-Ia,aph(3')-Iib,aac(6')-Ib3,fosA,sul1,catB7,blaPAO,blaIMP-14,blaIMP-48,blaOXA-10,blaOXA-50,blaIMP-54,qacE" 95.4 Good 1 1 1 1 1 1 1 0 0 1 0 1

Pseudomonas aeruginosa strain PA0750 287 WGS PA0750 "JACJGW010000010,JACJGW010000011,JACJGW010000012,JACJGW010000013,JACJGW010000014,JACJGW010000015,JACJGW010000016" 54 6392362 hospital patient USA "Human, Homo sapiens" Resistant Computational Prediction "catB7,blaPAO,blaOXA-486,fosA,aph(3')-Iib" 96.5 Good 1 1 0 1 1 1 1 0 0 0 0 0

Pseudomonas aeruginosa AZPAE13872 287 WGS AZPAE13872 JTZG00000000 136 6958295 Mexico "Human, Homo sapiens" Resistant AMR Panel "crpP,catB7,blaPAO,blaOXA-488,fosA,aph(3')-Iib" 99.3 100 1.2 Good 1 1 0 1 1 1 1 0 0 0 0 1

Pseudomonas aeruginosa strain PA0750 287 WGS PA0750 "JACJGU010000010,JACJGU010000011,JACJGU010000012,JACJGU010000013,JACJGU010000014,JACJGU010000015,JACJGU010000016" 77 6832764 hospital patient USA "Human, Homo sapiens" Resistant Computational Prediction "crpP,aac(6')-Ib-cr,aadA1b,aac(6')-Ib-Hangzhou,aph(3')-IIbaac(6')-Ilaph(3')-Via,fosA,sul1,catB7,blaPAO,blaIMP-15,blaOXA-494,blaOXA-396,qacE" 95.7 Good 1 1 1 1 1 1 1 0 0 1 1 1

Pseudomonas aeruginosa AZPAE13864 287 WGS AZPAE13864 JTZI00000000 157 6909435 India "Human, Homo sapiens" Susceptible;Resistant AMR Panel "crpP,aac(6')-Ib-cr,aph(3')-Iib,aph(3'')-Ib,aac(6')-Ib3,ant(2'')-Ia,aph(6)-Id,aac(6')-Iai,fosA,sul1,dfrA1,cmx,catB7,blaPAO,blaOXA-396,blaOXA-494," 99.3 100 0.3 Good 1 1 1 1 1 1 1 0 0 1 0 1

Pseudomonas aeruginosa strain PHELES15 287 WGS PHELES15 "JACELP010000001,JACELP010000010,JACELP010000100,JACELP010000101,JACELP010000011,JACELP010000012,JACELP010000013" 101 6491969 lung United Kingdom "Human, Homo sapiens" Resistant Computational Prediction "catB7,fosA,aph(3')-Iib,blaPAO,blaOXA-50" 95.9 Good 1 1 0 1 1 1 1 0 0 0 0 0

Pseudomonas aeruginosa strain PHELES14 287 WGS PHELES14 "JACELO010000001,JACELO010000010,JACELO010000100,JACELO010000101,JACELO010000102,JACELO010000103,JACELO010000104" 105 6507922 lung United Kingdom "Human, Homo sapiens" Resistant Computational Prediction "catB7,fosA,aph(3')-Iib,blaPAO,blaOXA-50" 96.2 Good 1 1 0 1 1 1 1 0 0 0 0 0

Pseudomonas aeruginosa AZPAE13860 287 WGS AZPAE13860 JTZJ00000000 119 6888735 India "Human, Homo sapiens" Resistant AMR Panel "catB7,fosA,aph(3')-Iib,blaPAO,blaOXA-50" 99.5 100 Good 1 1 0 1 1 1 1 0 0 0 0 0

Pseudomonas aeruginosa strain LiP3 287 WGS LiP3 "JACELA010000001,JACELA010000010,JACELA010000011,JACELA010000012,JACELA010000013,JACELA010000014,JACELA010000015" 37 6455607 United Kingdom "Human, Homo sapiens" Resistant Computational Prediction "crpP,aph(3')-Via,aph(3')-Iib,ant(2'')-Ia,fosA,sul1,dfrA1,catB7,blaVEB-1,blaPAO,blaVIM-5,blaOXA-395,qacE" 96.1 Good 1 1 1 1 1 1 1 0 0 0 1 1

Pseudomonas aeruginosa strain PHELES13 287 WGS PHELES13 "JACELN010000001,JACELN010000010,JACELN010000100,JACELN010000101,JACELN010000102,JACELN010000103,JACELN010000104" 111 6473724 lung United Kingdom "Human, Homo sapiens" Resistant Computational Prediction "catB7,fosA,aph(3')-Iib,blaPAO,blaOXA-50" 95.9 Good 1 1 0 1 1 1 1 0 0 0 0 0

Pseudomonas aeruginosa strain 30642g 287 WGS 30642g "JACEKH010000001,JACEKH010000010,JACEKH010000011,JACEKH010000012,JACEKH010000013,JACEKH010000014,JACEKH010000015" 88 6440392 lung United Kingdom "Human, Homo sapiens" Resistant Computational Prediction "catB7,fosA,aph(3')-Iib,blaPAO,blaOXA-50" 96.2 Good 1 1 0 1 1 1 1 0 0 0 0 0

Pseudomonas aeruginosa AZPAE13858 287 WGS AZPAE13858 JTZK00000000 92 7017824 India "Human, Homo sapiens" Resistant;Susceptible AMR Panel "catB7,fosA,aph(3')-Iib,blaPAO,blaOXA-50" 99 100 0.6 Good 1 1 0 1 1 1 1 0 0 0 0 0

Pseudomonas aeruginosa strain 11112 287 WGS 11112 "JACEJG010000001,JACEJG010000010,JACEJG010000100,JACEJG010000011,JACEJG010000012,JACEJG010000013,JACEJG010000014" 100 6492581 lung United Kingdom "Human, Homo sapiens" Resistant Computational Prediction "catB7,fosA,aph(3')-Iib,blaPAO,blaOXA-50" 96.2 Good 1 1 0 1 1 1 1 0 0 0 0 0

Pseudomonas aeruginosa strain PAL1.47 287 WGS PAL1.47 "JACEPH010000010,JACEPH010000011,JACEPH010000012,JACEPH010000013,JACEPH010000014,JACEPH010000015,JACEPH010000016" 72 7240226 bronchial France "Human, Homo sapiens" Resistant Computational Prediction "crpP,fosA,aph(3')-Iib,aadA2b,ant(2'')-Ia,sul1,catB7,blaPAO,blaCARB-2,blaOXA-488,qacE" 95.3 Good 1 1 0 1 1 1 1 0 0 1 0 1

Pseudomonas aeruginosa strain PAL1.26 287 WGS PAL1.26 "JACEPZ010000100,JACEPZ010000101,JACEPZ010000102,JACEPZ010000103,JACEPZ010000104,JACEPZ010000105,JACEPZ010000106" 266 7556858 trachea France "Human, Homo sapiens" Resistant Computational Prediction "crpP,catB7,fosA,aph(3')-Iib,blaPAO,blaOXA-488" 93.6 Good 1 1 0 1 1 1 1 0 0 1 0 1

Pseudomonas aeruginosa AZPAE13856 287 WGS AZPAE13856 JTZL00000000 168 7373661 India "Human, Homo sapiens" Resistant AMR Panel "crpP,catB7,fosA,aph(3')-Iib,blaPAO,blaOXA-396,blaOXA-494" 98.4 99.7 Good 1 1 1 1 1 1 1 0 0 0 0 0

Pseudomonas aeruginosa strain CF023-Psa42 287 WGS CF023-Psa42 "JACEGH010000001,JACEGH010000160,JACEGH010000161,JACEGH010000162,JACEGH010000163,JACEGH010000164,JACEGH010000165" 327 6593126 Pharyngeal secretion Mexico "Human, Homo sapiens" Resistant Computational Prediction "crpP,fosA,aac(6')-Ib-cr,aac(6')-33,aph(3')-Iib,aac(6')-Ib3,aph(3')-Iia,aadA1b,aph(6)-Ic,sul1,catB7,blaPAO,blaGES-19,blaOXA-2,blaOXA-488,qacE" 94.8 Good 1 1 0 1 1 1 1 0 0 1 0 1

Pseudomonas aeruginosa AZPAE13853 287 WGS AZPAE13853 JTZM00000000 88 7040099 India "Human, Homo sapiens" Resistant;Susceptible AMR Panel "crpP,aph(3')-Ib,aac(3)-Id,aac(6')-Il,aph(3')-Iib.fosA,dfrB5,sul1,tet(G),catB7,blaPAO,blaVIM-2,blaOXA-488" 98.9 100 1.2 Good 1 1 1 1 1 1 1 0 1 1 0 1

Pseudomonas aeruginosa AZPAE13850 287 WGS AZPAE13850 JTZN00000000 372 5501656 India "Human, Homo sapiens" Resistant AMR Panel "crpP,aac(3)-Ic,aph(3')-Iib,aac(6')-Il,aph(3')-Iib,fosA,sul1,cmlA1,blaOXA-488,blaPAO,qacE" 93.3 85.9 Good 1 1 0 1 1 1 1 0 0 1 0 1

Pseudomonas aeruginosa AZPAE13848 287 WGS AZPAE13848 JTZO00000000 110 6958460 India "Human, Homo sapiens" Resistant AMR Panel "crpP,aac(6')-Ib-cr,fosA,tet(G),aph(3')-Iib,aph(6)-Id,aac(6')-Ib-Hangzhou,aph(3'')-Ib,rmtF,catB7,blaPAO,blaOXA-395,blaGES-9," 98.7 100 0.6 Good 1 1 1 1 1 1 1 0 1 1 0 1

Pseudomonas aeruginosa AZPAE13757 287 WGS AZPAE13757 JTZP00000000 64 6469470 respiratory tract infection Canada "Human, Homo sapiens" Resistant;Susceptible AMR Panel "catB7,fosA,aph(3')-Iib,blaPAO,blaOXA-50" 99.3 99.7 0.6 Good 1 1 0 1 1 1 1 0 0 0 0 0

Pseudomonas aeruginosa AZPAE13756 287 WGS AZPAE13756 JTZQ00000000 113 7335469 respiratory tract infection Canada "Human, Homo sapiens" Resistant AMR Panel "crpP,fosA,aph(3')-Iib,aac(6')-29a,sul1,catB7,blaPAO,blaVIM-2,blaOXA-395,qacE,blaIMP-18,blaOXA-2," 99 100 Good 1 1 1 1 1 1 1 0 0 0 0 1

Pseudomonas aeruginosa AZPAE12422 287 WGS AZPAE12422 JTZS00000000 119 6331130 cystic fibrosis isolate United States "Human, Homo sapiens" Susceptible;Resistant AMR Panel "catB7,fosA,aph(3')-Iib,blaPAO,blaOXA-395" 98 99.7 2.7 Good 1 1 1 1 1 1 1 0 0 0 0 0

Pseudomonas aeruginosa AZPAE12419 287 WGS AZPAE12419 JTZV00000000 89 6290772 cystic fibrosis isolate United States "Human, Homo sapiens" Susceptible;Intermediate AMR Panel "catB7,fosA,aph(3')-Iib,blaPAO,blaOXA-396,crpP" 99 99.7 Good 1 1 0 1 1 1 1 0 0 0 0 1

Pseudomonas aeruginosa AZPAE12417 287 WGS AZPAE12417 JTZX00000000 85 6360200 cystic fibrosis isolate United States "Human, Homo sapiens" Susceptible;Intermediate AMR Panel "catB7,fosA,aph(3')-Iib,blaPAO,blaOXA-396,crpP,blaOXA-50,blaOXA-494" 99.2 99.7 Good 1 1 1 1 1 1 1 0 0 0 0 1

Pseudomonas aeruginosa AZPAE12416 287 WGS AZPAE12416 JTZY00000000 124 6527924 cystic fibrosis isolate United States "Human, Homo sapiens" Resistant AMR Panel "catB7,fosA,aph(3')-Iib,blaPAO,crpP,blaOXA-88,blaOXA-485" 98.3 98.5 0.3 Good 1 1 0 1 1 1 1 0 0 0 0 1

Pseudomonas aeruginosa AZPAE12415 287 WGS AZPAE12415 JTZZ00000000 84 6740389 cystic fibrosis isolate United States "Human, Homo sapiens" Resistant;Susceptible AMR Panel "crpP,catB7,fosA,aph(3')-Iib,blaPAO,blaOXA-50" 97.6 99.4 1.2 Good 1 1 0 1 1 1 1 0 0 0 0 1

Pseudomonas aeruginosa AZPAE12414 287 WGS AZPAE12414 JUAA00000000 112 6736819 cystic fibrosis isolate United States "Human, Homo sapiens" Resistant;Susceptible AMR Panel "crpP,catB7,fosA,aph(3')-Iib,blaPAO,blaOXA-50" 97.1 99.7 1.3 Good 1 1 0 1 1 1 1 0 0 0 0 1

Pseudomonas aeruginosa AZPAE12413 287 WGS AZPAE12413 JUAB00000000 114 6742361 cystic fibrosis isolate United States "Human, Homo sapiens" Resistant;Susceptible AMR Panel "crpP,catB7,fosA,aph(3')-Iib,blaPAO,blaOXA-50" 97 98.5 1.8 Good 1 1 0 1 1 1 1 0 0 0 0 1

Pseudomonas aeruginosa AZPAE12410 287 WGS AZPAE12410 JUAE00000000 118 6457280 cystic fibrosis isolate United States "Human, Homo sapiens" Susceptible;Intermediate AMR Panel "crpP,catB7,fosA,aph(3')-Iib,blaPAO,blaOXA-488" 98.6 100 0.3 Good 1 1 0 1 1 1 1 0 0 0 0 1

Pseudomonas aeruginosa AZPAE12156 287 WGS AZPAE12156 JUAG00000000 199 6571733 cystic fibrosis isolate United States "Human, Homo sapiens" Resistant AMR Panel "crpP,catB7,fosA,aph(3')-Iib,blaPAO,blaOXA-494,blaOXA-396" 97.8 98.8 Good 1 1 1 1 1 1 1 0 0 0 0 1

Pseudomonas aeruginosa AZPAE12155 287 WGS AZPAE12155 JUAH00000000 138 6732273 cystic fibrosis isolate United States "Human, Homo sapiens" Susceptible;Intermediate AMR Panel "crpP,catB7,fosA,aph(3')-Iib,blaPAO,blaOXA-50" 98.3 100 0.6 Good 1 1 0 1 1 1 1 0 0 0 0 1

Pseudomonas aeruginosa AZPAE12154 287 WGS AZPAE12154 JUAI00000000 155 6299347 cystic fibrosis isolate United States "Human, Homo sapiens" Resistant;Intermediate;Susceptible AMR Panel "crpP,catB7,fosA,aph(3')-Iib,blaPAO,blaOXA-50" 99 99.1 0.3 Good 1 1 0 1 1 1 1 0 0 0 0 1

Pseudomonas aeruginosa AZPAE12151 287 WGS AZPAE12151 JUAL00000000 65 6332026 cystic fibrosis isolate United States "Human, Homo sapiens" Intermediate;Resistant;Susceptible AMR Panel "crpP,catB7,fosA,aph(3')-Iib,blaPAO,blaOXA-395" 98.7 99.7 0.6 Good 1 1 1 1 1 1 1 0 0 0 0 1

Pseudomonas aeruginosa AZPAE12150 287 WGS AZPAE12150 JUAM00000000 114 6594453 cystic fibrosis isolate United States "Human, Homo sapiens" Resistant AMR Panel "crpP,catB7,fosA,aph(3')-Iib,blaPAO,blaOXA-396" 98.4 99.4 0.3 Good 1 1 1 1 1 1 1 0 0 0 0 1

Pseudomonas aeruginosa AZPAE12149 287 WGS AZPAE12149 JUAN00000000 83 6355203 cystic fibrosis isolate United States "Human, Homo sapiens" Resistant AMR Panel "crpP,catB7,fosA,aph(3')-Iib,blaPAO,blaOXA-396,blaOXA-494" 98.5 100 1.5 Good 1 1 1 1 1 1 1 0 0 0 0 1

Pseudomonas aeruginosa AZPAE12148 287 WGS AZPAE12148 JUAO00000000 54 6650984 cystic fibrosis isolate United States "Human, Homo sapiens" Susceptible;Resistant AMR Panel "catB7,fosA,aph(3')-Iib,blaPAO,blaOXA-50" 99 100 Good 1 1 0 1 1 1 1 0 0 0 0 0

Pseudomonas aeruginosa AZPAE12147 287 WGS AZPAE12147 JUAP00000000 114 6522044 cystic fibrosis isolate United States "Human, Homo sapiens" Resistant;Intermediate;Susceptible AMR Panel "crpP,fosA,aadA2,ant(2'')-Ia,aph(3')-Iib,sul1,catB7,catA1,blaPAO,blaOXA-396,qacE,blaOXA-494" 98.5 99.4 0.6 Good 1 1 1 1 1 1 1 0 0 1 0 1

Pseudomonas aeruginosa AZPAE12145 287 WGS AZPAE12145 JUAR00000000 62 6452923 cystic fibrosis isolate United States "Human, Homo sapiens" Resistant;Intermediate AMR Panel "catB7,fosA,aph(3')-Iib,blaPAO,blaOXA-50" 99.2 100 0.9 Good 1 1 0 1 1 1 1 0 0 0 0 0

Pseudomonas aeruginosa AZPAE12143 287 WGS AZPAE12143 JUAT00000000 103 6473536 cystic fibrosis isolate United States "Human, Homo sapiens" Intermediate;Susceptible AMR Panel "crpP,catB7,fosA,aph(3')-Iib,blaPAO,blaOXA-50" 98.7 99.7 Good 1 1 0 1 1 1 1 0 0 1 0 0

Pseudomonas aeruginosa AZPAE12142 287 WGS AZPAE12142 JUAU00000000 99 6323807 cystic fibrosis isolate United States "Human, Homo sapiens" Resistant;Intermediate;Susceptible AMR Panel "catB7,fosA,aph(3')-Iib,blaPAO,blaOXA-396,blaOXA-494" 99.4 99.4 Good 1 1 1 1 1 1 1 0 0 0 0 0

Pseudomonas aeruginosa AZPAE12140 287 WGS AZPAE12140 JUAV00000000 54 6250380 cystic fibrosis isolate United States "Human, Homo sapiens" Resistant;Susceptible;Intermediate AMR Panel "catB7,fosA,aph(3')-Iib,blaPAO,blaOXA-50" 99.1 99.7 Good 1 1 0 1 1 1 1 0 0 0 0 0

Pseudomonas aeruginosa AZPAE12137 287 WGS AZPAE12137 JUAX00000000 82 6304247 cystic fibrosis isolate United States "Human, Homo sapiens" Resistant;Intermediate;Susceptible AMR Panel "catB7,fosA,aph(3')-Iib,blaPAO,blaOXA-395" 99.3 100 Good 1 1 1 1 1 1 1 0 0 0 0 0

Pseudomonas aeruginosa strain P469 287 WGS P469 "JACTAE010000115,JACTAE010000001,JACTAE010000010,JACTAE010000100,JACTAE010000101,JACTAE010000102,JACTAE010000103" 216 6817219 Secretion Chile "Human, Homo sapiens" Resistant Computational Prediction "fosA,msr€,aadA1,aph(3')-Iib,aac(6')-Ib-cr,aac(6')-Ib3,aph(6)-Id,blaPAU-1,sul1,catB7,blaOXA-396,blaPAO,blaVIM-2,blaOXA-10,blaTEM-1B,blaNDM-1,mph€,mph(A),qacE" 98.9 Good 1 1 1 1 1 1 1 1 0 0 0 1

Pseudomonas aeruginosa strain SRS1 287 WGS SRS1 "JACXLF010000001,JACXLF010000002,JACXLF010000003,JACXLF010000004,JACXLF010000005,JACXLF010000006,JACXLF010000007" 179 6850324 Bangladesh "Human, Homo sapiens" Resistant Computational Prediction "crpP,fosA,aadA1,aac(6')-Il,sul1,ant(2'')-Ia,aph(3')-Iib,dfrB2,tet(G),tet(A),catB7,blaPAO,blaOXA-395,blaOXA-10,blaVEB-1,blaPME-1,qacE" 98.2 Good 1 1 1 1 1 1 1 0 1 1 0 1

Pseudomonas aeruginosa strain PA179 287 Complete PA179 CP058257 1 6580692 blood Saudi Arabia "Human, Homo sapiens" Resistant Computational Prediction "fosA,catB7,blaPAO,aph(3')-Iib,blaOXA-50" 99.2 Good 1 1 0 1 1 1 1 0 0 0 0 0

Pseudomonas aeruginosa strain R31 287 Complete R31 "CP061850,CP061851" 2 6923280 sputum China Resistant Computational Prediction "crpP,catB7,aph(3')-Iib,blaPAO,blaOXA-486,blaKPC-2,fosA" 97.1 Good 1 1 1 1 1 1 1 0 0 0 0 1

Pseudomonas aeruginosa strain JT86 287 Complete JT86 CP062219 1 6520277 soil China Resistant Computational Prediction "fosA,catB7,blaPAO,aph(3')-Iib,blaOXA-50" 99.6 Good 1 1 0 1 1 1 1 0 0 0 0 0

Pseudomonas aeruginosa strain 6-38 287 WGS 6-38 "JABDUD010000001,JABDUD010000010,JABDUD010000100,JABDUD010000101,JABDUD010000102,JABDUD010000103,JABDUD010000104" 277 6886718 Intra-abdominal infection Portugal "Human, Homo sapiens" Resistant Computational Prediction "crpP,aadA7,aph(3')-Iib,fosA,sul1,catB7,blaOXA-488,blapAO,qacE" 96.8 Good 1 1 0 1 1 1 1 0 0 0 0 1

Pseudomonas aeruginosa strain 5-23 287 WGS 5-23 "JABDUG010000001,JABDUG010000010,JABDUG010000100,JABDUG010000101,JABDUG010000102,JABDUG010000103,JABDUG010000104" 398 6919984 Low respiratory tract infection Portugal "Human, Homo sapiens" Resistant Computational Prediction "crpP,fosA,catB7,blaPAO,aph(3')-Iib,blaOXA-396,blaOXA-494" 96.1 Good 1 1 1 1 1 1 1 0 0 0 0 1

Pseudomonas aeruginosa strain 4-92 287 WGS 4-92 "JABDUK010000001,JABDUK010000010,JABDUK010000100,JABDUK010000101,JABDUK010000102,JABDUK010000103,JABDUK010000104" 333 6760891 Urinary tract infection Portugal "Human, Homo sapiens" Resistant Computational Prediction "aadA11,aph(3')-Iib,ant(2'')-Ia,aac(6')-Il,fosA,sul1,dfrB1,catB7,blaPAO,blaOXA-488,blaGES-13,blaGES-6,blaGES-7,blaGES-5,qacE" 96.2 Good 1 1 0 1 1 1 1 0 0 1 0 0

Pseudomonas aeruginosa strain 4-93 287 WGS 4-93 "JABDUJ010000001,JABDUJ010000010,JABDUJ010000100,JABDUJ010000101,JABDUJ010000102,JABDUJ010000103,JABDUJ010000104" 512 6744172 Urinary tract infection Portugal "Human, Homo sapiens" Resistant Computational Prediction "aadA11,aph(3')-Iib,ant(2'')-Ia,aac(6')-Il,fosA,sul1,dfrB1,catB7,blaPAO,blaOXA-488,blaGES-13,blaGES-6,blaGES-7,blaGES-5,qacE" 94.1 Good 1 1 0 1 1 1 1 0 0 1 0 0

Pseudomonas aeruginosa strain 5-15 287 WGS 5-15 "JABDUH010000001,JABDUH010000010,JABDUH010000100,JABDUH010000101,JABDUH010000102,JABDUH010000103,JABDUH010000104" 886 6760892 Intra-abdominal infection Portugal "Human, Homo sapiens" Resistant Computational Prediction "crpP,aac(6')-Il,aph(3')-Iib,aac(6')-31,aadA6,fosA,sul1,catB7,blaOXA-488,blaOXA-2,blaPAO,qacE" 92.3 Good 1 1 0 1 1 1 1 0 0 1 0 1

Pseudomonas aeruginosa strain 4-94 287 WGS 4-94 "JABDUI010000001,JABDUI010000010,JABDUI010000100,JABDUI010000101,JABDUI010000102,JABDUI010000103,JABDUI010000104" 795 6715242 Intra-abdominal infection Portugal "Human, Homo sapiens" Resistant Computational Prediction "fosA,ant(2'')-Ia,aph(3')-Iib,aac(6')-Il,aadA11,sul1,dfrB1,catB7,blaPAO,blaOXA-488,blaGES-7,blaGES-6,blaGES-5,blaGES-13,qacE" 93.1 Good 1 1 0 1 1 1 1 0 0 1 0 0

Pseudomonas aeruginosa strain 4-86 287 WGS 4-86 "JABDUL010000001,JABDUL010000010,JABDUL010000100,JABDUL010000101,JABDUL010000102,JABDUL010000103,JABDUL010000104" 195 6777176 Intra-abdominal infection Portugal "Human, Homo sapiens" Resistant Computational Prediction "fosA,ant(2'')-Ia,aph(3')-Iib,aac(6')-Il,aadA11,sul1,dfrB1,catB7,blaPAO,blaOXA-488,blaGES-7,blaGES-6,blaGES-5,blaGES-13,qacE" 98 Good 1 1 0 1 1 1 1 0 0 1 0 0

Pseudomonas aeruginosa strain 4-79 287 WGS 4-79 "JABDUM010000001,JABDUM010000010,JABDUM010000100,JABDUM010000101,JABDUM010000102,JABDUM010000103,JABDUM010000104" 145 6777316 Urinary tract infection Portugal "Human, Homo sapiens" Resistant Computational Prediction "fosA,ant(2'')-Ia,aph(3')-Iib,aac(6')-Il,aadA11,sul1,dfrB1,catB7,blaPAO,blaOXA-488,blaGES-7,blaGES-6,blaGES-5,blaGES-13,qacE" 98.2 Good 1 1 0 1 1 1 1 0 0 1 0 0

Pseudomonas aeruginosa strain 4-17 287 WGS 4-17 "JABDUP010000001,JABDUP010000010,JABDUP010000100,JABDUP010000101,JABDUP010000102,JABDUP010000103,JABDUP010000104" 435 6746936 Urinary tract infection Portugal "Human, Homo sapiens" Resistant Computational Prediction "fosA,ant(2'')-Ia,aph(3')-Iib,aac(6')-Il,aadA11,sul1,dfrB1,catB7,blaPAO,blaOXA-488,blaGES-7,blaGES-6,blaGES-5,blaGES-13,qacE" 95.1 Good 1 1 0 1 1 1 1 0 0 1 0 0

Pseudomonas aeruginosa strain 4-120 287 WGS 4-120 "JABDUT010000001,JABDUT010000010,JABDUT010000100,JABDUT010000101,JABDUT010000102,JABDUT010000103,JABDUT010000104" 180 6782398 Urinary tract infection Portugal "Human, Homo sapiens" Resistant Computational Prediction "fosA,ant(2'')-Ia,aph(3')-Iib,aac(6')-Il,aadA11,sul1,dfrB1,catB7,blaPAO,blaOXA-488,blaGES-7,blaGES-6,blaGES-5,blaGES-13,qacE" 98.1 Good 1 1 0 1 1 1 1 0 0 1 0 0

Pseudomonas aeruginosa strain 4-71 287 WGS 4-71 "JABDUN010000001,JABDUN010000010,JABDUN010000100,JABDUN010000101,JABDUN010000102,JABDUN010000103,JABDUN010000104" 290 6768358 Intra-abdominal infection Portugal "Human, Homo sapiens" Resistant Computational Prediction "fosA,ant(2'')-Ia,aph(3')-Iib,aac(6')-Il,aadA11,sul1,dfrB1,catB7,blaPAO,blaOXA-488,blaGES-7,blaGES-6,blaGES-5,blaGES-13,qacE" 95.7 Good 1 1 0 1 1 1 1 0 0 1 0 0

Pseudomonas aeruginosa strain 3-58 287 WGS 3-58 "JABDUV010000001,JABDUV010000010,JABDUV010000100,JABDUV010000101,JABDUV010000102,JABDUV010000103,JABDUV010000104" 487 6536342 Intra-abdominal infection Portugal "Human, Homo sapiens" Resistant Computational Prediction "crpP,aac(6')-Ib-cr,fosA,ARR-3,aadA6,sul1,aph(3')-Iib,catB3,catB7,blaPAO,blaOXA-1,blaOXA-396,blaOXA-494" 94.5 Good 1 1 1 1 1 1 1 0 0 0 0 1

Pseudomonas aeruginosa strain 3-49 287 WGS 3-49 "JABDUX010000001,JABDUX010000010,JABDUX010000100,JABDUX010000101,JABDUX010000102,JABDUX010000103,JABDUX010000104" 665 6741104 Intra-abdominal infection Portugal "Human, Homo sapiens" Resistant Computational Prediction "fosA,ant(2'')-Ia,aph(3')-Iib,aac(6')-Il,aadA11,sul1,dfrB1,catB7,blaPAO,blaOXA-488,blaGES-7,blaGES-6,blaGES-5,blaGES-13,qacE" 93.7 Good 1 1 0 1 1 1 1 0 0 1 0 0

Pseudomonas aeruginosa strain 3-38 287 WGS 3-38 "JABDUZ010000001,JABDUZ010000010,JABDUZ010000100,JABDUZ010000101,JABDUZ010000102,JABDUZ010000103,JABDUZ010000104" 511 6745386 Low respiratory tract infection Portugal "Human, Homo sapiens" Resistant Computational Prediction "crpP,aac(6')-Ib-cr,fosA,ARR-3,aadA6,sul1,aph(3')-Iib,catB3,catB7,blaPAO,blaOXA-1,blaOXA-396,blaOXA-494" 94.5 Good 1 1 1 1 1 1 1 0 0 0 0 1

Pseudomonas aeruginosa strain 1-13 287 WGS 1-13 "JABDVD010000001,JABDVD010000010,JABDVD010000100,JABDVD010000101,JABDVD010000102,JABDVD010000103,JABDVD010000104" 307 6801915 Intra-abdominal infection Portugal "Human, Homo sapiens" Resistant Computational Prediction "fosA,ant(2'')-Ia,aph(3')-Iib,aac(6')-Il,aadA11,sul1,dfrB1,catB7,blaPAO,blaOXA-488,blaGES-7,blaGES-6,blaGES-5,blaGES-13,qacE" 96.5 Good 1 1 0 1 1 1 1 0 0 1 0 0

Pseudomonas aeruginosa strain 10-58 287 WGS 10-58 "JABDVG010000001,JABDVG010000010,JABDVG010000100,JABDVG010000101,JABDVG010000102,JABDVG010000103,JABDVG010000104" 302 6787579 Low respiratory tract infection Portugal "Human, Homo sapiens" Resistant Computational Prediction "crpP,fosA,catB7,blaPAO,aph(3')-Iib,blaOXA-494,blaOXA-396" 97.5 Good 1 1 1 1 1 1 1 0 0 0 0 1

Pseudomonas aeruginosa strain 2-29 287 WGS 2-29 "JABDVA010000001,JABDVA010000010,JABDVA010000100,JABDVA010000101,JABDVA010000102,JABDVA010000103,JABDVA010000104" 585 6735296 Urinary tract infection Portugal "Human, Homo sapiens" Resistant Computational Prediction "aadA11,aph(3')-Iib,ant(2'')-Ia,aac(6')-Il,fosA,sul1,dfrB1,catB7,blaPAO,blaOXA-488,blaGES-13,blaGES-6,blaGES-7,blaGES-5,qacE" 93.9 Good 1 1 0 1 1 1 1 0 0 1 0 0

Pseudomonas aeruginosa strain NEF156 287 WGS NEF156 WODV01000000 172 7099625 Italy "Human, Homo sapiens" Resistant Computational Prediction "crpP,fosA,sul1,aph(3')-Iib,catB7,blaOXA-488,blaPAO,qacE" 98.8 Good 1 1 0 1 1 1 1 0 0 0 0 1

Pseudomonas aeruginosa strain MINF_7A-sc-2280434 287 Complete MINF_7A-sc-2280434 LR890619 1 6270455 Resistant Computational Prediction "fosA,aph(3')-Iib,catB7,blaOXA-396,blaPAO," 98.7 Good 1 1 1 1 1 1 1 0 0 0 0 0

Pseudomonas aeruginosa strain CCBH28850 287 WGS CCBH28850 "JADPHL010000001,JADPHL010000010,JADPHL010000100,JADPHL010000101,JADPHL010000102,JADPHL010000103,JADPHL010000104" 227 6812677 blood Brazil "Human, Homo sapiens" Resistant Computational Prediction "fosA,aac(6')-Ib-cr,aac(6')-Ib3,aph(3')-Iib,aac(6')-Iq,dfrA21,sul1,catB7,cmx,blaKPC-2,blaGES-1,blaPAO,blaOXA-50,qacE" 99.4 Good 1 1 1 1 1 1 1 0 0 1 0 1

Pseudomonas aeruginosa strain CCBH27928 287 WGS CCBH27928 "JADPHX010000100,JADPHX010000101,JADPHX010000102,JADPHX010000103,JADPHX010000104,JADPHX010000105,JADPHX010000106" 598 7610463 blood Brazil "Human, Homo sapiens" Resistant Computational Prediction "crpP,aac(6')-Ib-cr,fosA,aac(6')-Ib3,aph(3')-Iib,ere(A),sul1,catB7,blaPAO,blaOXA-101,blaOXA-56,blaIMP-56,blaOXA-396,qacE" 95.9 Good 1 1 1 1 1 1 1 1 0 0 0 1

Pseudomonas aeruginosa strain B-I-1 287 Complete B-I-1 CP060242 1 6935851 urine France "Human, Homo sapiens" Resistant Computational Prediction "aadA11,aac(6')-Ib-cr,aph(3')-Iib,ant(2'')-Ia,aac(6')-Ib3,fosA,sul1,v,blaPAO,blaOXA-488,blaOXA-35,qacE" 98.2 Good 1 1 0 1 1 1 0 0 0 1 0 1

Pseudomonas aeruginosa strain A-I-1 287 Complete A-I-1 CP060243 1 7087087 urine France "Human, Homo sapiens" Resistant Computational Prediction "crpP,aac(6')-Ib-cr,aac(6')-Ib3,aph(3')-Iib,aadA2b,fosA,sul1,blaCARB-2,catB7,blaPAO,blaOXA-395,qacE" 99 Good 1 0 1 1 1 1 1 0 0 0 0 1

Pseudomonas aeruginosa strain LVP25 287 WGS LVP25 "JACHQX010000001,JACHQX010000002,JACHQX010000003,JACHQX010000004,JACHQX010000005,JACHQX010000006,JACHQX010000009" 9 7067473 eye India "Human, Homo sapiens" Resistant Computational Prediction "addA1,msr€,aac(6')-Il,aph(3')-Iib,ant(2'')-Ia,aph(3')-VI,aph(6)-Id,aph(3'')-Ib,fosA,dfrB2,mph€,sul1,dfrA1,tet(A),catB7,cmlA1,ARR-3,blaNDM-1,blaVEB-1,blaPAO,blaOXA-10,blaOXA-50,qacE,crpP" 85 Good 1 1 1 1 1 1 1 1 1 1 0 1

Pseudomonas aeruginosa strain E-I-1 287 WGS E-I-1 "JACKWS010000001,JACKWS010000002" 2 7079389 urine France "Human, Homo sapiens" Resistant Computational Prediction "catB7,aph(3')-Iib,fosA,tet(G),blaPAO,blaOXA-488" 98.7 Good 1 1 0 1 1 1 1 0 1 1 0 1

Pseudomonas aeruginosa strain P33 287 Complete P33 "CP065412,CP065413,CP065414,CP065415,CP065416" 5 7117586 China "Human, Homo sapiens" Resistant Computational Prediction "crpP,fosA,vant(2'')-Ia,aac(6')-Iia,sul1,catB7,blaCARB-2,blaKPC-2,blaPAO,qacE" 99.3 Good 1 1 1 1 1 1 1 0 0 1 0 1

Pseudomonas aeruginosa strain P23 287 Complete P23 "CP065417,CP065418" 2 6950191 China "Human, Homo sapiens" Resistant Computational Prediction "crpP,fosA,aph(3')-Iib,catB7,blaOXA-486,blaKPC-2,blaPAO," 99.3 Good 1 1 1 1 1 1 1 0 0 0 0 1

Pseudomonas aeruginosa strain 39648-20 287 WGS 39648-20 "JADZIB010000099,JADZIB010000100,JADZIB010000101,JADZIB010000102,JADZIB010000103,JADZIB010000104,JADZIB010000105" 131 7077485 Russia "Human, Homo sapiens" Resistant Computational Prediction "crpP,aac(6')-29b,aph(3')-Iib,fosA,sul1,catB7,blaOXA-395,blaVIM-2,blaPAO,qacE" 99 Good 1 1 1 1 1 1 1 0 0 0 0 1

Pseudomonas aeruginosa strain CCBH28865 287 WGS CCBH28865 "JAECSD010000001,JAECSD010000010,JAECSD010000100,JAECSD010000101,JAECSD010000102,JAECSD010000103,JAECSD010000104" 211 6838033 blood Brazil "Human, Homo sapiens" Resistant Computational Prediction "fosA,msr€,aph(3')-Iib,aac(6')-Il,aac(3)-Id,mph€,dfrB5,catB7,blaPAO,blaOXA-486,blaVIM-2,qacE" 98.6 Good 1 1 1 1 1 1 1 1 0 1 0 0

Pseudomonas aeruginosa strain CCBH28717 287 WGS CCBH28717 "JAECSF010000001,JAECSF010000010,JAECSF010000100,JAECSF010000101,JAECSF010000102,JAECSF010000103,JAECSF010000104" 196 6881183 blood Brazil "Human, Homo sapiens" Resistant Computational Prediction "crpP,fosA,aadA2,aac(6')-Il,aac(3)-Id,aph(3')-Iib,dfrB5,tet(G),cmlA1,catB7,blaVIM-2,blaPAO,blaOXA-486,qacE" 98.9 Good 1 1 1 1 1 1 1 0 1 1 0 1

Pseudomonas aeruginosa strain CCBH28242 287 WGS CCBH28242 "JAECSH010000001,JAECSH010000010,JAECSH010000100,JAECSH010000101,JAECSH010000102,JAECSH010000103,JAECSH010000104" 265 6893074 Catheter Tip Brazil "Human, Homo sapiens" Resistant Computational Prediction "crpP,fosA,aadA2,aac(6')-Il,aac(3)-Id,aph(3')-Iib,dfrB5,sul1,tet(G),cmlA1,catB7,blaVIM-2,blaPAO,blaOXA-486,qacE,blaOXA-4" 98.2 Good 1 1 1 1 1 1 1 0 1 1 0 1

Pseudomonas aeruginosa strain CCBH27919 287 WGS CCBH27919 "JAECSQ010000101,JAECSQ010000102,JAECSQ010000103,JAECSQ010000104,JAECSQ010000105,JAECSQ010000106,JAECSQ010000107" 533 6979135 blood Brazil "Human, Homo sapiens" Resistant Computational Prediction "crpP,fosA,aac(6')-Il,aac(3)-Id,aph(3')-Iib,dfrB5,catB7,blaVIM-2,blaPAO,blaOXA-486,blaOXA-4" 95 Good 1 1 1 1 1 1 1 0 0 1 0 1

Pseudomonas aeruginosa strain CCBH27678 287 WGS CCBH27678 "JAECSX010000100,JAECSX010000101,JAECSX010000102,JAECSX010000103,JAECSX010000104,JAECSX010000105,JAECSX010000106" 324 6972554 blood Brazil "Human, Homo sapiens" Resistant Computational Prediction "fosA,aac(6')-Ib-cr,aac(6')-Iq,aph(3')-Iib,aac(6')-Ib3,dfrA21,sul1,catB7,cmx,blaGES-1,blaPAO,blaOXA-50,qace" 96.8 Good 1 1 0 1 1 1 1 0 0 0 0 1

Pseudomonas aeruginosa strain CCBH27615 287 WGS CCBH27615 "JAECTE010000100,JAECTE010000101,JAECTE010000102,JAECTE010000103,JAECTE010000104,JAECTE010000105,JAECTE010000106" 362 7082807 blood Brazil "Human, Homo sapiens" Resistant Computational Prediction "crpP,fosA,catB7,blaPAO,blaOXA-50,aph(3')-Iib,blaKPC-2,aph(3'')-Ib,aph(3')-Via" 94 Good 1 1 1 1 1 1 1 0 0 1 1 1

Pseudomonas aeruginosa strain CCBH27339 287 WGS CCBH27339 "JAECTS010000097,JAECTS010000098,JAECTS010000099,JAECTS010000100,JAECTS010000101,JAECTS010000102,JAECTS010000103" 108 6889343 blood Brazil "Human, Homo sapiens" Resistant Computational Prediction "catB7,qacE,blaOXA-494,blaOXA-396,blaSPM-1,blaOXA-56,blaPAO,crpP,aadA7,aac(6')-Ib-cr,aph(3')-Iib,aac(6')-Ib3,rmtD,fosA,sul1" 98.8 Good 1 1 1 1 1 1 1 0 0 1 0 1

Pseudomonas aeruginosa strain CCBH27346 287 WGS CCBH27346 "JAECTR010000100,JAECTR010000101,JAECTR010000102,JAECTR010000103,JAECTR010000104,JAECTR010000105,JAECTR010000106" 186 6911115 blood Brazil "Human, Homo sapiens" Resistant Computational Prediction "fosA,v,aac(6')-Ib3,aph(3')-Iib,aac(6')-Iq,sul1,dfrA21,cmx,catB7,blaGES-1,blaPAO,blaKPC-2,blaOXA-50,qacE" 98.4 Good 1 1 1 1 1 1 1 0 0 0 0 1

Pseudomonas aeruginosa strain CCBH27337 287 WGS CCBH27337 "JAECTT010000097,JAECTT010000098,JAECTT010000099,JAECTT010000100,JAECTT010000101,JAECTT010000102,JAECTT010000103" 160 6910465 blood Brazil "Human, Homo sapiens" Resistant Computational Prediction "crpP,aac(6')-Ib-cr,rmtD,aadA7,aph(3')-Iib,fosA,sul1,aac(6')-Ib3,catB7,blaOXA-396,blaPAO,blaOXA-56,blaSPM-1,blaOXA-494,qacE," 98.3 Good 1 1 1 1 1 1 1 0 0 1 0 1

Pseudomonas aeruginosa strain CCBH26731 287 WGS CCBH26731 "JAECUE010000100,JAECUE010000101,JAECUE010000102,JAECUE010000103,JAECUE010000104,JAECUE010000105,JAECUE010000106" 140 7425370 blood Brazil "Human, Homo sapiens" Resistant Computational Prediction "fosA,msr€,aac(3)-Id,aac(6')-Ib3aadA2,aph(3')-Iib,aac(6')-Il,aac(6')-Ib-cr,ARR-3,mph(A),mph€,msr€,ere(A),sul1,dfrB5,dfrA27,catB7,tet(G),cmx,cmlA1,blaOXA-101,blaPAO,blaCARB-2,blaIMP-56,blaOXA-486,blaOXA-4,blaVIM-2,aadA2,crpP,qnrVC6,qacE" 98 Good 1 1 1 1 1 1 1 1 1 1 0 1

Pseudomonas aeruginosa strain CMC-097 287 Complete CMC-097 CP065848 1 7044064 tracheal aspirate USA "Human, Homo sapiens" Resistant Computational Prediction "crpP,aph(3')-Iib,aac(6')-Iic,fosA,sul1,blaPAO,blaOXA-50,blaOXA-2,qacE" 99 Good 1 1 1 1 0 1 0 0 0 1 0 1

Pseudomonas aeruginosa strain SPA02 287 WGS SPA02 "JAEMBW010000001,JAEMBW010000010,JAEMBW010000011,JAEMBW010000012,JAEMBW010000013,JAEMBW010000014,JAEMBW010000015" 24 6766710 Knee joint aspiration India "Human, Homo sapiens" Resistant Computational Prediction "sul1,fosA,catB7,blaPAO,blaOXA-396,aph(3')-Iib" 98.7 Good 1 1 1 1 1 1 1 0 0 0 0 0

Pseudomonas aeruginosa strain SPA03 287 WGS SPA03 "JAENHR010000001,JAENHR010000002,JAENHR010000003,JAENHR010000004,JAENHR010000005,JAENHR010000006,JAENHR010000007" 9 6829266 Per urether catheter urine India "Human, Homo sapiens" Resistant Computational Prediction "crpP,aph(3')-VI,aadA1,aph(3')-Iib,fosA,sul1,tet(A),cmlA1,catB7,ARR-3,blaNDM-1,blaOXA-50,blaPAO,blaVEB-1,qace" 99.3 Good 1 1 1 1 1 1 1 0 1 0 0 1

Pseudomonas aeruginosa strain MMRPA01 287 WGS MMRPA01 "JAEMUV010000001,JAEMUV010000010,JAEMUV010000100,JAEMUV010000101,JAEMUV010000102,JAEMUV010000103,JAEMUV010000104" 145 6808493 sputum Myanmar "Human, Homo sapiens" Resistant Computational Prediction "crpP,aac(6')-Ib3,aadA6,aph(3')-Iib,fosA,sul1,catB7,blaOXA-488,blaPAO,blaIMP-1,qace,aac(6')-Ib-cr," 99 Good 1 1 1 1 1 1 1 0 0 1 0 1

Pseudomonas aeruginosa strain PA19-3047 287 Complete PA19-3047 CP068239 1 6857397 sputum China male Resistant Computational Prediction "crpP,aac(6')-Ib-cr,ant(2'')-Ia,aadA6,aph(3')-Iib,aac(6')-Ib3,fosA,sul1,dfrA5,cmx,catB7,catB3,blaPER-4,blaPAO,blaOXA-488,blaOXA-129,blaOXA-17,qacE" 98.5 Good 1 1 0 1 1 1 1 0 0 1 0 1

Pseudomonas aeruginosa strain SE5357 287 Complete SE5357 CP054844 1 7043467 sputum China "Human, Homo sapiens" Resistant Computational Prediction "crpP,aac(6')-Ib-cr,aph(3')-Iib,aadA5,aac(6')-Ib3,aph(6)-Id,aph(3'')-Ib,fosA,sul1,catB7,blaPER-1,blaPAO,blaOXA-494,blaOXA-396,blaOXA-101,qacE" 98.1 Good 1 1 1 1 1 1 1 0 0 0 0 1

Pseudomonas aeruginosa strain SE5352 287 Complete SE5352 CP054843 1 6904218 sputum China "Human, Homo sapiens" Resistant Computational Prediction "fosA,aac(6')-Ib-cr,blaPAO,aph(3')-XV,aadA6,aph(3')-Iib,aac(6')-Ib3fosA,tet(G),sul1,catB7,blaGES-1,blaoXA-488qacE" 98.8 Good 1 1 1 1 1 1 1 0 1 1 0 1

Pseudomonas aeruginosa strain SE5429 287 Complete SE5429 CP054845 1 7103853 urine China "Human, Homo sapiens" Resistant Computational Prediction "crpP,aph(3')-VI,aph(3')-Iib,aadA1,fosA,sul1,catB7,blaOXA-10,blaOXA-488,blaVEB-3,qacE" 99 Good 1 1 1 1 1 1 1 0 0 1 0 1

Pseudomonas aeruginosa strain YTSEY8 287 Complete YTSEY8 CP054581 1 7177049 China "Human, Homo sapiens" Resistant Computational Prediction "crpP,aac(6')-Ib-cr,aph(6)-Id,ant(2'')-Ia,aac(6')-Ib3aadA2b,aph(3'')-Ib,aph(6)-Id,aac(6')-Iia,aph(3'')-Ib,aph(3')-Iib,tet(G),sul1,catB7,catB3,blaCARB-2,blaPAO,blaOXA-396,blaOXA-494,qacE,fosA" 98.6 Good 1 1 1 1 1 1 1 0 1 1 0 1

Pseudomonas aeruginosa strain 1903031130 287 Complete 1903031130 CP060392 1 6905506 sputum China "Human, Homo sapiens" Resistant Computational Prediction "fosA,aac(6')-Ib-cr,aadA6,aph(3')-Iib,aph(3')-XV,aac(6')-Ib3,sul1,aac(6')-Ib3,catB7,blaPAO,blaOXA-488,blaGES-15,qacE," 99 Good 1 1 0 1 1 1 1 0 1 1 0 1

Pseudomonas aeruginosa strain PA1120 287 WGS PA1120 "JAEVLV010000005,JAEVLV010000015,JAEVLV010000001,JAEVLV010000010,JAEVLV010000011,JAEVLV010000012,JAEVLV010000013" 19 7336059 sputum Homo sapiens Resistant Computational Prediction "crpP,fosA,catB7,blaKPC-2,blaPAO,blaOXA-488,aph(3')-Iib" 97 Good 1 1 1 1 1 1 1 0 0 0 0 1

Pseudomonas aeruginosa strain 152962 287 Complete 152962 CP069198 1 6950174 blood France Homo sapiens Resistant Computational Prediction "fosA,aph(6)-Id,aac(6')-Ib-cr,aph(3'')-Ib,ant(2'')-Ia,aph(3')-Iib,aac(6')-Ib-Hangzhou,sul1,sul2,dfrB5,tet(A),catB7,blaTEM-1B,blaPAO,blaCTX-M-15,blaOXA-396,blaVIM-2,qacE" 99.4 Good 1 1 1 1 1 1 1 0 1 1 0 1

Pseudomonas aeruginosa strain S33 287 WGS S33 "JAFBLH010000001,JAFBLH010000010,JAFBLH010000011,JAFBLH010000012,JAFBLH010000013,JAFBLH010000014,JAFBLH010000015" 28 7066852 sputum Homo sapiens Resistant Computational Prediction "crpP,aph(3')-Iib,catB7,blaKPC-2,blaOXA-486,blaPAO,fosA," 99.1 Good 1 1 1 1 1 1 1 0 0 0 0 1

Pseudomonas aeruginosa strain S77 287 WGS S77 "JAFBLJ010000001,JAFBLJ010000002,JAFBLJ010000003,JAFBLJ010000004,JAFBLJ010000005,JAFBLJ010000006,JAFBLJ010000007" 8 6891040 sputum Homo sapiens Resistant Computational Prediction "crpP,aph(3')-Iib,catB7,blaKPC-2,blaOXA-486,blaPAO,fosA," 98.8 Good 1 1 1 1 1 1 1 0 0 0 0 1

Pseudomonas aeruginosa strain S76 287 WGS S76 "JAFBLK010000001,JAFBLK010000002,JAFBLK010000003,JAFBLK010000004,JAFBLK010000005,JAFBLK010000006,JAFBLK010000007" 9 7053372 urine Homo sapiens Resistant Computational Prediction "crpP,aph(3')-Iib,catB7,blaKPC-2,blaOXA-486,blaPAO,fosA," 99.2 Good 1 1 1 1 1 1 1 0 0 0 0 1

Pseudomonas aeruginosa strain S72 287 WGS S72 "JAFBLL010000001,JAFBLL010000002,JAFBLL010000003,JAFBLL010000004,JAFBLL010000005,JAFBLL010000006,JAFBLL010000007" 8 6976991 sputum Homo sapiens Resistant Computational Prediction "crpP,aph(3')-Iib,catB7,blaKPC-2,blaOXA-486,blaPAO,fosA," 98.5 Good 1 1 1 1 1 1 1 0 0 0 0 1

Pseudomonas aeruginosa strain S28 287 WGS S28 "JAFBLN010000001,JAFBLN010000010,JAFBLN010000011,JAFBLN010000012,JAFBLN010000013,JAFBLN010000014,JAFBLN010000015" 23 7498812 sputum Homo sapiens Resistant Computational Prediction "crpP,aph(3')-Iib,catB7,blaKPC-2,blaOXA-486,blaPAO,fosA," 98.7 Good 1 1 1 1 1 1 1 0 0 0 0 1

Pseudomonas aeruginosa strain T3 287 WGS T3 "JAFCHL010000001,JAFCHL010000002,JAFCHL010000003,JAFCHL010000004,JAFCHL010000005,JAFCHL010000006" 6 6865446 Hospital Tap Water India Resistant Computational Prediction "fosA,aph(3'')-Ib,ant(2'')-Ia,aph(3')-Iib,aph(6)-Id,catB7,sul1,blaOXA-50" 99.1 Good 1 1 0 1 1 1 1 0 0 1 0 0

Pseudomonas aeruginosa strain T4 287 WGS T4 "JAFCHM010000001,JAFCHM010000002,JAFCHM010000003,JAFCHM010000004,JAFCHM010000005,JAFCHM010000006,JAFCHM010000007" 8 6865897 Hospital Tap Water India Resistant Computational Prediction "catB7,fosA,sul1,blapAO,blaOXA-50,aph(3')-Iib,aph(3'')-Ib,aph(6)-Id,ant(2'')-Ia," 99.1 Good 1 1 0 1 1 1 1 0 0 1 0 0

Pseudomonas aeruginosa strain 251 287 WGS 251 "JAFCHJ010000001,JAFCHJ010000002,JAFCHJ010000003,JAFCHJ010000004,JAFCHJ010000005,JAFCHJ010000006,JAFCHJ010000007" 9 6955433 Per urether catheter urine India Homo sapiens Resistant Computational Prediction "fosA,aac(6')-Ib-cr,sul1,rmtF,tet(G),aph(6)-Id,aph(3'')-Ib,aph(3')-Iib,aac(6')-Ib-Hangzhou,catB7,blaNDM-1,blaPAO,blaOXA-396,blaGES-9," 98.9 Good 1 1 1 1 1 1 1 0 0 1 0 1

Pseudomonas aeruginosa strain 255 287 WGS 255 "JAFCHK010000001,JAFCHK010000010,JAFCHK010000002,JAFCHK010000003,JAFCHK010000004,JAFCHK010000005,JAFCHK010000006" 10 6917762 Supra pubic catheter urine India Homo sapiens Resistant Computational Prediction "fosA,aac(6')-Ib-cr,sul1,rmtF,tet(G),aph(6)-Id,aph(3'')-Ib,aph(3')-Iib,aac(6')-Ib-Hangzhou,catB7,blaNDM-1,blaPAO,blaOXA-396,blaGES-9," 98.9 Good 1 1 1 1 1 1 1 0 1 1 0 1

Pseudomonas aeruginosa strain 239 287 WGS 239 "JAFCHH010000001,JAFCHH010000010,JAFCHH010000011,JAFCHH010000012,JAFCHH010000002,JAFCHH010000003,JAFCHH010000004" 12 6955249 Midstream urine India Homo sapiens Resistant Computational Prediction "fosA,aac(6')-Ib-cr,sul1,rmtF,tet(G),aph(6)-Id,aph(3'')-Ib,aph(3')-Iib,aac(6')-Ib-Hangzhou,catB7,blaNDM-1,blaPAO,blaOXA-396,blaGES-9," 98.9 Good 1 1 1 1 1 1 1 0 1 1 0 1

Pseudomonas aeruginosa strain 237 287 WGS 237 "JAFCHG010000001,JAFCHG010000010,JAFCHG010000011,JAFCHG010000012,JAFCHG010000013,JAFCHG010000014,JAFCHG010000002" 14 6959626 Pericardial fluid India Homo sapiens Resistant Computational Prediction "crpP,aac(6')-Ib-cr,fosA,ere(A),sul1,dfrA5,aph(3'')-Ib,aac(6')-Ib-Hangzhou,aph(6)-Id,aph(3')-Iib,rmtF,catB7,blaPAO,blaGES-9,blaOXA-395,qacE" 97.9 Good 1 1 1 1 1 1 1 1 1 1 0 1

Pseudomonas aeruginosa strain 236 287 WGS 236 "JAFCHF010000001,JAFCHF010000010,JAFCHF010000011,JAFCHF010000012,JAFCHF010000013,JAFCHF010000014,JAFCHF010000015" 20 6948035 Endotracheal tube India Homo sapiens Resistant Computational Prediction "crpP,aac(6')-Ib-cr,aph(3')-Iib,aph(3'')-Ib,rmTf,aph(6)-Id,aac(6')-Ib-Hangzhou,tet(G),ere(A),sul1,dfrA5,floR,catB7,blaPAO,blaOXA-395,blaGES-9,qacE,fosA" 98.1 Good 1 1 1 1 1 1 1 1 1 1 0 1

Pseudomonas aeruginosa strain 227 287 WGS 227 "JAFCHE010000001,JAFCHE010000010,JAFCHE010000011,JAFCHE010000012,JAFCHE010000013,JAFCHE010000014,JAFCHE010000015" 17 6701694 sputum India Homo sapiens Resistant Computational Prediction "aadA5,aac(6')-Ib-cr,aph(3')-Iib,aph(3')-Via,aadA6,aac(6')-Ib3,fosA,sul1,catB8,catB7,blapAO,blaOXA-10,blaVEB-1,blaOXA-488,qacE" 98.9 Good 1 1 0 1 1 1 1 0 0 1 1 1

Pseudomonas aeruginosa strain 217 287 WGS 217 "JAFCHC010000001,JAFCHC010000010,JAFCHC010000002,JAFCHC010000003,JAFCHC010000004,JAFCHC010000005,JAFCHC010000006" 10 6958100 Midstream urine India Homo sapiens Resistant Computational Prediction "rmtF,aac(6')-Ib-cr,aph(3'')-Ib,aph(3')-Iib,aph(6)-Id,aac(6')-Ib-Hangzhou,tet(G),sul1,blaOXA-396,catB7,blapAO,blaGES-9,blaNDM-1,fosA," 98.9 Good 1 1 1 1 1 1 1 0 1 1 0 1

Pseudomonas aeruginosa strain 225 287 WGS 225 "JAFCHD010000001,JAFCHD010000002,JAFCHD010000003,JAFCHD010000004,JAFCHD010000005,JAFCHD010000006,JAFCHD010000007" 9 6956667 Per urether catheter urine India Homo sapiens Resistant Computational Prediction "fosA,aac(6')-Ib-cr,aph(3'')-Ib,aph(3')-Iib,aph(6)-Id,aac(6')-Ib-Hangzhou,tet(G),rmtF,catB7,blaGES-9,blaOXA-396,blaNDM-1,blaPAO" 98.9 Good 1 1 1 1 1 1 1 0 1 1 0 1

Pseudomonas aeruginosa strain 208 287 WGS 208 "JAFCHB010000001,JAFCHB010000010,JAFCHB010000002,JAFCHB010000003,JAFCHB010000004,JAFCHB010000005,JAFCHB010000006" 10 6954895 Midstream urine India Homo sapiens Resistant Computational Prediction "fosA,aac(6')-Ib-cr,aph(3'')-Ib,aac(6')-Ib-Hangzhou,aph(3')-Iib,rmtF,aph(6)-Id,sul1,tet(G),catB7,blaPAO,blaOXA-396,blaNDM-1,blaGES-9," 98.9 Good 1 1 1 1 1 1 1 0 1 1 0 1

Pseudomonas aeruginosa strain 200 287 WGS 200 "JAFCHA010000001,JAFCHA010000010,JAFCHA010000002,JAFCHA010000003,JAFCHA010000004,JAFCHA010000005,JAFCHA010000006" 10 6955968 Midstream urine India Homo sapiens Resistant Computational Prediction "fosA,aac(6')-Ib-cr,aph(3'')-Ib,aac(6')-Ib-Hangzhou,aph(3')-Iib,rmtF,aph(6)-Id,sul1,tet(G),catB7,blaPAO,blaOXA-396,blaNDM-1,blaGES-9," 98.9 Good 1 1 1 1 1 1 1 0 1 1 0 1

Pseudomonas aeruginosa strain 199 287 WGS 199 "JAFCGZ010000001,JAFCGZ010000002,JAFCGZ010000003,JAFCGZ010000004,JAFCGZ010000005,JAFCGZ010000006" 6 6813180 Midstream urine India Homo sapiens Resistant Computational Prediction "fosA,sul1,aph(3')-Iib,tet(G),catB7,blaPAO,v,blaOXA-396" 98.5 Good 1 1 1 1 1 1 1 0 1 0 0 0

Pseudomonas aeruginosa strain CLB 24388 287 WGS CLB 24388 "JAFFGY010000001,JAFFGY010000010,JAFFGY010000011,JAFFGY010000012,JAFFGY010000013,JAFFGY010000014,JAFFGY010000015" 36 7091263 urine USA Homo sapiens Resistant;Not defined;Intermediate Computational Prediction "fosA,aac(6')-Ib-cr,aph(3')-Iib,aac(3)-I,aac(6')-Ib3,sul1,catB7,blaPAO,blaOXA-5,blaGES-2,blaOXA-50,qacE" 98.4 Good 1 1 0 1 1 1 1 0 0 1 0 1

Pseudomonas aeruginosa strain B17932 287 Complete B17932 "CP070471,CP070472,CP070473" 3 6850198 blood India Homo sapiens Resistant Computational Prediction "crpP,aadA1,aac(6')-Il,ant(2'')-Ia,aph(3')-Iib,aph(3')-VI,fosA,sul1,dfrB2,tet(A),catB7,blaPAO,blaOXA-10,blaNDM-1,blaVEB-1,blaOXA-50,qacE" 99.3 Good 1 1 1 1 1 1 1 0 1 1 0 1

Pseudomonas aeruginosa strain B17416 287 Complete B17416 "CP070467,CP070468,CP070469,CP070470" 4 6850181 blood India Homo sapiens Resistant Computational Prediction "crpP,aac(6')-Il,aph(3')-Iib,ant(2'')-Ia,aadA1,aph(3')-VI,fosA,dfrB2,sul1,catB7,tet(A),blaPAO,blaOXA-10,blaOXA_50,blaVEB-1,blaNDM-1,qacE" 99.3 Good 1 1 1 1 1 1 1 0 1 1 0 1

Pseudomonas aeruginosa strain KPA13 287 WGS KPA13 "JABTVR010000001,JABTVR010000002,JABTVR010000003,JABTVR010000004,JABTVR010000005,JABTVR010000006,JABTVR010000007" 258 6663633 Kenya Homo sapiens Resistant Computational Prediction "crpP,ant(2'')-Ia,aadA1,aph(3')-Iib,aac(6')-Il,aph(3')-VI,ARR-3,sul1,dfrB2,catB7,cmlA1,tet(A),blaPAO,blaOXA-10,blaOXA-50,blaVEB-1,blaNDM-1,qacE" 98.6 Good 1 1 1 1 1 1 1 0 1 1 0 1

Pseudomonas aeruginosa strain KPA12 287 WGS KPA12 "JABTVT010000001,JABTVT010000002,JABTVT010000003,JABTVT010000004,JABTVT010000005,JABTVT010000006,JABTVT010000007" 267 6656449 Kenya Homo sapiens Resistant Computational Prediction "crpP,ant(2'')-Ia,aadA1,aph(3')-Iib,aac(6')-Il,aph(3')-VI,ARR-3,sul1,dfrB2,catB7,cmlA1,tet(A),blaPAO,blaOXA-10,blaOXA-50,blaVEB-1,blaNDM-1,qacE" 98.7 Good 1 1 1 1 1 1 1 0 1 1 0 1

Pseudomonas aeruginosa strain KPA11 287 WGS KPA11 "JABTVX010000001,JABTVX010000002,JABTVX010000003,JABTVX010000004,JABTVX010000005,JABTVX010000006,JABTVX010000007" 240 6665398 Kenya Homo sapiens Resistant Computational Prediction "crpP,ant(2'')-Ia,aadA1,aph(3')-Iib,aac(6')-Il,aph(3')-VI,ARR-3,sul1,dfrB2,catB7,cmlA1,tet(A),blaPAO,blaOXA-10,blaOXA-50,blaVEB-1,blaNDM-1,qacE" 98.7 Good 1 1 1 1 1 1 1 0 1 1 0 1

Pseudomonas aeruginosa strain KPA10 287 WGS KPA10 "JABTVZ010000001,JABTVZ010000002,JABTVZ010000003,JABTVZ010000004,JABTVZ010000005,JABTVZ010000006,JABTVZ010000007" 275 6657489 Kenya Homo sapiens Resistant Computational Prediction "crpP,ant(2'')-Ia,aadA1,aph(3')-Iib,aac(6')-Il,aph(3')-VI,ARR-3,sul1,dfrB2,catB7,cmlA1,tet(A),blaPAO,blaOXA-10,blaOXA-50,blaVEB-1,blaNDM-1,qacE" 98.5 Good 1 1 1 1 1 1 1 0 1 1 0 1

Pseudomonas aeruginosa strain KPA14 287 WGS KPA14 "JABTWB010000001,JABTWB010000002,JABTWB010000003,JABTWB010000004,JABTWB010000005,JABTWB010000006,JABTWB010000007" 282 6866679 Kenya Homo sapiens Resistant Computational Prediction "catB7,fosA,sul1,blaPAO,blaOXA-396,blaNDM-1,aph(3')-Iib,aph(3'')-Ib,aph(6)-Id" 98.5 Good 1 1 1 1 1 1 1 0 0 0 0 0

Pseudomonas aeruginosa strain KPA7 287 WGS KPA7 "JAFDTA010000001,JAFDTA010000002,JAFDTA010000003,JAFDTA010000004,JAFDTA010000005,JAFDTA010000006,JAFDTA010000007" 82 6900283 Kenya Homo sapiens Resistant Computational Prediction "crpP,aac(6')-Ib-cr,qnrVC1,aadA1,aac(6')-Ib3,aph(3')-Iib,ant(2'')-Ia,sul1,dfrA5,dfrB5,ere(A),catB7,blaPAO,blaOXA-10,blaOXA-395,blaVIM-6,qacE" 98.3 Good 1 1 1 1 1 1 1 1 0 1 0 1

Pseudomonas aeruginosa strain KPA9 287 WGS KPA9 "JABTWA010000001,JABTWA010000002,JABTWA010000003,JABTWA010000004,JABTWA010000005,JABTWA010000006,JABTWA010000007" 281 6657294 Kenya Homo sapiens Resistant Computational Prediction "crpP,ant(2'')-Ia,aadA1,aph(3')-Iib,aac(6')-Il,aph(3')-VI,ARR-3,sul1,dfrB2,catB7,cmlA1,tet(A),blaPAO,blaOXA-10,blaOXA-50,blaVEB-1,blaNDM-1,qacE" 98.5 Good 1 1 1 1 1 1 1 0 1 1 0 1

Pseudomonas aeruginosa strain KPA8 287 WGS KPA8 "JAFDSZ010000001,JAFDSZ010000002,JAFDSZ010000003,JAFDSZ010000004,JAFDSZ010000005,JAFDSZ010000006,JAFDSZ010000007" 85 6900423 Kenya Homo sapiens Resistant Computational Prediction "crpP,aac(6')-Ib-cr,qnrVC1,aadA1,aac(6')-Ib3,aph(3')-Iib,ant(2'')-Ia,sul1,dfrA5,dfrB5,ere(A),catB7,blaPAO,blaOXA-10,blaOXA-395,blaVIM-6,qacE" 98.5 Good 1 1 1 1 1 1 1 1 0 1 0 1

Pseudomonas aeruginosa strain KPA5 287 WGS KPA5 "JABTWL010000001,JABTWL010000002,JABTWL010000003,JABTWL010000004,JABTWL010000005,JABTWL010000006,JABTWL010000007" 196 6653100 Kenya Homo sapiens Resistant Computational Prediction "crpP,ant(2'')-Ia,aadA1,aph(3')-Iib,aac(6')-Il,aph(3')-VI,ARR-3,sul1,dfrB2,catB7,cmlA1,tet(A),blaPAO,blaOXA-10,blaOXA-50,blaVEB-1,blaNDM-1,qacE" 98.7 Good 1 1 1 1 1 1 1 0 1 1 0 1

Pseudomonas aeruginosa strain KPA3 287 WGS KPA3 "JABTWR010000001,JABTWR010000002,JABTWR010000003,JABTWR010000004,JABTWR010000005,JABTWR010000006,JABTWR010000007" 182 6617236 Kenya Homo sapiens Resistant Computational Prediction "crpP,ant(2'')-Ia,aadA1,aph(3')-Iib,aac(6')-Il,aph(3')-VI,ARR-3,sul1,dfrB2,catB7,cmlA1,tet(A),blaPAO,blaOXA-10,blaOXA-50,blaVEB-1,blaNDM-1,qacE" 98.5 Good 1 1 1 1 1 1 1 0 1 1 0 1

Pseudomonas aeruginosa strain KPA4 287 WGS KPA4 "JABTWQ010000001,JABTWQ010000002,JABTWQ010000003,JABTWQ010000004,JABTWQ010000005,JABTWQ010000006,JABTWQ010000007" 188 6619589 Kenya Homo sapiens Resistant Computational Prediction "crpP,ant(2'')-Ia,aadA1,aph(3')-Iib,aac(6')-Il,aph(3')-VI,ARR-3,sul1,dfrB2,catB7,cmlA1,tet(A),blaPAO,blaOXA-10,blaOXA-50,blaVEB-1,blaNDM-1,qacE" 99 Good 1 1 1 1 1 1 1 0 1 1 0 1

Pseudomonas aeruginosa strain KPA1 287 WGS KPA1 "JABTWU010000001,JABTWU010000002,JABTWU010000003,JABTWU010000004,JABTWU010000005,JABTWU010000006,JABTWU010000007" 234 6916168 Homo sapiens Resistant Computational Prediction "crpP,aac(6')-Ib-cr,aph(3'')-Ib,aac(6')-Ib3,aph(6)-Id,aph(3')-Iib,sul1,tet(G),catB7,blaPAO,blaOXA-10,blaOXA-395,blaOXA-1,blaOXA-4,blaVIM-2,qacE,fosA" 97.8 Good 1 1 1 1 1 1 1 0 1 0 0 1

Pseudomonas aeruginosa strain KPA2 287 WGS KPA2 "JABTWW010000001,JABTWW010000002,JABTWW010000003,JABTWW010000004,JABTWW010000005,JABTWW010000006,JABTWW010000007" 216 6658824 Kenya Homo sapiens Resistant Computational Prediction "crpP,ant(2'')-Ia,aadA1,aph(3')-Iib,aac(6')-Il,aph(3')-VI,ARR-3,sul1,dfrB2,catB7,cmlA1,tet(A),blaPAO,blaOXA-10,blaOXA-50,blaVEB-1,blaNDM-1,qacE" 98.5 Good 1 1 1 1 1 1 1 0 1 1 0 1

Pseudomonas aeruginosa strain KPA6 287 WGS KPA6 "JABTXA010000001,JABTXA010000002,JABTXA010000003,JABTXA010000004,JABTXA010000005,JABTXA010000006,JABTXA010000007" 126 6878425 Kenya Homo sapiens Resistant Computational Prediction "catB7,fosA,sul1,blaPAO,blaOXA-396,blaNDM-1,aph(3')-Iib,aph(3'')-Ib,aph(6)-Id" 99 Good 1 1 1 1 1 1 1 0 0 0 0 0

Pseudomonas aeruginosa strain 15965 287 WGS 15965 "JAFKQW010000001,JAFKQW010000010,JAFKQW010000011,JAFKQW010000012,JAFKQW010000013,JAFKQW010000014,JAFKQW010000015" 91 6690617 urine Homo sapiens Resistant Computational Prediction "fosA,qnrVC1,rmtB,aadA10,aph(3')-Iib,sul1,catB7,blaPAO,blaOXA-395,tet(G),qacE" 99.3 Good 1 1 1 1 1 1 1 0 1 1 0 1

Pseudomonas aeruginosa strain 16018 287 WGS 16018 "JAGFEJ010000001,JAGFEJ010000010,JAGFEJ010000100,JAGFEJ010000101,JAGFEJ010000102,JAGFEJ010000103,JAGFEJ010000104" 119 7119809 urine Homo sapiens Resistant Computational Prediction "qnrVC1,aac(6')-Ib-cr,aadA1,aph(3')-Ia,aadA10,aac(3)-Iia,aac(6')-Ib3,aph(3')-Iib,aph(3'')-Ib,aph(6)-Id,tet(A),fosA,sul1,catB7,cmlA1,ARR-2,blaTEM-1B,blaPAO,blaOXA-10,blaOXA-396,blaSCO-1,qacE" 99.1 Good 1 1 1 1 1 1 1 0 1 1 0 1

Pseudomonas aeruginosa strain 15986 287 WGS 15986 "JAGFDP010000001,JAGFDP010000010,JAGFDP010000100,JAGFDP010000101,JAGFDP010000102,JAGFDP010000103,JAGFDP010000104" 127 7046396 wound Homo sapiens Resistant Computational Prediction "qnrVC1,aac(6')-Ib-cr,fosA,tet(A),sul1,aadA10,aadA1,aac(6')-Ib3,aph(6)-Id,aph(3')-Iib,aph(3'')-Ib,ARR-2,cmlA1,catB7,blaPAO,blaOXA-396,blaOXA-10,qacE" 99 Good 1 1 1 1 1 1 1 0 1 0 0 1

Pseudomonas aeruginosa strain Pae3007bg 287 WGS Pae3007bg "JAGFBD010000061,JAGFBD010000046,JAGFBD010000022,JAGFBD010000012,JAGFBD010000019,JAGFBD010000056,JAGFBD010000048" 90 7162522 hospital Bulgaria Homo sapiens Resistant Computational Prediction "crpP,aac(6')-29a,aph(3')-Iib,fosA,sul1,catB7,blaOXA-395,blaVIM-2,blaPAO,qacE" 99 Good 1 1 1 1 1 1 1 0 0 0 0 1

Pseudomonas aeruginosa strain AR_0103 287 WGS AR_0103 MPBP00000000 3 6900175 Intermediate;Resistant;Susceptible AMR Panel "fosA,aac(6')-Ib-cr,aph(3'')-Ib,ant(2'')-Ia,aac(6')-Ib3,aph(3')-Iib,aph(3')-VI,aph(6)-Id,sul1,tet(G),tet(A),catB7,blaIMP-1,blaPAO,blaOXA-396,qacE" 99.3 100 0.9 Good 1 1 1 1 1 1 1 0 1 1 0 1

Pseudomonas aeruginosa strain AR_0092 287 Complete AR_0092 MPBS00000000 1 6963676 Resistant;Susceptible AMR Panel "fosA,aac(6')-Ib-cr,ant(2'')-Ia,aac(3)-Ic,aac(6')-Ib3,aph(3')-VI,aph(3')-Iib,aadA6,tet(A),tet(G),sul1,cmlA1,catB7,blaIMP-14,blaPAO,blaOXA-488,blaIMP-54,blaOXA-10,blaVEB-1,qacE," 99.3 100 Good 1 1 1 1 1 1 1 0 1 1 0 1

Pseudomonas aeruginosa strain AR_0100 287 WGS AR_0100 MPBQ00000000 12 6929574 Resistant;Susceptible;Intermediate AMR Panel "fosA,aac(3)-Id,aac(6')-Il,aph(3')-Iib,aadA6,dfrB5,catB7,blaOXA-488,blaPAO,blaVIM-2,qacE" 99 100 0.6 Good 1 1 1 1 1 1 1 0 0 1 0 0

Pseudomonas aeruginosa strain AR_0105 287 WGS AR_0105 MPBO00000000 6 6425652 Susceptible;Resistant;Intermediate AMR Panel "fosA,aadA6,aph(3')-Iib,aadA1b,sul1,catB7,blaPAO,blaOXA-2,blaOXA-488,qacE" 99.1 99.1 1.5 Good 1 1 1 1 1 1 1 0 0 1 0 0

Pseudomonas aeruginosa strain AR_0054 287 WGS AR_0054 MPBV00000000 9 7267705 Susceptible;Intermediate;Resistant AMR Panel "fosA,blaOXA-2,aph(3')-Iib,aph(3'')-Ib,ant(2'')-Ia,sul1,tet(A),catB7,blaVIM-4,blaOXA-396,blaPAO," 98.7 100 0.6 Good 1 1 1 1 1 1 1 0 1 1 0 0

Pseudomonas aeruginosa strain AR_0064 287 WGS AR_0064 MPBU00000000 16 6827144 Susceptible;Intermediate;Resistant AMR Panel "crpP,aac(6')-Ib-cr,fosA,aac(6')-Ib3,aph(3')-Iib,sul1,aadA7,cmx,catB7,blaSPM-1,blaPAO,blaOXA-396,blaOXA-494,blaOXA-56,qacE" 98.5 100 1.2 Good 1 1 1 1 1 1 1 0 0 0 0 1

Pseudomonas aeruginosa strain AR_0094 287 WGS AR_0094 MPBR00000000 5 6882581 Intermediate;Resistant;Susceptible AMR Panel "crpP,fosA,catB7,blaPAO,blaOXA-396,aph(3')-Iib" 98.3 100 0.3 Good 1 1 1 1 1 1 1 0 0 1 0 1

Pseudomonas aeruginosa strain AR_0108 287 WGS AR_0108 MPBN00000000 3 6971125 Resistant;Susceptible AMR Panel "fosA,aac(3)-Id,aac(6')-Il,aph(3')-Iib,aadA2,sul1,cmlA1,dfrB5,catB7,blaOXA-486,blaPAO,blaVIM-2,qacE,tet(G),blaOXA-4" 98.8 100 Good 1 1 1 1 1 1 1 0 1 1 0 1

Pseudomonas aeruginosa strain SCH_ABX16 287 WGS SCH_ABX16 MULB00000000 51 6312238 Airway secretion United States "Human, Homo sapiens" Resistant Computational Prediction "fosA,catB7,blaPAO,blaOXA-396,blaOXA-494,aph(3')-Iib" 99.6 100 Good 1 1 1 1 1 1 1 0 0 0 0 0

Pseudomonas aeruginosa strain SCH_ABX12 287 WGS SCH_ABX12 MULF00000000 79 6777246 Airway secretion United States "Human, Homo sapiens" Resistant Computational Prediction "crpP,fosA,catB7,blaPAO,blaOXA-396,blaOXA-494,aph(3')-Iib" 98.8 100 Good 1 1 1 1 1 1 1 0 0 0 0 1

Pseudomonas aeruginosa strain Kasamber 287 WGS Kasamber MVDK00000000 240 6764168 Urine Sudan "Human, Homo sapiens" Resistant Computational Prediction "fosA,aph(3')-VI,aadA6,aph(3')-Iib,tet(A),tet(G),blaVEB-1,catB7,blaOXA-488,blaPAO,qacE" 97.8 100 1.8 Good 1 1 0 1 1 1 1 0 1 1 0 0

Pseudomonas aeruginosa strain SCH_ABX08 287 WGS SCH_ABX08 MVBQ00000000 73 6776755 Airway secretion United States "Human, Homo sapiens" Resistant Computational Prediction "crpP,fosA,catB7,blaPAO,blaOXA-396,blaOXA-494,aph(3')-Iib" 98.8 100 Good 1 1 1 1 1 1 1 0 0 0 0 1

Pseudomonas aeruginosa strain COPD2d 287 WGS COPD2d MWLA00000000 228 6191379 Palma de Mallorca Spain "Human, Homo sapiens" Resistant Computational Prediction "fosA,catB7,blaPAO,blaOXA-396,blaOXA-494,aph(3')-Iib" 97.4 98.8 0.9 Good 1 1 1 1 1 1 1 0 0 0 0 0

Pseudomonas aeruginosa strain DZ-B1 287 WGS DZ-B1 NBVZ00000000 102 7202108 feces swab China swallow Resistant Computational Prediction "fosA,aac(6')-Ib-cr,aph(3')-Iib,aac(6')-Ib3,aac(6')-Iia,aph(3'')-Ib,aph(3')-Iia,aph(6)-Id,tet(G),dfrB1,sul1,catB7,blaPAO,blaOXA-396,blaVIM-2" 97.9 100 0.6 Good 1 1 1 1 1 1 1 0 1 1 0 1

Pseudomonas aeruginosa strain PASGNDM571 287 WGS PASGNDM571 NDFR00000000 89 6835318 urine Singapore "Human, Homo sapiens" Resistant Computational Prediction "crpP,qnrVC1,aph(3'')-Ib,aac(3)-Id,aph(3')-Iib,aac(6')-Il,aadA10,aph(6)-Id,fosA,dfrB5,sul2,floR,catB7,blaNDM-1,blaOXA-488,blaPAO,msr€" 98.7 100 Good 1 1 1 1 1 1 1 1 0 1 0 1

Pseudomonas aeruginosa strain PASGNDM544 287 WGS PASGNDM544 NDFQ00000000 111 6835576 endotracheal tube (ETT) aspirate Singapore "Human, Homo sapiens" Resistant Computational Prediction "crpP,qnrVC1,aph(3'')-Ib,aac(3)-Id,aph(3')-Iib,aac(6')-Il,aadA10,aph(6)-Id,fosA,dfrB5,sul2,floR,catB7,blaNDM-1,blaOXA-488,blaPAO,msr€" 98.6 100 Good 1 1 1 1 1 1 1 1 0 1 0 1

Pseudomonas aeruginosa strain PASGNDM587 287 WGS PASGNDM587 NDFV00000000 108 6786631 foot wound swab Singapore "Human, Homo sapiens" Resistant Computational Prediction "crpP,qnrVC1,aph(3'')-Ib,aac(3)-Id,aph(3')-Iib,aac(6')-Il,aadA10,aph(6)-Id,fosA,dfrB5,sul2,floR,catB7,blaNDM-1,blaOXA-488,blaPAO,msr€" 98.4 99.7 0.3 Good 1 1 1 1 1 1 1 1 0 1 0 1

Pseudomonas aeruginosa strain PASGNDM592 287 WGS PASGNDM592 NDFX00000000 87 6826648 urine Singapore "Human, Homo sapiens" Resistant Computational Prediction "crpP,qnrVC1,aph(3'')-Ib,aac(3)-Id,aph(3')-Iib,aac(6')-Il,aadA10,aph(6)-Id,fosA,dfrB5,sul2,floR,catB7,blaNDM-1,blaOXA-488,blaPAO,msr€" 98.8 100 0.1 Good 1 1 1 1 1 1 1 1 0 1 0 1

Pseudomonas aeruginosa strain PASGNDM586 287 WGS PASGNDM586 NDFT00000000 101 6765961 urine Singapore "Human, Homo sapiens" Resistant Computational Prediction "crpP,qnrVC1,aph(3'')-Ib,aac(3)-Id,aph(3')-Iib,aac(6')-Il,aadA10,aph(6)-Id,fosA,dfrB5,sul2,floR,catB7,blaNDM-1,blaOXA-488,blaPAO,msr€" 98.3 99.4 0.5 Good 1 1 1 1 1 1 1 1 0 1 0 1

Pseudomonas aeruginosa strain PASGNDM590 287 WGS PASGNDM590 NDFU00000000 99 6827528 urine Singapore "Human, Homo sapiens" Resistant Computational Prediction "crpP,qnrVC1,aph(3'')-Ib,aac(3)-Id,aph(3')-Iib,aac(6')-Il,aadA10,aph(6)-Id,fosA,dfrB5,sul2,floR,catB7,blaNDM-1,blaOXA-488,blaPAO,msr€" 98.7 100 0.6 Good 1 1 1 1 1 1 1 1 0 1 0 1

Pseudomonas aeruginosa strain S769_C16_RS 287 WGS S769_C16_RS NFFM00000000 104 6992746 Respiratory Sample Italy "Human, Homo sapiens" Resistant Computational Prediction "crpP,aac(6')-Ib-cr,aph(3')-Iib,aac(6')-Ib3,fosA,sul1,catB7blaIMP-13,blaPAO,blaOXA-50,qacE" 99.2 100 Good 1 1 1 1 1 1 1 0 0 0 0 1

Pseudomonas aeruginosa strain S749_C15_RS 287 WGS S749_C15_RS NFFN00000000 169 7115643 Respiratory Sample Italy "Human, Homo sapiens" Resistant Computational Prediction "crpP,aph(3')-Iib,aac(6')-29a,aac(6')-29b,fosA,sul1,catB7,blaPAO,blaOXA-395,blaVIM-2,qacE" 99 100 Good 1 1 1 1 1 1 1 0 0 0 0 1

Pseudomonas aeruginosa strain S742_C15_BS 287 WGS S742_C15_BS NFFO00000000 125 7181459 blood Italy "Human, Homo sapiens" Resistant Computational Prediction "crpP,aac(6')-Ib-cr,aph(3')-Iib,Gar,aac(6')-Ib3,aadA13,sul1,blaVIM-1,blaOXA-2,blaOXA-395,blaPAO,qacE" 98.9 99.4 Good 1 1 1 1 1 1 1 0 0 1 0 1

Pseudomonas aeruginosa strain S668_C14_BS 287 WGS S668_C14_BS NFFS00000000 127 7103130 blood Italy "Human, Homo sapiens" Resistant Computational Prediction "crpP,aac(6')-Ib-cr,aph(3')-Iib,aac(6')-Ib3,fosA,sul1,catB7blaIMP-13,blaPAO,blaOXA-50,qacE" 98.7 100 0.3 Good 1 1 1 1 1 1 1 0 0 0 0 1

Pseudomonas aeruginosa strain S626_C13_RS 287 WGS S626_C13_RS NFFV00000000 135 6993511 Respiratory Sample Italy "Human, Homo sapiens" Resistant Computational Prediction "crpP,aac(6')-Ib-cr,aph(3')-XV,aph(3')-Iib,aac(6')-Ib3,aadA1,sul1,blaVIM-1,blaOXA-396,blaPAO,qacE" 98.7 100 0.4 Good 1 1 1 0 1 1 1 0 0 0 0 1

Pseudomonas aeruginosa strain S611_C13_RS 287 WGS S611_C13_RS NFFY00000000 110 7146674 Respiratory Sample Italy "Human, Homo sapiens" Resistant Computational Prediction "crpP,aac(6')-Ib-cr,aph(3')-XV,aph(3')-Iib,aac(6')-Ib3,aadA1,sul1,blaVIM-1,blaOXA-9,blaOXA-395,blaPAO,qacE" 99 100 0.1 Good 1 1 1 0 1 1 1 0 0 0 0 1

Pseudomonas aeruginosa strain S442_C09_BS 287 WGS S442_C09_BS NFGE00000000 104 6992620 blood Italy "Human, Homo sapiens" Resistant Computational Prediction "crpP,fosA,aph(3')-Iib,aac(6')-Il,ant(2'')-Ia,sul1,ARR-4,catB7,blaPAO,blaOXA-50,blaVIM-2,qacE" 99 99.7 Good 1 1 1 1 1 1 1 0 0 1 0 1

Pseudomonas aeruginosa strain S435_C09_BS 287 WGS S435_C09_BS NFGG00000000 106 6992710 blood Italy "Human, Homo sapiens" Resistant Computational Prediction "crpP,fosA,aph(3')-Iib,aac(6')-Il,ant(2'')-Ia,sul1,ARR-4,catB7,blaPAO,blaOXA-50,blaVIM-2,qacE" 99 99.7 Good 1 1 1 1 1 1 1 0 0 1 0 1

Pseudomonas aeruginosa strain S426_C09_BS 287 WGS S426_C09_BS NFGJ00000000 139 7029308 blood Italy "Human, Homo sapiens" Resistant Computational Prediction "crpP,fosA,aph(3')-Iib,aac(6')-Il,ant(2'')-Ia,sul1,ARR-4,catB7,blaPAO,blaOXA-50,blaVIM-2,qacE" 99 99.7 Good 1 1 1 1 1 1 1 0 0 1 0 1

Pseudomonas aeruginosa strain S422_C09_BS 287 WGS S422_C09_BS NFGK00000000 124 7002804 blood Italy "Human, Homo sapiens" Resistant Computational Prediction "crpP,fosA,aph(3')-Iib,aac(6')-Il,ant(2'')-Ia,sul1,ARR-4,catB7,blaPAO,blaOXA-50,blaVIM-2,qacE" 99 99.7 Good 1 1 1 1 1 1 1 0 0 1 0 1

Pseudomonas aeruginosa strain S292_C06_RS 287 WGS S292_C06_RS NFGM00000000 89 6895685 Respiratory Sample Italy "Human, Homo sapiens" Resistant Computational Prediction "crpP,aac(6')-Ib-cr,fosA,aph(3')-Iib,aadA1,ant(2'')-Iaaac(6')-Ib3,blaVIM-1,blaoXA-50,blaPAO,qacE" 99 99.7 Good 1 1 1 1 1 1 1 0 0 1 0 1

Pseudomonas aeruginosa strain S252_C06_RS 287 WGS S252_C06_RS NFGN00000000 152 6918214 Respiratory Sample Italy "Human, Homo sapiens" Resistant Computational Prediction "crpP,aac(6')-Ib-cr,fosA,aph(3')-Iib,aadA1,ant(2'')-Iaaac(6')-Ib3,blaVIM-1,blaoXA-50,blaPAO,qacE" 99 99.7 0.3 Good 1 1 1 1 1 1 1 0 0 1 0 1

Pseudomonas aeruginosa strain S61_C01_BS 287 WGS S61_C01_BS NFGT00000000 116 7054993 blood Italy "Human, Homo sapiens" Resistant Computational Prediction "crpP,aph(3')-Iib,aac(6')-29b,fosA,sul1,catB7,blaPAO,blaOXA-395,blaVIM-2,qacE" 99 100 Good 1 1 1 1 1 1 1 0 0 0 0 1

Pseudomonas aeruginosa strain S53_C01_BS 287 WGS S53_C01_BS NFGV00000000 160 6919115 blood Italy "Human, Homo sapiens" Resistant Computational Prediction "crpP,aac(6')-Ib-cr,aac(6')-Ib3,aph(3')-Iib,aaDA1,fosA,sul1,catB7,blaVIM-1,blaPAO,blaOXA-395,qacE" 98.7 99.7 0.4 Good 1 1 1 1 1 1 1 0 0 0 0 1

Pseudomonas aeruginosa strain S854_C18_BS 287 WGS S854_C18_BS NFFC00000000 162 6851665 blood Italy "Human, Homo sapiens" Resistant Computational Prediction "aadA1,aac(6')-Ib-cr,aph(3')-XV,aac(3)-Ic,aac(6')-Ib3,aph(3')-Iib,fosA,sul1,catB7,catB10,cmlA1,blaVIM-1,blaOXA-488,blaPAO,qacE" 98.4 100 0.9 Good 1 1 1 1 1 1 1 0 0 1 0 1

Pseudomonas aeruginosa strain S787_C16_RS 287 WGS S787_C16_RS NFFK00000000 103 6976024 Respiratory Sample Italy "Human, Homo sapiens" Resistant Computational Prediction "crpP,aac(6')-Ib-cr,fosA,sul1,aph(3')-Iib,aac(6')-Ib3,catB7,blaOXA-50,blaPAO,blaIMP-13,qacE" 99.2 100 Good 1 1 1 1 1 1 1 0 0 0 0 1

Pseudomonas aeruginosa strain S708_C14_RS 287 WGS S708_C14_RS NFFP00000000 215 7301745 Respiratory Sample Italy "Human, Homo sapiens" Resistant Computational Prediction "crpP,aph(3')-Iib,aac(6')-29a,aac(6')-31,aac(6')-29b,fosA,sul1,catB7,blaOXA-395,blaOXA-15,blaVIM-2,blaPAO,qacE" 98.2 100 0.3 Good 1 1 1 1 1 1 1 0 0 1 0 1

Pseudomonas aeruginosa strain S440_C09_BS 287 WGS S440_C09_BS NFGF00000000 99 6992596 blood Italy "Human, Homo sapiens" Resistant Computational Prediction "crpP,fosA,aph(3')-Iib,ant(2'')-Ia,aac(6')-Il,sul1,ARR-4,catB7,blaPAO,blaOXA-50,blaVIM-2,qacE" 98.9 99.7 Good 1 1 1 1 1 1 1 0 0 1 0 1

Pseudomonas aeruginosa strain S220_C06_RS 287 WGS S220_C06_RS NFGP00000000 155 7152951 Respiratory Sample Italy "Human, Homo sapiens" Resistant Computational Prediction "crpP,aac(6')-Ib-cr,fosA,aac(6')-Ib3,aph(3')-Iib,sul1,catB7,blaOXA-50,blaIMP-13,qacE" 99 100 Good 1 1 1 0 1 1 1 0 0 0 0 0

Pseudomonas aeruginosa strain ICB10P 287 WGS ICB10P MWKP00000000 364 6436450 footpad infection Brazil "Magellanic penguin, Spheniscus magellanicus" Resistant Computational Prediction "crpP,aac(6')-Ib-cr,aadA2b,aph(3')-Iib,fosA,aac(6')-Ib3,sul1,cmlA1,catB7,tet(G),blaPAO,blaOXA-486,qacE" 95.6 99.7 4.6 Good 1 1 0 1 1 1 1 0 0 0 0 0

Pseudomonas aeruginosa strain FFUP_PS_CB5 287 WGS FFUP_PS_CB5 NINP00000000 87 6842831 Bronchial aspirate Portugal "Human, Homo sapiens" Resistant Computational Prediction "fosA,aadA6,aph(3')-Iib,cmx,sul1,catB7,blaPAO,blaVIM-2,blaOXA-488,qacE" 98.4 100 0.6 Good 1 1 1 1 1 1 1 0 0 1 0 0

Pseudomonas aeruginosa strain FFUP_PS_144 287 WGS FFUP_PS_144 NINR00000000 202 7275099 urine Portugal "Human, Homo sapiens" Resistant Computational Prediction "crpP,aac(6')-Ib-cr,aac(6')-Ib3,aph(3')-Iib,aac(3)-I,aac(6')-Il,fosA,sul1,catB7,blaPAO,blaOXA-396,blaVIM-2,qacE" 98.4 100 2.1 Good 1 1 1 1 1 1 1 0 0 1 0 1

Pseudomonas aeruginosa strain FFUP_PS_12 287 WGS FFUP_PS_12 NINQ00000000 540 6998934 Portugal "Human, Homo sapiens" Resistant Computational Prediction "crpP,aac(6')-Ib-cr,fosA,catB7,blaPAO,blaOXA-50,aac(6')-Ib3,aac(3)-I,aph(3')-Iib,aac(6')-Il,sul1,blaVIM-2,qacE," 94.1 99.7 4.7 Good 1 1 1 1 1 1 1 0 0 1 0 1

Pseudomonas aeruginosa strain FFUP_PS_105 287 WGS FFUP_PS_105 NINS00000000 136 7020553 urine Portugal "Human, Homo sapiens" Resistant Computational Prediction "crpP,aac(6')-Ib-cr,fosA,aadA2b,aac(6')-Ib3,aph(3')-Iib,blaCARB-2,blaOXA-395,blaPAO,blaVIM-2,qacE" 98.2 100 0.3 Good 1 1 1 1 1 1 1 0 0 0 0 1

Pseudomonas aeruginosa strain FFUP_PS_35 287 WGS FFUP_PS_35 NINU00000000 158 7002274 urine Portugal "Human, Homo sapiens" Resistant Computational Prediction "crpP,aac(6')-Ib-cr,fosA,aph(3')-Iib,aac(6')-Ib3,aac(6')-Il,sul1,catB7,blaPAO,blaOXA-396,v,qacE" 98.3 100 0.9 Good 1 1 1 1 1 1 1 0 0 0 0 0

Pseudomonas aeruginosa strain FFUP_PS_37 287 WGS FFUP_PS_37 NINT00000000 436 7504985 Bronchial secretions Portugal "Human, Homo sapiens" Resistant Computational Prediction "crpP,aac(6')-Ib-cr,aac(6')-Il,aph(3')-Iib,aac(3)-I,aac(6')-Ib3,fosA,sul1,catB7,blaPAO,blaoXA-488,blaVIM-2,qacE" 95.2 100 1.5 Good 1 1 1 1 1 1 1 0 0 1 0 1

Pseudomonas aeruginosa strain FFUP_PS_65 287 WGS FFUP_PS_65 NINN00000000 134 7192338 urine Portugal "Human, Homo sapiens" Resistant Computational Prediction "crpP,aac(6')-Ib-cr,aac(6')-Ib3,aac(6')-Il,aac(3)-I,aph(3')-Iib,fosA,sul1,catB7,blaPAO,blaOXA-494,blaOXA-396,blaVIM-2,qacE" 98.3 100 0.7 Good 1 1 1 1 1 1 1 0 0 1 0 1

Pseudomonas aeruginosa strain FFUP_PS_690 287 WGS FFUP_PS_690 NBEZ00000000 110 6782187 urine Portugal "Human, Homo sapiens" Resistant Computational Prediction "fosA,aadA11,aph(3')-Iib,aac(6')-Il,ant(2'')-Ia,sul1,dfrB1,catB7,blaPAO,blaOXA-488,blaGES-7,blaGES-5,blaGES-6,blaGES-13,qacE" 98.8 100 Good 1 1 0 1 1 1 1 0 0 1 0 0

Pseudomonas aeruginosa strain 124 287 Complete CP021774 1 7008516 bronchial washing Mexico "Human, Homo sapiens" Resistant Computational Prediction "fosA,aac(6')-33,aph(3')-Iib,sul1,tet(G),catB7,blaOXA-50,blaPAO,blaGES-19,blaGES-20,qacE" 99 100 0.3 Good 1 0 1 1 1 1 1 0 1 1 0 0

Pseudomonas aeruginosa strain 58 287 Complete CP021775 1 7241575 bronchial washing Mexico "Human, Homo sapiens" Resistant Computational Prediction "crpP,fosA,catB7,blaPAO,blaOXA-396,aph(3')-Iib" 97.5 99.4 0.9 Good 1 1 1 1 1 1 1 0 0 0 0 1

Pseudomonas aeruginosa strain 2330 287 WGS 2330 NIOQ00000000 81 6955793 eye drainage United States "Human, Homo sapiens" Resistant AMR Panel "fosA,catB7,blaPAO,blaOXA-50,aph(3')-Iib" 98.9 100 0.6 Good 1 1 0 1 1 1 1 0 0 0 0 0

Pseudomonas aeruginosa strain 2253 287 WGS 2253 NIOL00000000 125 6867867 wound United States "Human, Homo sapiens" Resistant AMR Panel "fosA,catB7,blaPAO,blaOXA-50,aph(3')-Iib" 99.2 100 Good 1 1 0 1 1 1 1 0 0 0 0 0

Pseudomonas aeruginosa strain 2570 287 WGS 2570 NIOT00000000 101 6831325 blood United States "Human, Homo sapiens" Resistant AMR Panel "fosA,catB7,blaPAO,blaOXA-50,aph(3')-Iib" 99.2 100 Good 1 1 0 1 1 1 1 0 0 0 0 0

Pseudomonas aeruginosa strain 2623 287 WGS 2623 NIOU00000000 178 7007284 blood United States "Human, Homo sapiens" Resistant AMR Panel "crpP,fosA,aadA1b,aph(3')-Iib,ant(2'')-Ia,aph(3')-Ib,sul1,catB7,blaOXA-2,blaPAO,blaOXA-396,qacE" 98.7 100 0.7 Good 1 1 1 1 1 1 1 0 0 1 0 1

Pseudomonas aeruginosa strain 797 287 WGS 797 NIPD00000000 163 7429754 infection control United States Resistant AMR Panel "crpP,aph(3')-Iib,aac(3)-IIIb,fosA,sul1,catB7,blaPAO,blaOXA-2,blaOXA-50,qacE" 98.2 100 2.1 Good 1 1 0 1 1 1 1 0 0 1 0 1

Pseudomonas aeruginosa strain 829 287 WGS 829 NIPE00000000 99 6832393 infection control United States Resistant AMR Panel "crpP,fosA,catB7,blaPAO,blaOXA-396,blaOXA-494,aph(3')-Iib" 98.3 100 Good 1 1 1 1 1 1 1 0 0 0 0 1

Pseudomonas aeruginosa strain 6003 287 WGS 6003 NIOW00000000 123 6910590 blood United States "Human, Homo sapiens" Resistant AMR Panel "crpP,fosA,catB7,blaPAO,blaOXA-396,blaOXA-494,aph(3')-Iib" 98.1 100 Good 1 1 1 1 1 1 1 0 0 0 0 1

Pseudomonas aeruginosa strain 833 287 WGS 833 NIPF00000000 128 7224440 infection control United States Resistant AMR Panel "crpP,aph(6)-Id,aph(3'')-Ib,aadA10,aph(3')-Iib,fosA,sul1,catB7,blaPAO,blaOXA-396,qacE" 98.7 100 Good 1 1 1 1 1 1 1 0 0 1 0 1

Pseudomonas aeruginosa strain 2671 287 WGS 2671 NIOV00000000 109 6877412 blood United States "Human, Homo sapiens" Resistant AMR Panel "crpP,fosA,catB7,blaPAO,blaOXA-396,blaOXA-494,aph(3')-Iib" 98.8 100 Good 1 1 1 1 1 1 1 0 0 0 0 1

Pseudomonas aeruginosa strain 2357 287 WGS 2357 NIOS00000000 93 6727243 nasopharynx United States "Human, Homo sapiens" Resistant AMR Panel "fosA,aadA6,aph(3')-Iib,catB7,blaPAO,blaOXA-488,qacE" 99 100 Good 1 1 0 1 1 1 1 0 0 1 0 0

Pseudomonas aeruginosa strain 759 287 WGS 759 NIPC00000000 184 6987420 infection control United States Resistant AMR Panel "crpP,fosA,catB7,blaPAO,blaOXA-50,aph(3')-Iib" 99 100 Good 1 1 0 1 1 1 1 0 0 0 0 1

Pseudomonas aeruginosa strain 836 287 WGS 836 NIPG00000000 80 6427172 infection control United States Resistant AMR Panel "crpP,fosA,catB7,blaPAO,blaOXA-488,aph(3')-Iib" 98.5 98.8 0.9 Good 1 1 0 1 1 1 1 0 0 0 0 1

Pseudomonas aeruginosa strain PAS5 287 WGS PAS5 NIJA00000000 230 6954778 wound Malaysia "Human, Homo sapiens" Resistant Computational Prediction "fosA,aac(6')-Ib-cr,aph(3')-Iib,aac(6')-Ib3,aph(3')-Iib,aadA6,aph(3')-XV,tet(G),catB7,blaPAO,blaGES-1,blaoXA-488qacE" 98.9 100 0.3 Good 1 1 0 1 1 1 1 0 1 1 0 1

Pseudomonas aeruginosa strain PAS6 287 WGS PAS6 NIIZ00000000 189 7036089 urine Malaysia "Human, Homo sapiens" Resistant Computational Prediction "fosA,aac(6')-Ib-cr,tet(G),aph(3')-Iib,aac(6')-Ib3,aph(3')-XV,aadA6,catB7,blaGES-1,blaPAO,blaOXA-488,qacE" 97.9 100 0.9 Good 1 1 1 1 1 1 1 0 1 1 0 1

Pseudomonas aeruginosa strain PAS2 287 WGS PAS2 NIJD00000000 243 6627283 wound Malaysia "Human, Homo sapiens" Resistant Computational Prediction "fosA,aac(6')-Ib-cr,aph(3')-Iib,aac(6')-Ib-Hangzhou,sul1,catB7,blaOXA-395,blaOXA-10,blaGES-13,blaPAO,qacE" 98.6 100 Good 1 1 1 1 1 1 1 0 0 0 0 1

Pseudomonas aeruginosa strain PAS3 287 WGS PAS3 NIJC00000000 322 6731789 urine Malaysia "Human, Homo sapiens" Resistant Computational Prediction "fosA,aac(6')-Ib-cr,aph(3')-Iib,aac(6')-Ib-Hangzhou,sul1,catB7,blaOXA-395,blaOXA-10,blaGES-13,blaPAO,qacE" 98.1 100 0.3 Good 1 1 1 1 1 1 1 0 0 0 0 1

Pseudomonas aeruginosa strain PAS10 287 WGS PAS10 NIIV00000000 239 6955811 blood Malaysia "Human, Homo sapiens" Resistant Computational Prediction "aadA6,aac(6')-Ib-cr,aph(3')-Iib,aph(3')-XV,aac(6')-Ib3,tet(G),catB7,blaPAO,blaOXA-488,blaGES-1,fosA,qacE" 98.7 100 0.3 Good 1 1 0 1 1 1 1 0 1 1 0 1

Pseudomonas aeruginosa strain PAS9 287 WGS PAS9 NIIW00000000 279 7050477 wound Malaysia "Human, Homo sapiens" Resistant Computational Prediction "aadA6,aac(6')-Ib-cr,aph(3')-Iib,aph(3')-XV,aac(6')-Ib3,tet(G),catB7,blaPAO,blaOXA-488,blaGES-1,fosA,qacE" 98.1 100 0.9 Good 1 1 0 1 1 1 1 0 1 1 0 1

Pseudomonas aeruginosa strain PAS8 287 WGS PAS8 NIIX00000000 293 6762669 blood Malaysia "Human, Homo sapiens" Resistant Computational Prediction "fosA,aac(6')-Ib-cr,aph(3')-Iib,aac(6')-Ib-Hangzhou,sul1,catB7,blaOXA-395,blaOXA-10,blaGES-13,blaPAO,qacE" 98.2 100 0.6 Good 1 1 1 1 1 1 1 0 0 0 0 1

Pseudomonas aeruginosa strain PAS7 287 WGS PAS7 NIIY00000000 492 6851779 urine Malaysia "Human, Homo sapiens" Resistant Computational Prediction "crpP,aac(6')-Ib-cr,fosA,aph(3')-Iib,aac(6')-Ib3,dfrA1,aadA6,sul1,catB7,catB3,blaOXA-488,blaPAO,blaIMP-26,qacE" 98.4 100 Good 1 1 1 1 1 1 1 0 0 1 0 1

Pseudomonas aeruginosa strain PAS4 287 WGS PAS4 NIJB00000000 185 6753415 urine Malaysia "Human, Homo sapiens" Resistant Computational Prediction "crpP,aac(6')-Ib-cr,fosA,aph(3')-Iib,aac(6')-Ib3,dfrA1,aadA6,sul1,catB7,catB3,blaOXA-488,blaPAO,blaIMP-26,qacE" 98.6 100 Good 1 1 1 1 1 1 1 0 0 1 0 1

Pseudomonas aeruginosa strain PAS1 287 WGS PAS1 NIJE00000000 325 6909968 wound Malaysia "Human, Homo sapiens" Resistant Computational Prediction "crpP,aac(6')-Ib-cr,fosA,aph(3')-Iib,aac(6')-Ib3,dfrA1,aadA6,sul1,catB7,catB3,blaOXA-488,blaPAO,blaIMP-26,qacE" 98.4 100 0.3 Good 1 1 1 1 1 1 1 0 0 1 0 1

Pseudomonas aeruginosa strain NA04 287 WGS NA04 MYFK00000000 52 6373592 Soil Mexico Nematode Resistant Computational Prediction "fosA,catB7,blaPAO,blaOXA-488,blaOXA-396,aph(3')-Iib" 99.3 100 Good 1 1 1 1 1 1 1 0 0 0 0 0

Pseudomonas aeruginosa strain 127 287 Complete CP022000 1 7148302 bronchial washing Mexico "Human, Homo sapiens" Resistant Computational Prediction "aadA1b,aac(6')-33,aph(3')-Iib,fosA,sul1,catB7,tet(G),blaPAO,blaOXA-50,blaGES-20,blaGES-19,qacE" 98.2 100 3.2 Good 1 1 0 1 1 1 1 0 1 1 0 0

Pseudomonas aeruginosa strain 1242 287 Complete CP022002 1 7050510 Blood Mexico "Human, Homo sapiens" Resistant Computational Prediction "crpP,fosA,catB7,blaPAO,blaOXA-488,blaOXA-396,aph(3')-Iib" 97.2 99.4 2.2 Good 1 1 1 1 1 1 1 0 0 0 0 1

Pseudomonas aeruginosa strain PA77 287 WGS PA77 MJMC00000000 4 7025617 blood Germany "Human, Homo sapiens" Resistant Computational Prediction "crpP,aac(6')-Ib-cr,fosA,aph(3')-Iib,aac(6')-Ib3,aph(3')-XV,aadA10,aac(6')-Ib3,blaOXA-10,sul1,blaOXA-488,blaIMP-8,blaOXA-2,qacE" 98.7 100 1.5 Good 1 1 1 1 1 1 1 0 0 1 0 1

Pseudomonas aeruginosa strain Env_12 287 WGS Env_12 NLBE00000000 100 6327326 Green pepper Canada Resistant Computational Prediction "fosA,catB7,blaPAO,blaOXA-488,aph(3')-Iib" 99.9 100 Good 1 1 0 1 1 1 1 0 0 0 0 0

Pseudomonas aeruginosa strain BK3 287 WGS BK3 LFDE00000000 114 7194702 cornea of keratitis patient India "Human, Homo sapiens" Resistant Computational Prediction "crpP,fosA,aph(3')-Iib,aph(6)-Id,aph(3'')-Ib,sul1,tet(G),catB7,blaOXA-488,blaPAO" 98.6 99.7 0.3 Good 1 1 0 1 1 1 1 0 1 1 0 1

Pseudomonas aeruginosa strain BK6 287 WGS BK6 LFDH00000000 142 7056854 cornea from keratitis patient India "Human, Homo sapiens" Resistant Computational Prediction "qnrVC1,fosA,crpP,aph(6)-Id,aph(3')-Iib,aph(3'')-Ib,rmtB,aac(6')-Il,aadA1,aac(3)-Id,sul1,dfrB5,tet(G),catB7,blaOXA-50,blaVIM-2,ere(A),qacE" 98.7 100 0.6 Good 1 1 1 1 1 1 1 1 1 1 0 1

Pseudomonas aeruginosa strain BK2 287 WGS BK2 LEOT00000000 50 6386147 Cornea India "Human, Homo sapiens" Resistant Computational Prediction "fosA,catB7,blaPAO,blaOXA-488,aph(3')-Iib" 99.4 100 Good 1 1 0 1 1 1 1 0 0 0 0 0

Pseudomonas aeruginosa strain 53012 287 WGS 53012 NMPU00000000 588 7042955 tracheal aspiration Belarus "Human, Homo sapiens" Resistant Computational Prediction "crpP,aph(3')-Iib,fosA,aac(6')-Il,sul1,cmlA1,catB7,blaOXA-198,blaOXA-395,blaPAO,qacE" 95.9 100 2.3 Good 1 1 1 1 1 1 1 0 0 1 0 1

Pseudomonas aeruginosa strain 53014 287 WGS 53014 NMPT00000000 383 7050840 tracheal aspiration Belarus "Human, Homo sapiens" Resistant Computational Prediction "crpP,aph(3')-Iib,fosA,aac(6')-Il,sul1,cmlA1,catB7,blaOXA-198,blaOXA-395,blaPAO,qacE" 97.4 100 1 Good 1 1 1 1 1 1 1 0 0 1 0 1

Pseudomonas aeruginosa strain 53011 287 WGS 53011 NMPV00000000 934 7005891 tracheal aspiration Belarus "Human, Homo sapiens" Resistant Computational Prediction "crpP,aph(3')-Iib,fosA,aac(6')-Il,sul1,cmlA1,catB7,blaOXA-198,blaOXA-395,blaPAO,qacE" 93.7 100 6.1 Good 1 1 1 1 1 1 1 0 0 1 0 1

Pseudomonas aeruginosa strain 00041437 287 WGS 41437 NMPW00000000 586 7100137 tracheal aspiration Belarus "Human, Homo sapiens" Resistant Computational Prediction "crpP,aph(3')-Iib,fosA,aac(6')-Il,sul1,cmlA1,catB7,blaOXA-198,blaOXA-395,blaPAO,qacE" 96.3 100 3.3 Good 1 1 1 1 1 1 1 0 0 1 0 1

Pseudomonas aeruginosa strain PAN3 287 WGS PAN3 NMQQ00000000 71 6698111 hospital effluent Brazil Resistant Computational Prediction "crpP,aac(6')-Ib-cr,fosA,rmtD,aph(3')-Iib,aac(6')-Ib3,aadA7,cmx,catB7,sul1,blaOXA-396,blaPAO,blaOXA-494,blaOXA-56,blaSPM-1,qacE" 99 100 0.3 Good 1 1 1 1 1 1 1 0 0 1 0 1

Pseudomonas aeruginosa strain Ocean-1170 287 WGS Ocean-1170 NMRU00000000 3 7008714 Open Ocean Resistant Computational Prediction "crpP,fosA,catB7,blaPAO,blaOXA-395,aph(3')-Iib" 98.9 100 0.6 Good 1 1 1 1 1 1 1 0 0 0 0 1

Pseudomonas aeruginosa strain Ocean-1187 287 WGS Ocean-1187 NMRQ00000000 90 7067962 Open Ocean Resistant Computational Prediction "crpP,fosA,catB7,blaPAO,blaOXA-395,aph(3')-Iib" 99 100 0.3 Good 1 1 1 1 1 1 1 0 0 0 0 1

Pseudomonas aeruginosa strain Ocean-1206 287 WGS Ocean-1206 NMRP00000000 76 6905003 Open Ocean Resistant Computational Prediction "crpP,fosA,catB7,blaPAO,blaOXA-395,aph(3')-Iib" 99.1 100 0.3 Good 1 1 1 1 1 1 1 0 0 0 0 1

Pseudomonas aeruginosa strain PAH13 287 WGS PAH13 NQWG00000000 83 6700021 hospital effluent Brazil Resistant Computational Prediction "crpP,aac(6')-Ib-cr,fosA,rmtD,aph(3')-Iib,aac(6')-Ib3,aadA7,cmx,catB7,sul1,blaOXA-396,blaPAO,blaOXA-494,blaOXA-56,blaSPM-1,qacE" 99 100 0.3 Good 1 1 1 1 1 1 1 0 0 1 0 1

Pseudomonas aeruginosa strain PA404 287 WGS PA404 NQWF00000000 146 6819751 clinical Brazil Resistant Computational Prediction "fosA,aac(6')-Ib-cr,aph(3')-Iib,aac(6')-Ib3,aph(3'')-Ib,aph(6)-Id,ant(2'')-Ia,aadA6,sul1,dfrA5,cmx,catB7,catB3,blaPAO,blaOXA-488,blaOXA-129,qacE" 98.7 99.7 0.7 Good 1 1 0 1 1 1 1 0 0 1 0 1

Pseudomonas aeruginosa strain SJU-S6_3 287 WGS SJU-S6_3 NRFH00000000 74 6222453 soil United States Resistant Computational Prediction "fosA,catB7,blaPAO,blaOXA-488,blaOXA-396,aph(3')-Iib" 99.6 100 Good 1 1 1 1 1 1 1 0 0 0 0 0

Pseudomonas aeruginosa strain PA327 287 WGS PA327 NQWE00000000 170 6749268 clinical Brazil Resistant Computational Prediction "crpP,aac(6')-Ib-cr,fosA,aph(3')-Iib,aadA7,aac(6')-Ib3,rmtD,cmx,catB7,blaOXA-56,blaPAO,blaOXA-494,blaOXA-396,blaSPM-1,qacE" 97.6 99.4 1.8 Good 1 1 1 1 1 1 1 0 0 1 0 1

Pseudomonas aeruginosa strain 392 287 WGS 392 MWYI00000000 73 6995410 infected lung Canada Resistant Computational Prediction "crpP,fosA,catB7,blaPAO,blaOXA-396,aph(3')-Iib" 99.1 100 Good 1 1 1 1 1 1 1 0 0 0 0 1

Pseudomonas aeruginosa strain 265 287 WGS 265 MWXJ00000000 37 6857537 Sputum Canada Resistant Computational Prediction "crpP,fosA,catB7,blaPAO,blaOXA-396,aph(3')-Iib" 99.1 100 Good 1 1 1 1 1 1 1 0 0 0 0 1

Pseudomonas aeruginosa strain W1c 287 WGS W1c NQZC00000000 72 6746577 lake water United States Resistant Computational Prediction "crpP,fosA,catB7,blaPAO,blaOXA-50,aph(3')-Iib" 99 100 Good 1 1 0 1 1 1 1 0 0 0 0 1

Pseudomonas aeruginosa strain S30a 287 WGS S30a NQZI00000000 226 6463857 soil United States Resistant Computational Prediction "crpP,fosA,catB7,blaPAO,blaOXA-486,aph(3')-Iib" 98.2 100 0.6 Good 1 1 0 1 1 1 1 0 0 0 0 1

Pseudomonas aeruginosa strain S1h 287 WGS S1h NQZP00000000 66 6474747 soil United States Resistant Computational Prediction "crpP,fosA,catB7,blaPAO,blaOXA-50,aph(3')-Iib" 99.4 100 Good 1 1 0 1 1 1 1 0 0 1 0 1

Pseudomonas aeruginosa strain S1g 287 WGS S1g NQZQ00000000 76 6474249 soil United States Resistant Computational Prediction "crpP,fosA,catB7,blaPAO,blaOXA-50,aph(3')-Iib" 99.4 100 Good 1 1 0 1 1 1 1 0 0 1 0 1

Pseudomonas aeruginosa strain S1e 287 WGS S1e NQZR00000000 78 6472664 soil United States Resistant Computational Prediction "crpP,fosA,catB7,blaPAO,blaOXA-50,aph(3')-Iib" 99.4 100 Good 1 1 0 1 1 1 1 0 0 1 0 1

Pseudomonas aeruginosa strain env345 287 WGS env345 NQZX00000000 105 6788260 sink United States Resistant Computational Prediction "crpP,fosA,catB7,blaPAO,blaOXA-50,aph(3')-Iib" 98.4 100 0.3 Good 1 1 0 1 1 1 1 0 0 0 0 1

Pseudomonas aeruginosa strain env165 287 WGS env165 NRAG00000000 103 6419204 sink drain United States Resistant Computational Prediction "fosA,catB7,blaPAO,blaOXA-488,aph(3')-Iib" 99.3 100 Good 1 1 0 1 1 1 1 0 0 0 0 0

Pseudomonas aeruginosa strain SJU-S9_3 287 WGS SJU-S9_3 NRFE00000000 72 6223177 soil United States Resistant Computational Prediction "fosA,catB7,blaPAO,blaOXA-396,blaOXA-494,aph(3')-Iib" 99.7 100 Good 1 1 1 1 1 1 1 0 0 0 0 0

Pseudomonas aeruginosa strain SJU-S6_2 287 WGS SJU-S6_2 NRFI00000000 83 6224153 soil United States Resistant Computational Prediction "fosA,catB7,blaPAO,blaOXA-396,blaOXA-494,aph(3')-Iib" 99.6 100 Good 1 1 1 1 1 1 1 0 0 0 0 0

Pseudomonas aeruginosa strain SJU-S6_1 287 WGS SJU-S6_1 NRFJ00000000 71 6223377 soil United States Resistant Computational Prediction "fosA,catB7,blaPAO,blaOXA-396,blaOXA-494,aph(3')-Iib" 99.7 100 Good 1 1 1 1 1 1 1 0 0 0 0 0

Pseudomonas aeruginosa strain SJU-S9_2 287 WGS SJU-S9_2 NRFF00000000 68 6224023 soil United States Resistant Computational Prediction "fosA,catB7,blaPAO,blaOXA-396,blaOXA-494,aph(3')-Iib" 99.7 100 Good 1 1 1 1 1 1 1 0 0 0 0 0

Pseudomonas aeruginosa strain SJU-S9_1 287 WGS SJU-S9_1 NRFG00000000 77 6223053 soil United States Resistant Computational Prediction "fosA,catB7,blaPAO,blaOXA-396,blaOXA-494,aph(3')-Iib" 99.6 100 Good 1 1 1 1 1 1 1 0 0 0 0 0

Pseudomonas aeruginosa strain S1f 287 WGS S1f NRFP00000000 99 6471987 soil United States Resistant Computational Prediction "crpP,fosA,catB7,blaPAO,blaOXA-50,aph(3')-Iib" 99.3 100 Good 1 1 0 1 1 1 1 0 0 1 0 1

Pseudomonas aeruginosa strain env189 287 WGS env189 NRGC00000000 291 6819151 shower drain United States Resistant Computational Prediction "crpP,fosA,catB7,blaPAO,blaOXA-396,aph(3')-Iib" 97.5 100 1.3 Good 1 1 1 1 1 1 1 0 0 0 0 1

Pseudomonas aeruginosa strain env091 287 WGS env091 NRBP00000000 102 7007827 sink drain United States Resistant Computational Prediction "crpP,fosA,catB7,blaPAO,blaOXA-396,aph(3')-Iib" 99 100 Good 1 1 1 1 1 1 1 0 0 0 0 1

Pseudomonas aeruginosa strain Ocean-1175 287 Complete Ocean-1175 CP022525 1 6943220 Open Ocean Resistant Computational Prediction "crpP,fosA,catB7,blaPAO,blaOXA-395,aph(3')-Iib" 99.1 100 0.3 Good 1 1 1 1 1 1 1 0 0 0 0 1

Pseudomonas aeruginosa strain Ocean-1155 287 Complete Ocean-1155 CP022526 1 6952237 Open Ocean Resistant Computational Prediction "crpP,fosA,catB7,blaPAO,blaOXA-395,aph(3')-Iib" 99 100 0.9 Good 1 1 1 1 1 1 1 0 0 0 0 1

Pseudomonas aeruginosa strain PA83 287 Complete PA83 "CP017293,CP017294" 2 7214314 Germany "Human, Homo sapiens" Resistant Computational Prediction "crpP,fosA,catB7,aac(3)-Id,aac(6')-Il,aph(3')-Iib,aadA2,sul1,dfrB5,tet(G),cmlA1,catB7,blaPAO,blaOXA-4,blaVIM-2,blaOXA-486,qacE" 99 100 0.3 Good 1 1 1 1 1 1 1 0 1 1 0 1

Pseudomonas aeruginosa strain Ocean-1175 287 Complete Ocean-1175 CP022525 1 6943220 Open Ocean Resistant Computational Prediction "crpP,fosA,catB7,blaPAO,blaOXA-395,aph(3')-Iib" 99.1 100 0.3 Good 1 1 1 1 1 1 1 0 0 0 0 1

Pseudomonas aeruginosa strain Ocean-1155 287 Complete Ocean-1155 CP022526 1 6952237 Open Ocean Resistant Computational Prediction "crpP,fosA,catB7,blaPAO,blaOXA-395,aph(3')-Iib" 99 100 0.9 Good 1 1 1 1 1 1 1 0 0 0 0 1

Pseudomonas aeruginosa strain ICBSVIM-2 287 WGS ICBSVIM-2 NWBW01000000 184 6980737 environmental swab Brazil Resistant Computational Prediction "crpP,aadA2b,aac(3)-Id,aac(6')-Il,aph(3')-Iib,fosA,dfrB5,tet(G),catB7,cmlA1,blaOXA-486,blaOXA-4,blaVIM-2,blaPAO,qacE" 98.8 100 0.3 Good 1 1 1 1 1 1 1 0 1 1 0 0

Pseudomonas aeruginosa strain ICBBVIM-2 287 WGS ICBBVIM-2 NWBV01000000 341 7029318 Oral swab Brazil "Dog, Canis lupus familiaris" Resistant Computational Prediction "crpP,aadA2b,aac(3)-Id,aac(6')-Il,aph(3')-Iib,fosA,dfrB5,tet(G),catB7,cmlA1,blaOXA-486,blaOXA-4,blaVIM-2,blaPAO,qacE" 98.4 100 0.9 Good 1 1 1 1 1 1 1 0 1 1 0 0

Pseudomonas aeruginosa strain RNS_PA69 287 WGS RNS_PA69 LVED01000000 345 7053741 Blood culture Australia "Human, Homo sapiens" Resistant Computational Prediction "crpP,aac(6')-Ib-cr,fosA,aadA6,tet(G),aac(6')-Ib3,aph(3')-Iib,aph(3')-XV,catB7,blaOXA-488,blaPAO,blaGES-5,qacE" 96.7 99.9 1.6 Good 1 1 0 1 1 1 1 0 1 1 0 1

Pseudomonas aeruginosa strain RNS_PA51 287 WGS RNS_PA51 LVEC01000000 603 6920270 Pseudomonas aeruginosa isolate from a burns ward Australia "Human, Homo sapiens" Resistant Computational Prediction ",aac(6')-Ib-cr,fosA,aadA6,tet(G),aac(6')-Ib3,aph(3')-Iib,aph(3')-XV,catB7,blaOXA-488,blaPAO,blaGES-1,qacE" 94.8 100 3.9 Good 1 1 0 1 1 1 1 0 1 1 0 1

Pseudomonas aeruginosa strain RNS_PAE08 287 WGS RNS_PAE08 LVEF01000000 273 7017577 Hospital gel hand wash Australia Resistant Computational Prediction "crpP,aac(6')-Ib-cr,fosA,aadA6,tet(G),aac(6')-Ib3,aph(3')-Iib,aph(3')-XV,catB7,blaOXA-488,blaPAO,blaGES-5,qacE" 97.3 100 0.9 Good 1 1 0 1 1 1 1 0 1 1 0 1

Pseudomonas aeruginosa strain 142 287 WGS 142 NXHN01000000 378 6877144 pleural fluid Ghana "Human, Homo sapiens" Resistant Computational Prediction "crpP,fosA,aac(6')-Ib-cr,aadA1,aac(6')-Ib3,aph(3')-Iib,dfrB5,sul1,catB7,ARR-2,blaIMP-34,blaPAO,blaOXA-10,blaDIM-1,blaOXA-486,blaOXA-129,qacE" 97 100 1.2 Good 1 1 1 1 1 1 1 0 0 0 0 1

Pseudomonas aeruginosa strain 140 287 WGS 140 NXHO01000000 285 6840806 wound swab Ghana "Human, Homo sapiens" Resistant Computational Prediction "crpP,qnrVC1,aac(6')-Ib-cr,aac(6')-Ib3,aadA1,aph(3')-Iib,aadA10,sul1,dfrB5,catB7,ARR-2,blaDIM-1,blaPAO,blaOXA-129,blaOXA-486,blaIMP-34,qacE,fosA,blaOXA-10" 97.6 100 1.2 Good 1 1 1 1 1 1 1 0 1 0 0 1

Pseudomonas aeruginosa strain 130 287 WGS 130 NXHP01000000 274 6892960 wound swab Ghana "Human, Homo sapiens" Resistant Computational Prediction "crpP,aac(6')-Ib-cr,aac(6')-Ib3,aadA1,aph(3')-Iib,sul1,dfrB5,catB7,ARR-2,blaDIM-1,blaPAO,blaOXA-129,blaOXA-486,blaIMP-34,qacE,fosA,blaOXA-10" 97.7 100 0.6 Good 1 1 1 1 1 1 1 0 1 0 0 1

Pseudomonas aeruginosa strain 97 287 WGS 97 NXHR01000000 167 6909721 urine Ghana "Human, Homo sapiens" Resistant Computational Prediction "crpP,qnrVC1,aac(6')-Ib-cr,aac(6')-Ib3,aadA1,aph(3')-Iib,aadA10,sul1,dfrB5,catB7,ARR-2,blaDIM-1,blaPAO,blaOXA-129,blaOXA-486,blaIMP-34,qacE,fosA,blaOXA-10" 99.1 100 Good 1 1 1 1 1 1 1 0 1 0 0 1

Pseudomonas aeruginosa strain 85 287 WGS 85 NXHS01000000 127 6719926 wound swab Ghana "Human, Homo sapiens" Resistant Computational Prediction "aadA1,aph(3')-Iib,fosA,sul1,dfrA22,dfrA33,ARR-2,catB7.cmlA1,blaOXA-10,blaOXA-395,qacE" 98.7 100 0.6 Good 1 1 1 1 1 1 1 0 0 1 0 0

Pseudomonas aeruginosa strain PA_150577 287 Complete PA_150577 CP017306 1 6334472 Hong Kong "Human, Homo sapiens" Resistant Computational Prediction "fosA,catB7,blaPAO,blaOXA-396,blaOXA-488,aph(3')-Iib" 99.3 100 0.3 Good 1 1 0 1 1 1 1 0 0 0 0 0

Pseudomonas aeruginosa strain 12939 287 Complete 12939 CP024477 1 6621378 China "Human, Homo sapiens" Resistant Computational Prediction "crpP,fosA,catB7,blaOXA-395,blaPAO,aph(3')-Iib,aph(3')-Iia,aac(6')-Il,aac(3)-Iid,blaIMP-34,blaIMP-70,blaIMP-1," 99 100 0.6 Good 1 1 1 1 1 1 1 0 0 1 0 1

Pseudomonas aeruginosa strain PB369 287 Complete PB369 CP025049 1 6526814 Abscess United States "Human, Homo sapiens" Resistant Computational Prediction "fosA,catB7,blaPAO,blaOXA-396,blaOXA-488,aph(3')-Iib" 97.5 97.3 Good 1 1 0 1 1 1 1 0 0 0 0 0

Pseudomonas aeruginosa strain PB368 287 Complete PB368 CP025050 1 6638559 Abscess United States "Human, Homo sapiens" Resistant Computational Prediction "fosA,catB7,blaPAO,blaOXA-396,blaOXA-488,aph(3')-Iib" 98.2 98.2 Good 1 1 0 1 1 1 1 0 0 0 0 0

Pseudomonas aeruginosa strain PB350 287 Complete PB350 CP025055 1 6752870 sputum United States "Human, Homo sapiens" Resistant Computational Prediction "fosA,ant(2'')-Ia,aph(3')-Iib,dfrA10,sul1,catB7,blaOXA-488,blaPAO,qacE" 99 100 Good 1 1 0 1 1 1 1 0 0 1 0 0

Pseudomonas aeruginosa strain PB367 287 Complete PB367 CP025056 1 6752906 tracheal aspirate United States "Human, Homo sapiens" Resistant Computational Prediction "fosA,ant(2'')-Ia,aph(3')-Iib,dfrA10,sul1,catB7,blaOXA-488,blaPAO,qacE" 99 100 Good 1 1 0 1 1 1 1 0 0 1 0 0

Pseudomonas aeruginosa strain PA19 287 WGS PA19 PHST01000000 395 6927007 river Brazil Resistant Computational Prediction "crpP,aadA7,aac(6')-Ib-cr,aac(6')-Ib3,fosA,aph(3')-Iib,sul1,cmx,blaOXA-494,blaPAO,blaSPM-1,blaOXA-396,blaOXA-56,qacE" 96.6 100 4.4 Good 1 1 1 1 1 1 1 0 0 0 0 1

Pseudomonas aeruginosa strain PA151 287 WGS PA151 PHSS01000000 337 6799801 river Brazil Resistant Computational Prediction "rmtD,aadA7,aac(6')-Ib-cr,aac(6')-Ib3,aph(3')-Iib,fosA,sul1,cmx,catB7,blaPAO,blaOXA-494,blaOXA-396,blaOXA-54,blaSPM-1,qacE" 97.6 100 1.5 Good 1 1 1 1 1 1 1 0 0 1 0 1

Pseudomonas aeruginosa strain Pa64 287 WGS Pa64 PQGF01000000 294 6959022 soft tissue Brazil "Human, Homo sapiens" Resistant Computational Prediction "crpP,aac(6')-Ib-cr,aph(3')-Iib,aac(6')-Il,aac(6')-Ib3,fosA,sul1,ARR-4,catB3,catB7,blaOXA-395,blaPAO,blaGES-5,qacE" 97 99.7 0.9 Good 1 1 1 1 1 1 1 0 0 0 0 1

Pseudomonas aeruginosa strain W25637 287 WGS W25637 JPFE01000000 6 7301038 sputum United States "Human, Homo sapiens" Resistant Computational Prediction "crpP,aph(6)-Ic,aph(3')-Iib,aph(3')-Iia,aac(3)-Ia,aac(6')-Il,fosA,sul1,catB7,blaPAO,blaOXA-395,qacE" 98.5 100 0.9 Good 1 1 1 1 1 1 1 0 0 1 0 1

Pseudomonas aeruginosa strain M74707 287 WGS M74707 JPFA01000000 4 6431245 urine United States "Human, Homo sapiens" Resistant Computational Prediction "crpP,fosA,catB7,blaPAO,blaOXA-396,blaOXA-494,aph(3')-Iib" 99 98.8 0.9 Good 1 1 1 1 1 1 1 0 0 0 0 1

Pseudomonas aeruginosa strain W91453 287 WGS W91453 JPFG01000000 4 7149174 sputum United States "Human, Homo sapiens" Resistant Computational Prediction "crpP,aph(6)-Ic,aph(3')-Iib,aph(3')-Iia,aac(3)-Ia,aac(6')-Il,fosA,sul1,catB7,blaPAO,blaOXA-395,qacE" 99 100 1.3 Good 1 1 1 1 1 1 1 0 0 1 0 1

Pseudomonas aeruginosa strain M55212 287 WGS M55212 JPEY01000000 10 7355395 blood United States "Human, Homo sapiens" Resistant Computational Prediction "crpP,aph(6)-Ic,aph(3')-Iib,aph(3')-Iia,aac(3)-Ia,aac(6')-Il,fosA,sul1,catB7,blaPAO,blaOXA-395,qacE" 99.2 100 0.9 Good 1 1 1 1 1 1 1 0 0 1 0 1

Pseudomonas aeruginosa strain F5677 287 Complete F5677 CP026680 1 6645227 urine United States "Human, Homo sapiens" Resistant Computational Prediction "crpP,fosA,catB7,blaPAO,blaOXA-395,aph(3'')-Ib,aph(6)-Id,aph(3')-Iib" 99 99.7 0.3 Good 1 1 1 1 1 1 1 0 0 0 0 1

Pseudomonas aeruginosa strain AR_0360 287 Complete AR_0360 CP027165 1 6463575 Susceptible;Resistant;Intermediate AMR Panel "fosA,catB7,blaPAO,blaOXA-486,aph(3')-Iib" 99.3 100 Good 1 1 0 1 1 1 1 0 0 0 0 1

Pseudomonas aeruginosa strain AR_0354 287 Complete AR_0354 CP027171 1 6747010 Susceptible;Intermediate;Resistant AMR Panel "crpP,fosA,aph(6)-Id,aadA11,aph(3')-Iib,aph(3'')-Ib,ant(2'')-Ia,dfrB1,sul1,catB7,blaPAO,ant(2'')-Ia,qacE" 98.7 100 0.3 Good 1 1 0 1 1 1 1 0 0 1 0 1

Pseudomonas aeruginosa strain AR_0353 287 Complete AR_0353 "CP027172,CP027173" 2 7282236 Resistant;Intermediate;Susceptible AMR Panel "crpP,aac(6')-Ib-cr,fosA,ant(2'')-Ia,aadA1b,aac(6')-Il,aadA6,aac(6')-Il,aph(3')-Iib,aac(6')-Ib3,sul1,catB7,blaOXA-2,blaOXA-488,blaGES-1,qacE," 98.8 100 0.3 Good 1 1 0 1 1 1 1 0 0 1 0 1

Pseudomonas aeruginosa strain AR_0230 287 Complete AR_0230 "CP027174,CP027175,CP027176" 3 7086054 Resistant;Susceptible AMR Panel "crpP,fosA,aac(6')-Il,aadA2,aac(3)-Id,aph(3')-Iib,sul1,dfrB5,tet(G),catB7,cmlA1,blaPAO,blaVIM-2,blaOXA-4,blaOXA-486,qacE" 99.1 100 Good 1 1 0 1 1 1 1 0 1 1 0 1

Pseudomonas aeruginosa strain AR_0356 287 Complete AR_0356 "CP027169,CP027168,CP027170,CP027167" 4 7247865 Susceptible;Resistant AMR Panel "aadA16,aac(6')-Ib-cr,aac(6')-Ib3,sul1,tet(G),blaKPC-2,qacE" 97.6 98.4 1.5 Good 1 1 1 1 1 1 1 0 1 0 0 1

Pseudomonas aeruginosa strain AR_0357 287 Complete AR_0357 CP027166 1 7162784 Resistant;Susceptible AMR Panel "crpP,aadA1,ant(2'')-Ia,aph(3')-Iib,aadA2,aac(6')-Ip,aadA6,fosA,sul1,dfrA10,ARR-2,cmlA1,catB7,catB8,blaPAO,blaOXA-10,blaOXA-488,blaVEB-1,qacE" 98.9 100 Good 1 1 0 1 1 1 1 0 0 1 0 1

Pseudomonas aeruginosa strain WCHPA075063 287 WGS WCHPA075063 PPDQ01000000 93 6417188 China "Human, Homo sapiens" Resistant Computational Prediction "catB7,blaPAO,blaOXA-50,fosA,aph(3')-Iib" 99.8 100 Good 1 1 0 1 1 1 1 0 0 0 0 0

Pseudomonas aeruginosa strain WCHPA075056 287 WGS WCHPA075056 PPDR01000000 99 6412745 China "Human, Homo sapiens" Resistant Computational Prediction "catB7,blaPAO,blaOXA-50,fosA,aph(3')-Iib" 99.8 100 Good 1 1 0 1 1 1 1 0 0 0 0 0

Pseudomonas aeruginosa strain AR_0355 287 WGS AR_0355 PSQQ01000000 1 7172470 Susceptible;Resistant;Intermediate AMR Panel "crpP,fosA,catB7,blaPAO,blaOXA-50,aph(3'')-Ib,aph(6)-Id,aph(3')-Iib" 98.7 100 0.3 Good 1 1 0 1 1 1 1 0 0 0 0 1

Pseudomonas aeruginosa strain AR_0359 287 WGS AR_0359 PSQS01000000 3 6940345 Susceptible;Resistant;Intermediate AMR Panel "crpP,catB7,blaPAO,blaOXA-396,blaOXA-494,fosA,aph(3')-Iib" 98.5 100 Good 1 1 1 1 1 1 1 0 0 0 0 1

Pseudomonas aeruginosa strain AR_0358 287 WGS AR_0358 PSQR01000000 5 7284124 Susceptible;Resistant;Intermediate AMR Panel "crpP,aph(3')-Iib,ant(2'')-Ia,fosA,dfrA8,sul1,catB7,blaPAO,blaOXA-488,qacE" 98.7 100 Good 1 1 0 1 1 1 1 0 0 1 0 1

Pseudomonas aeruginosa strain AR_0351 287 WGS AR_0351 PSQP01000000 4 6611054 Resistant;Susceptible;Intermediate AMR Panel "crpP,catB7,blaPAO,blaOXA-488,blaOXA-50,blaOXA-485,fosA,aph(3')-Iib" 99.3 100 Good 1 1 0 1 1 1 1 0 0 0 0 1

Pseudomonas aeruginosa strain FLR01 287 WGS FLR01 PXNR01000000 70 6161034 sputum United States "Human, Homo sapiens" Resistant Computational Prediction "catB7,blaPAO,blaOXA-486,fosA,aph(3')-Iib" 99.3 100 0.2 Good 1 1 0 1 1 1 1 0 0 0 0 0

Pseudomonas aeruginosa strain MRSN12280 287 Complete MRSN12280 CP028162 1 7050928 Sacrum United States "Human, Homo sapiens" Resistant Computational Prediction "crpP,fosA,mcr-5.1,aph(3')-Iib,catB7,blaOXA-488,blaPAO" 98.7 100 0.2 Good 1 1 0 1 1 1 1 0 0 1 0 1

Pseudomonas aeruginosa strain KCRI-260 strain R0004_260 287 WGS R0004_260 OVCU01000000 284 6738557 wound swab sample Tanzania "Human, Homo sapiens" Resistant Computational Prediction "crpP,aadA1,ant(2'')-Ia,aph(3')-Iib,aac(6')-Il,aph(3')-VI,tet(A),fosA,sul1,dfrB2,ARR-3,catB7,cmlA1,blaOXA-50,blaOXA-10,blaPAO,blaNDM-1,blaVEB-1,qacE" 98.6 100 0.3 Good 1 1 1 1 1 1 1 0 1 1 0 1

Pseudomonas aeruginosa strain KCRI-242 strain R0008_242 287 WGS R0008_242 OVCQ01000000 108 6470142 sputum sample Tanzania "Human, Homo sapiens" Resistant Computational Prediction "crpP,catB7,blaPAO,blaOXA-396,blaOXA-494,fosA,aph(3')-Iib" 99.2 100 0.6 Good 1 1 1 1 1 1 1 0 0 0 0 1

Pseudomonas aeruginosa strain 424 287 WGS 424 FRTZ01000000 243 6298679 Resistant Computational Prediction "crpP,catB7,blaPAO,blaOXA-396,blaOXA-494,fosA,aph(3')-Iib" 97 100 0.6 Good 1 1 1 1 1 1 1 0 0 1 0 1

Pseudomonas aeruginosa strain PAL1.1 287 WGS PAL1.1 QFRM01000000 108 6780687 lung France "Human, Homo sapiens" Resistant Computational Prediction "fosA,aac(6')-Il,aph(3')-Iib,ant(2'')-Ia,aadA11,sul1,dfrB1,catB7,blaPAO,blaOXA-488,qacE" 99.1 100 Good 1 1 0 1 1 1 1 0 0 1 0 0

Pseudomonas aeruginosa strain PAL0.1 287 WGS PAL0.1 QFRL01000000 131 7040354 lung France "Human, Homo sapiens" Resistant Computational Prediction "crpP,aph(3')-Iib,fosA,aac(6')-29b,sul1,catB7,blaPAO,blaOXA-395,blaVIM-2,qacE" 99 100 0.3 Good 1 1 1 1 1 1 1 0 0 0 0 1

Pseudomonas aeruginosa strain AR_0443 287 Complete AR_0443 CP029147 1 6776714 Susceptible;Resistant;Intermediate AMR Panel "crpP,fosA,aadA1,aph(3')-Iib,aac(6')-Il,ant(2'')-Iasul1,dfrB2,tet(A),catB7,blaPAO,blaOXA-10,blaOXA-50,blaVEB-1,qacE" 99.3 100 Good 1 1 0 1 1 1 1 0 1 1 0 1

Pseudomonas aeruginosa strain AR_0440 287 Complete AR_0440 CP029148 1 7167215 Susceptible;Resistant AMR Panel "crpP,fosA,catB7,blaPAO,blaOXA-50,aph(3'')-Ib,aph(6)-Id,aph(3')-Iib" 98.7 100 0.6 Good 1 1 0 1 1 1 1 0 0 0 0 1

Pseudomonas aeruginosa strain AR439 287 Complete AR439 "CP029097,CP029095,CP029096" 3 7578039 Resistant;Susceptible;Intermediate AMR Panel "crpP,aph(3')-Iib,aadA1b,aac(6')-Il,fosA,sul1,catB7,blaPAO,blaOXA-396,blaIMP-18,qacE" 98.5 100 0.3 Good 1 1 1 1 1 1 1 0 0 0 0 0

Pseudomonas aeruginosa strain AR442 287 Complete AR442 CP029090 1 7267567 Susceptible;Resistant AMR Panel "crpP,aph(3')-Iib,ant(2'')-Ia,fosA,sul1,catB7,blaPAO,blaOXA-488,qacE" 98.8 100 Good 1 1 0 1 1 1 1 0 0 1 0 1

Pseudomonas aeruginosa strain AR445 287 Complete AR445 CP029088 1 7125975 Resistant;Susceptible AMR Panel "crpP,aac(6')-Ib-cr,aac(6')-Ib3,aph(3')-Iib,aadA2b,fosA,sul1,blaCARB-2,catB7,blaPAO,blaOXA-395,blaOXA-9,qacE" 99.1 100 Good 1 1 1 1 1 1 1 0 0 0 0 1

Pseudomonas aeruginosa strain AR441 287 Complete AR441 "CP029093,CP029091,CP029092,CP029094" 4 7245771 Susceptible;Resistant AMR Panel "aadA16,aac(6')-Ib-cr,aac(6')-Ib3,sul1,tet(G),blaKPC-2,qacE" 96.9 98.1 1.9 Good 1 1 1 1 1 1 0 0 0 0 0 1

Pseudomonas aeruginosa strain AR444 287 Complete AR444 CP029089 1 6853499 Resistant;Intermediate;Susceptible AMR Panel "crpP,aadA2,aac(6')-Il,aph(3')-Iib,fosA,sul1,dfrB5,tet(G),catB7,blaVIM-2,blaPAO,blaOXA-4,blaOXA-486,qacE" 99.4 100 Good 1 1 1 1 1 1 1 0 1 0 0 1

Pseudomonas aeruginosa strain HIAE_PA07 287 WGS HIAE_PA07 QBEP01000000 124 6970574 blood Brazil "Human, Homo sapiens" Resistant Computational Prediction "crpP,fosA,catB7,blaPAO,blaOXA-486,aph(3')-Iib" 99 100 Good 1 1 0 1 1 1 1 0 0 0 0 1

Pseudomonas aeruginosa strain HIAE_PA17 287 WGS HIAE_PA17 QBEF01000000 59 6735153 tracheal secretion Brazil "Human, Homo sapiens" Resistant Computational Prediction "fosA,aac(6')-Ib-cr,aadA1,aph(3')-Iib,aac(6')-Ib-Hangzhou,sul1,catB7,blaPAO,blaOXA-488,blaOXA-4,qacE" 98.8 100 Good 1 1 0 1 1 1 1 0 0 1 0 1

Pseudomonas aeruginosa strain HIAE_PA19 287 WGS HIAE_PA19 QBED01000000 88 6847954 tracheal secretion Brazil "Human, Homo sapiens" Resistant Computational Prediction "aadA6,aph(3'')-Ib,aph(3'')-Iib,aac(6')-Ib-cr,aac(6')-Ib3,aph(6)-Id,fosA,sul1,catB7,catB3,cmx,blaPAO,blaOXA-17,blaOXA-488,qacE" 98.8 100 Good 1 1 0 1 1 1 1 0 0 1 0 1

Pseudomonas aeruginosa strain HIAE_PA20 287 WGS HIAE_PA20 QBEC01000000 79 6812852 tracheal secretion Brazil "Human, Homo sapiens" Resistant Computational Prediction "aadA6,aac(6')-Ib-cr,aph(3'')-Ib,aph(3')-Iib,aph(6)-Id,ant(2'')-Ia,aac(6')-Ib3,fosA,sul1,dfrA5,cmx,catB3,catB7,blaPAO,blaOXA-129,blaOXA-488,qacE" 98.7 100 Good 1 1 0 1 1 1 1 0 0 1 0 1

Pseudomonas aeruginosa strain WCHPA075019 287 Complete WCHPA075019 CP028584 1 6886080 China "Human, Homo sapiens" Resistant Computational Prediction "crpP,fosA,catB7,blaPAO,blaOXA-494,blaOXA-396,aph(3')-Iib" 97.5 99.4 1.2 Good 1 1 1 1 1 1 1 0 0 0 0 1

Pseudomonas aeruginosa strain AR_0095 287 Complete AR_0095 CP027538 1 6822666 Susceptible;Not defined;Resistant;Intermediate AMR Panel "catB7,aph(3')-Iib,blapAO,blaOXA-395,fosA" 98.3 100 Good 1 1 1 1 1 1 1 0 0 1 0 0

Pseudomonas aeruginosa strain LIM1030 287 WGS QHLT01000000 451 7046243 anal abscess Brazil "Human, Homo sapiens" Resistant Computational Prediction "crpP,fosA,aac(6')-Il,aadA10,aph(3')-Iib,sul1,cmx,catB7,blaVIM-36,blaOXA-488,blaPAO,qacE" 97.7 100 1.5 Good 1 1 1 1 1 1 1 0 0 1 0 1

Pseudomonas aeruginosa strain LIM4447 287 WGS QHLV01000000 762 6770429 blood Brazil "Human, Homo sapiens" Resistant Computational Prediction "fosA,ant(2'')-Ia,aac(6')-Ib3,aph(3')-Iib,aac(6')-Ib-cr,aadA6,sul1,dfrA5,catB7,catB3,blaOXA-494,blaPAO,blaOXA-129,blaOXA-396,qacE" 92.8 100 7.2 Good 1 1 1 1 1 1 1 0 0 1 0 1

Pseudomonas aeruginosa strain AR_0446 287 Complete AR_0446 CP029660 1 6475581 Susceptible;Resistant;Intermediate AMR Panel "catB7,aph(3')-Iib,blapAO,blaOXA-50,fosA" 98.8 99.1 0.3 Good 1 1 0 1 1 1 1 0 0 0 0 0

Pseudomonas aeruginosa strain CCUG 70744 287 Complete CCUG 70744 CP023255 1 6859232 sputum Sweden "Human, Homo sapiens" Intermediate;Not defined;Resistant;Susceptible AMR Panel "crpP,catB7,aph(3')-Iib,blapAO,blaOXA-488,fosA" 99 99.7 Good 1 1 0 1 1 1 1 0 0 0 0 1

Pseudomonas aeruginosa strain LIM4308 287 WGS QJOX01000000 722 6758099 blood Brazil "Human, Homo sapiens" Resistant Computational Prediction "crpP,aac(6')-Ib-cr,aac(6')-Ib3,aph(3')-Iib,rmtD,aadA7,fosA,sul1,catB7,cmx,blaPAO,blaSPM-1,blaOXA-396,blaOXA-494,qacE" 96.2 100 2.4 Good 1 1 1 1 1 1 1 0 0 1 0 1

Pseudomonas aeruginosa strain LIM4293 287 WGS QJOY01000000 808 6912026 blood Brazil "Human, Homo sapiens" Resistant Computational Prediction "crpP,fosA,aac(6')-Ib-cr,aac(6')-Ib3,sul1,dfrA21,catB7,blaOXA-395,blaOXA-17,blaGES-5,blaPAO,qacE" 92.9 100 5.2 Good 1 1 1 1 1 1 1 0 0 0 0 1

Pseudomonas aeruginosa strain AR_0110 287 Complete AR_0110 CP029745 1 6799785 Resistant;Not defined;Susceptible;Intermediate AMR Panel "crpP,fosA,aac(6')-Il,aph(3')-Iib,aac(3)-Id,sul1,dfrB5,tet(G),catB7,blaOXA-486,blaPAO,blaVIM-2" 98.5 99.1 Good 1 1 1 1 1 1 1 0 1 1 0 1

Pseudomonas aeruginosa strain LIM1166 287 WGS QJOZ01000000 449 6720099 feces Brazil "Human, Homo sapiens" Resistant Computational Prediction "crpP,fosA,aac(6')-Ib-cr,aac(6')-Ib3,aph(3')-Iib,aadA7,sul1,cmx,catB7,blaPAO,blaSPM-1,blaOXA-56,blaOXA-494,blaOXA-396,qacE" 97.3 100 1.5 Good 1 1 1 1 1 1 1 0 0 0 0 1

Pseudomonas aeruginosa strain LIM1136 287 WGS QJPA01000000 302 6771881 blood Brazil "Human, Homo sapiens" Resistant Computational Prediction "crpP,aac(6')-Ib-cr,aph(3')-Iib,aadA7,,rmtD,aac(6')-Ib3,fosA,sul1,cmx,catB7,blaOXA-494,blaPAO,blaOXA-56,blaOXA-396,blaSPM-1,qacE" 98.2 100 0.9 Good 1 1 1 1 1 1 1 0 0 1 0 1

Pseudomonas aeruginosa strain LIM4519 287 WGS QJPC01000000 766 6720823 blood Brazil "Human, Homo sapiens" Resistant Computational Prediction "crpP,fosA,aac(6')-Ib-cr,aph(3')-Iib,rmtD,aac(6')-Ib3,aadA7,sul1,cmx,catB7,blaPAO,blaSPM-1,blaOXA-494,blaOXA-56,blaOXA-396,qace" 94.5 100 2.6 Good 1 1 1 1 1 1 1 0 0 1 0 1

Pseudomonas aeruginosa strain LIM1256 287 WGS QJPD01000000 532 6668575 blood Brazil "Human, Homo sapiens" Resistant Computational Prediction "crpP,aac(6')-Ib-cr,fosA,aadA7,aph(3')-Iib,aac(6')-Ib3,sul1,catB7,cmx,blaPAO,blaOXA-56,blaOXA-494,blaOXA-396,blaSPM-1,qacE" 95.3 99.7 2.5 Good 1 1 1 1 1 1 1 0 0 0 0 1

Pseudomonas aeruginosa strain LIM1595 287 WGS QJPF01000000 741 6776848 blood Brazil "Human, Homo sapiens" Resistant Computational Prediction "crpP,aac(6')-Ib-cr,rmtD,aph(3')-Iib,aac(6')-Ib3,aadA7,sul1,catB7,cmx,blaPAO,blaSPM-1,blaOXA-494,blaOXA-396,qacE" 97.2 100 2.1 Good 1 1 1 1 1 1 1 0 0 1 0 1

Pseudomonas aeruginosa strain LIM1209 287 WGS QJPG01000000 271 6760068 blood Brazil "Human, Homo sapiens" Resistant Computational Prediction "crpP,fosA,aac(6')-Ib-cr,aac(6')-Ib3,aadA7,sul1,catB7,cmx,blaPAO,blaOXA-396,blaOXA-56,blaOXA-494,qacE" 98.8 100 Good 1 1 1 1 1 1 1 0 0 0 0 1

Pseudomonas aeruginosa strain K34-7 287 Complete K34-7 "CP029707,CP029708" 2 7042452 tracheal secretions Norway "Human, Homo sapiens" Resistant Computational Prediction "crpP,aac(3)-Id,aadA2,aph(3')-Iib,aac(6')-Il,aac(3)-Id,fosA,sul1,dfrB5,tet(K),tet(G),catB7,cmlA1,blaPAO,blaVIM-2,blaOXA-4,blaOXA-486,qacE" 99 100 Good 1 1 1 1 1 1 1 0 0 1 0 1

Pseudomonas aeruginosa strain AR_0447 287 WGS AR_0447 QHCK01000000 2 7178792 Susceptible;Intermediate;Resistant AMR Panel "crpP,fosA,catB7,blaPAO,blaOXA-396,aph(3')-Iib" 98.7 100 0.6 Good 1 1 1 1 1 1 1 0 0 0 0 1

Pseudomonas aeruginosa strain AR_460 287 Complete AR_460 CP030351 1 6303875 Resistant;Susceptible AMR Panel "fosA,catB7,blaPAO,blaOXA-486,aph(3')-Iib" 98.7 100 1.2 Good 1 1 0 1 1 1 1 0 0 0 0 0

Pseudomonas aeruginosa strain AR_455 287 Complete AR_455 CP030328 1 6540996 Susceptible;Resistant;Intermediate AMR Panel "crpP,fosA,catB7,blaPAO,blaOXA-395,aph(3')-Iib" 97.9 97 Good 1 1 1 1 1 1 1 0 0 1 0 1

Pseudomonas aeruginosa strain AR_458 287 Complete AR_458 CP030327 1 6685102 Susceptible;Resistant;Intermediate AMR Panel "fosA,catB7,blaPAO,blaOXA-486,aph(3')-Iib" 99.3 100 Good 1 1 0 1 1 1 1 0 0 0 0 0

Pseudomonas aeruginosa strain AR_0459 287 WGS AR_0459 QMGQ01000000 1 6752712 Susceptible;Resistant AMR Panel "crpP,fosA,catB7,blaPAO,blaOXA-395,aph(3')-Iib" 98.6 100 Good 1 1 1 1 1 1 1 0 0 0 0 1

Pseudomonas aeruginosa strain AR_0457 287 WGS AR_0457 QMGP01000000 2 7381251 Susceptible;Resistant;Intermediate AMR Panel "crpP,aadA6,aph(3')-Iib,fosA,sul1,catB7,blaPAO,blaVIM-2,blaOXA-488,qacE" 97.1 100 0.5 Good 1 1 1 1 1 1 1 0 0 1 0 1

Pseudomonas aeruginosa strain AR_0456 287 WGS AR_0456 QMGO01000000 1 7079305 Susceptible;Resistant;Intermediate AMR Panel "crpP,fosA,aph(3')-Iib,aadA6,sul1,catB7,blaPAO,blaOXA-488,qacE" 98.6 100 0.4 Good 1 1 0 1 1 1 1 0 0 1 0 1

Pseudomonas aeruginosa strain AR_0449 287 WGS AR_0449 QMGL01000000 6 6792240 Susceptible;Resistant AMR Panel "crpP,aac(6')-Ib-cr,aac(6')-Ib3,aph(3')-Iib,fosA,sul1,catB7,blaOXA-488,qacE," 97.2 98.5 0.8 Good 1 1 0 1 1 1 1 0 0 1 0 1

Pseudomonas aeruginosa strain AR_0241 287 WGS AR_0241 QMGI01000000 2 7226502 Intermediate;Resistant;Susceptible AMR Panel "crpP,aac(6')-Ib-cr,fosA,aac(6')-Ib3,aac(6')-Iic,aph(3')-Iib,aadA7,catB7,blaPAO,blaIMP-1,blaOXA-9,blaOXA-395,blaOXA-101,qacE" 98.3 100 Good 1 1 1 1 1 1 1 0 0 1 0 1

Pseudomonas aeruginosa strain AR_0352 287 WGS AR_0352 QMGJ01000000 1 6418505 Susceptible;Resistant AMR Panel "fosA,catB7,blaPAO,blaOXA-396,blaOXA-50,aph(3')-Iib,blaOXA-494" 99.4 100 Good 1 1 1 1 1 1 1 0 0 1 0 0

Pseudomonas aeruginosa strain TUEPA7472 287 WGS TUEPA7472 QOLE01000000 18 6806824 blood Germany "Human, Homo sapiens" Resistant Computational Prediction "crpP,fosA,blaPAO,blaOXA-488,aph(3')-Iib" 98.5 100 Good 1 1 0 1 1 1 0 0 0 1 0 1

Pseudomonas aeruginosa strain G1-402 287 WGS G1-402 QORD01000000 408 7028805 urine Brazil "Human, Homo sapiens" Resistant Computational Prediction "crpP,aac(6')-Ib-cr,fosA,ant(2'')-Ia,aph(3')-Iib,aph(3')-Via,aadA6,aac(6')-Ib3,dfrB5,sul1,cmx,catB7,blaPAO,blaOXA-2,blaOXA-396,blaOXA-494,blaCTX-M-2,qacE" 98.2 100 1.2 Good 1 1 1 1 1 1 1 0 0 1 1 1

Pseudomonas aeruginosa strain D2-463 287 WGS D2-463 QORB01000000 417 6905990 cerebrospinal fluid Brazil "Human, Homo sapiens" Resistant Computational Prediction "crpP,aac(6')-Ib-cr,aph(3')-Iib,aph(3'')-Ib,ant(2'')-Ia,aph(6)-Id,aac(6')-Ib3,aadA6,fosA,dfrA5,sul1,catB7,catB3,cmx,blaOXA-488,blaPAO,blaOXA-129,blaCTX-M-2,qacE" 97.3 100 1.3 Good 1 1 0 1 1 1 1 0 0 1 0 1

Pseudomonas aeruginosa strain H2-9me 287 WGS H2-9me QOQY01000000 410 6954783 cerebrospinal fluid Brazil "Human, Homo sapiens" Resistant Computational Prediction "fosA,aac(6')-Ib-cr,aph(3')-Iib,aac(6')-Ib3,rmtD,aadA7,sul1,catB7,blaOXA-488,cmx,blaPAO,blaOXA-56,qacE" 96.8 100 1.8 Good 1 1 0 1 1 1 1 0 0 1 0 1

Pseudomonas aeruginosa strain F2-1206 287 WGS F2-1206 QORA01000000 403 7047893 cerebrospinal fluid Brazil "Human, Homo sapiens" Resistant Computational Prediction "crpP,aac(6')-Ib-cr,fosA,ant(2'')-Ia,aph(3')-Iib,aph(3')-Via,aadA2b,aac(6')-Ib3,dfrB5,sul1,cmlA1,catB7,blaPAO,blaOXA-2,blaGES-1,blaOXA-395,blaCTX-M-2,qacE" 97.6 99.7 1.2 Good 1 1 1 1 1 1 1 0 0 1 1 1

Pseudomonas aeruginosa strain I1-408 287 WGS I1-408 QORC01000000 435 7002409 blood Brazil "Human, Homo sapiens" Resistant Computational Prediction "crpP,aac(6')-Ib-cr,fosA,ant(2'')-Ia,aph(3')-Iib,aph(3')-Via,aadA6,aac(6')-Ib3,dfrB5,sul1,cmx,catB7,blaPAO,blaOXA-2,blaOXA-396,blaOXA-494,blaCTX-M-2,qacE" 98.6 100 0.3 Good 1 1 1 1 1 1 1 0 0 1 1 1

Pseudomonas aeruginosa strain PA37 strain Not applicable 287 WGS Not applicable QDGN01000000 238 7154299 eye India "Human, Homo sapiens" Resistant Computational Prediction "crpP,fosA,aph(3'')-Ib,aph(6)-Id,aph(3')-Iib,tet(G),catB7,blaPAO,blaOXA-488" 97.8 100 Good 1 1 0 1 1 1 1 0 1 1 0 1

Pseudomonas aeruginosa strain PA33 strain Not applicable 287 WGS Not applicable QDGP01000000 164 7092296 eye India "Human, Homo sapiens" Resistant Computational Prediction "crpP,fosA,aph(3'')-Ib,aph(6)-Id,aph(3')-Iib,tet(G),catB7,blaPAO,blaOXA-488" 98.2 100 Good 1 1 0 1 1 1 1 0 0 1 0 1

Pseudomonas aeruginosa strain PA32 strain Not applicable 287 WGS Not applicable QDGX01000000 154 7101461 eye India "Human, Homo sapiens" Resistant Computational Prediction "crpP,fosA,aph(3'')-Ib,aph(6)-Id,aph(3')-Iib,tet(G),catB7,blaPAO,blaOXA-488" 98.3 100 Good 1 1 0 1 1 1 1 0 0 1 0 1

Pseudomonas aeruginosa strain PA35 strain Not applicable 287 WGS Not applicable QDGO01000000 154 7094655 eye India "Human, Homo sapiens" Resistant Computational Prediction "crpP,fosA,aph(3'')-Ib,aph(6)-Id,aph(3')-Iib,tet(G),catB7,blaPAO,blaOXA-488" 98.2 100 Good 1 1 0 1 1 1 1 0 0 1 0 1

Pseudomonas aeruginosa strain PA31 strain Not applicable 287 WGS Not applicable QDGQ01000000 136 7100450 eye India "Human, Homo sapiens" Resistant Computational Prediction "crpP,fosA,aph(3'')-Ib,aph(6)-Id,aph(3')-Iib,tet(G),catB7,blaPAO,blaOXA-488" 98.4 100 Good 1 1 0 1 1 1 1 0 0 1 0 1

Pseudomonas aeruginosa strain Y89 287 Complete Y89 "CP030913,CP030914" 2 6954674 sputum South Korea "Human, Homo sapiens" Resistant Computational Prediction "crpP,aph(3')-VI,aac(6')-31,ant(2'')-Ia,,aph(3')-Iib,fosA,sul1,catB7,cmx,blaOXA-494,blaOXA-1,blaPAO,blaOXA-396" 98.5 100 Good 1 1 1 1 1 1 1 0 0 1 0 1

Pseudomonas aeruginosa strain Y82 287 Complete Y82 CP030912 1 7106857 sputum South Korea "Human, Homo sapiens" Resistant Computational Prediction "crpP,aph(3')-VI,aac(6')-31,ant(2'')-Ia,,aph(3')-Iib,fosA,sul1,catB7,cmx,blaOXA-1,blaPAO,blaOXA-395" 99 99.7 Good 1 1 1 1 1 1 1 0 0 1 0 1

Pseudomonas aeruginosa strain AUH-PA28 287 WGS AUH-PA28 QFTF01000000 118 7007723 Lebanon "Human, Homo sapiens" Resistant Computational Prediction "crpP,fosA,aph(3'')-Ib,aadA2,aac(6')-Il,aph(3')-Iib,aph(6)-Id,aac(3)-Id,dfrB5,tet(G),cmlA1,catB7,blaPAO,blaVIM-2,blaOXA-4,blaOXA-486,qacE" 98.6 100 Good 1 1 1 1 1 1 1 0 1 1 0 1

Pseudomonas aeruginosa strain AUH-PA45 287 WGS AUH-PA45 QFTH01000000 120 7009409 Lebanon "Human, Homo sapiens" Resistant Computational Prediction "crpP,fosA,aph(3'')-Ib,aadA2,aac(6')-Il,aph(3')-Iib,aph(6)-Id,aac(3)-Id,dfrB5,tet(G),cmlA1,catB7,blaPAO,blaVIM-2,blaOXA-4,blaOXA-486,qacE" 98.6 100 0.3 Good 1 1 1 1 1 1 1 0 1 1 0 1

Pseudomonas aeruginosa strain AUH-PA43 287 WGS AUH-PA43 QFTG01000000 189 6647372 Lebanon "Human, Homo sapiens" Resistant Computational Prediction "crpP,fosA,aph(3')-Iib,catB7,blaPAO,blaOXA-488" 99 100 Good 1 1 0 1 1 1 1 0 0 1 0 1

Pseudomonas aeruginosa strain AUH-PA16 287 WGS AUH-PA16 QFTE01000000 125 7007890 Lebanon "Human, Homo sapiens" Resistant Computational Prediction "crpP,fosA,aph(3'')-Ib,aadA2,aac(6')-Il,aph(3')-Iib,aph(6)-Id,aac(3)-Id,dfrB5,tet(G),cmlA1,catB7,blaPAO,blaVIM-2,blaOXA-4,blaOXA-486,qacE" 98.6 100 0.2 Good 1 1 1 1 1 1 1 0 1 1 0 1

Pseudomonas aeruginosa strain AUH-PA120 287 WGS AUH-PA120 QFTI01000000 122 6624004 Lebanon "Human, Homo sapiens" Resistant Computational Prediction "fosA,aph(3')-Iib,catB7,blaPAO,blaOXA-395" 98.8 100 Good 1 1 1 1 1 1 1 0 0 0 0 0

Pseudomonas aeruginosa strain AUH-PA15 287 WGS AUH-PA15 QFVU01000000 116 7005523 Lebanon "Human, Homo sapiens" Resistant Computational Prediction "crpP,fosA,aph(3'')-Ib,aadA2,aac(6')-Il,aph(3')-Iib,aph(6)-Id,aac(3)-Id,dfrB5,tet(G),cmlA1,catB7,blaPAO,blaVIM-2,blaOXA-4,blaOXA-486,qacE" 98.6 100 0.2 Good 1 1 1 1 1 1 1 0 1 1 0 1

Pseudomonas aeruginosa strain Y71 287 Complete Y71 CP030911 1 6940949 sputum South Korea "Human, Homo sapiens" Resistant Computational Prediction "crpP,fosA,aph(3')-IIb,ant(2'')-Ia,aac(6')-31,aph(3')-VI,catB7,cmx,blaPAO,sul1,blaOXA-1,blaOXA-494,blaOXA-396" 98.3 100 0.3 Good 1 1 1 1 1 1 1 0 0 1 0 1

Pseudomonas aeruginosa strain PABL092 287 WGS PABL092 QVBE01000000 214 6734993 blood United States "Human, Homo sapiens" Resistant Computational Prediction "crpP,fosA,blaPAO,blaOXA-486,aph(3')-Iib" 98.7 100 0.3 Good 1 1 0 1 1 1 1 0 0 0 0 1

Pseudomonas aeruginosa strain PABL108 287 WGS PABL108 QVAP01000000 275 6626283 blood United States "Human, Homo sapiens" Resistant Computational Prediction "fosA,catB7,blaPAO,blaOXA-50,aph(3')-Iib" 97.5 100 0.6 Good 1 1 0 1 1 1 1 0 0 1 0 0

Pseudomonas aeruginosa strain PABL090 287 WGS PABL090 QVBG01000000 179 6835148 blood United States "Human, Homo sapiens" Resistant Computational Prediction "crpP,fosA,aac(3)-Id,aac(6')-Il,aadA2,v,dfrB5,tet(G),catB7,cmlA1,blaPAO,blaVIM-2,blaOXA-486,blaOXA-4" 97.8 100 Good 1 0 0 0 1 1 1 0 1 0 0 1

Pseudomonas aeruginosa strain PABL104 287 WGS PABL104 QVAT01000000 176 6731702 blood United States "Human, Homo sapiens" Resistant Computational Prediction "crpP,fosA,catB7,blaPAO,blaOXA-486,aph(3')-Iib" 99.1 100 0.3 Good 1 1 0 1 1 1 1 0 0 0 0 1

Pseudomonas aeruginosa strain PABL071 287 WGS PABL071 QVBY01000000 556 6661186 blood United States "Human, Homo sapiens" Resistant Computational Prediction "crpP,fosA,catB7,blaPAO,blaOXA-488,aph(3')-Iib" 96.4 100 0.6 Good 1 1 0 1 1 1 1 0 0 0 0 1

Pseudomonas aeruginosa strain PABL097 287 WGS PABL097 QVAZ01000000 246 6897023 blood United States "Human, Homo sapiens" Resistant Computational Prediction "crpP,fosA,catB7,blaPAO,blaOXA-395,aph(3')-Iib" 97.5 100 Good 1 1 0 1 1 1 1 0 0 1 0 1

Pseudomonas aeruginosa strain PABL058 287 WGS PABL058 QVCL01000000 231 6909730 blood United States "Human, Homo sapiens" Resistant Computational Prediction "fosA,catB7,blaPAO,blaOXA-396,aph(3')-Iib" 97.3 100 0.6 Good 1 1 1 1 1 1 1 0 0 0 0 0

Pseudomonas aeruginosa strain PABL095 287 WGS PABL095 QVBB01000000 305 6566911 blood United States "Human, Homo sapiens" Resistant Computational Prediction "crpP,fosA,catB7,blaPAO,blaOXA-494,blaOXA-396,aph(3')-Iib" 97.8 100 0.9 Good 1 1 1 1 1 1 1 0 0 0 0 1

Pseudomonas aeruginosa strain PABL094 287 WGS PABL094 QVBC01000000 414 6986064 blood United States "Human, Homo sapiens" Resistant Computational Prediction "crpP,fosA,catB7,blaPAO,blaOXA-395,aph(3')-Iib" 97.9 100 Good 1 1 1 1 1 1 1 0 0 0 0 1

Pseudomonas aeruginosa strain PABL093 287 WGS PABL093 QVBD01000000 414 6956626 blood United States "Human, Homo sapiens" Resistant Computational Prediction "crpP,fosA,catB7,blaPAO,blaOXA-488,aph(3')-Iib" 97.3 100 0.6 Good 1 1 0 1 1 1 1 0 0 0 0 1

Pseudomonas aeruginosa strain PABL072 287 WGS PABL072 QVBX01000000 165 6726822 blood United States "Human, Homo sapiens" Resistant Computational Prediction "crpP,fosA,catB7,blaPAO,blaOXA-395,aph(3')-Iib" 98.1 100 Good 1 1 1 1 1 1 1 0 0 1 0 1

Pseudomonas aeruginosa strain PABL086 287 WGS PABL086 QVBJ01000000 452 7019938 blood United States "Human, Homo sapiens" Resistant Computational Prediction "crpP,aac(6')-Ib-cr,aph(3')-Iib,aadA2b,aac(6')-Ib3,fosA,sul1,catB7,blaPAO,blaCARB-2,blaOXA-395,qacE" 97.3 100 0.5 Good 1 1 1 1 1 1 1 0 0 0 0 1

Pseudomonas aeruginosa strain PABL082 287 WGS PABL082 QVBN01000000 495 7081190 blood United States "Human, Homo sapiens" Resistant Computational Prediction "crpP,fosA,catB7,blaPAO,blaOXA-50,aph(3')-Iib" 97.9 100 0.6 Good 1 1 0 1 1 1 1 0 0 0 0 1

Pseudomonas aeruginosa strain PABL038 287 WGS PABL038 QVDD01000000 234 6819052 blood United States "Human, Homo sapiens" Resistant Computational Prediction "crpP,fosA,catB7,blaPAO,blaOXA-395,aph(3')-Iib" 97.4 100 Good 1 1 1 1 1 1 1 0 0 0 0 1

Pseudomonas aeruginosa strain PABL079 287 WGS PABL079 QVBQ01000000 924 6569748 blood United States "Human, Homo sapiens" Resistant Computational Prediction "crpP,fosA,catB7,blaPAO,blaOXA-488,aph(3')-Iib" 92.2 100 7 Good 1 1 0 1 1 1 1 0 0 0 0 1

Pseudomonas aeruginosa strain PABL056 287 WGS PABL056 QVCN01000000 663 7331427 blood United States "Human, Homo sapiens" Resistant Computational Prediction "crpP,aadA10,ant(2'')-Ia,aph(3')-Iib,fosA,sul1,catB7,blaPAO,blaOXA-10,blaOXA-395,qacE" 95.8 100 0.6 Good 1 1 1 1 1 1 1 0 0 1 0 1

Pseudomonas aeruginosa strain PABL070 287 WGS PABL070 QVBZ01000000 270 6559142 blood United States "Human, Homo sapiens" Resistant Computational Prediction "fosA,catB7,blaPAO,blaOXA-50,aph(3')-Iib" 97.8 100 Good 1 1 0 1 1 1 1 0 0 0 0 0

Pseudomonas aeruginosa strain PABL067 287 WGS PABL067 QVCC01000000 576 6884951 blood United States "Human, Homo sapiens" Resistant Computational Prediction "crpP,fosA,catB7,blaPAO,blaOXA-395,aph(3')-Iib" 96.8 100 1.2 Good 1 1 1 1 1 1 1 0 0 1 0 1

Pseudomonas aeruginosa strain PABL044 287 WGS PABL044 QVCY01000000 396 6962745 blood United States "Human, Homo sapiens" Resistant Computational Prediction "crpP,aac(6')-Ib-cr,aadA2b,aac(6')-Ib3,fosA,aph(3')-Iib,sul1,catB7,blaPAO,blaOXA-395,blaCARB-2,qacE" 95.6 100 1.3 Good 1 1 1 1 1 1 1 0 0 0 0 1

Pseudomonas aeruginosa strain PABL064 287 WGS PABL064 QVCF01000000 524 6914320 blood United States "Human, Homo sapiens" Resistant Computational Prediction "crpP,aph(3')-Iib,fosA,sul1,catB7,ant(2'')-Ia,blaPAO,blaOXA-395,qacE" 95.3 100 3.4 Good 1 1 1 1 1 1 1 0 0 1 0 1

Pseudomonas aeruginosa strain PABL043 287 WGS PABL043 QVCZ01000000 276 6278847 blood United States "Human, Homo sapiens" Resistant Computational Prediction "crpP,aph(3'')-Ib,aph(6)-Id,sul1" 97 98.9 1.9 Good 0 0 0 0 0 0 0 0 0 0 0 1

Pseudomonas aeruginosa strain PABL046 287 WGS PABL046 QVCW01000000 97 6209810 blood United States "Human, Homo sapiens" Resistant Computational Prediction "fosA,catB7,blaPAO,blaOXA-486,aph(3')-Iib" 98.5 100 0.3 Good 1 1 0 1 1 1 1 0 0 0 0 0

Pseudomonas aeruginosa strain PABL055 287 WGS PABL055 QVCO01000000 191 6778918 blood United States "Human, Homo sapiens" Resistant Computational Prediction "crpP,fosA,catB7,blaPAO,blaOXA-488,aph(3')-Iib" 97.4 100 1 Good 1 1 0 1 1 1 1 0 0 0 0 1

Pseudomonas aeruginosa strain PABL063 287 WGS PABL063 QVCG01000000 491 6880375 blood United States "Human, Homo sapiens" Resistant Computational Prediction "crpP,aph(3')-Iib,aadA6,fosA,sul1,catB7,blaPAO,blaOXA-488,qacE" 97 100 0.3 Good 1 1 0 1 1 1 1 0 0 1 0 1

Pseudomonas aeruginosa strain PABL045 287 WGS PABL045 QVCX01000000 165 6207985 blood United States "Human, Homo sapiens" Resistant Computational Prediction "fosA,catB7,blaPAO,blaOXA-486,aph(3')-Iib" 97.8 99.4 1.8 Good 1 1 0 1 1 1 1 0 0 0 0 0

Pseudomonas aeruginosa strain PABL054 287 WGS PABL054 QVCP01000000 540 7082770 blood United States "Human, Homo sapiens" Resistant Computational Prediction "crpP,aac(6')-Ib-cr,aph(3')-Iib,aac(6')-Ib3,fosA,sul1,blaPAO,blaCARB-2,blaOXA-395,qacE" 98 100 0.6 Good 1 1 1 1 1 1 0 0 0 0 0 0

Pseudomonas aeruginosa strain PABL053 287 WGS PABL053 QVCQ01000000 305 6817898 blood United States "Human, Homo sapiens" Resistant Computational Prediction "crpP,fosA,aph(3')-Iib,aadA6,sul1,catB7,blaPAO,blaOXA-488,qacE" 97 100 0.6 Good 1 1 0 1 1 1 1 0 0 1 0 1

Pseudomonas aeruginosa strain PABL040 287 WGS PABL040 QVDC01000000 431 6837974 blood United States "Human, Homo sapiens" Resistant Computational Prediction "crpP,fosA,catB7,blaPAO,blaOXA-395,aph(3')-Iib" 97 99.4 1 Good 1 1 1 1 1 1 1 0 0 1 0 1

Pseudomonas aeruginosa strain PABL048 287 WGS PABL048 QVCU01000000 382 7160491 blood United States "Human, Homo sapiens" Resistant Computational Prediction "crpP,ant(2'')-Ia,aph(3')-Iib.aadA10,fosA,sul1,catB7,blaOXA-395,blaOXA-10,blaPAO,qacE" 96.2 99.4 1.2 Good 1 1 1 1 1 1 1 0 0 1 0 1

Pseudomonas aeruginosa strain PABL047 287 WGS PABL047 QVCV01000000 208 6586823 blood United States "Human, Homo sapiens" Resistant Computational Prediction "crpP,fosA,catB7,blaPAO,blaOXA-396,blaOXA-494,aph(3')-Iib" 98.2 100 Good 1 1 1 1 1 1 1 0 0 0 0 1

Pseudomonas aeruginosa strain PABL037 287 WGS PABL037 QVDE01000000 135 6975581 blood United States "Human, Homo sapiens" Resistant Computational Prediction "crpP,aac(6')-Ib-craac(6')-Ib3,aph(3')-Iib,fosA,sul1,blaOXA-395,blaCARB-2,blaPAO,qacE" 98.7 100 Good 1 1 1 1 1 1 1 0 0 0 0 1

Pseudomonas aeruginosa strain PABL036 287 WGS PABL036 QVDF01000000 375 7226411 blood United States "Human, Homo sapiens" Resistant Computational Prediction "crpP,fosA,ant(2'')-Ia,aadA10,aph(3')-Iib,sul1,catB7,blaOXA-395,blaPAO,blaOXA-10,qacE" 96.6 99.4 0.6 Good 1 1 1 1 1 1 1 0 0 1 0 1

Pseudomonas aeruginosa strain PABL035 287 WGS PABL035 QVDG01000000 320 6779989 blood United States "Human, Homo sapiens" Resistant Computational Prediction "crpP,fosA,catB7,blaPAO,blaOXA-396,aph(3')-Iib" 96.9 99.7 0.6 Good 1 1 1 1 1 1 1 0 0 0 0 1

Pseudomonas aeruginosa strain PABL010 287 WGS PABL010 QVEB01000000 465 6859097 blood United States "Human, Homo sapiens" Resistant Computational Prediction "crpP,fosA,catB7,blaPAO,blaOXA-395,aph(3')-Iib" 95.8 100 2.3 Good 1 1 1 1 1 1 1 0 0 0 0 1

Pseudomonas aeruginosa strain PABL031 287 WGS PABL031 QVDJ01000000 170 6757528 blood United States "Human, Homo sapiens" Resistant Computational Prediction "crpP,fosA,catB7,blaPAO,blaOXA-486,aph(3')-Iib" 98.2 100 0.3 Good 1 1 0 1 1 1 1 0 0 0 0 1

Pseudomonas aeruginosa strain PABL004 287 WGS PABL004 QVEF01000000 130 6479856 blood United States "Human, Homo sapiens" Resistant Computational Prediction "crpP,fosA,catB7,blaPAO,blaOXA-396,blaOXA-494,aph(3')-Iib" 98.9 99.7 0.6 Good 1 1 1 1 1 1 1 0 0 0 0 1

Pseudomonas aeruginosa strain PABL006 287 WGS PABL006 QVEE01000000 466 6810419 blood United States "Human, Homo sapiens" Resistant Computational Prediction "crpP,aadA6,aph(3')-Iib,fosA,sul1,catB7,blaPAO,blaOXA-488,qacE" 95.4 100 1 Good 1 1 0 1 1 1 1 0 0 1 0 1

Pseudomonas aeruginosa strain PABL021 287 WGS PABL021 QVDS01000000 512 6506522 blood United States "Human, Homo sapiens" Resistant Computational Prediction "crpP,fosA,catB7,blaPAO,blaOXA-395,aph(3')-Iib" 94.5 98.2 1.7 Good 1 1 1 1 1 1 1 0 0 1 0 1

Pseudomonas aeruginosa strain PABL029 287 WGS PABL029 QVDL01000000 611 6962225 blood United States "Human, Homo sapiens" Resistant Computational Prediction "crpP,fosA,aadA2b,aac(6')-Ib-cr,sul1,catB7,blaPAO,aph(3')-Iib,blaCARB-2,aac(6')-Ib3,blaOXA-395,qacE" 93.9 100 3.3 Good 1 1 1 1 1 1 1 0 0 0 0 1

Pseudomonas aeruginosa strain PABL020 287 WGS PABL020 QVDT01000000 509 6759755 blood United States "Human, Homo sapiens" Resistant Computational Prediction "crpP,fosA,catB7,blaPAO,blaOXA-395,aph(3')-Iib" 95.4 99.4 1.8 Good 1 1 1 1 1 1 1 0 0 1 0 1

Pseudomonas aeruginosa strain PABL015 287 WGS PABL015 QVDX01000000 346 6524861 blood United States "Human, Homo sapiens" Resistant Computational Prediction "crpP,fosA,catB7,blaPAO,blaOXA-486,aph(3')-Iib" 96.2 100 1.6 Good 1 1 0 1 1 1 1 0 0 0 0 1

Pseudomonas aeruginosa strain PABL091 287 WGS PABL091 QVBF01000000 417 6755000 blood United States "Human, Homo sapiens" Resistant Computational Prediction "crpP,fosA,catB7,blaPAO,blaOXA-486,aph(3')-Iib" 97 100 0.3 Good 1 1 0 1 1 1 1 0 0 0 0 1

Pseudomonas aeruginosa strain 24Pae112 287 Complete 24Pae112 CP029605 1 7097241 blood Colombia "Human, Homo sapiens" Resistant Computational Prediction "crpP,fosA,aph(3')-Iib,ant(2'')-Ia,aadA1,aac(6')-Il,sul1,catB7,blaPAO,blaOXA-2,blaOXA-15,blaOXA-488,blaKPC-2,qacE" 98.6 100 1.2 Good 1 1 1 1 1 1 1 0 0 1 0 1

Pseudomonas aeruginosa strain WPB100 287 Complete WPB100 CP031877 1 6869565 hospital wastewaters Singapore Resistant Computational Prediction "crpP,qnrVC1,msr€,sul2,dfrB5,fosA,aph(6)-Id,aac(3)-Id,aac(6')-Il,aph(3'')-Ib,aph(3')-Iib,aadA10,floR,catB7,blaPAO,blaOXA-488,blaNDM-1" 98.8 100 Good 1 1 1 1 1 1 1 1 0 1 0 1

Pseudomonas aeruginosa strain WPB099 287 Complete WPB099 CP031878 1 6872115 hospital wastewaters Singapore Resistant Computational Prediction "crpP,qnrVC1,msr€,sul2,dfrB5,fosA,aph(6)-Id,aac(3)-Id,aac(6')-Il,aph(3'')-Ib,aph(3')-Iib,aadA10,floR,catB7,blaPAO,blaOXA-488,blaNDM-1" 98.7 100 Good 1 1 1 1 1 1 1 1 0 1 0 1

Pseudomonas aeruginosa strain WPB101 287 Complete WPB101 CP031876 1 6871984 hospital wastewaters Singapore Resistant Computational Prediction "crpP,qnrVC1,msr€,sul2,dfrB5,fosA,aph(6)-Id,aac(3)-Id,aac(6')-Il,aph(3'')-Ib,aph(3')-Iib,aadA10,floR,catB7,blaPAO,blaOXA-488,blaNDM-1" 98.8 100 Good 1 1 1 1 1 1 1 1 0 1 0 1

Pseudomonas aeruginosa strain N15-01092 287 WGS N15-01092 CP012901 1 6972899 Canada Resistant Computational Prediction "catB7,fosA,sul1,blaPAO,blaNDM-1,blaOXA-396,aph(3')-Iib,aph(6)-Id,aph(3'')-Ib" 98.8 100 0.9 Good 1 1 1 1 1 1 1 0 0 0 0 0

Pseudomonas aeruginosa strain AR_0111 287 Complete AR_0111 "CP032257,CP032256" 2 7075653 Resistant;Not defined;Susceptible;Intermediate AMR Panel "crpP,fosA,aac(6')-Il,aadA2aph(3')-Iib,sul1,aac(3)-Id,dfrB5,cmlA1,catB7,tet(G),blaOXA-4blaVIM-2,blaOXA-486,qacE" 98.6 100 Good 1 1 1 1 1 1 1 0 1 1 0 1

Pseudomonas aeruginosa strain PA_139357 287 WGS PA_139357 LSQB01000000 156 6900170 Hong Kong "Human, Homo sapiens" Resistant Computational Prediction "crpP,fosA,catB7,blaPAO,blaOXA-488,aph(3')-Iib" 98.1 100 1.2 Good 1 1 0 1 1 1 1 0 0 1 0 1

Pseudomonas aeruginosa strain PA_150210 287 WGS PA_150210 LSQD01000000 111 6784277 Hong Kong "Human, Homo sapiens" Resistant Computational Prediction "crpP,fosA,catB7,blaPAO,blaOXA-50,aph(3')-Iib" 98.3 99.4 0.6 Good 1 1 0 1 1 1 1 0 0 0 0 1

Pseudomonas aeruginosa strain PA_153543 287 WGS PA_153543 LSQM01000000 75 6682948 Hong Kong "Human, Homo sapiens" Resistant Computational Prediction "crpP,fosA,catB7,blaPAO,blaOXA-494,blaOXA-396,aph(3')-Iib" 99.5 100 Good 1 1 1 1 1 1 1 0 0 0 0 1

Pseudomonas aeruginosa strain PA_152361 287 WGS PA_152361 LSQO01000000 89 6855632 Hong Kong "Human, Homo sapiens" Resistant Computational Prediction "crpP,fosA,catB7,blaPAO,blaOXA-395,aph(3')-Iib" 99.2 100 Good 1 1 1 1 1 1 1 0 0 0 0 1

Pseudomonas aeruginosa strain PA_150209 287 WGS PA_150209 LSQC01000000 112 6780930 Hong Kong "Human, Homo sapiens" Resistant Computational Prediction "crpP,fosA,catB7,blaPAO,blaOXA-50,aph(3')-Iib" 98.4 99.4 0.6 Good 1 1 0 1 1 1 1 0 0 0 0 1

Pseudomonas aeruginosa strain PA_152165 287 WGS PA_152165 LSQN01000000 70 6877291 Hong Kong "Human, Homo sapiens" Resistant Computational Prediction "crpP,fosA,catB7,blaPAO,blaOXA-488,aph(3')-Iib" 99.2 99.7 Good 1 1 0 1 1 1 1 0 0 0 0 1

Pseudomonas aeruginosa strain NCTC13719 287 WGS NCTC13719 UGUM01000000 4 7028349 Blood United Kingdom "Human, Homo sapiens" Resistant Computational Prediction "crpP,aadA1,aac(6')-Il,aph(3')-Iib,ant(2'')-Ia,fosA,sul1,dfrB2,tet(A),catB7,blaVEB-1,blaVIM-2,blaOXA-50,blaOXA-10,blaPAO,qacE" 98.9 100 0.9 Good 1 1 1 1 1 1 1 0 1 1 0 1

Pseudomonas aeruginosa strain BA7823 287 Complete BA7823 CP032569 1 6876788 blood India "Human, Homo sapiens" Resistant Computational Prediction "crpP,aac(6')-Ib-cr,aac(6')-Ib3,aph(3')-Iib,aac(6')-Il,aac(3)-Id,ant(2'')-Ia,fosA,sul1,dfrB5,catB7,blaLCR-1,blaPAO,blaVIM-2,blaOXA-50,qacE" 85.6 Good 1 1 1 1 1 1 1 0 0 1 0 1

Pseudomonas aeruginosa strain HUM-313-D2 287 WGS HUM-313-D2 RAGA01000000 356 6413754 tracheal aspirate Estonia "Human, Homo sapiens" Resistant Computational Prediction "crpP,fosA,catB7,blaPAO,blaOXA-494,blaOXA-396,aph(3')-Iib" 96 Good 1 1 1 1 1 1 1 0 0 0 0 1

Pseudomonas aeruginosa strain HUM-313-D1 287 WGS HUM-313-D1 RAFZ01000000 127 6412772 tracheal aspirate Estonia "Human, Homo sapiens" Resistant Computational Prediction "crpP,fosA,catB7,blaPAO,blaOXA-494,blaOXA-396,aph(3')-Iib" 98.6 Good 1 1 1 1 1 1 1 0 0 0 0 1

Pseudomonas aeruginosa strain HUM-291 287 WGS HUM-291 RAFY01000000 784 7034642 urine Estonia "Human, Homo sapiens" Resistant Computational Prediction "crpP,fosA,aph(6)-Id,aph(3')-Iib,aac(6')-Il,sul1,catB7,cmx,blaPAO,blaOXA-396,blaOXA-2,blaOXA-494,qacE" 94.2 Good 1 1 1 1 1 1 1 0 0 0 0 1

Pseudomonas aeruginosa strain HUM-339 287 WGS HUM-339 RAFX01000000 191 6960555 tracheal aspirate Estonia "Human, Homo sapiens" Resistant Computational Prediction "crpP,fosA,catB7,blaPAO,aph(6)-Id,blaOXA-488,aph(3'')-Ib,aph(3')-Iib" 98.6 Good 1 1 0 1 1 1 1 0 0 0 0 1

Pseudomonas aeruginosa strain VET-26 287 WGS VET-26 RAFO01000000 173 6585533 skin Estonia "Dog, Canis lupus familiaris" Resistant Computational Prediction "fosA,catB7,blaPAO,blaOXA-486,aph(3')-Iib" 98.2 Good 1 1 0 1 1 1 1 0 0 0 0 0

Pseudomonas aeruginosa strain HUM-325 287 WGS HUM-325 RAFL01000000 140 6839621 blood Estonia "Human, Homo sapiens" Resistant Computational Prediction "aac(3)-Iia,aph(3')-Iib,aph(3'')-Ib,aph(6)-Id,fosA,sul1,catB7,blaPAO,blaOXA-486,blaTEM-1B" 98.4 Good 1 1 0 1 1 1 1 0 0 1 0 0

Pseudomonas aeruginosa strain HUM-289 287 WGS HUM-289 RAFK01000000 99 6881801 urine Estonia "Human, Homo sapiens" Resistant Computational Prediction "crpP,aac(3)-Iia,aph(3')-Iib,aph(3'')-Ib,aph(6)-Id,fosA,sul1,catB7,blaPAO,blaOXA-486,blaTEM-1B" 98.6 Good 1 1 0 1 1 1 1 0 0 1 0 1

Pseudomonas aeruginosa strain HUM-235 287 WGS HUM-235 RAFJ01000000 126 6839093 tracheal aspirate Estonia "Human, Homo sapiens" Resistant Computational Prediction "aac(3)-Iia,aph(3')-Iib,aph(3'')-Ib,aph(6)-Id,fosA,sul1,catB7,blaPAO,blaOXA-486,blaTEM-1B" 98 Good 1 1 0 1 1 1 1 0 0 1 0 0

Pseudomonas aeruginosa strain HUM-286 287 WGS HUM-286 RAFI01000000 112 7363811 urine Estonia "Human, Homo sapiens" Resistant Computational Prediction "crpP,aph(6)-Id,aph(3'')-Ib,ant(2'')-Ia,aac(3)-Iia,aph(3')-Iib,aadA11,fosA,sul1,catB7,blaPAO,blaTEM-1B,blaOXA-486,qacE" 97.6 Good 1 1 0 1 1 1 1 0 0 1 0 1

Pseudomonas aeruginosa strain HUM-333 287 WGS HUM-333 RAFH01000000 130 6818370 blood Estonia "Human, Homo sapiens" Resistant Computational Prediction "aac(3)-Iia,aph(3')-Iib,aph(3'')-Ib,aph(6)-Id,fosA,sul1,catB7,blaPAO,blaOXA-486,blaTEM-1B" 98.7 Good 1 1 0 1 1 1 1 0 0 1 0 0

Pseudomonas aeruginosa strain VET-59 287 WGS VET-59 RAEW01000000 569 6773801 ear secretion Estonia "Dog, Canis lupus familiaris" Resistant Computational Prediction "crpP,fosA,catB7,blaPAO,blaOXA-494,blaOXA-396,aph(3')-Iib" 93 Good 1 1 1 1 1 1 1 0 0 0 0 1

Pseudomonas aeruginosa strain VET-51 287 WGS VET-51 RAES01000000 306 6520644 ear secretion Estonia "Dog, Canis lupus familiaris" Resistant Computational Prediction "crpP,fosA,catB7,blaPAO,blaOXA-494,blaOXA-396,aph(3')-Iib" 97.7 Good 1 1 1 1 1 1 1 0 0 0 0 1

Pseudomonas aeruginosa strain VET-53 287 WGS VET-53 RAER01000000 267 6512056 ear secretion Estonia "Dog, Canis lupus familiaris" Resistant Computational Prediction "fosA,catB7,blaPAO,blaOXA-494,blaOXA-396,aph(3')-Iib" 97.2 Good 1 1 1 1 1 1 1 0 0 0 0 0

Pseudomonas aeruginosa strain VET-50 287 WGS VET-50 RAEQ01000000 395 6520303 ear secretion Estonia "Dog, Canis lupus familiaris" Resistant Computational Prediction "fosA,catB7,blaPAO,blaOXA-494,blaOXA-396,aph(3')-Iib" 95.3 Good 1 1 1 1 1 1 1 0 0 0 0 0

Pseudomonas aeruginosa strain VET-67 287 WGS VET-67 RAEP01000000 421 6509976 ear secretion Estonia "Dog, Canis lupus familiaris" Resistant Computational Prediction "fosA,catB7,blaPAO,blaOXA-494,blaOXA-396,aph(3')-Iib" 96.2 Good 1 1 1 1 1 1 1 0 0 0 0 0

Pseudomonas aeruginosa strain VET-66 287 WGS VET-66 RAEO01000000 527 6515734 ear secretion Estonia "Dog, Canis lupus familiaris" Resistant Computational Prediction "fosA,catB7,blaPAO,blaOXA-494,blaOXA-396," 93.1 Good 1 1 1 1 1 1 1 0 0 0 0 0

Pseudomonas aeruginosa strain VET-65 287 WGS VET-65 RAEN01000000 507 6533735 ear secretion Estonia "Dog, Canis lupus familiaris" Resistant Computational Prediction "fosA,catB7,blaPAO,blaOXA-494,blaOXA-396,aph(3')-Iib" 94.5 Good 1 1 1 1 1 1 1 0 0 0 0 0

Pseudomonas aeruginosa strain VET-22 287 WGS VET-22 RAEJ01000000 133 6590976 ear secretion Estonia "Dog, Canis lupus familiaris" Resistant Computational Prediction "crpP,fosA,catB7,blaPAO,blaOXA-494,blaOXA-396,aph(3')-Iib" 98.3 Good 1 1 1 1 1 1 1 0 0 0 0 1

Pseudomonas aeruginosa strain HUM-298 287 WGS HUM-298 RAEF01000000 178 6897443 wound secretion Estonia "Human, Homo sapiens" Resistant Computational Prediction "crpP,aadA1,aph(3')-Iib,fosA,sul1,catB7,blaPAO,blaOXA-486,qacE" 98.1 Good 1 1 0 1 1 1 1 0 0 0 0 1

Pseudomonas aeruginosa strain HUM-300 287 WGS HUM-300 RAEC01000000 117 6385058 wound secretion Estonia "Human, Homo sapiens" Resistant Computational Prediction "crpP,aadA1,aph(3')-Iib,fosA,sul2,catB7,blaPAO,blaOXA-486," 98.4 Good 1 1 0 1 1 1 1 0 0 0 0 1

Pseudomonas aeruginosa strain HUM-315-D1 287 WGS HUM-315-D1 RAEA01000000 170 6802706 wound secretion Estonia "Human, Homo sapiens" Resistant Computational Prediction "crpP,fosA,catB7,blaPAO,blaOXA-486,aph(3')-Iib," 98.4 Good 1 1 0 1 1 1 1 0 0 0 0 1

Pseudomonas aeruginosa strain HUM-315-D2 287 WGS HUM-315-D2 RADZ01000000 259 6809382 wound secretion Estonia "Human, Homo sapiens" Resistant Computational Prediction "crpP,fosA,catB7,blaPAO,blaOXA-486,aph(3')-Iib," 97.3 Good 1 1 0 1 1 1 1 0 0 0 0 1

Pseudomonas aeruginosa strain HUM-337 287 WGS HUM-337 RADK01000000 128 6501103 urine Estonia "Human, Homo sapiens" Resistant Computational Prediction "crpP,fosA,catB7,blaPAO,blaOXA-486,aph(3')-Iib," 99 Good 1 1 0 1 1 1 1 0 0 0 0 1

Pseudomonas aeruginosa strain HUM-249 287 WGS HUM-249 RACK01000000 452 6832444 tracheal aspirate Estonia "Human, Homo sapiens" Resistant Computational Prediction "fosA,aph(3')-Ia,aph(3')-Iib,ant(2'')-Ia,sul1,catB7,tet(A),blaPAO,blaOXA-101,blaOXA-396,blaOXA-494,blaOXA-50,qacE" 98 Good 1 1 1 1 1 1 1 0 1 1 0 0

Pseudomonas aeruginosa strain HUM-292 287 WGS HUM-292 RACI01000000 121 6758380 wound secretion Estonia "Human, Homo sapiens" Resistant Computational Prediction "fosA,aph(3')-Ia,aph(3')-Iib,ant(2'')-Ia,sul1,catB7,tet(A),blaPAO,blaOXA-101,blaOXA-396,blaOXA-494,blaOXA-50,qacE" 98.7 Good 1 1 1 1 1 1 1 0 1 1 0 0

Pseudomonas aeruginosa strain HUM-228 287 WGS HUM-228 RACJ01000000 201 6740756 intraabdominal fluid Estonia "Human, Homo sapiens" Resistant Computational Prediction "fosA,aph(3')-Ia,aph(3')-Iib,ant(2'')-Ia,sul1,catB7,tet(A),blaPAO,blaOXA-101,blaOXA-396,blaOXA-494,blaOXA-50,qacE" 97.7 Good 1 1 1 1 1 1 1 0 1 1 0 0

Pseudomonas aeruginosa strain HUM-352 287 WGS HUM-352 RACH01000000 743 6822411 urine Estonia "Human, Homo sapiens" Resistant Computational Prediction "crpP,fosA,aph(3')-Ia,aph(3')-Iib,ant(2'')-Ia,sul1,catB7,tet(A),blaPAO,blaOXA-101,blaOXA-396,blaOXA-494,blaOXA-50,qacE" 91.1 Good 1 1 1 1 1 1 1 0 1 1 0 1

Pseudomonas aeruginosa strain HUM-239 287 WGS HUM-239 RACG01000000 215 6736725 wound secretion Estonia "Human, Homo sapiens" Resistant Computational Prediction "crpP,fosA,aph(3')-Iib,ant(2'')-Ia,sul1,tet(A),catB7,blaOXA-396,blaOXA-101,blaOXA-494,blaOXA-50,qacE" 98.3 Good 1 1 1 1 1 1 1 0 1 1 0 1

Pseudomonas aeruginosa strain HUM-248 287 WGS HUM-248 RACF01000000 97 6649890 tracheal aspirate Estonia "Human, Homo sapiens" Resistant Computational Prediction "fosA,sul1,aph(3')-Iib,ant(2'')-Ia,tet(A),catB7,blaPAO,blaOXA-494,blaOXA-101,blaOXA-396,blaOXA-50,qacE" 98.5 Good 1 1 1 1 1 1 1 0 0 1 0 0

Pseudomonas aeruginosa strain HUM-266 287 WGS HUM-266 RACD01000000 256 6873348 urine Estonia "Human, Homo sapiens" Resistant Computational Prediction "crpP,fosA,aph(3')-Ia,aph(3')-Iib,ant(2'')-Ia,sul1,catB7,tet(A),blaPAO,blaOXA-101,blaOXA-396,blaOXA-494,blaOXA-50,qacE" 98 Good 1 1 1 1 1 1 1 0 1 1 0 1

Pseudomonas aeruginosa strain HUM-317-D1 287 WGS HUM-317-D1 RACE01000000 110 6660335 bronchoalveolar lavage Estonia "Human, Homo sapiens" Resistant Computational Prediction "fosA,catB7,blaPAO,blaOXA-494,blaOXA-396,blaOXA-50,aph(3')-Iib" 98.9 Good 1 1 1 1 1 1 1 0 0 0 0 0

Pseudomonas aeruginosa strain HUM-287 287 WGS HUM-287 RACC01000000 120 6904523 tracheal aspirate Estonia "Human, Homo sapiens" Resistant Computational Prediction "fosA,aph(3')-Ia,aph(3')-Iib,ant(2'')-Ia,sul1,catB7,tet(A),blaPAO,blaOXA-101,blaOXA-396,blaOXA-494,blaOXA-50,qacE" 98.4 Good 1 1 1 1 1 1 1 0 1 1 0 0

Pseudomonas aeruginosa strain HUM-317-D2 287 WGS HUM-317-D2 RACB01000000 491 6659458 bronchoalveolar lavage Estonia "Human, Homo sapiens" Resistant Computational Prediction "fosA,catB7,blaPAO,blaOXA-494,blaOXA-396,blaOXA-50,aph(3')-Iib" 93.9 Good 1 1 1 1 1 1 1 0 0 0 0 0

Pseudomonas aeruginosa strain HUM-397 287 WGS HUM-397 RACA01000000 535 6752424 wound secretion Estonia "Human, Homo sapiens" Resistant Computational Prediction "crpP,fosA,catB7,blaPAO,blaOXA-494,blaOXA-396,blaOXA-50,aph(3')-Iib" 92.5 Good 1 1 1 1 1 1 1 0 0 0 0 1

Pseudomonas aeruginosa strain HUM-309 287 WGS HUM-309 RABZ01000000 306 6695528 wound secretion Estonia "Human, Homo sapiens" Resistant Computational Prediction "fosA,catB7,blaPAO,blaOXA-494,blaOXA-396,blaOXA-50,aph(3')-Iib" 97.1 Good 1 1 1 1 1 1 1 0 0 0 0 0

Pseudomonas aeruginosa strain HUM-310 287 WGS HUM-310 RABY01000000 154 6843946 wound secretion Estonia "Human, Homo sapiens" Resistant Computational Prediction "crpP,fosA,aph(3')-Ia,aph(3')-Iib,ant(2'')-Ia,sul1,catB7,tet(A),blaPAO,blaOXA-101,blaOXA-396,blaOXA-494,blaOXA-50,qacE" 97.7 Good 1 1 1 1 1 1 1 0 1 1 0 1

Pseudomonas aeruginosa strain HUM-307 287 WGS HUM-307 RABX01000000 755 6808365 wound secretion Estonia "Human, Homo sapiens" Resistant Computational Prediction "fosA,ant(2'')-Ia,aph(3')-Iib,aph(3')-Ia,sul1,tet(A),catB7,blaOXA-396,blaOXA-101,blaOXA-50,blaOXA-494,blaTEM-1B,qacE" 93.7 Good 1 1 1 1 1 1 1 0 1 1 0 0

Pseudomonas aeruginosa strain HUM-241 287 WGS HUM-241 RABJ01000000 139 6461456 urine Estonia "Human, Homo sapiens" Resistant Computational Prediction "crpP,fosA,catB7,blaPAO,blaOXA-494,blaOXA-396,blaOXA-50,aph(3')-Iib" 98.8 Good 1 1 1 1 1 1 1 0 0 0 0 1

Pseudomonas aeruginosa strain HUM-7 287 WGS HUM-7 RABI01000000 180 6419657 tracheal aspirate Estonia "Human, Homo sapiens" Resistant Computational Prediction "crpP,fosA,catB7,blaPAO,blaOXA-486,aph(3')-Iib" 98.3 Good 1 1 0 1 1 1 1 0 0 0 0 1

Pseudomonas aeruginosa strain VET-44 287 WGS VET-44 RABF01000000 374 6516698 ear secretion Estonia "Dog, Canis lupus familiaris" Resistant Computational Prediction "fosA,catB7,blaPAO,blaOXA-50,aph(3')-Iib" 98 Good 1 1 0 1 1 1 1 0 0 0 0 0

Pseudomonas aeruginosa strain VET-39-D2 287 WGS VET-39-D2 RABE01000000 92 6436081 ear secretion Estonia "Dog, Canis lupus familiaris" Resistant Computational Prediction "fosA,catB7,blaPAO,blaOXA-50,aph(3')-Iib" 99.4 Good 1 1 0 1 1 1 1 0 0 0 0 0

Pseudomonas aeruginosa strain VET-77 287 WGS VET-77 RABD01000000 412 6467478 ear secretion Estonia "Dog, Canis lupus familiaris" Resistant Computational Prediction "fosA,catB7,blaPAO,blaOXA-50,aph(3')-Iib" 95 Good 1 1 0 1 1 1 1 0 0 0 0 0

Pseudomonas aeruginosa strain HUM-257 287 WGS HUM-257 RABB01000000 111 6426230 bronchoalveolar lavage Estonia "Human, Homo sapiens" Resistant Computational Prediction "fosA,catB7,blaPAO,blaOXA-486,aph(3')-Iib" 99.3 Good 1 1 0 1 1 1 1 0 0 0 0 0

Pseudomonas aeruginosa strain HUM-253 287 WGS HUM-253 RABA01000000 94 6714069 urine Estonia "Human, Homo sapiens" Resistant Computational Prediction "crpP,fosA,catB7,blaPAO,blaOXA-50,aph(3')-Iib" 97.9 Good 1 1 0 1 1 1 1 0 0 0 0 1

Pseudomonas aeruginosa strain HUM-334 287 WGS HUM-334 RAAZ01000000 168 6644284 bronchoalveolar lavage Estonia "Human, Homo sapiens" Resistant Computational Prediction "fosA,catB7,blaPAO,blaOXA-50,aph(3')-Iib" 97.6 Good 1 1 0 1 1 1 1 0 0 0 0 0

Pseudomonas aeruginosa strain HUM-323-D2 287 WGS HUM-323-D2 RAAK01000000 703 6567303 bronchoalveolar lavage Estonia "Human, Homo sapiens" Resistant Computational Prediction "crpP,fosA,catB7,blaOXA-396,aph(3')-Iib" 91.1 Good 1 1 1 0 1 1 1 0 0 0 0 1

Pseudomonas aeruginosa strain HUM-323-D1 287 WGS HUM-323-D1 QZZS01000000 325 6641788 bronchoalveolar lavage Estonia "Human, Homo sapiens" Resistant Computational Prediction "crpP,fosA,catB7,blaPAO,blaOXA-396,aph(3')-Iib" 96.7 Good 1 1 1 1 1 1 1 0 0 0 0 1

Pseudomonas aeruginosa strain HUM-375 287 WGS HUM-375 QZZH01000000 609 6922561 urine Estonia "Human, Homo sapiens" Resistant Computational Prediction "crpP,aac(6')-Ib-cr,fosA,tet(G),aadA10,aph(3')-Iib,aac(6')-Ib3,aph(3')-XV,catB7,blaPAO,blaGES-5,blaOXA-488qacE" 93.3 Good 1 0 0 1 1 1 1 0 1 1 0 1

Pseudomonas aeruginosa strain HUM-231 287 WGS HUM-231 QZYZ01000000 152 6666129 sputum Estonia "Human, Homo sapiens" Resistant Computational Prediction "crpP,fosA,catB7,blaPAO,blaOXA-488,aph(3')-Iib" 98.2 Good 1 1 0 1 1 1 1 0 0 0 0 1

Pseudomonas aeruginosa strain HUM-237 287 WGS HUM-237 QZZA01000000 211 6988886 sputum Estonia "Human, Homo sapiens" Resistant Computational Prediction "crpP,fosA,catB7,blaPAO,blaOXA-488,aph(3')-Iib" 98.2 Good 1 1 0 1 1 1 1 0 0 0 0 1

Pseudomonas aeruginosa strain HUM-254 287 WGS HUM-254 QZYX01000000 148 6662298 tracheal aspirate Estonia "Human, Homo sapiens" Resistant Computational Prediction "crpP,fosA,catB7,blaPAO,blaOXA-488,aph(3')-Iib" 98.6 Good 1 1 0 1 1 1 1 0 0 0 0 1

Pseudomonas aeruginosa strain HUM-283 287 WGS HUM-283 QZYW01000000 112 6667621 tracheal aspirate Estonia "Human, Homo sapiens" Resistant Computational Prediction "crpP,fosA,catB7,blaPAO,blaOXA-488,aph(3')-Iib" 98.8 Good 1 1 0 1 1 1 1 0 0 0 0 1

Pseudomonas aeruginosa strain HUM-270 287 WGS HUM-270 QZYT01000000 78 6341042 wound secretion Estonia "Human, Homo sapiens" Resistant Computational Prediction "crpP,fosA,catB7,blaPAO,blaOXA-395,aph(3')-Iib" 98.5 Good 1 1 1 1 1 1 1 0 0 0 0 1

Pseudomonas aeruginosa strain ENV-246 287 WGS ENV-246 QZYR01000000 247 6091066 sewage Estonia Resistant Computational Prediction "crpP,fosA,catB7,blaPAO,blaOXA-395,aph(6)-Id,ant(2'')-Ia,aph(3')-Iib" 96.7 Good 1 1 1 1 1 1 1 0 0 1 0 1

Pseudomonas aeruginosa strain ENV-205 287 WGS ENV-205 QZYS01000000 234 6086858 sewage Estonia Resistant Computational Prediction "crpP,fosA,catB7,blaPAO,blaOXA-395,aph(6)-Id,ant(2'')-Ia,aph(3')-Iib" 97.3 Good 1 1 1 1 1 1 1 0 0 1 0 1

Pseudomonas aeruginosa strain ENV-247 287 WGS ENV-247 QZYQ01000000 367 6037268 sewage Estonia Resistant Computational Prediction "crpP,fosA,catB7,blaPAO,blaOXA-395,aph(6)-Id,ant(2'')-Ia,aph(3')-Iib" 95.7 Good 1 1 1 1 1 1 1 0 0 1 0 1

Pseudomonas aeruginosa strain ENV-227 287 WGS ENV-227 QZYL01000000 376 7089245 sewage Estonia Resistant Computational Prediction "crpP,fosA,catB7,blaPAO,blaOXA-488,aph(3')-Iib" 96.2 Good 1 1 0 1 1 1 1 0 0 0 0 1

Pseudomonas aeruginosa strain HUM-247 287 WGS HUM-247 QZYG01000000 196 6907778 wound secretion Estonia "Human, Homo sapiens" Resistant Computational Prediction "crpP,fosA,aph(3')-Iib,aadA7,sul1,catB7,blaPAO,blaOXA-488,qacE" 98.6 Good 1 1 0 1 1 1 1 0 0 0 0 1

Pseudomonas aeruginosa strain HUM-350 287 WGS HUM-350 QZYD01000000 394 6732821 tracheal aspirate Estonia "Human, Homo sapiens" Resistant Computational Prediction "crpP,aph(3')-Iib,blaOXA-488,catB7,blaPAO,fosA" 95 Good 1 1 0 1 1 1 1 0 0 1 0 1

Pseudomonas aeruginosa strain VET-64 287 WGS VET-64 QZZL01000000 498 6468191 ear secretion Estonia "Dog, Canis lupus familiaris" Resistant Computational Prediction "crpP,fosA,catB7,blaPAO,blaOXA-396,blaOXA-50,blaOXA-494,aph(3')-Iib" 93.9 Good 1 1 1 1 1 1 1 0 0 0 0 1

Pseudomonas aeruginosa strain HUM-258 287 WGS HUM-258 QZZD01000000 155 7118270 wound secretion Estonia "Human, Homo sapiens" Resistant Computational Prediction "crpP,fosA,aph(3'')-Ib,aph(6)-Id,aph(3')-Iib,sul1,tet(G),catB7,blaOXA-395,blaPAO,blaPME-1,blaNDM-1" 97.8 Good 1 1 1 1 1 1 1 0 1 0 0 1

Pseudomonas aeruginosa strain VET-24 287 WGS VET-24 QZZC01000000 157 6579663 ear secretion Estonia "Dog, Canis lupus familiaris" Resistant Computational Prediction "crpP,aph(3')-Iib,blaOXA-488,catB7,blaPAO,fosA" 98.5 Good 1 1 0 1 1 1 1 0 0 0 0 1

Pseudomonas aeruginosa strain HUM-236 287 WGS HUM-236 QZZB01000000 185 6991577 tracheal aspirate Estonia "Human, Homo sapiens" Resistant Computational Prediction "crpP,aph(3')-Iib,blaOXA-488,catB7,blaPAO,fosA" 97.8 Good 1 1 0 1 1 1 1 0 0 0 0 1

Pseudomonas aeruginosa strain HUM-256 287 WGS HUM-256 QZYY01000000 186 6671123 tracheal aspirate Estonia "Human, Homo sapiens" Resistant Computational Prediction "crpP,aph(3')-Iib,blaOXA-488,catB7,blaPAO,fosA" 97.3 Good 1 1 0 1 1 1 1 0 0 0 0 1

Pseudomonas aeruginosa strain HUM-282 287 WGS HUM-282 QZYP01000000 87 6390549 bronchoalveolar lavage Estonia "Human, Homo sapiens" Resistant Computational Prediction "crpP,aph(3')-Iib,blaOXA-395,catB7,blaPAO,fosA" 98.7 Good 1 1 1 1 1 1 1 0 0 0 0 1

Pseudomonas aeruginosa strain ENV-208 287 WGS ENV-208 QZYK01000000 822 7506148 sewage Estonia Resistant Computational Prediction "crpP,aph(3')-Iib,blaOXA-488,catB7,blaPAO,fosA" 92.4 Good 1 1 0 1 1 1 1 0 0 0 0 1

Pseudomonas aeruginosa strain HUM-301 287 WGS HUM-301 QZYF01000000 118 6957734 uterine swab Estonia "Human, Homo sapiens" Resistant Computational Prediction "aadA7,aph(3')-Iib,aph(6)-Id,aph(3'')-Ib,fosA,sul1,crpP,catB7,blaOXA-488,blaPAO,qacE" 98.7 Good 1 1 0 1 1 1 1 0 0 0 0 1

Pseudomonas aeruginosa strain HUM-227 287 WGS HUM-227 QZYA01000000 150 6758508 tracheal aspirate Estonia "Human, Homo sapiens" Resistant Computational Prediction "crpP,aph(3')-Iib,blaOXA-488,catB7,blaPAO,fosA" 97.6 Good 1 1 0 1 1 1 1 0 0 1 0 1

Pseudomonas aeruginosa strain VET-27 287 WGS VET-27 QZXZ01000000 161 6704983 skin Estonia "Dog, Canis lupus familiaris" Resistant Computational Prediction "crpP,aph(3')-Iib,blaOXA-488,catB7,blaPAO,fosA" 97.8 Good 1 1 0 1 1 1 1 0 0 1 0 1

Pseudomonas aeruginosa strain 6762 287 Complete 6762 CP030075 1 6714842 hospital China Resistant Computational Prediction "fosA,aadA2b,msr€,aac(6')-Ib-cr,aac(6')-Ib3,aph(3')-Iib,aac(6')-Il,sul1,crpP,blaPAO,blaVIM-4,blaOXA-395,mph€,qacE,catB7" 99.2 Good 1 1 1 1 1 1 1 1 0 0 0 1

Pseudomonas aeruginosa strain Pa1123 287 WGS Pa1123 NRBY01000000 130 6831240 University Hospital of Abidjan-Cocody Cote d'Ivoire "Human, Homo sapiens" Resistant Computational Prediction "fosA,aph(3')-Iib,aadA2,aac(6')-Il,aac(3)-Id,aac(3)-Id,dfrB5,tet(G),cmlA1,blaOXA-4,blaVIM-2,blaOXA-486,blaPAO,qacE" 98.4 Good 1 1 1 1 1 1 1 0 0 1 0 1

Pseudomonas aeruginosa strain Pa1810 287 WGS Pa1810 NRBX01000000 101 6852744 University Hospital of Abidjan-Cocody Cote d'Ivoire "Human, Homo sapiens" Resistant Computational Prediction "fosA,aph(3')-Iib,aadA2,aac(6')-Il,aac(3)-Id,aac(3)-Id,dfrB5,tet(G),cmlA1,blaOXA-4,blaVIM-2,blaOXA-486,blaPAO,qacE" 98.7 Good 1 1 1 1 1 1 1 0 0 1 0 1

Pseudomonas aeruginosa strain Pa1354 287 WGS Pa1354 NRBW01000000 113 6702439 University Hospital of Abidjan-Cocody Cote d'Ivoire "Human, Homo sapiens" Resistant Computational Prediction "fosA,aac(6')-Il,aac(3)-Id,aph(3')-Iib,crpP,dfrB5,blaVIM-2,blaOXA-486,blaPAO,catB7" 98.6 Good 1 1 1 1 1 1 1 0 0 1 0 1

Pseudomonas aeruginosa strain Pa2562 287 WGS Pa2562 NRBV01000000 64 6763469 University Hospital of Abidjan-Cocody Cote d'Ivoire "Human, Homo sapiens" Resistant Computational Prediction "fosA,aadA2,aph(3')-Iib,aac(3)-Id,aac(6')-Il,dfrB5,tet(G),crpP,blaOXA-486,blaOXA-4blaVIM-2,blaPAO,qacE,catB7,cmlA1" 99 Good 1 1 1 1 1 1 1 0 1 1 0 1

Pseudomonas aeruginosa strain Pa1060 287 WGS Pa1060 NRBU01000000 76 6851988 University Hospital of Abidjan-Cocody Cote d'Ivoire "Human, Homo sapiens" Resistant Computational Prediction "fosA,aadA2,aph(3')-Iib,aac(3)-Id,aac(6')-Il,dfrB5,tet(G),crpP,blaOXA-486,blaOXA-4blaVIM-2,blaPAO,qacE,catB7,cmlA1" 99 Good 1 1 1 1 1 1 1 0 1 1 0 1

Pseudomonas aeruginosa strain Pa2568 287 WGS Pa2568 NRBS01000000 82 6761342 University Hospital of Abidjan-Cocody Cote d'Ivoire "Human, Homo sapiens" Resistant Computational Prediction "fosA,aph(3')-Iib,aadA2,aac(6')-Il,aac(3)-Id,aac(3)-Id,dfrB5,tet(G),cmlA1,blaOXA-4,blaVIM-2,blaOXA-486,blaPAO,qacE" 99 Good 1 1 1 1 1 1 1 0 1 1 0 1

Pseudomonas aeruginosa strain Pa1780 287 WGS Pa1780 NRBQ01000000 99 6852001 University Hospital of Abidjan-Cocody Cote d'Ivoire "Human, Homo sapiens" Resistant Computational Prediction "aadA2,aac(3)-Id,aac(6')-Il,aph(3')-Iib,fosA,crpP,dfrB5,tet(G),blaPAO,blaVIM-2,blaOXA-486,blaOXA-4,cmlA1,catB7,qacE" 98.8 Good 1 1 1 1 1 1 1 0 1 1 0 1

Pseudomonas aeruginosa strain Pa1014 287 WGS Pa1014 NRBR01000000 102 6832053 University Hospital of Abidjan-Cocody Cote d'Ivoire "Human, Homo sapiens" Resistant Computational Prediction "fosA,aac(3)-Id,aac(6')-Il,aph(3')-Iib,crpP,sul1,dfrB5,blaVIM-2,blaOXA-486,blaPAO,blaOXA-4,catB7," 99 Good 1 1 1 1 1 1 1 0 0 1 0 1

Pseudomonas aeruginosa strain Pa1175 287 WGS Pa1175 NRBT01000000 98 6838612 University Hospital of Abidjan-Cocody Cote d'Ivoire "Human, Homo sapiens" Resistant Computational Prediction "aadA2,aac(3)-Id,aac(6')-Il,aph(3')-Iib,fosA,crpP,dfrB5,tet(G),blaPAO,blaVIM-2,blaOXA-486,blaOXA-4,cmlA1,catB7,qacE" 98.6 Good 1 1 1 1 1 1 1 0 1 1 0 1

Pseudomonas aeruginosa strain 55AA 287 WGS 55AA MCMY01000000 23 6379104 "Human, Homo sapiens" Resistant Computational Prediction "fosA,catB7,blaPAO,blaOXA-494,blaOXA-396,aph(3')-Iib" 99.1 Good 1 1 1 1 1 1 1 0 0 0 0 0

Pseudomonas aeruginosa strain E82A 287 WGS E82A MCML01000000 32 6343337 Cystic fibrosis patient Australia "Human, Homo sapiens" Resistant Computational Prediction "fosA,catB7,blaPAO,blaOXA-494,blaOXA-396,blaOXA-50,aph(3')-Iib" 99.2 Good 1 1 1 1 1 1 1 0 0 0 0 0

Pseudomonas aeruginosa strain 239A 287 WGS 239A MCMD01000000 95 6981334 patient with acute infection France "Human, Homo sapiens" Resistant Computational Prediction "fosA,aph(3')-Iib,sul1,aadA6,crpP,blaPAO,blaOXA-396,blaOXA-494,qacE,catB7" 98.5 Good 1 1 1 1 1 1 1 0 0 0 0 1

Pseudomonas aeruginosa strain F429 287 WGS F429 MCMA01000000 47 6843839 "Human, Homo sapiens" Resistant Computational Prediction "crpP,aph(3')-Iib,blaOXA-488,catB7,blaPAO,fosA" 98.9 Good 1 1 0 1 1 1 1 0 0 1 0 1

Pseudomonas aeruginosa strain D429(Q) strain D429(Q) strain D429(Q) 287 WGS D429(Q) MCMF01000000 109 6586986 Burn United States "Human, Homo sapiens" Resistant Computational Prediction "crpP,aph(3')-Iib,blaOXA-488,catB7,blaPAO,fosA,aph(3')-Iia" 98.1 Good 1 1 0 1 1 1 1 0 0 1 0 1

Pseudomonas aeruginosa strain EC22 287 WGS EC22 MCMK01000000 23 6209179 Cystic fibrosis patient Australia "Human, Homo sapiens" Resistant Computational Prediction "fosA,catB7,blaPAO,blaOXA-50,aph(3')-Iib" 99.6 Good 1 1 0 1 1 1 1 0 0 0 0 0

Pseudomonas aeruginosa strain ER06896 287 WGS ER06896 NTGC01000000 6 6766003 bronchoscopy tissue United States "Human, Homo sapiens" Resistant Computational Prediction "crpP,fosA,catB7,blaPAO,blaOXA-395,aph(3')-Iib" 98.2 Good 1 1 1 1 1 1 1 0 0 0 0 1

Pseudomonas aeruginosa strain PS00100 287 WGS PS00100 NTGD01000000 4 6804509 Humidifier basin United States Resistant Computational Prediction "crpP,fosA,catB7,blaPAO,blaOXA-395,aph(3')-Iib" 98.2 Good 1 1 1 1 1 1 1 0 0 0 0 1

Pseudomonas aeruginosa strain BA15561 287 Complete BA15561 CP033432 1 6793961 blood India "Human, Homo sapiens" Resistant Computational Prediction "aadA1,aph(3')-VI,aph(3')-Iib,ARR-3,sul1,crpP,dfrB2,tet(A),blaVEB-1,blaPAO,blaOXA-10,blaOXA-246,blaOXA-50,blaNDM-1,qacE,cmlA1,catB7" 95.6 Good 1 1 1 1 1 1 1 0 1 0 0 1

Pseudomonas aeruginosa strain SP4528 287 Complete SP4528 CP033439 1 6877287 sputum India "Human, Homo sapiens" Resistant Computational Prediction "aadA1,msr€,aph(3')-Iib,aac(6')-Il,ant(2'')-Ia,fosA,sul1,dfrB2,crpP,tet(A),blaPME-1,blaPAO,blaOXA-50,blaNDM-1,blaNDM-11,blaVEB-1,blaOXA-10,qacE,catB7" 96.9 Good 1 1 1 1 1 1 1 1 1 1 0 1

Pseudomonas aeruginosa strain PS1 287 WGS PS1 RHDU01000000 33 6720818 urine clinical sample Hungary "Human, Homo sapiens" Resistant Computational Prediction "fosA,aadA10,aph(3')-Iib,qnrVC1,blaPAO,blaNDM-1,blaOXA-395,qacE,catB7," 99.2 Good 1 1 0 1 1 1 1 0 0 1 0 1

Pseudomonas aeruginosa strain FDAARGOS_532 strain Not applicable 287 Complete Not applicable "CP033771,CP033772,CP033773" 3 6931252 Throat (pharynx) "Human, Homo sapiens" Resistant Computational Prediction "crpP,fosA,catB7,blaPAO,blaOXA-396,aph(3')-Iib" 98.7 Good 1 1 1 1 1 1 1 0 0 0 0 1

Pseudomonas aeruginosa strain FDAARGOS_571 strain Not applicable 287 Complete Not applicable CP033833 1 6999770 "Human, Homo sapiens" Resistant Computational Prediction "fosA,aph(3')-Iib,aadA6,aac(6')-Ib3,aac(6')-Ib-cr,sul1,tet(G),blaPAO,blaOXA-2,blaOXA-488,qacE,catB7" 98.8 Good 1 1 0 1 1 1 1 0 1 1 0 1

Pseudomonas aeruginosa strain FDAARGOS_501 strain Not applicable 287 Complete Not applicable CP033843 1 6865838 "Human, Homo sapiens" Resistant Computational Prediction "fosA,aph(3')-Iib,aac(3)-Ic,sul1,blaPAO,blaOXA-488,qacE,cmlA1,catB7" 98.7 Good 1 1 0 1 1 1 1 0 0 1 0 0

Pseudomonas aeruginosa strain FDAARGOS_505 strain Not applicable 287 Complete Not applicable CP033832 1 7029824 Endotracheal aspirate "Human, Homo sapiens" Resistant Computational Prediction "crpP,fosA,catB7,blaPAO,blaOXA-396,aph(3')-Iib" 99 Good 1 1 1 1 1 1 1 0 0 0 0 1

Pseudomonas aeruginosa strain FDAARGOS_570 strain Not applicable 287 Complete Not applicable "CP033835,CP033834" 2 7155137 "Human, Homo sapiens" Resistant Computational Prediction "crpP,aph(3'')-Ib,,aph(6)-Id,aac(6')-Ib3,aac(6')-Ib-cr,aph(3')-Iib,fosA,sul1,blaVIM-6,blaOXA-10,blaOXA-396,blaOXA-494,blaPAO,catB7,cmx,qacE" 99.3 Good 1 1 1 1 1 1 1 0 0 0 0 1

Pseudomonas aeruginosa strain E1-WATER-2 287 WGS E1-WATER-2 NSMS01000000 89 6989446 "Water line, poultry barn" Canada Resistant Computational Prediction "fosA,aph(3')-Iib,aadA6,crpP,sul1,blaOXA-494,blaPAO,blaOXA-396,catB7,qacE" 98.7 Good 1 1 1 1 1 1 1 0 0 0 0 1

Pseudomonas aeruginosa strain AL191 strain MCF191 287 WGS MCF191 NSMV01000000 54 6589375 throat Germany "Human, Homo sapiens" Resistant Computational Prediction "crpP,fosA,catB7,blaPAO,blaOXA-488,aph(3')-Iib" 99 Good 1 1 0 1 1 1 1 0 0 0 0 1

Pseudomonas aeruginosa strain PA-W37 287 WGS PA-W37 NSND01000000 49 6322616 toe wound United Kingdom "Human, Homo sapiens" Resistant Computational Prediction "crpP,fosA,catB7,blaPAO,blaOXA-494,blaOXA-396,aph(3')-Iib" 98.5 Good 1 1 1 1 1 1 1 0 0 0 0 1

Pseudomonas aeruginosa strain 21BR 287 WGS 21BR NSNX01000000 52 6668862 Brazil "Human, Homo sapiens" Resistant Computational Prediction "fosA,aac(6')-Ib3,aph(3')-Iib,aac(6')-Ib-cr,aadA7,sul1,crpP,blaPAO,blaOXA-494,blaOXA-396,blaOXA-56,qacE,catB7,cmx" 98.7 Good 1 1 1 1 1 1 1 0 0 0 0 1

Pseudomonas aeruginosa strain 205BR 287 WGS 205BR NSNZ01000000 54 6724009 Brazil "Human, Homo sapiens" Resistant Computational Prediction "fosA,aac(6')-Ib3,aadA7,aph(3')-Iib,sul1,catB7,crpP,blaOXA-396,blaPAO,blaOXA-494,blaSPM-1,blaOXA-56,qacE,cmx" 98.9 Good 1 1 1 1 1 1 1 0 0 0 0 1

Pseudomonas aeruginosa strain HB392 287 WGS HB392 NSON01000000 86 7024402 adult male urine Portugal "Human, Homo sapiens" Resistant Computational Prediction "crpP,fosA,catB7,blaPAO,blaOXA-494,blaOXA-396,aph(3')-Iib" 99.2 Good 1 1 1 1 1 1 1 0 0 0 0 1

Pseudomonas aeruginosa strain G1-WATER-2A 287 WGS G1-WATER-2A NSMP01000000 53 6848242 "Water line, poultry barn" Canada Resistant Computational Prediction "crpP,fosA,catB7,blaPAO,blaOXA-485,aph(3')-Iib" 98.9 Good 1 1 0 1 1 1 1 0 0 1 0 1

Pseudomonas aeruginosa strain PA-W43 287 WGS PA-W43 NSMZ01000000 50 6858332 shin wound United Kingdom "Human, Homo sapiens" Resistant Computational Prediction "crpP,fosA,catB7,blaPAO,blaOXA-396,aph(3')-Iib" 98.9 Good 1 1 1 1 1 1 1 0 0 0 0 1

Pseudomonas aeruginosa strain PA-W14 287 WGS PA-W14 NSNO01000000 71 6785995 foot ulcer United Kingdom "Human, Homo sapiens" Resistant Computational Prediction "aadA6,fosA,sul1,catB7,blaPAO,blaOXA-50,aph(3')-Iib" 98.3 Good 1 1 0 1 1 1 1 0 0 0 0 0

Pseudomonas aeruginosa strain 211BR 287 WGS 211BR NSOA01000000 59 6759888 Brazil "Human, Homo sapiens" Resistant Computational Prediction "aadA7,rmtD,aac(6')-Ib-cr,aac(6')-Ib3,aph(3')-Iib,fosA,crpP,blaPAO,blaOXA-396,blaOXA-56,blaOXA-494,qacE,catB7,cmx" 98.6 Good 1 1 1 1 1 1 1 0 0 1 0 1

Pseudomonas aeruginosa strain 36BR 287 WGS 36BR NSNW01000000 50 6709379 Brazil "Human, Homo sapiens" Resistant Computational Prediction "aadA7,rmtD,aac(6')-Ib-cr,aac(6')-Ib3,aph(3')-Iib,fosA,crpP,blaPAO,blaOXA-396,blaOXA-56,blaOXA-494,qacE,catB7,cmx,sul1" 98.5 Good 1 1 1 1 1 1 1 0 0 1 0 1

Pseudomonas aeruginosa strain 172BR 287 WGS 172BR NSNY01000000 46 6859879 Brazil "Human, Homo sapiens" Resistant Computational Prediction "aadA7,rmtD,aac(6')-Ib-cr,aac(6')-Ib3,aph(3')-Iib,fosA,crpP,blaPAO,blaOXA-396,blaOXA-56,blaOXA-494,qacE,catB7,blaSPM-1,sul1," 98.7 Good 1 1 1 1 1 1 1 0 0 1 0 1

Pseudomonas aeruginosa strain 175BR 287 WGS 175BR NSOB01000000 46 6854066 Brazil "Human, Homo sapiens" Resistant Computational Prediction "aadA7,rmtD,aac(6')-Ib-cr,aac(6')-Ib3,aph(3')-Iib,fosA,crpP,blaPAO,blaOXA-396,blaOXA-56,blaOXA-494,qacE,catB7,blaSPM-1,sul1," 98.7 Good 1 1 1 1 1 1 1 0 0 1 0 1

Pseudomonas aeruginosa strain CF27 287 WGS CF27 NSOU01000000 33 6492407 cystic fibrosis patient United States "Human, Homo sapiens" Resistant Computational Prediction "fosA,catB7,blaPAO,blaOXA-50,aph(3')-Iib" 99 Good 1 1 0 1 1 1 1 0 0 0 0 0

Pseudomonas aeruginosa strain JYH14 287 WGS JYH14 NSOI01000000 625 6823394 River Japan Resistant Computational Prediction "crpP,fosA,catB7,blaPAO,blaOXA-396,aph(3')-Iib" 91.4 Good 1 1 1 1 1 1 1 0 0 0 0 1

Pseudomonas aeruginosa strain X24509 287 WGS X24509 NSOP01000000 37 6405850 urinary tract infection United States "Human, Homo sapiens" Resistant Computational Prediction "crpP,fosA,catB7,blaPAO,blaOXA-486,aph(3')-Iib" 99.3 Good 1 1 0 1 1 1 1 0 0 0 0 1

Pseudomonas aeruginosa strain U2504 287 WGS U2504 NSOQ01000000 55 6827994 urinary tract infection United States "Human, Homo sapiens" Resistant Computational Prediction "fosA,aph(6)-Ic,aph(3')-Iib,aac(6')-Ib-cr,aac(6')-Ib3,aph(3')-Iia,sul1,blaOXA-488,blaPAO,catB7,qacE" 99 Good 1 1 0 1 1 1 1 0 0 1 0 1

Pseudomonas aeruginosa strain CF127 287 WGS CF127 NSOT01000000 40 6972917 cystic fibrosis patient United States "Human, Homo sapiens" Resistant Computational Prediction "crpP,fosA,catB7,blaPAO,blaOXA-396,aph(3')-Iib" 98.9 Good 1 1 1 1 1 1 1 0 0 0 0 1

Pseudomonas aeruginosa strain B9(T2436) strain B9(T2436) 287 WGS T2436 NSPL01000000 80 7150150 Sputum Thailand "Human, Homo sapiens" Resistant Computational Prediction "fosA,tet(g),aph(3'')-Ib,aadA1,aph(6)-Id,aph(3')-Iib,ant(4')-Iib,ant(2'')-Ia,crpP,ARR-2,blaOXA-488,blaVEB-2,blaPAO,blaOXA-10,qacE,catB7,cmlA1" 98.2 Good 1 1 1 1 1 1 1 0 0 1 0 1

Pseudomonas aeruginosa strain B3(T2101) strain B3(T2101) 287 WGS T2101 NSPN01000000 58 6919171 Sputum Thailand "Human, Homo sapiens" Resistant Computational Prediction "fosA,aph(3')-Iib,ant(2'')-Ia,aadA1,crpP,tet(G),blaVEB-1,blaPAO,blaCARB-2,blaOXA-50,blaOXA-10,qacE,catB7" 98.4 Good 1 1 0 1 1 1 1 0 1 1 0 1

Pseudomonas aeruginosa strain E9(4068) strain E9(4068) 287 WGS 4068 NSPH01000000 71 7068852 urine Thailand "Human, Homo sapiens" Resistant Computational Prediction "fosA,aac(6')-Ib-cr,aac(6')-Ib3,ant(2'')-Ia,aph(3')-Iib,crpP,sul1,blaIMP-54,blaPAO,blaOXA-10,blaOXA-50,qacE,catB7" 98.1 Good 1 1 1 1 1 1 1 0 0 1 0 1

Pseudomonas aeruginosa strain H3 287 WGS H3 NSQK01000000 42 6365570 sputum of adult cystic fibrosis patient United Kingdom "Human, Homo sapiens" Resistant Computational Prediction "fosA,catB7,blaPAO,blaOXA-494,blaOXA-396,aph(3')-Iib" 99.3 Good 1 1 1 1 1 1 1 0 0 0 0 0

Pseudomonas aeruginosa strain LiP3 287 WGS LiP3 NSQD01000000 37 6455927 sputum of adult cystic fibrosis patient United Kingdom "Human, Homo sapiens" Resistant Computational Prediction "fosA,catB7,blaPAO,blaOXA-50,aph(3')-Iib" 99.2 Good 1 1 0 1 1 1 1 0 0 0 0 0

Pseudomonas aeruginosa strain F2 287 WGS F2 NSQM01000000 70 6914814 urine of adult cystic fibrosis patient United Kingdom "Human, Homo sapiens" Resistant Computational Prediction "fosA,aac(6')-29a,aac(6')-29b,aph(3')-Iib,crpP,sul1,blaPAO,blaOXA-395,blaVIM-2,qacE,catB7" 98.7 Good 1 1 1 1 1 1 1 0 0 0 0 1

Pseudomonas aeruginosa strain E2_H133420224 287 WGS E2_H133420224 NSQN01000000 46 6994546 sputum of child cystic fibrosis patient United Kingdom "Human, Homo sapiens" Resistant Computational Prediction "crpP,fosA,catB7,blaPAO,blaOXA-396,aph(3')-Iib" 98.8 Good 1 1 1 1 1 1 1 0 0 0 0 1

Pseudomonas aeruginosa strain DUN-006 287 WGS DUN-006 NSRD01000000 44 6733675 cystic fibrosis patient New Zealand "Human, Homo sapiens" Resistant Computational Prediction "crpP,fosA,catB7,blaPAO,blaOXA-488,aph(3')-Iib" 99.1 Good 1 1 0 1 1 1 1 0 0 0 0 1

Pseudomonas aeruginosa strain CF5 287 WGS CF5 NSOV01000000 56 6890506 cystic fibrosis patient United States "Human, Homo sapiens" Resistant Computational Prediction "fosA,catB7,blaPAO,blaOXA-396,aph(3')-Iib" 98.7 Good 1 1 1 1 1 1 1 0 0 0 0 0

Pseudomonas aeruginosa strain C8(T3532) strain C8(T3532) 287 WGS T3532 NSPJ01000000 63 6912106 Sputum Thailand "Human, Homo sapiens" Resistant Computational Prediction "fosA,aadA6,aph(3')-Iib,sul1,crpP,blaPAO,blaOXA-488,qacE,catB7" 98.2 Good 1 1 0 1 1 1 1 0 0 1 0 1

Pseudomonas aeruginosa strain A3(H638) strain A3(H638) 287 WGS H638 NSPM01000000 73 7150152 Blood Thailand "Human, Homo sapiens" Resistant Computational Prediction "aadA1,aph(6)-Id,aph(3'')-Ib,ant(2'')-Ia,aph(3')-Iib,ant(4')-Iib,ARR-2,crpP,tet(G),blaPAO,blaVEB-2,blaOXA-10,catB7,qac,EcmlA1,fosA" 98.2 Good 1 1 0 1 1 1 1 0 1 0 0 1

Pseudomonas aeruginosa strain A1(A2448) strain A1(A2448) 287 WGS A2448 NSPO01000000 89 7092398 Urine Thailand "Human, Homo sapiens" Resistant Computational Prediction "rmtE,aadA1,aph(3'')-Ib,ant(2'')-Ia,aph(6)-Id,aph(3')-Iib,tet(G),fosA,crpP,aadA1,ARR-2,blaPAO,blaOXA-488,blaOXA-485,blaIMP-48,blaIMP-14,blaIMP-54,blaOXA-10,blaVEB-1,qacE,cmx,cmlA1" 98.9 Good 1 1 1 1 1 1 1 0 1 1 0 1

Pseudomonas aeruginosa strain C7(T3583) strain C7(T3583) 287 WGS T3583 NSPP01000000 58 6908303 Sputum Thailand "Human, Homo sapiens" Resistant Computational Prediction "fosA,aadA1,ant(2'')-Ia,aph(3')-Iib,crpP,blaPAO,blaOXA-50,blaOXA-10,blaVEB-1,blaCARB-2,catB7,qacE" 98 Good 1 1 0 1 1 1 1 0 1 1 0 1

Pseudomonas aeruginosa strain HB159 287 WGS HB159 NSOO01000000 83 7022734 adult male urine Portugal "Human, Homo sapiens" Resistant Computational Prediction "crpP,fosA,catB7,blaPAO,blaOXA-494,blaOXA-396,aph(3')-Iib" 99.2 Good 1 1 1 1 1 1 1 0 0 0 0 1

Pseudomonas aeruginosa strain S54485 287 WGS S54485 NSOR01000000 49 6864662 urinary tract infection United States "Human, Homo sapiens" Resistant Computational Prediction "crpP,aph(3')-Iib,catB7,blaPAO,blaOXA-488,qacE" 98.9 Good 1 1 0 1 1 1 1 0 0 1 0 1

Pseudomonas aeruginosa strain DUN-024-1 287 WGS DUN-024-1 NSQT01000000 37 6733997 cystic fibrosis patient New Zealand "Human, Homo sapiens" Resistant Computational Prediction "crpP,fosA,catB7,blaPAO,blaOXA-494,blaOXA-396,blaOXA-50aph(3')-Iib" 98.7 Good 1 1 1 1 1 1 1 0 0 0 0 1

Pseudomonas aeruginosa strain DUN-015A 287 WGS DUN-015A NSQW01000000 37 6843059 cystic fibrosis patient New Zealand "Human, Homo sapiens" Resistant Computational Prediction "fosA,catB7,blaPAO,blaOXA-494,blaOXA-396,aph(3')-Iib" 97.9 Good 1 1 1 1 1 1 1 0 0 0 0 0

Pseudomonas aeruginosa strain U454A 287 WGS U454A NSRR01000000 35 6290391 cystic fibrosis patient Australia "Human, Homo sapiens" Resistant Computational Prediction "fosA,catB7,blaPAO,blaOXA-494,blaOXA-396,blaOXA-50aph(3')-Iib" 99.1 Good 1 1 1 1 1 1 1 0 0 0 0 0

Pseudomonas aeruginosa strain U350 287 WGS U350 NSRY01000000 30 6246202 cystic fibrosis patient Australia "Human, Homo sapiens" Resistant Computational Prediction "fosA,catB7,blaPAO,blaOXA-50,aph(3')-Iib" 99.6 Good 1 1 0 1 1 1 1 0 0 0 0 0

Pseudomonas aeruginosa strain U0330A 287 WGS U0330A NSRZ01000000 29 6413097 cystic fibrosis patient Australia "Human, Homo sapiens" Resistant Computational Prediction "fosA,catB7,blaPAO,blaOXA-486,aph(3')-Iib" 98.9 Good 1 1 0 1 1 1 1 0 0 0 0 0

Pseudomonas aeruginosa strain U372 287 WGS U372 NSRX01000000 30 6211156 cystic fibrosis patient Australia "Human, Homo sapiens" Resistant Computational Prediction "fosA,catB7,blaPAO,blaOXA-50,aph(3')-Iib" 99.6 Good 1 1 0 1 1 1 1 0 0 0 0 0

Pseudomonas aeruginosa strain PMM38 287 WGS PMM38 NSSL01000000 80 6662434 nasopharynx South Korea "Human, Homo sapiens" Resistant Computational Prediction "fosA,catB7,blaPAO,blaOXA-395,aph(3')-Iib" 99 Good 1 1 0 1 1 1 1 0 0 1 0 0

Pseudomonas aeruginosa strain U0272B 287 WGS U0272B NSSE01000000 25 6207735 cystic fibrosis patient Australia "Human, Homo sapiens" Resistant Computational Prediction "fosA,catB7,blaPAO,blaOXA-50,aph(3')-Iib" 99.7 Good 1 1 0 1 1 1 1 0 0 0 0 0

Pseudomonas aeruginosa strain Aa249 287 WGS Aa249 NSSY01000000 72 7124686 burn Germany "Human, Homo sapiens" Resistant Computational Prediction "fosA,aph(3')-Iib,aac(6')-Ib-cr,aac(6')-Ib3,crpP,sul1,blaPAO,blaOXA-488,blaOXA-129qacE,catB7" 98 Good 1 1 0 1 1 1 1 0 0 0 0 1

Pseudomonas aeruginosa strain W5Aug16 287 WGS W5Aug16 NSTB01000000 55 6997822 river Belgium Resistant Computational Prediction "crpP,fosA,catB7,blaPAO,blaOXA-50,aph(3')-Iib" 98.3 Good 1 1 0 1 1 1 1 0 0 0 0 1

Pseudomonas aeruginosa strain Co380791 287 WGS Co380791 NSUH01000000 85 7290475 blood Colombia "Human, Homo sapiens" Resistant Computational Prediction "fosA,aac(6')-29b,aac(6')-29a,aph(3')-Iib,crpP,sul1,blaVIM-2,blaPAO,blaOXA-395,qacE,catB7," 98.7 Good 1 1 1 1 1 1 1 0 0 0 0 1

Pseudomonas aeruginosa strain U451 287 WGS U451 NSRS01000000 24 6207123 cystic fibrosis patient Australia "Human, Homo sapiens" Resistant Computational Prediction "fosA,catB7,blaPAO,blaOXA-50,aph(3')-Iib" 99.6 Good 1 1 0 1 1 1 1 0 0 0 0 0

Pseudomonas aeruginosa strain So098 287 WGS So098 NSVA01000000 65 7088127 wound Bulgaria "Human, Homo sapiens" Resistant Computational Prediction "fosA,aph(6)-Id,aph(3'')-Ib,aac(6')-Ib3,aph(3')-Ia,aph(3')-Iib,aac(6')-Ib-cr,aph(3')-XV,crpP,sul1,tet(G),blaOXA-161,blaOXA-2,blaOXA-541,blaOXA-141,blaOXA-210,blaOXA-415,blaOXA-539,blaOXA-15,blaOXA-396,blaOXA-226,blaOXA-541,blaOXA-494,blaOXA-229,blaOXA-210,qacE,catB7" 98.4 Good 1 1 1 1 1 1 1 0 1 0 0 1

Pseudomonas aeruginosa strain U397A 287 WGS U397A NSRW01000000 34 6211991 cystic fibrosis patient Australia "Human, Homo sapiens" Resistant Computational Prediction "fosA,catB7,blaPAO,blaOXA-50,aph(3')-Iib" 99.6 Good 1 1 0 1 1 1 1 0 0 0 0 0

Pseudomonas aeruginosa strain U0306 287 WGS U0306 NSSB01000000 42 6638608 cystic fibrosis patient Australia "Human, Homo sapiens" Resistant Computational Prediction "crpP,fosA,catB7,blaPAO,blaOXA-486,aph(3')-Iib" 98.6 Good 1 1 0 1 1 1 1 0 0 0 0 1

Pseudomonas aeruginosa strain LMG5031 287 WGS LMG5031 NSST01000000 100 6525186 Puerto Rico Aglaonema Resistant Computational Prediction crpP 97.6 Good 0 0 0 0 0 0 0 0 0 0 0 1

Pseudomonas aeruginosa strain LiA96_2004 287 WGS LiA96_2004 NSTC01000000 66 6623728 respiratory system Portugal Delphinidae Resistant Computational Prediction "fosA,catB7,blaPAO,blaOXA-395,aph(3')-Iib" 98.3 Good 1 1 1 1 1 1 1 0 0 1 0 0

Pseudomonas aeruginosa strain LiA50_2005 287 WGS LiA50_2005 NSTF01000000 63 6902557 ear Portugal "Dog, Canis lupus familiaris" Resistant Computational Prediction "crpP,fosA,catB7,blaPAO,blaOXA-396,aph(3')-Iib" 98.4 Good 1 1 1 1 1 1 1 0 0 1 0 1

Pseudomonas aeruginosa strain Jp1140 287 WGS Jp1140 NSTM01000000 82 6797755 Sea water (coastal) Japan Resistant Computational Prediction "crpP,fosA,catB7,blaPAO,blaOXA-395,aph(3')-Iib" 98.7 Good 1 1 1 1 1 1 1 0 0 0 0 1

Pseudomonas aeruginosa strain Jp224 287 WGS Jp224 NSTO01000000 94 7008188 Sea water (open ocean) Japan Resistant Computational Prediction "crpP,fosA,catB7,blaPAO,blaOXA-395,aph(3')-Iib" 98.7 Good 1 1 1 1 1 1 1 0 0 0 0 1

Pseudomonas aeruginosa strain PN586(35)w strain PN586(35)w strain PN586(35)w 287 WGS PN586(35)w NSVB01000000 76 7386855 catheter Panama "Human, Homo sapiens" Resistant Computational Prediction "fosA,aac(6')-29a,aac(6')-29b,aph(3')-Iib,crpP,sul1,blaPAO,blaOXA-395,blaVIM-2,qacE,catB7" 98.7 Good 1 1 1 1 1 1 0 0 0 0 0 1

Pseudomonas aeruginosa strain CND03 287 WGS CND03 NSUL01000000 75 6620172 wound Georgia "Human, Homo sapiens" Resistant Computational Prediction "fosA,catB7,blaPAO,blaOXA-488,aph(3')-Iib" 98.4 Good 1 1 0 1 1 1 1 0 0 1 0 0

Pseudomonas aeruginosa strain HPA16 287 WGS HPA16 NSVE01000000 63 7006712 non cystic fibrosis Italy "Human, Homo sapiens" Resistant Computational Prediction "fosA,aac(6')-Ib-cr,aph(3')-Iib,aac(6')-Ib3,aadA2b,crpP,blaCARB-2,blaPAO,blaOXA-395,qacE" 98.9 Good 1 1 1 1 1 1 1 0 0 0 0 1

Pseudomonas aeruginosa strain Co398373 287 WGS Co398373 NSVD01000000 72 7343076 Hospital environment Colombia Resistant Computational Prediction "fosA,aac(6')-29a,aac(6')-29b,aph(3')-Iib,crpP,sul1,blaPAO,blaOXA-395,blaVIM-2,qacE,catB7" 98.6 Good 1 1 1 1 1 1 1 0 0 0 0 1

Pseudomonas aeruginosa strain AMT0046-109 287 WGS AMT0046-109 NSVL01000000 25 6451066 sputum of cystic fibrosis patient United States "Human, Homo sapiens" Resistant Computational Prediction "fosA,catB7,blaPAO,blaOXA-50,aph(3')-Iib" 99 Good 1 1 0 1 1 1 0 0 0 0 0 0

Pseudomonas aeruginosa strain AMT0026-67 287 WGS AMT0026-67 NSVN01000000 47 6832062 sputum of cystic fibrosis patient United States "Human, Homo sapiens" Resistant Computational Prediction "crpP,fosA,catB7,blaPAO,blaOXA-396,aph(3')-Iib" 98.8 Good 1 1 1 1 1 1 1 0 0 0 0 1

Pseudomonas aeruginosa strain AMT0020-84 287 WGS AMT0020-84 NSVQ01000000 91 6713302 sputum of cystic fibrosis patient United States "Human, Homo sapiens" Resistant Computational Prediction "fosA,catB7,blaPAO,blaOXA-488,aph(3')-Iib" 98.7 Good 1 1 0 1 1 1 1 0 0 1 0 0

Pseudomonas aeruginosa strain AUS719 287 WGS AUS719 NSVV01000000 42 6244219 sputum of adult cystic fibrosis patient Australia "Human, Homo sapiens" Resistant Computational Prediction "fosA,catB7,blaPAO,blaOXA-395,aph(3')-Iib" 99.4 Good 1 1 1 1 1 1 1 0 0 0 0 0

Pseudomonas aeruginosa strain AUS307 287 WGS AUS307 NSVZ01000000 37 6769535 Bacteraemia patient Australia "Human, Homo sapiens" Resistant Computational Prediction "crpP,fosA,catB7,blaPAO,blaOXA-50,aph(3')-Iib" 98.4 Good 1 1 0 1 1 1 1 0 0 0 0 1

Pseudomonas aeruginosa strain AUS344 287 WGS AUS344 NSWC01000000 27 6371733 urinary tract infection Australia "Human, Homo sapiens" Resistant Computational Prediction "fosA,catB7,blaPAO,blaOXA-486,aph(3')-Iib" 99.2 Good 1 1 0 1 1 1 1 0 0 0 0 0

Pseudomonas aeruginosa strain AUS345 287 WGS AUS345 NSWB01000000 29 6443199 ear Australia "Dog, Canis lupus familiaris" Resistant Computational Prediction "crpP,fosA,catB7,blaPAO,blaOXA-486,aph(3')-Iib" 99 Good 1 1 0 1 1 1 1 0 0 0 0 1

Pseudomonas aeruginosa strain AUS457 287 WGS AUS457 NSWF01000000 88 7428696 ear Australia "Dog, Canis lupus familiaris" Resistant Computational Prediction "crpP,fosA,catB7,blaPAO,blaOXA-396,aph(3')-Iib" 97.9 Good 1 1 1 1 1 1 1 0 0 0 0 1

Pseudomonas aeruginosa strain AUS496 287 WGS AUS496 NSWW01000000 38 6362176 sputum of adult bronchiectasis patient Australia "Human, Homo sapiens" Resistant Computational Prediction "fosA,catB7,blaPAO,blaOXA-486,aph(3')-Iib" 98.8 Good 1 1 0 1 1 1 1 0 0 0 0 0

Pseudomonas aeruginosa strain AUS150 287 WGS AUS150 NSXG01000000 46 6853725 Bacteraemia patient Australia "Human, Homo sapiens" Resistant Computational Prediction "fosA,catB7,blaPAO,blaOXA-486,aph(3')-Iib" 98.1 Good 1 1 0 1 1 1 1 0 0 0 0 0

Pseudomonas aeruginosa strain AUS148 287 WGS AUS148 NSXI01000000 56 6301508 sputum of adult cystic fibrosis patient Australia "Human, Homo sapiens" Resistant Computational Prediction "fosA,catB7,blaPAO,blaOXA-486,aph(3')-Iib" 99.3 Good 1 1 0 1 1 1 1 0 0 0 0 0

Pseudomonas aeruginosa strain AUS154 287 WGS AUS154 NSXE01000000 26 6341366 wound Australia "Dog, Canis lupus familiaris" Resistant Computational Prediction "fosA,catB7,blaPAO,blaOXA-50,aph(3')-Iib" 99.3 Good 1 1 0 1 1 1 1 0 0 0 0 0

Pseudomonas aeruginosa strain AUS066 287 WGS AUS066 NSXW01000000 40 6222918 sputum of adult cystic fibrosis patient Australia "Human, Homo sapiens" Resistant Computational Prediction "fosA,catB7,blaPAO,blaOXA-494,blaOXA-396,aph(3')-Iib" 98.8 Good 1 1 1 1 1 1 1 0 0 0 0 0

Pseudomonas aeruginosa strain AUS502 287 WGS AUS502 NSZE01000000 59 7105329 River Australia Resistant Computational Prediction "crpP,fosA,catB7,blaPAO,blaOXA-396,aph(3')-Iib,aph(6)-Id,aph(3'')-Ib" 98.5 Good 1 1 1 1 1 1 1 0 0 0 0 1

Pseudomonas aeruginosa strain 982 287 WGS 982 NTAB01000000 40 6477730 urine United Kingdom "Dog, Canis lupus familiaris" Resistant Computational Prediction "crpP,fosA,catB7,blaPAO,blaOXA-486,aph(3')-Iib" 99.1 Good 1 1 0 1 1 1 1 0 0 0 0 1

Pseudomonas aeruginosa strain 903 287 WGS 903 NTAE01000000 44 6413757 urine United Kingdom "Horse, Equus caballus" Resistant Computational Prediction "fosA,catB7,blaPAO,blaOXA-486,aph(3')-Iib" 98.8 Good 1 1 0 1 1 1 1 0 0 0 0 0

Pseudomonas aeruginosa strain 1098 287 WGS 1098 NTAC01000000 63 7045350 vaginal mucosa United Kingdom "Dog, Canis lupus familiaris" Resistant Computational Prediction "crpP,fosA,catB7,blaPAO,blaOXA-488,aph(3')-Iib" 98.5 Good 1 1 0 1 1 1 1 0 0 1 0 1

Pseudomonas aeruginosa strain AUS440 287 WGS AUS440 NSWJ01000000 40 7003782 ear Australia "Human, Homo sapiens" Resistant Computational Prediction "crpP,fosA,catB7,blaPAO,blaOXA-396,aph(3')-Iib" 98.5 Good 1 1 1 1 1 1 1 0 0 1 0 1

Pseudomonas aeruginosa strain AUS021 287 WGS AUS021 NSWD01000000 23 6338835 sputum of adult cystic fibrosis patient Australia "Human, Homo sapiens" Resistant Computational Prediction "fosA,catB7,blaPAO,blaOXA-486,aph(3')-Iib" 99.3 Good 1 1 0 1 1 1 1 0 0 0 0 0

Pseudomonas aeruginosa strain AUS353 287 WGS AUS353 NSWT01000000 51 6450046 ear Australia "Dog, Canis lupus familiaris" Resistant Computational Prediction "fosA,catB7,blaPAO,blaOXA-494,blaOXA-396,aph(3')-Iib" 99.6 Good 1 1 1 1 1 1 1 0 0 0 0 0

Pseudomonas aeruginosa strain AUS491 287 WGS AUS491 NSWX01000000 34 6347473 sputum of adult bronchiectasis patient Australia "Human, Homo sapiens" Resistant Computational Prediction "fosA,catB7,blaPAO,blaOXA-50,aph(3')-Iib" 99 Good 1 1 0 1 1 1 1 0 0 0 0 0

Pseudomonas aeruginosa strain AUS306 287 WGS AUS306 NSYL01000000 38 6425832 River Australia Resistant Computational Prediction "fosA,catB7,blaPAO,blaOXA-50,aph(3')-Iib" 99.6 Good 1 1 0 1 1 1 1 0 0 0 0 0

Pseudomonas aeruginosa strain AUS460 287 WGS AUS460 NSYQ01000000 43 6505696 "bronchial mucosa, Respiratory infection" Australia Felidae Resistant Computational Prediction "fosA,catB7,blaPAO,blaOXA-494,blaOXA-396,aph(3')-Iib" 99.6 Good 1 1 1 1 1 1 1 0 0 0 0 0

Pseudomonas aeruginosa strain AUS343 287 WGS AUS343 NSYU01000000 38 6433806 River Australia Resistant Computational Prediction "crpP,fosA,catB7,blaPAO,blaOXA-486,aph(3')-Iib" 98.4 Good 1 1 0 1 1 1 1 0 0 0 0 1

Pseudomonas aeruginosa strain Mi162 287 WGS Mi162 NTAF01000000 61 7125908 burn United States "Human, Homo sapiens" Resistant Computational Prediction "fosA,aac(6')-Ib-cr,aadA2b,aac(6')-Ib3,sul1,aph(3')-Iib,crpP,blaPAO,blaOXA-395,blaCARB-2,qacE,catB7" 98.8 Good 1 1 1 1 1 1 1 0 0 0 0 1

Pseudomonas aeruginosa strain PA-W19 287 WGS PA-W19 NTAS01000000 32 6450047 skin United Kingdom "Human, Homo sapiens" Resistant Computational Prediction "fosA,catB7,blaPAO,blaOXA-50,aph(3')-Iib" 99.2 Good 1 1 0 1 1 1 1 0 0 0 0 0

Pseudomonas aeruginosa strain PA-W7 287 WGS PA-W7 NTBB01000000 68 7242167 skin United Kingdom "Human, Homo sapiens" Resistant Computational Prediction "crpP,fosA,catB7,blaPAO,blaOXA-488,aph(3')-Iib" 98.8 Good 1 1 0 1 1 1 1 0 0 0 0 1

Pseudomonas aeruginosa strain U0284 287 WGS U0284 NSSD01000000 23 6207090 cystic fibrosis patient Australia "Human, Homo sapiens" Resistant Computational Prediction "fosA,catB7,blaPAO,blaOXA-50,aph(3')-Iib" 99.5 Good 1 1 0 1 1 1 1 0 0 0 0 0

Pseudomonas aeruginosa strain 1663 287 WGS 1663 RRBU01000000 148 6354546 cystic fibrosis patient United Kingdom "Human, Homo sapiens" Resistant Computational Prediction "fosA,catB7,blaPAO,blaOXA-50,aph(3')-Iib" 98.7 Good 1 1 0 1 1 1 1 0 0 0 0 0

Pseudomonas aeruginosa strain 1689 287 WGS 1689 RRBX01000000 95 5820849 cystic fibrosis patient United Kingdom "Human, Homo sapiens" Resistant Computational Prediction "fosA,catB7,blaPAO,blaOXA-50,aph(3')-Iib" 97.9 Good 1 1 0 1 1 1 1 0 0 0 0 0

Pseudomonas aeruginosa strain 1658 287 WGS 1658 RRBT01000000 81 6431751 cystic fibrosis patient United Kingdom "Human, Homo sapiens" Resistant Computational Prediction "fosA,catB7,blaPAO,blaOXA-50,aph(3')-Iib" 99.1 Good 1 1 0 1 1 1 1 0 0 0 0 0

Pseudomonas aeruginosa strain 1622 287 WGS 1622 RRBN01000000 51 6285478 cystic fibrosis patient United Kingdom "Human, Homo sapiens" Resistant Computational Prediction "crpP,fosA,catB7,blaPAO,blaOXA-486,aph(3')-Iib" 98.8 Good 1 1 0 1 1 1 1 0 0 0 0 0

Pseudomonas aeruginosa strain 1608 287 WGS 1608 RRBJ01000000 128 6278206 cystic fibrosis patient United Kingdom "Human, Homo sapiens" Resistant Computational Prediction "crpP,fosA,catB7,blaPAO,blaOXA-486,aph(3')-Iib" 98 Good 1 1 0 1 1 1 1 0 0 0 0 0

Pseudomonas aeruginosa strain 1472 287 WGS 1472 RRBF01000000 313 6981537 cystic fibrosis patient United Kingdom "Human, Homo sapiens" Resistant Computational Prediction "crpP,fosA,catB7,blaPAO,blaOXA-494,blaOXA-396,aph(3')-Iib" 96.5 Good 1 1 1 1 1 1 1 0 0 0 0 1

Pseudomonas aeruginosa strain 1664 287 WGS 1664 RRBV01000000 114 6355238 cystic fibrosis patient United Kingdom "Human, Homo sapiens" Resistant Computational Prediction "fosA,catB7,blaPAO,blaOXA-50,aph(3')-Iib" 98.8 Good 1 1 0 1 1 1 1 0 0 0 0 0

Pseudomonas aeruginosa strain 1631 287 WGS 1631 RRBO01000000 78 6426292 cystic fibrosis patient United Kingdom "Human, Homo sapiens" Resistant Computational Prediction "crpP,fosA,catB7,blaPAO,blaOXA-486,aph(3')-Iib" 98.2 Good 1 1 0 1 1 1 1 0 0 0 0 1

Pseudomonas aeruginosa strain 1311 287 WGS 1311 RRBC01000000 142 6347873 cystic fibrosis patient United Kingdom "Human, Homo sapiens" Resistant Computational Prediction "fosA,catB7,blaPAO,blaOXA-486,aph(3')-Iib" 98.2 Good 1 1 0 1 1 1 1 0 0 0 0 0

Pseudomonas aeruginosa strain PA-81 287 WGS PA-81 QXJN01000000 87 6725297 tracheal secretions Pakistan "Human, Homo sapiens" Resistant Computational Prediction "fosA,ant(2'')-Ia,aph(3')-Iib,aac(6')-Il,aph(3')-Via,aadA1,crpP,sul1,dfrB2,blaNDM-1,blaOXA-50,blaOXA-10,blaPAO,catB7,qacE" 98.7 Good 1 1 1 1 1 1 1 0 0 1 1 1

Pseudomonas aeruginosa strain PA_HTX2 287 WGS PA_HTX2 QVIR01000000 87 6907917 blood United States "Human, Homo sapiens" Resistant Computational Prediction "fosA,aadA1b,aac(6')-33,aph(3')-Iib,sul1,tet(G),blaGES-1,blaGES-19,blaGES-20,blaOXA-50,blaOXA-2,blaPAO,qacE,catB7" 98.8 Good 1 1 0 1 1 1 1 0 1 1 0 0

Pseudomonas aeruginosa strain PA_HTX1 287 WGS PA_HTX1 QVIQ01000000 127 6929324 blood United States "Human, Homo sapiens" Resistant Computational Prediction "aadA1b,aac(6')-Ib3,aph(3')-Iib,aac(6')-Ib-cr,aac(6')-33,fosA,sul1,tet(G),blaGES-19,blaPAO,blaOXA-2,blaOXA-50,catB7,qacE" 98.8 Good 1 1 0 1 1 1 1 0 1 1 0 1

Pseudomonas aeruginosa strain PA_180 287 WGS PA_180 RHSL01000000 55 6248492 bedside light switch in hospital intensive care unit Pakistan Susceptible;Intermediate AMR Panel "fosA,catB7,blaPAO,blaOXA-50,aph(3')-Iib" 99.5 Good 1 1 0 1 1 1 1 0 0 0 0 0

Pseudomonas aeruginosa strain PA_112 287 WGS PA_112 RHSS01000000 60 6247134 nursing call button in hospital intensive care unit Pakistan Susceptible;Resistant AMR Panel "fosA,catB7,blaPAO,blaOXA-50,aph(3')-Iib" 99.3 Good 1 1 0 1 1 1 1 0 0 0 0 0

Pseudomonas aeruginosa strain PA_038 287 WGS PA_038 RHSZ01000000 151 7029502 nursing call button in hospital intensive care unit Pakistan Resistant;Intermediate;Susceptible AMR Panel "rmtF,aac(6')-Ib-Hangzhou,aac(6')-Ib-cr,aph(3')-Iib,fosA,crpP,sul1,tet(G),blaPAO,blaOXA-50,blaOXA-10,blaOXA-4,blaOXA-1,catB7,qacE" 98.8 Good 1 1 0 1 1 1 1 0 1 1 0 1

Pseudomonas aeruginosa strain PA_254 287 WGS PA_254 RHSF01000000 120 7026352 alcohol foam dispenser in hospital intensive care unit Pakistan Resistant;Susceptible;Intermediate AMR Panel "aadA10,aac(6')-Ib3,aph(3')-XV,aph(3')-Iib,aac(6')-Ib-cr,fosA,crpP,tet(G),blaGES-1,blaPAO,blaOXA-488,qacE,catB7" 98 Good 1 1 0 1 1 1 1 0 1 1 0 1

Pseudomonas aeruginosa strain PA_187 287 WGS PA_187 RHSI01000000 131 6882642 washroom sink in hospital intensive care unit Pakistan Resistant;Intermediate;Susceptible AMR Panel "fosA,aac(6')-Ib-cr,aph(3')-Iib,aph(3')-XV,aac(6')-Ib3,aadA6,crpP,tet(G),blaPAO,blaOXA-488,blaGES-5,qacE,catB7" 98.7 Good 1 1 0 1 1 1 1 0 1 1 0 1

Pseudomonas aeruginosa strain PA_011 287 WGS PA_011 RHTB01000000 218 7054341 nursing call button in hospital intensive care unit Pakistan Resistant;Susceptible AMR Panel "aadA10,aac(6')-Ib3,aph(3')-XV,aph(3')-Iib,aac(6')-Ib-cr,fosA,crpP,tet(G),blaGES-1,blaPAO,blaOXA-488,qacE,catB7" 97.4 Good 1 1 0 1 1 1 1 0 1 1 0 1

Pseudomonas aeruginosa strain PA_041 287 WGS PA_041 RHSY01000000 134 7031211 bedside rail in hospital intensive care unit Pakistan Resistant;Intermediate;Susceptible AMR Panel "rmtF,aac(6')-Ib-Hangzhou,aac(6')-Ib-cr,aph(3')-Iib,fosA,crpP,sul1,tet(G),blaPAO,blaOXA-50,blaOXA-10,blaOXA-4,blaOXA-1,catB7,qacE" 98.9 Good 1 1 0 1 1 1 1 0 1 1 0 1

Pseudomonas aeruginosa strain FDAARGOS_502 strain Not applicable 287 WGS Not applicable RKJI01000000 1 7087687 "Human, Homo sapiens" Resistant Computational Prediction "aadA1b,aac(6')-Ib3,aac(6')-Ib-cr,aph(3')-Iib,fosA,sul1,crpP,blaPAO,blaOXA-50,blaOXA-2,qacE,catB7" 98.6 Good 1 1 0 1 1 1 1 0 0 0 0 1

Pseudomonas aeruginosa strain PA_304 287 WGS PA_304 RHRZ01000000 84 6710039 nursing call button in hospital intensive care unit Pakistan Resistant;Susceptible;Intermediate AMR Panel "fosA,aac(6')-Ib-cr,aph(3')-Iib,aph(3')-XV,aac(6')-Ib3,aadA6,crpP,tet(G),blaPAO,blaOXA-488,blaGES-5,qacE,catB7" 98.4 Good 1 1 0 1 1 1 1 0 1 1 0 1

Pseudomonas aeruginosa strain PA_294 287 WGS PA_294 RHSB01000000 92 6894293 washroom sink in hospital intensive care unit Pakistan Resistant;Susceptible;Intermediate AMR Panel "fosA,aac(6')-Ib-cr,aph(3')-Iib,aph(3')-XV,aac(6')-Ib3,aadA6,crpP,tet(G),blaPAO,blaOXA-488,blaGES-5,qacE,catB7" 98.7 Good 1 1 0 1 1 1 1 0 1 1 0 1

Pseudomonas aeruginosa strain PA_032 287 WGS PA_032 RHTA01000000 136 6793955 washroom sink in hospital intensive care unit Pakistan Susceptible;Resistant;Intermediate AMR Panel "fosA,aadA6,aph(3')-Iib,aac(6')-Ib3,aac(6')-Ib-cr,sul1,crpP,tet(A),ARR-3,blaOXA-488,blaPAO,blaCTX-M-3,blaTEM-1B,catB7" 98.4 Good 1 1 0 1 1 1 1 0 1 1 0 1

Pseudomonas aeruginosa strain IMP-13 287 Complete IMP-13 "CP034354,CP034355" 2 7178010 urine Belgium "Human, Homo sapiens" Resistant Computational Prediction "fosA,aph(3')-Via,aph(3')-Iib,aac(6')-Ib3,aac(6')-Ib-cr,crpP,sul1,blaPAO,blaOXA_50,blaIMP-13,qacE,catB7" 99 Good 1 1 1 1 1 1 1 0 0 1 1 1

Pseudomonas aeruginosa strain B41226 287 Complete B41226 CP034368 1 6814295 blood India "Human, Homo sapiens" Resistant Computational Prediction "aadA1,aac(6')-Il,aph(3')-VI,aph(3')-Iib,ant(2'')-Ia,tet(A),sul1,dfrB2,crpP,ARR-3,blaPAO,blaOXA-10,blaNDM-1,blaOXA-50,blaVEB-1,qacE,fosA,catB7,cmlA1" 95.8 Good 1 1 1 1 1 1 1 0 1 1 0 1

Pseudomonas aeruginosa strain SP4371 287 Complete SP4371 CP034369 1 6937609 sputum India "Human, Homo sapiens" Resistant Computational Prediction "fosA,msr€,ant(2'')-Ia,aac(6')-Il,aph(3')-VI,aph(3')-Iib,aadA1,sul1,dfrB2,crpP,tet(A),blaNDM-1,blaVEB-1,blaPAO,blaOXA-50,blaOXA-10,blaPME-1,qacE,catB7" 96.3 Good 1 1 1 1 1 1 0 1 0 1 0 1

Pseudomonas aeruginosa strain SP2230 287 Complete SP2230 CP034434 1 6976603 sputum India "Human, Homo sapiens" Resistant Computational Prediction "sul1,aph(3')-Iib,aph(3'')-Ib,aph(6)-Id,catB7,blaPAO,blaOXA-50,fosA" 96.5 Good 1 1 0 1 1 1 1 0 0 0 0 0

Pseudomonas aeruginosa strain B14130 287 Complete B14130 CP034435 1 6759594 blood India "Human, Homo sapiens" Resistant Computational Prediction "fosA,aph(3')-Iib,aadA1,crpP,sul1,dfrB2,tet(A),blaPAO,blaVEB-1,blaOXA-10,blaOXA-50,qacE,catB7" 97 Good 1 1 0 1 1 1 1 0 1 0 0 1

Pseudomonas aeruginosa strain B17932 287 Complete B17932 CP034436 1 6744658 blood India "Human, Homo sapiens" Resistant Computational Prediction "aadA1,aac(6')-Il,aph(3')-Iib,ant(2'')-Ia,fosA,sul1,dfrB2,crpP,tet(A),blaPAO,blaOXA-50,blaOXA-10,blaOXA-233,blaVEB-1,qacE,catB7" 97.6 Good 1 1 0 1 1 1 1 0 1 1 0 1

Pseudomonas aeruginosa strain R997 287 WGS R997 RWWN01000000 229 6948564 Resistant Computational Prediction "fosA,aph(6)-Id,aph(3')-Iib,aph(3'')-Ib,crpP,sul1,catB7,blaPAO,blaOXA-50" 98.4 Good 1 1 0 1 1 1 1 0 0 0 0 1

Pseudomonas aeruginosa strain R4671 287 WGS R4671 RWWL01000000 181 6777889 Resistant Computational Prediction "fosA,aph(6)-Id,aph(3'')-Ib,aph(3'')-Ib,blaPAO,blaOXA-396," 98.2 Good 1 1 1 1 1 1 0 0 0 0 0 0

Pseudomonas aeruginosa strain U3484 287 WGS U3484 RWWM01000000 147 6870810 Resistant Computational Prediction "rmtB,aac(6')-Ib-Hangzhou,aph(3')-Iib,aac(6')-Ib-cr,ARR-2,tet(G),fosA,sul1,qnrVC1,aadA10,blaPAO,blaOXA-1,blaOXA-4,blaOX-395,blaNDM-1,qacE,catB7" 98.6 Good 1 1 1 1 1 1 1 0 1 1 0 1

Pseudomonas aeruginosa strain R3870 287 WGS R3870 RWWO01000000 226 7033918 Resistant Computational Prediction "fosA,aph(3')-Iib,aac(6')-31,ant(2'')-Ia,crpP,sul1,blaPAO,blaOXA-1,blaOXA-395,catB7,cmx" 98.5 Good 1 1 1 1 1 1 1 0 0 1 0 1

Pseudomonas aeruginosa strain U1849 287 WGS U1849 RWWP01000000 146 6827334 Resistant Computational Prediction "fosA,tet(G)aadA10,aac(6')-Ib-cr,rmtB,aac(6')-Ib-Hangzhou,qnrVC1,sul1,ARR-2,blaNDM-1,blaPAO,blaOXA-1,blaOXA-4,blaOXA-395,qacE,catB7" 98.5 Good 1 1 1 1 1 1 1 0 1 1 0 1

Pseudomonas aeruginosa strain R4730 287 WGS R4730 RWWK01000000 235 6929080 Resistant Computational Prediction "fosA,aph(3')-Iib,aph(6)-Id,aph(3'')-Ib,crpP,sul1,blaPAO,blaOXA-50,catB7" 98.1 Good 1 1 0 1 1 1 1 0 0 0 0 1

Pseudomonas aeruginosa strain R486 287 WGS R486 RWWU01000000 196 7061901 Resistant Computational Prediction "fosA,fosG,aac(6')-Ib-cr,aph(3')-Iib,aac(6')-Ib-Hangzhou,crpP,sul1,blaPAO,blaOXA-494,blaOXA-396,blaOXA-2,catB7,cmx,qacE" 98.8 Good 1 1 1 1 1 1 1 0 0 0 0 1

Pseudomonas aeruginosa strain R3890 287 WGS R3890 RWXR01000000 158 6853219 Resistant Computational Prediction "crpP,fosA,catB7,blaPAO,blaOXA-488,aph(3')-Iib" 96.9 Good 1 1 0 1 1 1 1 0 0 1 0 1

Pseudomonas aeruginosa strain R4656 287 WGS R4656 RWXM01000000 132 6909135 Resistant Computational Prediction "fosA,catB7,blaPAO,blaOXA-395,aph(3')-Iib" 97.3 Good 1 1 1 0 1 1 1 0 0 1 0 0

Pseudomonas aeruginosa strain R4537 287 WGS R4537 RWWR01000000 208 6974001 Resistant Computational Prediction "crpP,fosA,catB7,blaPAO,blaOXA-395,aph(3')-Iib" 96.7 Good 1 1 1 1 1 1 1 0 0 1 0 1

Pseudomonas aeruginosa strain R4393 287 WGS R4393 RWWS01000000 227 7144488 Resistant Computational Prediction "crpP,fosA,catB7,blaPAO,blaOXA-494,blaOXA-396,aph(3')-Iib" 98.9 Good 1 1 1 1 1 1 1 0 0 0 0 1

Pseudomonas aeruginosa strain R2483 287 WGS R2483 RWXP01000000 226 6972514 Resistant Computational Prediction "sul1,aph(3')-Iib,aph(3'')-Ib,aph(6)-Id,catB7,blaPAO,blaOXA-50,fosA" 98.4 Good 1 1 0 1 1 1 1 0 0 0 0 0

Pseudomonas aeruginosa strain B1486 287 WGS B1486 RWWV01000000 169 6910289 Resistant Computational Prediction "fosA,catB7,blaPAO,blaOXA-395,aph(3')-Iib" 97.3 Good 1 1 1 1 1 1 1 0 0 1 0 0

Pseudomonas aeruginosa strain R2637 287 WGS R2637 RWXN01000000 142 6925597 Resistant Computational Prediction "fosA,aph(6)-Id,aac(6')-Ib-cr,aph(3'')-Ib,aph(3')-Iib,aac(6')-Ib-Hangzhou,aph(3')-Ia,crpP,sul1,blaPAO,blaOXA-494,blaOXA-396,blaOXA-2,qacE,catB7,catB3" 98.2 Good 1 1 1 1 1 1 1 0 0 0 0 1

Pseudomonas aeruginosa strain R4643 287 WGS R4643 RWWZ01000000 221 6972192 Resistant Computational Prediction "crpP,fosA,catB7,blaPAO,blaOXA-494,blaOXA-396,aph(3')-Iib" 98.5 Good 1 1 1 1 1 1 1 0 0 0 0 1

Pseudomonas aeruginosa strain TUM17825 287 WGS TUM17825 BIFN01000000 219 6881717 Vietnamese medical tourist Japan "Human, Homo sapiens" Resistant Computational Prediction "rmtB,aph(3')-Iib,aac(3)-Id,aac(6')-Il,aac(6')-Iia,fosA,crpP,qnrVC1,dfrB5,sul1,tet(G),blaPAO,blaNDM-1,blaOXA-486,qacE,catB7" 97.6 Good 1 1 1 1 1 1 1 0 1 1 0 1

Pseudomonas aeruginosa strain TUM17826 287 WGS TUM17826 BIFO01000000 193 6924888 Vietnamese medical tourist Japan "Human, Homo sapiens" Resistant Computational Prediction "fosA,aac(6')-Il,aac(6')-Iia,aph(3')-Iib,aac(3)-Id,aph(3')-Via,rmtB,crpP,qnrVC1,dfrB5,sul1,catB7,blaPAO,tet(G),blaOXA-486,blaNDM-1,qacE" 98.1 Good 1 1 1 1 1 1 1 0 1 0 1 1

Pseudomonas aeruginosa strain MRSN8914 287 WGS MRSN8914 RXTB01000000 315 7356800 bone United States "Human, Homo sapiens" Intermediate;Resistant AMR Panel "fosA,aadA1,aph(3')-Iib,aac(6')-Ib3,ant(2'')-Ia,aac(6')-Ib-cr,sul1,crpP,blaPAO,blaOXA-10,blaOXA-395,qacE,catB7" 97 Good 1 1 1 1 1 1 1 0 0 1 0 1

Pseudomonas aeruginosa strain MRSN8915 287 WGS MRSN8915 RXTA01000000 329 7064713 urine United States "Human, Homo sapiens" Susceptible;Resistant AMR Panel "aadA2b,aac(6')-Ib-cr,aph(3')-Iib,aac(6')-Ib3,fosA,sul1,crpP,blaOXA-395,blaPAO,blaCARB-2,qacE" 97.9 Good 1 1 1 1 1 1 1 0 0 0 0 1

Pseudomonas aeruginosa strain MRSN994 287 WGS MRSN994 RXSX01000000 57 6734108 respiratory United States "Human, Homo sapiens" Susceptible;Resistant AMR Panel "aadA6,aph(3')-Iib,sul1,crpP,sul1,blaPAO,blaOXA-494,blaOXA-396,qacE,catB7," 98.8 Good 1 1 1 1 1 1 1 0 0 0 0 1

Pseudomonas aeruginosa strain MRSN8139 287 WGS MRSN8139 RXTE01000000 223 6575059 wound United States "Human, Homo sapiens" Susceptible;Resistant;Intermediate AMR Panel "crpP,fosA,catB7,blaPAO,blaOXA-395,aph(3')-Iib" 97.5 Good 1 1 1 1 1 1 1 0 0 0 0 1

Pseudomonas aeruginosa strain MRSN7014 287 WGS MRSN7014 RXTH01000000 57 6420453 respiratory United States "Human, Homo sapiens" Susceptible;Intermediate;Resistant AMR Panel "fosA,catB7,blaPAO,blaOXA-494,blaOXA-396,aph(3')-Iib" 98.7 Good 1 1 1 1 1 1 1 0 0 0 0 0

Pseudomonas aeruginosa strain MRSN8912 287 WGS MRSN8912 RXTC01000000 132 6986301 urine United States "Human, Homo sapiens" Susceptible;Resistant AMR Panel "fosA,v,aac(6')-Ib-Hangzhou,aadA1,aac(6')-Ib-cr,crpP,sul1,blaPAO,blaOXA-10,blaOXA-395.cmlA1,catB7,qacE" 98.8 Good 1 1 1 1 1 1 1 0 0 0 0 1

Pseudomonas aeruginosa strain MRSN8136 287 WGS MRSN8136 RXTF01000000 96 6907900 wound United States "Human, Homo sapiens" Susceptible;Resistant;Intermediate AMR Panel "fosA,aph(3')-Iib,aadA7,sul1,blaPAO,blaOXA-50,catB7,qacE" 98.8 Good 1 1 0 1 1 1 1 0 0 0 0 0

Pseudomonas aeruginosa strain MRSN6695 287 WGS MRSN6695 RXTJ01000000 55 6235816 urine United States "Human, Homo sapiens" Susceptible;Resistant AMR Panel "fosA,catB7,blaPAO,blaOXA-494,blaOXA-50,aph(3')-Iib" 99.1 Good 1 1 0 1 1 1 1 0 0 0 0 0

Pseudomonas aeruginosa strain MRSN6241 287 WGS MRSN6241 RXTL01000000 135 7161217 wound United States "Human, Homo sapiens" Susceptible;Resistant AMR Panel "aadA6,aph(3'')-Ib,aph(6)-Id,aph(3')-Vib,aph(3')-XV,aac(6')-Ib-cr,aac(6')-Ib3,sul1,crpP,blaPER-1,blaOXA-2,qacE," 97 Good 1 1 0 1 1 1 1 0 0 1 1 1

Pseudomonas aeruginosa strain MRSN6678 287 WGS MRSN6678 RXTK01000000 127 6782774 sterile tissue United States "Human, Homo sapiens" Susceptible;Resistant AMR Panel "fosA,aph(3')-Iib,aac(6')-Ib3,aac(6')-Ib-cr,aadA6,sul1,tet(G),blaPAO,blaOXA-2blaOXA-488,qacE" 99 Good 1 1 0 1 1 1 1 0 1 1 0 1

Pseudomonas aeruginosa strain MRSN6220 287 WGS MRSN6220 RXTM01000000 413 6910198 wound United States "Human, Homo sapiens" Resistant AMR Panel "fosA,aac(6')-Ib3,aac(6')-Ib-cr,aph(3'')-Ib,aph(6)-Id,aph(3')-Iib,crpP,sul1,blaOXA-10,blaPAO,blaOXA-396,blaOXA-494,blaVIM-6,qacE,cmx,catB7" 98.7 Good 1 1 1 1 1 1 1 0 0 0 0 1

Pseudomonas aeruginosa strain MRSN5524 287 WGS MRSN5524 RXTO01000000 151 6725073 urine United States "Human, Homo sapiens" Susceptible;Resistant;Intermediate AMR Panel "aadA6,ant(2'')-Ia,aph(3')-Iib,fosA,sul1,tet(A),blaPAO,blaOXA-488,qacE,blaOXA-392,catB7," 98.7 Good 1 1 0 1 1 1 1 0 1 1 0 0

Pseudomonas aeruginosa strain MRSN5539 287 WGS MRSN5539 RXTN01000000 113 6769662 sterile tissue United States "Human, Homo sapiens" Susceptible;Resistant AMR Panel "fosA,aac(3)-Ic,aph(3')-Iib,sul1,blaOXA-488,blaPAO,qacE,catB7" 98.5 Good 1 1 0 1 1 1 1 0 0 1 0 0

Pseudomonas aeruginosa strain MRSN552 287 WGS MRSN552 RXTP01000000 54 6444919 urine United States "Human, Homo sapiens" Susceptible;Resistant AMR Panel "crpP,fosA,catB7,blaPAO,blaOXA-486,aph(3')-Iib" 99 Good 1 1 0 1 1 1 1 0 0 0 0 1

Pseudomonas aeruginosa strain MRSN5519 287 WGS MRSN5519 RXTQ01000000 150 6751302 wound United States "Human, Homo sapiens" Resistant AMR Panel "fosA,aph(3')-Iib,aac(6')-Il,aac(6')-31,aac(6')-Ib3,aac(6')-Ib-craadA6,aadA2b,sul1,blaPAO,blaOXA-2,blaOXA-488,qacE,catB7" 98.7 Good 1 1 0 1 1 1 1 0 0 1 0 1

Pseudomonas aeruginosa strain MRSN4841 287 WGS MRSN4841 RXTT01000000 124 6475204 urine United States "Human, Homo sapiens" Susceptible;Intermediate;Resistant AMR Panel "aadA6,aph(3')-Iib,fosA,sul1,crpP,blaPAO,blaOXA-488,qacE,catB7" 98.7 Good 1 1 0 1 1 1 1 0 0 1 0 1

Pseudomonas aeruginosa strain MRSN5498 287 WGS MRSN5498 RXTS01000000 95 6821230 sterile tissue United States "Human, Homo sapiens" Susceptible;Resistant AMR Panel "fosA,aadA1,aac(6')-Ib-cr,aac(6')-Ib-Hangzhou,aph(3')-Iib,crpP,sul1,blaPAO,blaOXA-395,blaOXA-10,qacE,catB7,cmlA1" 99.3 Good 1 1 1 1 1 1 1 0 0 0 0 1

Pseudomonas aeruginosa strain MRSN315 287 WGS MRSN315 RXUI01000000 58 6696919 respiratory United States "Human, Homo sapiens" Susceptible;Intermediate AMR Panel "crpP,fosA,catB7,blaPAO,blaOXA-396,aph(3')-Iib" 99 Good 1 1 1 1 1 1 1 0 0 0 0 1

Pseudomonas aeruginosa strain MRSN19711 287 WGS MRSN19711 RXUX01000000 179 6265327 respiratory United States "Human, Homo sapiens" Susceptible;Resistant;Intermediate AMR Panel "fosA,catB7,blaPAO,blaOXA-488,aph(3')-Iib" 98.8 Good 1 1 0 1 1 1 1 0 0 0 0 0

Pseudomonas aeruginosa strain MRSN20190 287 WGS MRSN20190 RXUV01000000 113 6191883 respiratory United States "Human, Homo sapiens" Resistant;Susceptible AMR Panel "fosA,catB7,blaPAO,blaOXA-494,blaOXA-396,aph(3')-Iib" 98.5 Good 1 1 1 1 1 1 1 0 0 0 0 0

Pseudomonas aeruginosa strain MRSN9873 287 WGS MRSN9873 RXSY01000000 192 7146707 urine United States "Human, Homo sapiens" Susceptible;Resistant AMR Panel "fosA,aph(3')-Iib,aac(6')-Ib3,aac(6')-Ib-cr,crpP,blaPAO,blaOXA-486,qacE,catB7" 97.4 Good 1 1 0 1 1 1 1 0 0 0 0 1

Pseudomonas aeruginosa strain MRSN9718 287 WGS MRSN9718 RXSZ01000000 300 6861353 urine United States "Human, Homo sapiens" Susceptible;Intermediate;Resistant AMR Panel "crpP,fosA,catB7,blaPAO,blaOXA-494,blaOXA-396,aph(3')-Iib" 97.5 Good 1 1 1 1 1 1 1 0 0 0 0 1

Pseudomonas aeruginosa strain MRSN8141 287 WGS MRSN8141 RXTD01000000 139 7161718 respiratory United States "Human, Homo sapiens" Susceptible;Resistant AMR Panel "aadA6,,aph(3'')-Ib,aph(6)-Id,aac(6')-Ib3,aph(3')-XV,aac(6')-Ib-cr,sul1,crpP,blaPER-1,blaOXA-2,qacE" 97 Good 1 1 0 1 1 1 1 0 0 1 1 1

Pseudomonas aeruginosa strain MRSN8130 287 WGS MRSN8130 RXTG01000000 250 6828028 blood United States "Human, Homo sapiens" Susceptible;Resistant AMR Panel "crpP,aac(6')-Ib-cr,aph(3')-Iib,blaOXA-395,catB7,blaOXA-1,blaPAO,fosA" 97.7 Good 1 1 1 1 1 1 1 0 0 1 0 1

Pseudomonas aeruginosa strain MRSN6739 287 WGS MRSN6739 RXTI01000000 67 6298064 urine United States "Human, Homo sapiens" Susceptible;Intermediate AMR Panel "fosA,catB7,blaPAO,blaOXA-50,aph(3')-Iib" 99.3 Good 1 1 0 1 1 1 1 0 0 0 0 0

Pseudomonas aeruginosa strain MRSN5508 287 WGS MRSN5508 RXTR01000000 223 6439859 sterile body fluid United States "Human, Homo sapiens" Susceptible;Resistant;Intermediate AMR Panel "crpP,fosA,catB7,blaPAO,blaOXA-50,aph(3')-Iib" 98.2 Good 1 1 0 1 1 1 1 0 0 0 0 1

Pseudomonas aeruginosa strain MRSN435288 287 WGS MRSN435288 RXTW01000000 161 6363888 respiratory United States "Human, Homo sapiens" Susceptible;Intermediate;Resistant AMR Panel "crpP,fosA,catB7,blaPAO,blaOXA-494,blaOXA-396,blaOXA-50,aph(3')-Iib" 98.4 Good 1 1 1 1 1 1 1 0 0 0 0 1

Pseudomonas aeruginosa strain MRSN321 287 WGS MRSN321 RXUG01000000 50 6260875 wound United States "Human, Homo sapiens" Susceptible;Resistant AMR Panel "fosA,catB7,blaPAO,blaOXA-396,aph(3')-Iib" 99.7 Good 1 1 1 1 1 1 1 0 0 0 0 0

Pseudomonas aeruginosa strain MRSN25678 287 WGS MRSN25678 RXUN01000000 299 6509751 urine United States "Human, Homo sapiens" Susceptible;Intermediate;Resistant AMR Panel "fosA,catB7,blaPAO,blaOXA-50,aph(3')-Iib" 98.1 Good 1 1 0 1 1 1 1 0 0 0 0 0

Pseudomonas aeruginosa strain MRSN390231 287 WGS MRSN390231 RXTZ01000000 42 6149846 respiratory United States "Human, Homo sapiens" Susceptible;Intermediate AMR Panel "fosA,catB7,blaPAO,blaOXA-488,aph(3')-Iib" 99.2 Good 1 1 0 1 1 1 1 0 0 0 0 0

Pseudomonas aeruginosa strain MRSN436311 287 WGS MRSN436311 RXTV01000000 52 6473360 urine United States "Human, Homo sapiens" Susceptible;Resistant;Intermediate AMR Panel "crpP,fosA,catB7,blaPAO,blaOXA-395,aph(3')-Iib" 98.7 Good 1 1 1 1 1 1 1 0 0 1 0 1

Pseudomonas aeruginosa strain MRSN373401 287 WGS MRSN373401 RXUA01000000 75 6985982 urine United States "Human, Homo sapiens" Susceptible;Resistant;Intermediate AMR Panel "crpP,fosA,catB7,blaPAO,blaOXA-486,aph(3')-Iib" 98.7 Good 1 1 0 1 1 1 1 0 0 0 0 1

Pseudomonas aeruginosa strain MRSN3705 287 WGS MRSN3705 RXUB01000000 234 6419743 respiratory Guam "Human, Homo sapiens" Susceptible;Resistant AMR Panel crpP 97.2 Good 0 0 0 0 0 0 0 0 0 0 0 1

Pseudomonas aeruginosa strain MRSN29192 287 WGS MRSN29192 RXUK01000000 52 6375161 urine United States "Human, Homo sapiens" Susceptible;Resistant;Intermediate AMR Panel "fosA,catB7,blaPAO,blaOXA-488,blaOXA-485,aph(3')-Iib" 99.4 Good 1 1 0 1 1 1 1 0 0 0 0 0

Pseudomonas aeruginosa strain MRSN25762 287 WGS MRSN25762 RXUM01000000 72 6845305 respiratory United States "Human, Homo sapiens" Susceptible;Resistant AMR Panel "crpP,fosA,catB7,blaPAO,blaOXA-50,aph(3')-Iib" 98.8 Good 1 1 0 1 1 1 1 0 0 0 0 1

Pseudomonas aeruginosa strain MRSN2444 287 WGS MRSN2444 RXUP01000000 275 6843047 respiratory United States "Human, Homo sapiens" Susceptible;Resistant;Intermediate AMR Panel "fosA,ant(2'')-Ia,aph(6)-Id,aph(3')-Iib,aph(3')-VI,aph(3'')-Ib,sul1,tet(A),tet(G),blaPAO,blaOXA-396,catB7" 98.4 Good 1 1 1 1 1 1 1 0 1 1 0 0

Pseudomonas aeruginosa strain MRSN3587 287 WGS MRSN3587 RXUU01000000 186 6494873 urine United States "Human, Homo sapiens" Susceptible;Intermediate AMR Panel "crpP,fosA,catB7,blaPAO,blaOXA-486,aph(3')-Iib" 98.3 Good 1 1 0 1 1 1 1 0 0 0 0 1

Pseudomonas aeruginosa strain MRSN23861 287 WGS MRSN23861 RXUQ01000000 253 7025487 respiratory United States "Human, Homo sapiens" Susceptible;Resistant;Intermediate AMR Panel "fosA,aph(3')-Iib,aac(6')-Iic,crpP,sul1,blaOXA-50,blaPAO,blaKPC-2,blaOXA-2,qacE,catB7" 97.8 Good 1 1 1 1 1 1 1 0 0 1 0 1

Pseudomonas aeruginosa strain MRSN20176 287 WGS MRSN20176 RXUW01000000 290 6717057 groin Afghanistan "Human, Homo sapiens" Intermediate;Resistant;Susceptible AMR Panel "fosA,aac(6')-Ib-cr,aac(6')-Ib3,ant(2'')-Ia,aph(3')-Iib,aph(3')-Ia,aph(3'')-Ib,aph(6)-Id,crpP,sul1,tet(G),blaOXA-10,blaPAO,blaOXA-395,blaVIM-11,cmlA1,catB7,qacE" 98.3 Good 1 1 1 1 1 1 1 0 0 1 0 0

Pseudomonas aeruginosa strain MRSN1906 287 WGS MRSN1906 RXVB01000000 96 7049219 groin United States "Human, Homo sapiens" Susceptible;Resistant AMR Panel "fosA,aadA1,aph(3')-Iib,ant(2'')-Ia,qnrA1,dfrA23,sul1,dfrA5,blaPAO,blaOXA-494,blaOXA-4396,blaOXA-10,qacE,cmlA1,catB7" 98.5 Good 1 1 1 1 1 1 1 0 0 1 0 1

Pseudomonas aeruginosa strain MRSN1938 287 WGS MRSN1938 RXUZ01000000 317 7052906 urine United States "Human, Homo sapiens" Susceptible;Resistant;Intermediate AMR Panel "fosA,aph(3')-Iib,ant(2'')-Ia,aph(3')-Ib,aph(3')-Iib,crpP,sul1,blaOXA-396,blaPAO,qacE,cmlA1,catB7" 98.4 Good 1 1 1 1 1 1 1 0 0 1 0 1

Pseudomonas aeruginosa strain MRSN1899 287 WGS MRSN1899 RXVD01000000 69 6573139 groin United States "Human, Homo sapiens" Susceptible;Resistant AMR Panel "crpP,fosA,catB7,blaPAO,blaOXA-50,aph(3')-Iib" 99 Good 1 1 0 1 1 1 1 0 0 0 0 1

Pseudomonas aeruginosa strain MRSN1902 287 WGS MRSN1902 RXVC01000000 67 6344431 groin United States "Human, Homo sapiens" Susceptible;Resistant AMR Panel "fosA,catB7,blaPAO,blaOXA-494,blaOXA-396,aph(3')-Iib" 99.4 Good 1 1 1 1 1 1 1 0 0 0 0 0

Pseudomonas aeruginosa strain MRSN17849 287 WGS MRSN17849 RXVK01000000 165 6738933 respiratory United States "Human, Homo sapiens" Susceptible;Intermediate AMR Panel "fosA,catB7,blaPAO,blaOXA-485,aph(3')-Iib" 98.4 Good 1 1 0 1 1 1 1 0 0 0 0 0

Pseudomonas aeruginosa strain MRSN16744 287 WGS MRSN16744 RXVO01000000 71 6776946 sterile tissue United States "Human, Homo sapiens" Susceptible;Intermediate;Resistant AMR Panel "fosA,catB7,blaPAO,blaOXA-50,aph(3')-Iib" 98.9 Good 1 1 0 1 1 1 1 0 0 1 0 1

Pseudomonas aeruginosa strain MRSN16740 287 WGS MRSN16740 RXVP01000000 186 6670940 respiratory United States "Human, Homo sapiens" Susceptible;Intermediate;Resistant AMR Panel "crpP,fosA,catB7,blaPAO,blaOXA-396,aph(3')-Iib" 98.2 Good 1 1 1 1 1 1 1 0 0 0 0 1

Pseudomonas aeruginosa strain MRSN16383 287 WGS MRSN16383 RXVQ01000000 56 6302224 respiratory United States "Human, Homo sapiens" Susceptible;Intermediate;Resistant AMR Panel "fosA,catB7,blaPAO,blaOXA-494,blaOXA-396,aph(3')-Iib" 99.1 Good 1 1 1 1 1 1 1 0 0 0 0 0

Pseudomonas aeruginosa strain MRSN16345 287 WGS MRSN16345 RXVR01000000 177 6333273 urine United States "Human, Homo sapiens" Susceptible;Resistant;Intermediate AMR Panel "fosA,catB7,blaPAO,blaOXA-486,aph(3')-Iib" 98.4 Good 1 1 0 1 1 1 1 0 0 0 0 0

Pseudomonas aeruginosa strain MRSN1583 287 WGS MRSN1583 RXVX01000000 222 6463376 respiratory United States "Human, Homo sapiens" Susceptible;Resistant;Intermediate AMR Panel "fosA,catB7,blaPAO,blaOXA-486,aph(3')-Iib" 98.3 Good 1 1 0 1 1 1 1 0 0 0 0 0

Pseudomonas aeruginosa strain MRSN1617 287 WGS MRSN1617 RXVT01000000 171 6780137 respiratory United States "Human, Homo sapiens" Susceptible;Resistant;Intermediate AMR Panel "crpP,fosA,catB7,blaPAO,blaOXA-494,blaOXA-396,aph(3')-Iib" 98 Good 1 1 1 1 1 1 1 0 0 0 0 1

Pseudomonas aeruginosa strain MRSN1388 287 WGS MRSN1388 RXWC01000000 122 7171428 groin United States "Human, Homo sapiens" Susceptible;Resistant AMR Panel "crpP,fosA,catB7,blaPAO,blaOXA-395,aph(3')-Iib,aph(6)-Id,aph(3'')-Ib," 98.1 Good 1 1 1 1 1 1 1 0 0 0 0 0

Pseudomonas aeruginosa strain MRSN15678 287 WGS MRSN15678 RXVZ01000000 203 6780257 wound United States "Human, Homo sapiens" Susceptible;Intermediate;Resistant AMR Panel "fosA,aadA6,aph(3')-Iib,sul1,crpP,blaPAO,blaOXA-488,qacE,catB7" 98.4 Good 1 1 0 1 1 1 1 0 0 1 0 1

Pseudomonas aeruginosa strain MRSN14981 287 WGS MRSN14981 RXWB01000000 204 6508561 respiratory United States "Human, Homo sapiens" Susceptible;Intermediate;Resistant AMR Panel "crpP,fosA,catB7,blaPAO,blaOXA-494,blaOXA-396,blaOXA-50,aph(3')-Iib" 98.2 Good 1 1 1 1 1 1 1 0 0 0 0 1

Pseudomonas aeruginosa strain MRSN409937 287 WGS MRSN409937 RXTX01000000 129 6487691 United States "Human, Homo sapiens" Susceptible;Resistant AMR Panel "crpP,fosA,catB7,blaPAO,blaOXA-50,aph(3')-Iib" 98.9 Good 1 1 0 1 1 1 1 0 0 0 0 1

Pseudomonas aeruginosa strain MRSN351791 287 WGS MRSN351791 RXUE01000000 248 6812488 urine United States "Human, Homo sapiens" Susceptible;Intermediate;Resistant AMR Panel "crpP,fosA,catB7,blaPAO,blaOXA-486,aph(3')-Iib" 97.9 Good 1 1 0 1 1 1 1 0 0 0 0 1

Pseudomonas aeruginosa strain MRSN30858 287 WGS MRSN30858 RXUJ01000000 186 6382502 respiratory United States "Human, Homo sapiens" Susceptible;Resistant;Intermediate AMR Panel "fosA,catB7,blaPAO,blaOXA-395,aph(3')-Iib" 98.7 Good 1 1 1 1 1 1 1 0 0 1 0 0

Pseudomonas aeruginosa strain MRSN26263 287 WGS MRSN26263 RXUL01000000 149 6430277 respiratory United States "Human, Homo sapiens" Susceptible;Resistant;Intermediate AMR Panel "crpP,fosA,catB7,blaPAO,blaOXA-50,aph(3')-Iib" 98.2 Good 1 1 0 1 1 1 1 0 0 0 0 1

Pseudomonas aeruginosa strain MRSN25623 287 WGS MRSN25623 RXUO01000000 140 6635285 respiratory United States "Human, Homo sapiens" Susceptible;Resistant AMR Panel "fosA,catB7,blaPAO,blaOXA-494,blaOXA-396,blaOXA-50,aph(3')-Iib" 98.4 Good 1 1 1 1 1 1 1 0 0 0 0 0

Pseudomonas aeruginosa strain MRSN18562 287 WGS MRSN18562 RXVI01000000 90 6494310 respiratory United States "Human, Homo sapiens" Susceptible;Intermediate;Resistant AMR Panel "crpP,fosA,catB7,blaPAO,blaOXA-50,aph(3')-Iib" 99.2 Good 0 1 1 1 1 1 1 0 0 0 0 1

Pseudomonas aeruginosa strain MRSN16344 287 WGS MRSN16344 RXVS01000000 220 6318735 wound United States "Human, Homo sapiens" Intermediate;Resistant;Susceptible AMR Panel "fosA,catB7,blaPAO,blaOXA-488,blaOXA-485,aph(3')-Iib" 98.8 Good 1 1 0 1 1 1 1 0 0 0 0 0

Pseudomonas aeruginosa strain MRSN1739 287 WGS MRSN1739 RXVL01000000 151 7031489 blood United States "Human, Homo sapiens" Susceptible;Resistant;Intermediate AMR Panel "aadA1b,aac(6')-Iic,aph(3')-Iib,fosA,crpP,sul1,blaPAO,blaOXA-2,blaOXA-486,qacE,catB7" 98.9 Good 1 1 0 1 1 1 1 0 0 1 0 1

Pseudomonas aeruginosa strain MRSN16847 287 WGS MRSN16847 RXVN01000000 63 6594082 groin United States "Human, Homo sapiens" Susceptible;Resistant AMR Panel "crpP,fosA,catB7,blaPAO,blaOXA-488,aph(3')-Iib" 98.6 Good 1 1 0 1 1 1 1 0 0 0 0 1

Pseudomonas aeruginosa strain MRSN15753 287 WGS MRSN15753 RXVY01000000 207 6547438 respiratory United States "Human, Homo sapiens" Susceptible;Resistant;Intermediate AMR Panel "fosA,catB7,blaPAO,blaOXA-486,aph(3')-Iib" 98.5 Good 1 1 0 1 1 1 1 0 0 0 0 0

Pseudomonas aeruginosa strain MRSN15566 287 WGS MRSN15566 RXWA01000000 238 6408848 urine United States "Human, Homo sapiens" Susceptible;Resistant AMR Panel "fosA,catB7,blaPAO,blaOXA-50,aph(3')-Iib" 99 Good 1 1 0 1 1 1 1 0 0 0 0 0

Pseudomonas aeruginosa strain MRSN12914 287 WGS MRSN12914 RXWH01000000 236 6756810 urine Afghanistan "Human, Homo sapiens" Susceptible;Resistant AMR Panel "fosA,aadA1,aph(3')-Iib,aac(6')-Il,ant(2'')-Ia,sul1,dfrB2,tet(A),blaPAO,blaOXA-10,blaVEB-1,blaOXA-50,qacE,catB7" 98.7 Good 1 1 0 1 1 1 1 0 1 1 0 1

Pseudomonas aeruginosa strain MRSN12283 287 WGS MRSN12283 RXWK01000000 56 6301640 urine United States "Human, Homo sapiens" Susceptible;Resistant;Intermediate AMR Panel "fosA,catB7,blaPAO,blaOXA-494,blaOXA-396,blaOXA-50,aph(3')-Iib" 99.6 Good 1 1 1 1 1 1 1 0 0 0 0 0

Pseudomonas aeruginosa strain MRSN12365 287 WGS MRSN12365 RXWJ01000000 258 6869499 respiratory United States "Human, Homo sapiens" Susceptible;Intermediate;Resistant AMR Panel "crpP,fosA,catB7,blaPAO,blaOXA-488,aph(3')-Iib" 98.5 Good 1 1 0 1 1 1 1 0 0 1 0 0

Pseudomonas aeruginosa strain MRSN358800 287 WGS MRSN358800 RXUD01000000 195 6501460 respiratory United States "Human, Homo sapiens" Intermediate;Resistant;Susceptible AMR Panel "fosA,catB7,blaPAO,blaOXA-395,aph(3')-Iib" 98.1 Good 1 1 1 1 1 1 1 0 0 0 0 0

Pseudomonas aeruginosa strain MRSN11538 287 WGS MRSN11538 RXWN01000000 212 6528585 wound United States "Human, Homo sapiens" Susceptible;Intermediate;Resistant AMR Panel "fosA,catB7,blaPAO,blaOXA-50,aph(3')-Iib" 98.4 Good 1 1 0 1 1 1 1 0 0 0 0 0

Pseudomonas aeruginosa strain MRSN11278 287 WGS MRSN11278 RXWS01000000 212 6893110 respiratory United States "Human, Homo sapiens" Intermediate;Resistant;Susceptible AMR Panel "aadA6,aph(3')-Iib,ant(2'')-Ia,fosA,crpP,sul1,blaPAO,blaOXA-395,qacE.catB7" 97.8 Good 1 1 1 1 1 1 1 0 0 1 0 1

Pseudomonas aeruginosa strain MRSN11281 287 WGS MRSN11281 RXWR01000000 48 6323478 wound United States "Human, Homo sapiens" Susceptible;Resistant;Intermediate AMR Panel "crpP,fosA,catB7,blaPAO,blaOXA-50,aph(3')-Iib" 99.6 Good 1 1 0 1 1 1 1 0 0 0 0 1

Pseudomonas aeruginosa strain MRSN13488 287 WGS MRSN13488 RXWF01000000 38 5912421 urine United States "Human, Homo sapiens" Susceptible;Intermediate;Resistant AMR Panel "fosA,catB7,blaPAO,blaOXA-50,aph(3')-Iib" 98.3 Good 1 1 0 1 1 1 1 0 0 0 0 0

Pseudomonas aeruginosa strain MRSN1344 287 WGS MRSN1344 RXWG01000000 213 6232278 groin United States "Human, Homo sapiens" Susceptible;Resistant AMR Panel "fosA,catB7,blaPAO,blaOXA-486,aph(3')-Iib" 98.9 Good 1 1 0 1 1 1 1 0 0 0 0 0

Pseudomonas aeruginosa strain MRSN12368 287 WGS MRSN12368 RXWI01000000 194 6340159 blood United States "Human, Homo sapiens" Susceptible;Intermediate;Resistant AMR Panel "fosA,catB7,blaPAO,blaOXA-494,blaOXA-396,blaOXA-50,aph(3')-Iib" 98.9 Good 1 1 1 1 1 1 1 0 0 0 0 0

Pseudomonas aeruginosa strain MRSN12282 287 WGS MRSN12282 RXWL01000000 277 6876949 respiratory United States "Human, Homo sapiens" Susceptible;Intermediate;Resistant AMR Panel "crpP,fosA,catB7,blaPAO,blaOXA-494,blaOXA-396,blaOXA-50,aph(3')-Iib" 97.8 Good 1 1 1 1 1 1 1 0 0 0 0 1

Pseudomonas aeruginosa strain MRSN11536 287 WGS MRSN11536 RXWO01000000 217 6935556 wound United States "Human, Homo sapiens" Susceptible;Resistant AMR Panel "aadA1b,aac(6')-Ib3,aph(3')-Iib,aac(6')-Ib-cr,fosA,sul1,crpP,blaPAO,blaOXA-50,blaOXA-2,catB7,qacE" 98.4 Good 1 1 0 1 1 1 1 0 0 0 0 1

Pseudomonas aeruginosa strain MRSN11286 287 WGS MRSN11286 RXWP01000000 224 6711313 wound United States "Human, Homo sapiens" Susceptible;Resistant;Intermediate AMR Panel "crpP,fosA,catB7,blaPAO,blaOXA-486,aph(3')-Iib" 98.2 Good 1 1 0 1 1 1 1 0 0 0 0 1

Pseudomonas aeruginosa strain U4581 287 WGS U4581 RXFP01000000 189 6891948 Resistant Computational Prediction "aadA1,aac(6')-Ib-cr,aph(3')-Iib,aac(6')-Ib-Hangzhou,aph(3')-VI,fosA,sul1,crpP,blaPAO,blaIMP-6,blaOXA-488,blaOXA-1,blaIMP-1,blaIMP-25mcatB3,cmx,catB7,qacE" 98.1 Good 1 1 1 1 1 1 1 0 0 1 0 1

Pseudomonas aeruginosa strain SMC5451 287 WGS SMC5451 RWZL01000000 58 6464084 sputum United States "Human, Homo sapiens" Resistant Computational Prediction "crpP,fosA,catB7,blaPAO,blaOXA-494,blaOXA-396,blaOXA-50,aph(3')-Iib" 97.9 Good 1 1 1 1 1 1 1 0 0 0 0 1

Pseudomonas aeruginosa strain HCF86 287 WGS HCF86 RXAL01000000 37 6648956 throat Germany "Human, Homo sapiens" Resistant Computational Prediction "crpP,fosA,catB7,blaPAO,blaOXA-488,aph(3')-Iib" 99.1 Good 1 1 0 1 1 1 1 0 0 1 0 1

Pseudomonas aeruginosa strain Zw75_2 287 WGS Zw75_2 RWZS01000000 35 6477516 throat Germany "Human, Homo sapiens" Resistant Computational Prediction "crpP,fosA,catB7,blaPAO,blaOXA-486,aph(3')-Iib" 98.4 Good 1 1 0 1 1 1 1 0 0 0 0 1

Pseudomonas aeruginosa strain HCF367 287 WGS HCF367 RXAG01000000 49 6333536 throat Germany "Human, Homo sapiens" Resistant Computational Prediction "crpP,fosA,catB7,blaPAO,blaOXA-50,blaOXA-396," 98.2 Good 1 1 1 1 1 1 1 0 0 0 0 1

Pseudomonas aeruginosa strain Zw9 287 WGS Zw9 RXAS01000000 39 6558087 throat Germany "Human, Homo sapiens" Resistant Computational Prediction "crpP,fosA,catB7,blaPAO,blaOXA-488,aph(3')-Iib" 98.5 Good 1 1 0 1 1 1 1 0 0 1 0 1

Pseudomonas aeruginosa strain MCF199 287 WGS MCF199 RXBD01000000 99 7223943 throat Germany "Human, Homo sapiens" Resistant Computational Prediction "crpP,fosA,catB7,blaPAO,blaOXA-396,aph(3')-Iib" 98.4 Good 1 1 1 1 1 1 1 0 0 0 0 1

Pseudomonas aeruginosa strain W15Dec4 287 WGS W15Dec4 RXCS01000000 52 6875213 River Belgium Resistant Computational Prediction "crpP,fosA,catB7,blaPAO,blaOXA-494,blaOXA-396,aph(3')-Iib" 98.5 Good 1 1 1 1 1 1 1 0 0 0 0 1

Pseudomonas aeruginosa strain Jp1155 287 WGS Jp1155 RXDA01000000 67 6805497 Sea water (open ocean) Japan Resistant Computational Prediction "crpP,fosA,catB7,blaPAO,blaOXA-395,aph(3')-Iib" 98.7 Good 1 1 1 1 1 1 1 0 0 0 0 1

Pseudomonas aeruginosa strain Jp1200 287 WGS Jp1200 RXCY01000000 90 6975512 Sea water (open ocean) Japan Resistant Computational Prediction "crpP,fosA,catB7,blaPAO,blaOXA-395,aph(3')-Iib" 98.7 Good 1 1 1 1 1 1 1 0 0 0 0 1

Pseudomonas aeruginosa strain AMT0046-108 287 WGS AMT0046-108 RXDH01000000 21 6449966 Sputum United States "Human, Homo sapiens" Resistant Computational Prediction "fosA,catB7,blaPAO,blaOXA-50,aph(3')-Iib" 99.1 Good 1 1 0 1 1 1 1 0 0 0 0 0

Pseudomonas aeruginosa strain AUS727 287 WGS AUS727 RXDS01000000 41 6156005 Australia "Human, Homo sapiens" Resistant Computational Prediction "fosA,catB7,blaPAO,blaOXA-395,aph(3')-Iib" 98.2 Good 1 1 1 1 1 1 1 0 0 0 0 0

Pseudomonas aeruginosa strain AUS501 287 WGS AUS501 RXEC01000000 35 6489430 River Australia Resistant Computational Prediction "crpP,fosA,catB7,blaPAO,blaOXA-395,aph(3')-Iib" 99 Good 1 1 1 1 1 1 1 0 0 0 0 1

Pseudomonas aeruginosa strain AUS178 287 WGS AUS178 RXEF01000000 30 6350641 Pool Australia Resistant Computational Prediction "fosA,catB7,blaPAO,blaOXA-396,aph(3')-Iib" 99.3 Good 1 1 1 1 1 1 1 0 0 0 0 0

Pseudomonas aeruginosa strain JYH11 287 WGS JYH11 RXBW01000000 53 6846009 River Japan Resistant Computational Prediction "crpP,fosA,catB7,blaPAO,blaOXA-50,blaOXA-488,blaOXA-395,blaOXA-485,aph(3')-Iib" 98.5 Good 1 1 1 1 1 1 1 0 0 1 0 1

Pseudomonas aeruginosa strain T3044 287 WGS T3044 RXCD01000000 45 6733566 Nasal mucosa Thailand "Human, Homo sapiens" Resistant Computational Prediction "crpP,fosA,catB7,blaPAO,blaOXA-494,blaOXA-396,aph(3')-Iib" 98.2 Good 1 1 1 1 1 1 1 0 0 0 0 1

Pseudomonas aeruginosa strain T3677 287 WGS T3677 RXCE01000000 40 6474838 Maxillary Sinus Thailand "Human, Homo sapiens" Resistant Computational Prediction "crpP,fosA,catB7,blaPAO,blaOXA-50,aph(3')-Iib" 99.1 Good 1 1 0 1 1 1 1 0 0 0 0 1

Pseudomonas aeruginosa strain T2584 287 WGS T2584 RXCI01000000 39 6993634 Sputum Thailand "Human, Homo sapiens" Resistant Computational Prediction "fosA,ant(2'')-Ia,aph(3')-Iib,aph(3'')-Ib,aph(6)-Id,tet(G),blaOXA-396,blapAO,blaOXA-494,blaOXA-50,qacE,cmlA1,catB7" 99 Good 1 1 1 1 1 1 1 0 1 1 0 0

Pseudomonas aeruginosa strain W15Okt31 287 WGS W15Okt31 RXCR01000000 41 6671598 River Belgium Resistant Computational Prediction "crpP,fosA,catB7,blaPAO,blaOXA-396,aph(3')-Iib" 99 Good 1 1 1 1 1 1 1 0 0 0 0 1

Pseudomonas aeruginosa strain Jp1206 287 WGS Jp1206 RXCX01000000 81 6813593 Sea water (open ocean) Japan Resistant Computational Prediction "crpP,fosA,catB7,blaPAO,blaOXA-395,aph(3')-Iib" 98.9 Good 1 1 1 1 1 1 1 0 0 0 0 1

Pseudomonas aeruginosa strain Jp1170 287 WGS Jp1170 RXCZ01000000 68 6914139 Sea water (open ocean) Japan Resistant Computational Prediction "crpP,fosA,catB7,blaPAO,blaOXA-395,aph(3')-Iib" 98.7 Good 1 1 1 1 1 1 1 0 0 0 0 1

Pseudomonas aeruginosa strain AMT0046-107 287 WGS AMT0046-107 RXDI01000000 19 6449216 Sputum United States "Human, Homo sapiens" Resistant Computational Prediction "fosA,catB7,blaPAO,blaOXA-50,aph(3')-Iib" 99 Good 1 1 0 1 1 1 1 0 0 0 0 0

Pseudomonas aeruginosa strain AMT0020-83 287 WGS AMT0020-83 RXDL01000000 88 6707393 Sputum United States "Human, Homo sapiens" Resistant Computational Prediction "fosA,catB7,blaPAO,blaOXA-50,aph(3')-Iib" 98.6 Good 1 1 0 1 1 1 1 0 0 1 0 0

Pseudomonas aeruginosa strain AUS456 287 WGS AUS456 RXDT01000000 70 7068727 milk Australia "Goat, Capra hircus" Resistant Computational Prediction "crpP,fosA,catB7,blaPAO,blaOXA-396,aph(3')-Iib" 98.7 Good 1 1 1 1 1 1 1 0 0 0 0 1

Pseudomonas aeruginosa strain AUS455 287 WGS AUS455 RXDU01000000 49 6977036 Australia "Human, Homo sapiens" Resistant Computational Prediction "crpP,fosA,catB7,blaPAO,blaOXA-396,aph(3')-Iib" 98.8 Good 1 1 1 1 1 1 1 0 0 0 0 1

Pseudomonas aeruginosa strain AUS074 287 WGS AUS074 RXDV01000000 52 7036422 sputum Australia "Human, Homo sapiens" Resistant Computational Prediction "crpP,fosA,catB7,blaPAO,blaOXA-396,aph(3')-Iib" 98.7 Good 1 1 1 1 1 1 1 0 0 0 0 1

Pseudomonas aeruginosa strain AUS153 287 WGS AUS153 RXEO01000000 31 6347317 River Australia Resistant Computational Prediction "fosA,catB7,blaPAO,blaOXA-50,aph(3')-Iib" 99.3 Good 1 1 0 1 1 1 1 0 0 0 0 0

Pseudomonas aeruginosa strain AUS141 287 WGS AUS141 RXEP01000000 31 6558573 River Australia Resistant Computational Prediction "fosA,catB7,blaPAO,blaOXA-50,aph(3')-Iib" 99.2 Good 1 1 0 1 1 1 1 0 0 0 0 0

Pseudomonas aeruginosa strain AUS265 287 WGS AUS265 RXEY01000000 46 6822691 Pool Australia Resistant Computational Prediction "fosA,catB7,blaPAO,blaOXA-50,aph(3')-Iib" 98 Good 1 1 0 1 1 1 1 0 0 0 0 0

Pseudomonas aeruginosa strain AUS125 287 WGS AUS125 RXEU01000000 37 6986406 Pool Australia Resistant Computational Prediction "crpP,fosA,catB7,blaPAO,blaOXA-494,blaOXA-396,aph(3')-Iib" 98.1 Good 1 1 1 1 1 1 1 0 0 0 0 0

Pseudomonas aeruginosa strain GCID_CRE_0006 287 WGS GCID_CRE_0006 RYXU01000000 191 6845168 urine Egypt Resistant Computational Prediction "fosA,ant(2'')-Ia,aph(3'')-Ib,aph(3')-Via,aph(3')-Iib,crpP,sul1,blaPAO,blaVIM-24,blaOXA-10,blaOXA-488,qacE" 98.3 Good 1 1 1 1 1 1 1 0 0 1 1 1

Pseudomonas aeruginosa strain MRSN401528 287 WGS MRSN401528 RXTY01000000 98 6486368 urine United States "Human, Homo sapiens" Susceptible;Resistant;Intermediate AMR Panel "crpP,fosA,catB7,blaPAO,blaOXA-494,blaOXA-396,blaOXA-50,aph(3')-Iib" 98.9 Good 1 1 1 1 1 1 1 0 0 0 0 1

Pseudomonas aeruginosa strain MRSN369569 287 WGS MRSN369569 RXUC01000000 189 6316238 United States "Human, Homo sapiens" Intermediate;Susceptible;Resistant AMR Panel "fosA,catB7,blaPAO,blaOXA-486,aph(3')-Iib" 98.7 Good 1 1 0 1 1 1 1 0 0 0 0 0

Pseudomonas aeruginosa strain MRSN2144 287 WGS MRSN2144 RXUR01000000 129 7032738 urine United States "Human, Homo sapiens" Susceptible;Resistant AMR Panel "aadA6,aph(3')-Iib,fosA,crpP,sul1,blaPAO,blaOXA-396,qacE,catB7" 98.9 Good 1 1 1 1 1 1 1 0 0 0 0 1

Pseudomonas aeruginosa strain MRSN2108 287 WGS MRSN2108 RXUS01000000 71 6394452 sterile tissue United States "Human, Homo sapiens" Susceptible;Resistant;Intermediate AMR Panel "crpP,fosA,catB7,blaPAO,blaOXA-50,aph(3')-Iib" 98.9 Good 1 1 0 1 1 1 1 0 0 0 0 1

Pseudomonas aeruginosa strain MRSN18803 287 WGS MRSN18803 RXVG01000000 144 6191380 respiratory United States "Human, Homo sapiens" Susceptible;Intermediate;Resistant AMR Panel "fosA,catB7,blaPAO,blaOXA-486,aph(3')-Iib" 98.5 Good 1 1 0 1 1 1 1 0 0 0 0 0

Pseudomonas aeruginosa strain MRSN317 287 WGS MRSN317 RXUH01000000 69 6349621 wound United States "Human, Homo sapiens" Susceptible;Resistant AMR Panel "fosA,catB7,blaPAO,blaOXA-488,aph(3')-Iib" 99.4 Good 1 1 0 1 1 1 1 0 0 1 0 0

Pseudomonas aeruginosa strain MRSN18560 287 WGS MRSN18560 RXVJ01000000 38 6268481 wound United States "Human, Homo sapiens" Susceptible;Intermediate AMR Panel "fosA,catB7,blaPAO,blaOXA-486,aph(3')-Iib" 99.6 Good 1 1 0 1 1 1 1 0 0 0 0 0

Pseudomonas aeruginosa strain SP4527 287 Complete SP4527 CP034409 1 7005215 sputum India "Human, Homo sapiens" Resistant Computational Prediction "aadA1,msr€,aph(3')-Iib,ant(2'')-Ia,aph(3')-VI,aac(6')-Il,aac(6')-Il,sul1,ARR-3,dfrB2,crpP,tet(A),blaPAO,blaOXA-10,blaOXA-50,blaNDM-1,blaNDM-11,blaVEB-1,blaPME-1,qacE,catB7,cmlA1,fosA" 97 Good 1 1 1 1 1 1 1 1 1 1 0 1

Pseudomonas aeruginosa strain GIMC5016:PA1840 287 WGS GIMC5016:PA1840 CP034430 1 6750271 burn wound Russia "Human, Homo sapiens" Resistant Computational Prediction "aadA1,aac(6')-Il,ant(2'')-Ia,aph(3')-Iib,fosA,sul1,dfrB2,tet(A),blaVEB-1,blaOXA-50,blaOXA-10.blaPAO,qacE,catB7" 99 Good 1 1 0 1 1 1 1 0 1 1 0 1

Pseudomonas aeruginosa strain LW 287 Complete LW CP022478 1 6824837 sputum China "Human, Homo sapiens" Resistant Computational Prediction "crpP,fosA,catB7,blaPAO,blaOXA-395,aph(3')-Iib" 98.6 Good 1 1 1 1 1 1 1 0 0 0 0 1

Pseudomonas aeruginosa strain paerg001 287 WGS paerg001 UWVR01000000 2 6501980 hospital Switzerland "Human, Homo sapiens" Resistant Computational Prediction "fosA,catB7,blaPAO,blaOXA-494,blaOXA-396,aph(3')-Iib" 98.6 Good 1 1 1 1 1 1 1 0 0 0 0 0

Pseudomonas aeruginosa strain paerg008 287 WGS paerg008 UWXL01000000 2 6508797 hospital Switzerland "Human, Homo sapiens" Resistant Computational Prediction "fosA,catB7,blaPAO,blaOXA-494,blaOXA-396,aph(3')-Iib" 98.7 Good 1 1 1 1 1 1 1 0 0 0 0 0

Pseudomonas aeruginosa strain paerg000 287 Complete paerg000 LR130528 1 6493562 hospital Switzerland "Human, Homo sapiens" Resistant Computational Prediction "fosA,catB7,blaPAO,blaOXA-494,blaOXA-396,aph(3')-Iib" 98.9 Good 1 1 1 1 1 1 1 0 0 0 0 0

Pseudomonas aeruginosa strain NCTC13715 287 Complete NCTC13715 LR134330 1 6765311 urine United Kingdom "Human, Homo sapiens" Resistant Computational Prediction "fosA,aac(6')-Ib-cr,aadA1,aac(6')-Ib3,sul1,dfrB2,aph(3')-Iib,ARR-2,blaPAO,blaOXA-10,blaOXA-395,blaVIM-48,blaVIM-2,cmlA1,qacE,catB7" 99.2 Good 1 1 1 1 1 1 1 0 0 1 0 1

Pseudomonas aeruginosa AZPAE15071 287 WGS AZPAE15071 JTMB00000000 110 7403124 respiratory tract infection Germany "Human, Homo sapiens" Resistant;Susceptible AMR Panel "crpP,fosA,catB7,blaPAO,blaOXA-50,aph(3')-Iib,aph(3'')-Ib,aph(6)-Id" 98.4 100 Good 1 1 0 1 1 1 1 0 0 0 0 1

Pseudomonas aeruginosa AZPAE15070 287 WGS AZPAE15070 JTMC00000000 99 7164330 urinary tract infection Germany "Human, Homo sapiens" Resistant AMR Panel "fosA,aph(3')-Iib,aac(6')-Ib3,aac(6')-Ib-cr,crpP,sul1,blaPAO,blaOXA-488,qacE,catB7" 98.5 100 Good 1 1 0 1 1 1 1 0 0 0 0 1

Pseudomonas aeruginosa AZPAE15069 287 WGS AZPAE15069 JTMD00000000 128 6980745 itra-abdominal tract infection Germany "Human, Homo sapiens" Resistant AMR Panel "crpP,fosA,catB7,blaPAO,blaOXA-396,aph(3')-Iib" 98.7 100 0.6 Good 1 1 1 1 1 1 1 0 0 1 0 1

Pseudomonas aeruginosa AZPAE15065 287 WGS AZPAE15065 JTMH00000000 68 6184461 respiratory tract infection Brazil "Human, Homo sapiens" Resistant;Susceptible AMR Panel "fosA,catB7,blaPAO,blaOXA-396,aph(3')-Iib" 99.4 99.7 Good 1 1 1 1 1 1 1 0 0 0 0 0

Pseudomonas aeruginosa AZPAE15064 287 WGS AZPAE15064 JTMI00000000 124 6687065 itra-abdominal tract infection Brazil "Human, Homo sapiens" Resistant;Susceptible AMR Panel "fosA,sul1,aac(6')-Il,blaOXA-395,ARR-4,blapAO,aph(3')-Iib,qacE,catB7,catB3" 98.7 100 0.3 Good 1 1 1 1 1 1 1 0 0 0 0 0

Pseudomonas aeruginosa AZPAE15058 287 WGS AZPAE15058 JTMO00000000 103 6567883 respiratory tract infection France "Human, Homo sapiens" Resistant AMR Panel "fosA,catB7,blaPAO,blaOXA-488,aph(3')-Iib" 98.7 99.7 Good 1 1 0 1 1 1 1 0 0 1 0 0

Pseudomonas aeruginosa AZPAE15057 287 WGS AZPAE15057 JTMP00000000 85 6451298 respiratory tract infection China "Human, Homo sapiens" Susceptible;Resistant AMR Panel "fosA,catB7,blaPAO,blaOXA-494,blaOXA-396,aph(3')-Iib" 99.2 100 Good 1 1 1 1 1 1 1 0 0 0 0 0

Pseudomonas aeruginosa AZPAE15056 287 WGS AZPAE15056 JTMQ00000000 90 6287511 respiratory tract infection China "Human, Homo sapiens" Resistant;Intermediate;Susceptible AMR Panel "blaOXA-395,blaPAO,fosA,catB7,aph(3')-IIb," 98.4 99.1 0.3 Good 1 1 1 1 1 1 1 0 0 0 0 0

Pseudomonas aeruginosa AZPAE15054 287 WGS AZPAE15054 JTMS00000000 101 6647129 urinary tract infection Colombia "Human, Homo sapiens" Resistant AMR Panel "fosA,aadA1b,aph(3')-Iib,aac(6')-Ib-cr,aac(6')-Ib-Hangzhou,sul1,crpP,blaOXA-395,blaOXA-2,blaPAO,qacE,catB7" 98.6 98.8 Good 1 1 1 1 1 1 1 0 0 1 0 1

Pseudomonas aeruginosa AZPAE15051 287 WGS AZPAE15051 JTMV00000000 117 6814130 itra-abdominal tract infection China "Human, Homo sapiens" Resistant AMR Panel "fosA,catB7,blaPAO,blaOXA-488,aph(3')-Iib" 99 99.7 Good 1 1 0 1 1 1 1 0 0 0 0 0

Pseudomonas aeruginosa AZPAE15048 287 WGS AZPAE15048 JTMY00000000 128 6924072 urinary tract infection Germany "Human, Homo sapiens" Intermediate;Susceptible AMR Panel "aph(3')-IIb,blaOXA-396,blaPAO,fosA,catB7" 99.1 100 Good 1 1 1 1 1 1 1 0 0 0 0 0

Pseudomonas aeruginosa AZPAE15046 287 WGS AZPAE15046 JTNA00000000 85 6345609 urinary tract infection Argentina "Human, Homo sapiens" Resistant;Intermediate;Susceptible AMR Panel "aph(3')-IIb,catB7,blaOXA-396,blaPAO,fosA" 99.4 100 0.3 Good 1 1 1 1 1 1 1 0 0 0 0 0

Pseudomonas aeruginosa AZPAE15040 287 WGS AZPAE15040 JTNG00000000 117 6601216 itra-abdominal tract infection Germany "Human, Homo sapiens" Resistant;Susceptible AMR Panel "aph(3')-IIb,catB7,blaOXA-486,blaPAO,fosA" 99 99.7 Good 1 1 0 1 1 1 1 0 0 0 0 0

Pseudomonas aeruginosa AZPAE15035 287 WGS AZPAE15035 JTNL00000000 95 6673047 respiratory tract infection Spain "Human, Homo sapiens" Resistant AMR Panel "crpP,aph(3')-IIb,catB7,blaOXA-488,blaPAO,fosA" 99 100 Good 1 1 0 1 1 1 1 0 0 1 0 1

Pseudomonas aeruginosa strain MRSN 17623 287 WGS MRSN 17623 JYGB02000000 40 6933068 USA "Human, Homo sapiens" Resistant Computational Prediction "fosA,aac(6')-Ib-cr,aph(3'')-Ib,aph(3')-Ia,aac(6')-Ib3,aph(3')-Iib,aph(6)-Id,crpP,sul1,blaVIM-6,blaPAO,blaOXA-494,blaOXA-396,blaOXA-10,qacE,cmx,catB7" 99.5 Good 1 1 1 1 1 1 1 0 0 0 0 1

Pseudomonas aeruginosa AZPAE15030 287 WGS AZPAE15030 JTNQ00000000 94 6608631 itra-abdominal tract infection Germany "Human, Homo sapiens" Resistant;Susceptible AMR Panel "armA,msr€,aph(3')-Iib,mph€,fosA,crpP,blaOXA-488,blaPAO,catB7" 98.5 100 Good 1 1 0 1 1 1 1 1 0 1 0 1

Pseudomonas aeruginosa strain PA12GY72 287 WGS PA12GY72 MKEL01000000 691 7084423 urine China "Human, Homo sapiens" Resistant Computational Prediction "crpP,fosA,catB7,blaPAO,blaOXA-494,blaOXA-396,aph(3')-Iib" 92.8 Good 1 1 1 1 1 1 1 0 0 0 0 1

Pseudomonas aeruginosa strain PA13SY16 287 WGS PA13SY16 MKEM01000000 439 6842275 urine China "Human, Homo sapiens" Resistant Computational Prediction "armA,msr€,aph(3')-Iib,aac(6')-Ib3,aac(6')-Ib-cr,aph(3')-Ia,fosA,mph€,tet(C) ,blaPAO,blaPER-1,blaOXA-395,blaIMP-45,blaOXA-1,catB3,catB7" 93.9 Good 1 1 1 1 1 1 1 1 0 1 0 1

Pseudomonas aeruginosa strain AR_0090 287 WGS AR_0090 MPBT01000000 219 6773465 Intermediate;Not defined;Resistant;Susceptible Computational Prediction "fosA,aac(6')-33,msr€,ant(2'')-Ia,aph(3')-Iib,mph€,sul1,blaKPC-5,blaPAO,blaOXA-50,qacE,catB7" 95.9 Good 1 1 1 1 1 1 1 1 0 1 0 0

Pseudomonas aeruginosa strain Pae42 287 WGS Pae42 SBJW01000000 132 7151967 sputum Brazil "Human, Homo sapiens" Resistant Computational Prediction "crpP,aph(3')-IIb,catB7,blaOXA-50,blaPAO,fosA" 98.7 Good 1 1 0 1 1 1 1 0 0 0 0 1

Pseudomonas aeruginosa strain Pae28 287 WGS Pae28 SBJT01000000 185 6750423 Suture material Brazil "Human, Homo sapiens" Resistant Computational Prediction "fosA,aac(6')-Ib3,aac(6')-Ib-cr,aac(6')-Iq,dfrA21,sul1,blaPAO,blaOXA-50,blaGES-1,qacE,cmx,catB7" 99.2 Good 1 1 0 0 1 1 1 0 0 0 0 1

Pseudomonas aeruginosa AZPAE15029 287 WGS AZPAE15029 JTNR00000000 89 6860916 respiratory tract infection France "Human, Homo sapiens" Resistant AMR Panel "fosA,aac(6')-Ib-cr,aph(3')-Iib,aac(6')-Iq,aac(6')-Ib3,sul1,dfrA21,blaGES-5,blaPAO,blaOXA-50,catB7,cmx,qacE" 98.9 99.7 Good 1 1 0 1 1 1 1 0 0 0 0 1

Pseudomonas aeruginosa strain Pae83 287 WGS Pae83 SBKB01000000 191 6837897 sputum Brazil "Human, Homo sapiens" Resistant Computational Prediction "aadA2,aac(6')-Il,aph(3')-Iib,aac(3)-Id,fosA,dfrB5,crpP,tet(G),blaVIM-2,blaPAO,blaOXA-4,blaOXA-486,cmlA1,qacE,catB7" 99.2 Good 1 1 1 1 1 1 1 0 1 1 0 1

Pseudomonas aeruginosa strain Pae29 287 WGS Pae29 SBJU01000000 143 6947214 sputum Brazil "Human, Homo sapiens" Resistant Computational Prediction "crpP,aph(3')-IIb,catB7,blaOXA-50,blaPAO,fosA" 98.7 Good 1 1 0 1 1 1 1 0 0 0 0 1

Pseudomonas aeruginosa strain Pae12 287 WGS Pae12 SBJQ01000000 199 6975640 Tissue fragment Brazil "Human, Homo sapiens" Resistant Computational Prediction "fosA,aac(6')-Ib-cr,aph(3')-Iib,aac(6')-Ib3,crpP,sul1,blaPAO,blaOXA-494,blaOXA-396,qacE,catB3,catB7" 99.2 Good 1 1 1 1 1 1 1 0 0 0 0 1

Pseudomonas aeruginosa strain Pae39 287 WGS Pae39 SBJV01000000 186 6765150 sputum Brazil "Human, Homo sapiens" Resistant Computational Prediction "fosA,aac(6')-Ib-cr,aac(6')-Ib3,aph(3')-Iib,aac(6')-Iq,dfrA21,sul1,catB7,cmx,blaKPC-2,blaGES-1,blaPAO,blaOXA-50,qacE" 99.2 Good 1 1 0 1 1 1 1 0 0 0 0 1

Pseudomonas aeruginosa strain CCBH5698 287 WGS CCBH5698 SELC01000000 99 6883915 Secretion swab Brazil "Human, Homo sapiens" Resistant Computational Prediction "fosA,aac(6')-Ib-cr,rmtD,aac(6')-Ib3,aph(3')-Iib,aadA7,crpP,sul1,blaSPM-1,blaPAO,blaOXA-56,blaOXA-494,blaOXA-396,catB7,qacE" 98.7 Good 1 1 1 1 1 1 1 0 0 1 0 1

Pseudomonas aeruginosa strain CCBH18249 287 WGS CCBH18249 SELB01000000 99 7003379 urine Brazil "Human, Homo sapiens" Resistant Computational Prediction "fosA,aac(6')-Ib-cr,rmtD,aac(6')-Ib3,aph(3')-Iib,aadA7,crpP,sul1,blaSPM-1,blaPAO,blaOXA-56,blaOXA-494,blaOXA-396,catB7,qacE" 98.9 Good 1 1 1 1 1 1 1 0 0 1 0 1

Pseudomonas aeruginosa strain CCBH5939 287 WGS CCBH5939 SEME01000000 129 6669061 urine Brazil "Human, Homo sapiens" Resistant Computational Prediction "aadA7,aac(6')-Ib-cr,aph(3')-Iib,aac(6')-Ib3,rmtD,fosA,crpP,sul1,blaPAO,blaoXA-56,blaOXA-494,blaOXA-396,cmx,catB7" 98.5 Good 1 1 1 1 1 1 1 0 0 1 0 1

Pseudomonas aeruginosa strain CCBH4850 287 WGS CCBH4850 SEMF01000000 103 6699288 urine Brazil "Human, Homo sapiens" Resistant Computational Prediction "fosA,aac(6')-Ib-cr,rmtD,aac(6')-Ib3,aph(3')-Iib,aadA7,crpP,sul1,blaSPM-1,blaPAO,blaOXA-56,blaOXA-494,blaOXA-396,catB7" 98.8 Good 1 1 1 1 1 1 1 0 0 1 0 1

Pseudomonas aeruginosa strain 1334/14 287 Complete 1334/14 CP035739 1 6902135 eye Poland "Human, Homo sapiens" Resistant Computational Prediction "aadA1,msr€,rmtD2,aph(3'')-Ib,aph(3')-VI,aph(6)-Id,ant(2'')-Ia,aph(3')-Iib,sul1.dfrA1,fosA,mph€,mph(F),qnrVC1,crpP,tet(G),ARR-2,blaDIM-1,blaPME-1,blaOXA-10,blaOXA-486,blaPAO,blaNDM-1,cmlA1,qacE,catB7" 98.7 Good 1 1 1 1 1 1 1 1 1 1 0 1

Pseudomonas aeruginosa strain PAE1880 287 WGS PAE1880 SGUI01000000 205 7257822 urine Spain "Human, Homo sapiens" Resistant Computational Prediction "fosA,crpP,catB7,blaPAO,blaVIM-7,blaOXA-50,aac(6')-Iia,aph(3')-Iib" 98.2 Good 1 1 1 1 1 1 1 0 0 1 0 1

Pseudomonas aeruginosa strain PA-VAP-3 287 Complete PA-VAP-3 CP028330 1 6665262 BAL France "Human, Homo sapiens" Resistant Computational Prediction "aph(3')-IIb,catB7,blaOXA-488,blaPAO,fosA" 99.3 Good 1 1 0 1 1 1 1 0 0 0 0 0

Pseudomonas aeruginosa strain 116 287 WGS 116 SCYH01000000 984 6491669 hospital Italy "Human, Homo sapiens" Resistant Computational Prediction "sul1,aac(3)-Ic,aph(3')-Iib,aac(6')-Ib-Hangzhou,aac(6')-Ib-cr,blaOXA-488,blaPAO,qacE,cmlA1,catB7,catB10" 91.3 Good 1 1 0 1 0 1 1 0 0 1 0 1

Pseudomonas aeruginosa strain 178 287 WGS 178 SCWI01000000 182 7063068 hospital Italy "Human, Homo sapiens" Resistant Computational Prediction "aadA13,ant(2'')-Ia,aph(3')-Iib,fosA,crpP,blaPAO,blaOXA-50,qacE,catB7" 97.4 Good 1 1 0 1 1 1 1 0 0 1 0 1

Pseudomonas aeruginosa strain 171 287 WGS 171 SCWP01000000 129 6343678 hospital Italy "Human, Homo sapiens" Resistant Computational Prediction "crpP,aph(3')-IIb,catB7,blaOXA-50,blaPAO,fosA" 98.6 Good 1 1 0 1 1 1 1 0 0 0 0 1

Pseudomonas aeruginosa strain 165 287 WGS 165 SCWU01000000 243 7020770 hospital Italy "Human, Homo sapiens" Resistant Computational Prediction "aph(3')-IIb,catB7,blaOXA-488,blaPAO,fosA" 97.8 Good 1 1 0 1 1 1 1 0 0 1 0 0

Pseudomonas aeruginosa strain 162 287 WGS 162 SCWW01000000 98 6700969 hospital Italy "Human, Homo sapiens" Resistant Computational Prediction "fosA,aac(6')-Ib-cr,aac(3)-Ic,aph(3')-Iib,aac(6')-Ib-Hangzhou,sul1,blapAO,qacE,cmlA1,catB10,blaOXA-488,catB7" 98.7 Good 1 1 0 1 1 1 1 0 0 1 0 1

Pseudomonas aeruginosa strain 160 287 WGS 160 SCWX01000000 123 6985284 hospital Italy "Human, Homo sapiens" Resistant Computational Prediction "aadA13,aph(3')-Iib,ant(2'')-Ia,fosA,crpP,blapAO,blaOXA-50,qacE,catB7" 98.2 Good 1 1 0 1 1 1 1 0 0 1 0 1

Pseudomonas aeruginosa strain 158 287 WGS 158 SCWZ01000000 214 6911232 hospital Italy "Human, Homo sapiens" Resistant Computational Prediction "fosA,aph(3')-Iib,aac(3)-Ic,aac(6')-Ib-cr,aac(6')-Ib-Hangzhou,blaPAO,cmlA1,catB10,catB7,blaOXA-488" 97.1 Good 1 1 0 1 1 1 1 0 0 1 0 1

Pseudomonas aeruginosa strain 152 287 WGS 152 SCXF01000000 326 6595459 hospital Italy "Human, Homo sapiens" Resistant Computational Prediction "crpP,aph(3')-IIb,catB7,blaOXA-488,blaPAO,fosA" 95.4 Good 1 1 0 1 1 1 1 0 0 0 0 1

Pseudomonas aeruginosa strain 153 287 WGS 153 SCXE01000000 289 7143659 hospital Italy "Human, Homo sapiens" Resistant Computational Prediction "crpP,aph(3')-IIb,catB7,blaOXA-396,blaPAO,fosA" 96.6 Good 1 1 1 1 1 1 1 0 0 0 0 1

Pseudomonas aeruginosa strain 150 287 WGS 150 SCXG01000000 93 6928423 hospital Italy "Human, Homo sapiens" Resistant Computational Prediction "fosA,aph(3')-Iib,aac(6')-Ib-cr,aac(3)-Ic,aac(6')-Ib-Hangzhou,sul1,qacEc,lA1,catB10,catB7,blaPAO,blaOXA-488" 98.7 Good 1 1 0 1 1 1 1 0 0 1 0 1

Pseudomonas aeruginosa strain 149 287 WGS 149 SCXH01000000 105 6933131 hospital Italy "Human, Homo sapiens" Resistant Computational Prediction "fosA,aph(3')-Iib,aac(6')-Ib-cr,aac(3)-Ic,aac(6')-Ib-Hangzhou,sul1,qacEc,lA1,catB10,catB7,blaPAO,blaOXA-488" 98.8 Good 1 1 0 1 1 1 1 0 0 1 0 1

Pseudomonas aeruginosa strain 145 287 WGS 145 SCXK01000000 117 6923444 hospital Italy "Human, Homo sapiens" Resistant Computational Prediction "fosA,aph(3')-Iib,aac(6')-Ib-cr,aac(3)-Ic,aac(6')-Ib-Hangzhou,sul1,qacEc,lA1,catB10,catB7,blaPAO,blaOXA-488" 98.6 Good 1 1 0 1 1 1 1 0 0 1 0 1

Pseudomonas aeruginosa strain 143 287 WGS 143 SCXL01000000 134 6924494 hospital Italy "Human, Homo sapiens" Resistant Computational Prediction "fosA,aph(3')-Iib,aac(6')-Ib-cr,aac(3)-Ic,aac(6')-Ib-Hangzhou,sul1,qacEc,lA1,catB10,catB7,blaPAO,blaOXA-488" 98.4 Good 1 1 0 1 1 1 1 0 0 1 0 1

Pseudomonas aeruginosa AZPAE15023 287 WGS AZPAE15023 JTNX00000000 101 6351355 itra-abdominal tract infection Spain "Human, Homo sapiens" Resistant;Intermediate;Susceptible AMR Panel "crpP,fosA,catB7,blaPAO,blaOXA-50,blaOXA-494,blaOXA-396,aph(3')-Iib" 99.3 100 Good 1 1 1 1 1 1 1 0 0 0 0 1

Pseudomonas aeruginosa strain 135 287 WGS 135 SCXS01000000 472 6942179 hospital Italy "Human, Homo sapiens" Resistant Computational Prediction "fosA,aac(6')-Ib-cr,aac(6')-Ib3,aph(3')-Iib,crpP,blaVIM-1,blaPAO,blaOXA-50,qacE,catB7" 94.8 Good 1 1 1 1 1 1 1 0 0 0 0 1

Pseudomonas aeruginosa strain 132 287 WGS 132 SCXV01000000 493 6879817 hospital Italy "Human, Homo sapiens" Resistant Computational Prediction "aadA6,aac(6')-Il,aac(6')-31,aph(3')-Iib,fosA,sul1,blaPAO,blaOXA-488,blaOXA-2,qacE,catB7" 95.6 Good 1 1 0 1 1 1 1 0 0 1 0 0

Pseudomonas aeruginosa strain 177 287 WGS 177 SCWJ01000000 127 6752889 hospital Italy "Human, Homo sapiens" Resistant Computational Prediction "crpP,fosA,catB7,blaPAO,blaOXA-50,aph(3')-Iib" 97.9 Good 1 1 0 1 1 1 1 0 0 0 0 1

Pseudomonas aeruginosa AZPAE15022 287 WGS AZPAE15022 JTNY00000000 68 6219362 urinary tract infection France "Human, Homo sapiens" Susceptible;Resistant AMR Panel "fosA,aph(3')-Iib,aac(6')-Ib-cr,aac(3)-Ic,aac(6')-Ib-Hangzhou,sul1,qacEc,lA1,catB10,catB7,blaPAO,blaOXA-488" 99.3 99.7 0.3 Good 1 1 0 1 1 1 1 0 0 1 0 1

Pseudomonas aeruginosa strain 176 287 WGS 176 SCWK01000000 132 6930192 hospital Italy "Human, Homo sapiens" Resistant Computational Prediction "aph(3')-IIb,catB7,blaOXA-396,blaOXA-494,blaPAO,fosA" 98.5 Good 1 1 1 1 1 1 1 0 0 0 0 0

Pseudomonas aeruginosa strain 156 287 WGS 156 SCXB01000000 169 6358784 hospital Italy "Human, Homo sapiens" Resistant Computational Prediction "crpP,fosA,catB7,blaPAO,blaOXA-50,aph(3')-Iib" 99 Good 1 1 0 1 1 1 1 0 0 0 0 1

Pseudomonas aeruginosa strain 155 287 WGS 155 SCXC01000000 110 6793734 hospital Italy "Human, Homo sapiens" Resistant Computational Prediction "aadA6,aac(6')-Il,aac(6')-31,aph(3')-Iib,fosA,sul1,blaPAO,blaOXA-488,blaOXA-415,qacE,catB7" 98.2 Good 1 1 0 1 1 1 1 0 0 1 0 0

Pseudomonas aeruginosa strain 139 287 WGS 139 SCXP01000000 101 6839005 hospital Italy "Human, Homo sapiens" Resistant Computational Prediction "aadA6,aac(6')-Il,aac(6')-31,aph(3')-Iib,fosA,sul1,blaPAO,blaOXA-488,blaOXA-415,qacE,catB7" 98.7 Good 1 1 0 1 1 1 1 0 0 1 0 0

Pseudomonas aeruginosa strain 119 287 WGS 119 SCYE01000000 970 6503997 hospital Italy "Human, Homo sapiens" Resistant Computational Prediction "crpP,fosA,catB7,blaPAO,blaOXA-395,aph(3')-Iib" 91.2 Good 1 1 1 1 1 1 1 0 0 1 0 1

Pseudomonas aeruginosa AZPAE15020 287 WGS AZPAE15020 JTOA00000000 129 7163187 respiratory tract infection France "Human, Homo sapiens" Resistant;Intermediate;Susceptible AMR Panel "aph(3')-IIb,catB7,crpP,blaOXA-488,blaPAO,fosA" 98.3 99.7 Good 1 1 0 1 1 1 1 0 0 0 0 1

Pseudomonas aeruginosa strain 37 287 WGS 37 SEMM01000000 751 6822496 hospital Italy "Human, Homo sapiens" Resistant Computational Prediction "fosA,aadA6,aac(6')-31,aac(6')-Il,aph(3')-Iib,sul1,blaPAO,blaOXA-2,blaOXA-488,qace,catB7" 93.1 Good 1 1 0 1 1 1 1 0 0 0 0 0

Pseudomonas aeruginosa strain 80 287 WGS 80 SCZB01000000 420 6497590 hospital Italy "Human, Homo sapiens" Resistant Computational Prediction "fosA,aph(3')-Iib,aac(6')-Ib-Hangzhou,aac(3)-Ic,aac(6')-Ib-cr,sul1,blaPAO,blaOXA-488,qacE,cmlA1,catB7,catB10" 95.2 Good 1 1 0 1 1 1 1 0 0 1 0 1

Pseudomonas aeruginosa strain 21 287 WGS 21 SDAC01000000 316 6649023 hospital Italy "Human, Homo sapiens" Resistant Computational Prediction "fosA,aph(3')-Iib,aac(6')-Ib-Hangzhou,aac(6')-Ib-Suzhou,aac(3)-Ic,aac(6')-Ib-cr,sul1,blaPAO,blaOXA-488,qacE,cmlA1,catB7,catB10" 97.1 Good 1 1 0 1 1 1 1 0 0 1 0 0

Pseudomonas aeruginosa AZPAE15018 287 WGS AZPAE15018 JTOC00000000 81 6933997 itra-abdominal tract infection United States "Human, Homo sapiens" Susceptible;Resistant AMR Panel "crpP,fosA,catB7,blaPAO,blaOXA-494,blaOXA-396,aph(3')-Iib" 99 100 Good 1 1 1 1 1 1 1 0 0 0 0 1

Pseudomonas aeruginosa strain 110 287 WGS 110 SCYM01000000 663 6815484 hospital Italy "Human, Homo sapiens" Resistant Computational Prediction "fosA,aadA6,aph(3')-Iib,aac(6')-Il,aac(6')-31,sul1,blaPAO,blaOXA-488,blaOXA-415,qacE,catB7" 93.6 Good 1 1 0 1 1 1 1 0 0 1 0 0

Pseudomonas aeruginosa strain 102 287 WGS 102 SCYQ01000000 912 6667845 hospital Italy "Human, Homo sapiens" Resistant Computational Prediction "fosA,aph(3')-Iib,ant(2'')-Ia,crpP,blaPAO,catB7,blaOXA-50,qacE," 91.9 Good 1 1 0 1 1 1 1 0 0 1 0 1

Pseudomonas aeruginosa strain 105 287 WGS 105 SCYP01000000 749 6855732 hospital Italy "Human, Homo sapiens" Resistant Computational Prediction "aadA13,fosA,aph(3')-Iib,ant(2'')-Ia,crpP,blaPAO,catB7,blaOXA-50,qacE," 93.5 Good 1 1 0 1 1 1 1 0 0 1 0 1

Pseudomonas aeruginosa strain 96 287 WGS 96 SCYU01000000 534 6899441 hospital Italy "Human, Homo sapiens" Resistant Computational Prediction "aadA13,sul1,fosA,aph(3')-Iib,ant(2'')-Ia,crpP,blaPAO,catB7,blaOXA-50,qacE," 95.1 Good 1 1 0 1 1 1 1 0 0 1 0 1

Pseudomonas aeruginosa strain 70 287 WGS 70 SCZE01000000 649 6834463 hospital Italy "Human, Homo sapiens" Resistant Computational Prediction "fosA,aac(6')-Ib-cr,aph(3')-Iib,aac(6')-Ib-Hangzhou,crpP,sul1,blaPAO,blaOXA-488,qacE,catB7,catB10" 94 Good 1 1 0 1 1 1 1 0 0 1 0 1

Pseudomonas aeruginosa strain 64 287 WGS 64 SCZH01000000 723 6547758 hospital Italy "Human, Homo sapiens" Resistant Computational Prediction "fosA,aph(3')-Iib,aac(3)-Ic,aac(6')-Ib-Hangzhou,aac(6')-Ib-cr,sul1,blaOXA-488,blaPAO,catB7,cmlA1,catB10" 92.4 Good 1 1 0 1 1 1 1 0 0 1 0 1

Pseudomonas aeruginosa strain 54 287 WGS 54 SCZJ01000000 975 6755179 hospital Italy "Human, Homo sapiens" Resistant Computational Prediction "aadA13,fosA,ant(2'')-Ia,aph(3')-Iib,crpP,blaPAO,blaOXA-50,qacE" 89.6 Good 1 1 0 1 1 1 1 0 0 1 0 1

Pseudomonas aeruginosa AZPAE15016 287 WGS AZPAE15016 JTOE00000000 161 6756824 urinary tract infection United States "Human, Homo sapiens" Susceptible;Intermediate AMR Panel "aph(3')-IIb,crpP,fosA,catB7,blaPAO,blaOXA-488" 98.7 99.1 0.3 Good 1 1 0 1 1 1 1 0 0 0 0 1

Pseudomonas aeruginosa strain 38 287 WGS 38 SCZS01000000 693 6846187 hospital Italy "Human, Homo sapiens" Resistant Computational Prediction "aadA6,aph(3')-Iib,aac(6')-Il,aac(6')-31,fosA,sul1,blaPAO,blaOXA-488,blaOXA-2,qacE,catB7" 94 Good 1 1 0 1 1 1 1 0 0 1 0 0

Pseudomonas aeruginosa strain 22 287 WGS 22 SDAB01000000 723 6931430 hospital Italy "Human, Homo sapiens" Resistant Computational Prediction "aph(3')-IIb,crpP,fosA,catB7,blaPAO,blaOXA-395,aac(6')-Ib-cr,aac(6')-Ib-Hangzhou," 93.3 Good 1 1 1 1 1 1 1 0 0 0 0 1

Pseudomonas aeruginosa AZPAE15015 287 WGS AZPAE15015 JTOF00000000 113 6804229 urinary tract infection Germany "Human, Homo sapiens" Resistant AMR Panel "fosA,aadA6,aph(3')-Iib,aac(6')-31,aac(6')-Il,sul1,blaPAO,blaOXA-488,blaOXA-2,qacE,catB7" 98.5 100 Good 1 1 0 1 1 1 1 0 0 1 0 0

Pseudomonas aeruginosa strain 20 287 WGS 20 SDAD01000000 433 6832411 hospital Italy "Human, Homo sapiens" Resistant Computational Prediction "aadA6,aph(3')-Iib,fosA,sul1,blaPAO,blaOXA-488,qacE,catB7," 95.6 Good 1 1 0 1 1 1 1 0 0 1 0 0

Pseudomonas aeruginosa strain 13 287 WGS 13 SDAH01000000 393 6596361 hospital Italy "Human, Homo sapiens" Resistant Computational Prediction "aph(3')-IIb,crpP,fosA,catB7,blaPAO,blaOXA-486" 96.9 Good 1 1 0 1 1 1 1 0 0 0 0 1

Pseudomonas aeruginosa AZPAE15011 287 WGS AZPAE15011 JTOJ00000000 120 6590340 itra-abdominal tract infection Spain "Human, Homo sapiens" Intermediate;Susceptible AMR Panel "fosA,aph(6)-Id,aph(3')-Iib,aph(3'')-Ib,aadA6,crpP,sul1,blaPAO,blaOXA-488,qacE,catB7" 98.4 98.8 Good 1 1 0 1 1 1 1 0 0 0 0 1

Pseudomonas aeruginosa strain DMC-27b 287 WGS DMC-27b SMRY02000000 649 6958186 urine Bangladesh "Human, Homo sapiens" Resistant Computational Prediction "fosA,aph(3')-Iib,aac(6')-Il,aac(6')-31,aph(3')-VI,aadA6,sul1,crpP,tet(A),tet(G),blaPAO,blaVEB-1,blaVIM-5,blaOXA-2,blaOXA-488,qacE,catB7" 93.1 Good 1 1 1 1 1 1 1 0 1 1 0 1

Pseudomonas aeruginosa strain AES1M 287 Complete AES1M CP037925 1 6373139 Cystic Fibrosis lung sputum Australia "Human, Homo sapiens" Resistant Computational Prediction "aph(3')-IIb,blaOXA-50,blaOXA-396,fosA,catB7,blaPAO,blaOXA-494" 99.1 Good 1 1 1 1 1 1 1 0 0 0 0 0

Pseudomonas aeruginosa strain AES1R 287 Complete AES1R CP037926 1 6373893 Cystic Fibrosis lung sputum Australia "Human, Homo sapiens" Resistant Computational Prediction "aph(3')-IIb,blaOXA-50,blaOXA-396,fosA,catB7,blaPAO,blaOXA-494" 98.9 Good 1 1 1 1 1 1 1 0 0 0 0 0

Pseudomonas aeruginosa strain 20 287 WGS 20 RWLA01000000 30 6415050 Sputum Canada "Human, Homo sapiens" Resistant Computational Prediction "aph(3')-IIb,blaOXA-395,fosA,catB7,blaPAO," 99.1 Good 1 1 1 1 1 1 1 0 0 0 0 0

Pseudomonas aeruginosa strain 18 287 WGS 18 RWLC01000000 43 6327698 Sputum Canada "Human, Homo sapiens" Resistant Computational Prediction "aph(3')-IIb,blaOXA-486,fosA,catB7,blaPAO," 99.5 Good 1 1 0 1 1 1 1 0 0 0 0 0

Pseudomonas aeruginosa strain 17 287 WGS 17 RWLD01000000 38 6995916 Sputum Canada "Human, Homo sapiens" Resistant Computational Prediction "aph(3')-IIb,crpP,blaOXA-396,fosA,catB7,blaPAO," 97.3 Good 1 1 1 1 1 1 1 0 0 0 0 1

Pseudomonas aeruginosa strain 16 287 WGS 16 RWLE01000000 30 6433177 Sputum Canada "Human, Homo sapiens" Resistant Computational Prediction "aph(3')-IIb,crpP,blaOXA-396,fosA,catB7,blaPAO," 98.2 Good 1 1 1 1 1 1 1 0 0 0 0 1

Pseudomonas aeruginosa strain 13 287 WGS 13 RWLI01000000 63 6815273 Sputum Canada "Human, Homo sapiens" Resistant Computational Prediction "aph(3')-IIb,blaOXA-488,fosA,catB7,blaPAO," 98.7 Good 1 1 0 1 1 1 1 0 0 0 0 0

Pseudomonas aeruginosa strain 8 287 WGS 8 RWLN01000000 52 6316805 Sputum Canada "Human, Homo sapiens" Resistant Computational Prediction "aph(3')-IIb,blaOXA-395,fosA,catB7,blaPAO," 99 Good 1 1 1 1 1 1 1 0 0 0 0 0

Pseudomonas aeruginosa strain 6 287 WGS 6 RWLP01000000 37 6404090 Sputum Canada "Human, Homo sapiens" Resistant Computational Prediction "aph(3')-IIb,crpP,blaOXA-486,fosA,catB7,blaPAO," 99.2 Good 1 1 0 1 1 1 1 0 0 0 0 1

Pseudomonas aeruginosa strain 1 287 WGS 1 RWLU01000000 27 6402434 Sputum Canada "Human, Homo sapiens" Resistant Computational Prediction "aph(3')-IIb,crpP,blaOXA-486,fosA,catB7,blaPAO," 99.3 Good 1 1 0 1 1 1 1 0 0 0 0 1

Pseudomonas aeruginosa AZPAE15009 287 WGS AZPAE15009 JTOL00000000 107 6958019 respiratory tract infection Spain "Human, Homo sapiens" Resistant AMR Panel "crpP,aph(3')-IIb,blaOXA-396,fosA,catB7,blaPAO,blaOXA-494" 98.9 100 Good 1 1 1 1 1 1 1 0 0 0 0 1

Pseudomonas aeruginosa strain 4104355315 287 WGS 4104355315 RWLY01000000 25 6836271 Sputum Canada "Human, Homo sapiens" Resistant Computational Prediction "crpP,aph(3')-IIb,blaOXA-396,fosA,catB7,blaPAO" 98 Good 1 1 1 1 1 1 1 0 0 0 0 1

Pseudomonas aeruginosa AZPAE15008 287 WGS AZPAE15008 JTOM00000000 107 6345734 urinary tract infection Spain "Human, Homo sapiens" Resistant;Susceptible AMR Panel "crpP,aph(3')-IIb,blaOXA-396,fosA,catB7,blaPAO" 99 99.1 Good 1 1 1 1 1 1 1 0 0 1 0 1

Pseudomonas aeruginosa strain 5024382749-14 287 WGS 5024382749-14 RWMM01000000 83 6341946 Sputum Canada "Human, Homo sapiens" Resistant Computational Prediction "crpP,blaOXA-396,fosA,catB7,blaPAO" 98.3 Good 1 1 1 0 1 1 1 0 0 0 0 1

Pseudomonas aeruginosa AZPAE15007 287 WGS AZPAE15007 JTON00000000 121 6814810 urinary tract infection Spain "Human, Homo sapiens" Resistant AMR Panel "crpP,aph(3')-IIb,blaOXA-396,fosA,catB7,blaPAO,blaOXA-494" 98.4 100 Good 1 1 1 1 1 1 1 0 0 0 0 1

Pseudomonas aeruginosa strain 4094345258 287 WGS 4094345258 RWNG01000000 56 6911040 Throat Canada "Human, Homo sapiens" Resistant Computational Prediction "crpP,aph(3')-IIb,blaOXA-396,fosA,catB7,blaPAO" 98.7 Good 1 1 1 1 1 1 1 0 0 0 0 1

Pseudomonas aeruginosa strain 5054407658-16 287 WGS 5054407658-16 RWNL01000000 36 6747385 Sputum Canada "Human, Homo sapiens" Resistant Computational Prediction "crpP,aph(3')-IIb,blaOXA-488,fosA,catB7,blaPAO" 98.3 Good 1 1 0 1 1 1 1 0 0 0 0 1

Pseudomonas aeruginosa strain 5024379144-15 287 WGS 5024379144-15 RWNH01000000 78 6919408 Throat Canada "Human, Homo sapiens" Resistant Computational Prediction "crpP,aph(3')-IIb,blaOXA-396,fosA,catB7,blaPAO" 98.4 Good 1 1 1 1 1 1 1 0 0 0 0 1

Pseudomonas aeruginosa strain 5054407658-17 287 WGS 5054407658-17 RWNK01000000 33 6972220 Sputum Canada "Human, Homo sapiens" Resistant Computational Prediction "crpP,aph(3')-IIb,blaOXA-488,fosA,catB7,blaPAO" 98.4 Good 1 1 0 1 1 1 1 0 0 0 0 1

Pseudomonas aeruginosa AZPAE15006 287 WGS AZPAE15006 JTOO00000000 109 6832413 respiratory tract infection United States "Human, Homo sapiens" Resistant AMR Panel "aph(3')-IIb,blaOXA-395,fosA,catB7,blaPAO" 98.4 99.1 0.6 Good 1 1 1 1 1 1 1 0 0 0 0 0

Pseudomonas aeruginosa strain 6098 287 WGS 6098 RWNP01000000 44 6332802 Sputum Canada "Human, Homo sapiens" Resistant Computational Prediction "aadA6,aph(3')-Iib,fosA,sul1,tet(G),catB7,blaOXA-488,blaPAO,qacE" 99 Good 1 1 0 1 1 1 1 0 1 1 0 1

Pseudomonas aeruginosa strain 6097.3 287 WGS 6097.3 RWNQ01000000 38 6338989 Sputum Canada "Human, Homo sapiens" Resistant Computational Prediction "aph(3')-IIb,blaOXA-395,fosA,catB7,blaPAO" 99.3 Good 1 1 1 1 1 1 1 0 0 0 0 0

Pseudomonas aeruginosa strain 6097.1 287 WGS 6097.1 RWNR01000000 36 6336017 Sputum Canada "Human, Homo sapiens" Resistant Computational Prediction "aph(3')-IIb,blaOXA-395,fosA,catB7,blaPAO" 99.3 Good 1 1 1 1 1 1 1 0 0 0 0 0

Pseudomonas aeruginosa strain 12 287 WGS 12 RWLJ01000000 37 6404318 Sputum Canada "Human, Homo sapiens" Resistant Computational Prediction "crpP,aph(3')-IIb,blaOXA-486,fosA,catB7,blaPAO" 99.3 Good 1 1 0 1 1 1 1 0 0 0 0 1

Pseudomonas aeruginosa AZPAE15005 287 WGS AZPAE15005 JTOP00000000 97 6889490 respiratory tract infection United States "Human, Homo sapiens" Resistant AMR Panel "crpP,aph(3')-IIb,blaOXA-396,fosA,catB7,blaPAO,blaOXA-494" 98.2 99.4 Good 1 1 1 1 1 1 1 0 0 0 0 1

Pseudomonas aeruginosa strain 4 287 WGS 4 RWLR01000000 24 6434446 Sputum Canada "Human, Homo sapiens" Resistant Computational Prediction "crpP,aph(3')-IIb,blaOXA-488,fosA,catB7,blaPAO" 98.9 Good 1 1 0 1 1 1 1 0 0 1 0 1

Pseudomonas aeruginosa strain 5024379144-14 287 WGS 5024379144-14 RWNI01000000 85 6927033 Throat Canada "Human, Homo sapiens" Resistant Computational Prediction "crpP,aph(3')-IIb,blaOXA-396,fosA,catB7,blaPAO" 98.2 Good 1 1 1 1 1 1 1 0 0 0 0 1

Pseudomonas aeruginosa strain 41004355322 287 WGS 41004355322 RWNJ01000000 44 6972769 Throat Canada "Human, Homo sapiens" Resistant Computational Prediction "crpP,aph(3')-IIb,blaOXA-488,fosA,catB7,blaPAO" 98.3 Good 1 1 0 1 1 1 1 0 0 0 0 1

Pseudomonas aeruginosa strain 6093 287 WGS 6093 RWNU01000000 39 6347605 Sputum Canada "Human, Homo sapiens" Resistant Computational Prediction "aph(3')-IIb,blaOXA-486,fosA,catB7,blaPAO" 98.8 Good 1 1 0 1 1 1 1 0 0 0 0 0

Pseudomonas aeruginosa strain 6000.2 287 WGS 6000.2 RWOJ01000000 45 6779313 Sputum Canada "Human, Homo sapiens" Resistant Computational Prediction "crpP,aph(3')-IIb,blaOXA-396,fosA,catB7,blaPAO" 98.6 Good 1 1 1 1 1 1 1 0 0 0 0 1

Pseudomonas aeruginosa strain 6000.1 287 WGS 6000.1 RWOK01000000 41 6778852 Sputum Canada "Human, Homo sapiens" Resistant Computational Prediction "crpP,aph(3')-IIb,blaOXA-396,fosA,blaPAO" 98.7 Good 1 1 1 1 1 1 0 0 0 0 0 1

Pseudomonas aeruginosa strain 5999.2 287 WGS 5999.2 RWOM01000000 64 6909742 Sputum Canada "Human, Homo sapiens" Resistant Computational Prediction "crpP,aph(3')-IIb,ant(2'')-Ia,sul1,aadA2b,cmlA1,qacEblaOXA-396,fosA,catB7,blaPAO" 98.4 Good 1 1 1 1 1 1 1 0 0 1 0 1

Pseudomonas aeruginosa strain 5993 287 WGS 5993 RWOT01000000 44 6793482 Sputum Canada "Human, Homo sapiens" Resistant Computational Prediction "crpP,aph(3')-IIb,blaOXA-396,fosA,catB7,blaPAO" 98.5 Good 1 1 1 1 1 1 1 0 0 0 0 1

Pseudomonas aeruginosa AZPAE15001 287 WGS AZPAE15001 JTOT00000000 75 6582180 itra-abdominal tract infection Colombia "Human, Homo sapiens" Resistant;Susceptible AMR Panel "aph(3')-IIb,blaOXA-396,fosA,catB7,blaPAO,blaOXA-494" 99 100 0.3 Good 1 1 1 1 1 1 1 0 0 0 0 0

Pseudomonas aeruginosa strain 5989 287 WGS 5989 RWOZ01000000 40 6753910 Sputum Canada "Human, Homo sapiens" Resistant Computational Prediction "crpP,aph(3')-IIb,blaOXA-50,fosA,catB7,blaPAO" 98.1 Good 1 1 0 1 1 1 1 0 0 0 0 1

Pseudomonas aeruginosa strain 5985 287 WGS 5985 RWPD01000000 42 6603436 Sputum Canada "Human, Homo sapiens" Resistant Computational Prediction "crpP,aph(3')-IIb,blaOXA-50,fosA,catB7,blaPAO" 98.2 Good 1 1 0 1 1 1 1 0 0 0 0 1

Pseudomonas aeruginosa strain 6000.3 287 WGS 6000.3 RWOI01000000 48 6848676 Sputum Canada "Human, Homo sapiens" Resistant Computational Prediction "crpP,aph(4)-Ia,aac(3)-IV,aph(3')-Iib,fosA,blaPAO,blaOXA-396,tet(A)," 98.6 Good 1 1 1 1 1 1 0 0 1 1 0 1

Pseudomonas aeruginosa strain 5999.3 287 WGS 5999.3 RWOL01000000 55 6948779 Sputum Canada "Human, Homo sapiens" Resistant Computational Prediction "fosA,aadA2b,aph(3')-Iib,ant(2'')-Ia,crpP,sul1,blaPAO,blaOXA-396,cmlA1,qacE,catB7" 98.6 Good 1 1 1 1 1 1 1 0 0 1 0 1

Pseudomonas aeruginosa strain PA-CL532 287 WGS PA-CL532 RWPL01000000 23 6988055 Hospital sink Canada Resistant Computational Prediction "crpP,aph(3')-IIb,blaOXA-396,fosA,catB7,blaPAO" 98.8 Good 1 1 1 1 1 1 1 0 0 0 0 1

Pseudomonas aeruginosa strain PA-CL520 287 WGS PA-CL520 RWPT01000000 25 7030507 Hospital sink Canada Resistant Computational Prediction "crpP,aph(3')-IIb,blaOXA-396,fosA,catB7,blaPAO" 98.8 Good 1 1 1 1 1 1 1 0 0 0 0 1

Pseudomonas aeruginosa strain PA-CL519 287 WGS PA-CL519 RWPU01000000 21 7024063 Hospital sink Canada Resistant Computational Prediction "crpP,aph(3')-IIb,blaOXA-396,fosA,catB7,blaPAO" 98.8 Good 1 1 1 1 1 1 1 0 0 0 0 1

Pseudomonas aeruginosa strain PA-CL512 287 WGS PA-CL512 RWQB01000000 17 6958739 Hospital sink Canada Resistant Computational Prediction "aadA6,fosA,sul1,catB7,blaPAO,blaOXA-396,qacE,aph(3')-Iib" 98.9 Good 1 1 1 1 1 1 1 0 0 0 0 1

Pseudomonas aeruginosa strain PA-CL511 287 WGS PA-CL511 RWQC01000000 18 6961613 Hospital sink Canada Resistant Computational Prediction "aadA6,fosA,sul1,catB7,blaPAO,blaOXA-396,qacE,aph(3')-Iib" 98.9 Good 1 1 1 1 1 1 1 0 0 0 0 1

Pseudomonas aeruginosa strain PA-CL510 287 WGS PA-CL510 RWQD01000000 17 6963155 Hospital sink Canada Resistant Computational Prediction "aadA6,fosA,sul1,catB7,blaPAO,blaOXA-396,qacE,aph(3')-Iib" 98.9 Good 1 1 1 1 1 1 1 0 0 0 0 1

Pseudomonas aeruginosa strain PA-CL518 287 WGS PA-CL518 RWPV01000000 19 7028181 Hospital sink Canada Resistant Computational Prediction "crpP,aph(3')-IIb,blaOXA-396,fosA,catB7,blaPAO" 98.7 Good 1 1 1 1 1 1 1 0 0 0 0 1

Pseudomonas aeruginosa strain PA-CL517 287 WGS PA-CL517 RWPW01000000 33 7033028 Hospital sink Canada Resistant Computational Prediction "crpP,aph(3')-IIb,blaOXA-396,fosA,catB7,blaPAO" 98.7 Good 1 1 1 1 1 1 1 0 0 0 0 1

Pseudomonas aeruginosa strain PA-CL513 287 WGS PA-CL513 RWQA01000000 14 6716548 Hospital sink Canada Resistant Computational Prediction "crpP,aph(3')-IIb,blaOXA-488,fosA,catB7,blaPAO" 99.3 Good 1 1 0 1 1 1 1 0 0 0 0 1

Pseudomonas aeruginosa strain PA-CL509 287 WGS PA-CL509 RWQE01000000 18 6958357 Hospital sink Canada Resistant Computational Prediction "aadA6,fosA,sul1,catB7,blaPAO,blaOXA-396,qacE,aph(3')-Iib" 98.9 Good 1 1 1 1 1 1 1 0 0 0 0 1

Pseudomonas aeruginosa strain PAC91A 287 WGS PAC91A RWQZ01000000 37 6781582 Sputum Canada "Human, Homo sapiens" Resistant Computational Prediction "crpP,aph(3')-IIb,blaOXA-396,fosA,catB7,blaPAO" 98.8 Good 1 1 1 1 1 1 1 0 0 0 0 1

Pseudomonas aeruginosa strain PAC81B 287 WGS PAC81B RWRA01000000 45 6663122 Sputum Canada "Human, Homo sapiens" Resistant Computational Prediction "crpP,aph(3')-IIb,blaOXA-396,fosA,catB7,blaPAO" 98.9 Good 1 1 1 1 1 1 1 0 0 0 0 1

Pseudomonas aeruginosa strain PAC127B 287 WGS PAC127B RWRF01000000 34 6319076 Sputum Canada "Human, Homo sapiens" Resistant Computational Prediction "crpP,aph(3')-IIb,blaOXA-488,fosA,catB7,blaPAO" 98.7 Good 1 1 0 1 1 1 1 0 0 0 0 1

Pseudomonas aeruginosa strain PAC38A 287 WGS PAC38A RWRI01000000 28 6280595 Sputum Canada "Human, Homo sapiens" Resistant Computational Prediction "crpP,aph(3')-IIb,blaOXA-488,fosA,catB7,blaPAO" 98.9 Good 1 1 0 1 1 1 1 0 0 0 0 1

Pseudomonas aeruginosa strain PAC98D 287 WGS PAC98D RWRJ01000000 27 6487583 Sputum Canada "Human, Homo sapiens" Resistant Computational Prediction "aph(3')-IIb,blaOXA-396,fosA,catB7,blaPAO" 99.2 Good 1 1 1 1 1 1 1 0 0 0 0 0

Pseudomonas aeruginosa strain PAC80A 287 WGS PAC80A RWRN01000000 30 6488535 Nasopharynx Canada "Human, Homo sapiens" Resistant Computational Prediction "aph(3')-IIb,blaOXA-396,fosA,catB7,blaPAO" 99.2 Good 1 1 1 1 1 1 1 0 0 0 0 0

Pseudomonas aeruginosa AZPAE14994 287 WGS AZPAE14994 JTPA00000000 156 6476787 itra-abdominal tract infection Germany "Human, Homo sapiens" Resistant;Susceptible AMR Panel "aph(3')-IIb,blaOXA-396,fosA,catB7,blaPAO" 97.6 97.9 0.3 Good 1 1 1 1 1 1 1 0 0 0 0 0

Pseudomonas aeruginosa strain PAC70B 287 WGS PAC70B RWRO01000000 28 6488042 Nasopharynx Canada "Human, Homo sapiens" Resistant Computational Prediction "crpP,aph(3')-IIb,blaOXA-50,fosA,catB7,blaPAO" 99.2 Good 1 1 0 1 1 1 1 0 0 0 0 1

Pseudomonas aeruginosa strain PAC31B 287 WGS PAC31B RWRR01000000 26 6488203 Sputum Canada "Human, Homo sapiens" Resistant Computational Prediction "aph(3')-IIb,blaOXA-396,fosA,catB7,blaPAO" 99.2 Good 1 1 1 1 1 1 1 0 0 0 0 0

Pseudomonas aeruginosa strain PAC70A 287 WGS PAC70A RWRP01000000 27 6487070 Nasopharynx Canada "Human, Homo sapiens" Resistant Computational Prediction "aph(3')-IIb,blaOXA-396,fosA,catB7,blaPAO" 99.2 Good 1 1 1 1 1 1 1 0 0 0 0 0

Pseudomonas aeruginosa strain PAC94A 287 WGS PAC94A RWSB01000000 58 6678836 Nasopharynx Canada "Human, Homo sapiens" Resistant Computational Prediction "aph(3')-IIb,blaOXA-488,fosA,catB7,blaPAO" 98.6 Good 1 1 0 1 1 1 1 0 0 1 0 0

Pseudomonas aeruginosa AZPAE14993 287 WGS AZPAE14993 JTPB00000000 223 6914857 urinary tract infection Spain "Human, Homo sapiens" Susceptible;Resistant AMR Panel "aph(3')-IIb,blaOXA-396,fosA,catB7,blaPAO,blaOXA-494" 98.2 99.4 Good 1 1 1 1 1 1 1 0 0 0 0 1

Pseudomonas aeruginosa strain PAC17A 287 WGS PAC17A RWSM01000000 38 6309437 Sputum Canada "Human, Homo sapiens" Resistant Computational Prediction "aph(3')-IIb,blaOXA-486,fosA,catB7,blaPAO" 98.8 Good 1 1 0 1 1 1 1 0 0 0 0 0

Pseudomonas aeruginosa strain PAC56A 287 WGS PAC56A RWSL01000000 35 6287159 Sputum Canada "Human, Homo sapiens" Resistant Computational Prediction "aph(3')-IIb,blaOXA-486,fosA,catB7,blaPAO" 98.8 Good 1 1 0 1 1 1 1 0 0 0 0 0

Pseudomonas aeruginosa strain PAC15B 287 WGS PAC15B RWSN01000000 54 6707813 Sputum Canada "Human, Homo sapiens" Resistant Computational Prediction "crpP,aph(3')-IIb,blaOXA-485,fosA,catB7,blaPAO" 97.9 Good 1 1 0 1 1 1 1 0 0 0 0 1

Pseudomonas aeruginosa strain PAC15A 287 WGS PAC15A RWSO01000000 49 6706713 Sputum Canada "Human, Homo sapiens" Resistant Computational Prediction "crpP,aph(3')-IIb,blaOXA-485,fosA,catB7,blaPAO" 98 Good 1 1 0 1 1 1 1 0 0 0 0 1

Pseudomonas aeruginosa strain PAC81A 287 WGS PAC81A RWRB01000000 76 6679635 Sputum Canada "Human, Homo sapiens" Resistant Computational Prediction "crpP,aph(3')-IIb,blaOXA-396,fosA,catB7,blaPAO" 98.6 Good 1 1 1 1 1 1 1 0 0 0 0 1

Pseudomonas aeruginosa strain PAC38B 287 WGS PAC38B RWRH01000000 27 6281608 Sputum Canada "Human, Homo sapiens" Resistant Computational Prediction "aph(3')-IIb,blaOXA-486,fosA,catB7,blaPAO" 99 Good 1 1 0 1 1 1 1 0 0 0 0 0

Pseudomonas aeruginosa strain PAC127A 287 WGS PAC127A RWRG01000000 21 6284126 Sputum Canada "Human, Homo sapiens" Resistant Computational Prediction "aph(3')-IIb,blaOXA-486,fosA,catB7,blaPAO" 98.7 Good 1 1 0 1 1 1 1 0 0 0 0 0

Pseudomonas aeruginosa strain PAC98B 287 WGS PAC98B RWRL01000000 25 6487905 Sputum Canada "Human, Homo sapiens" Resistant Computational Prediction "aph(3')-IIb,blaOXA-396,fosA,catB7,blaPAO" 99.2 Good 1 1 1 1 1 1 1 0 0 0 0 0

Pseudomonas aeruginosa strain PAC98C 287 WGS PAC98C RWRK01000000 28 6625618 Sputum Canada "Human, Homo sapiens" Resistant Computational Prediction "crpP,aph(3')-IIb,blaOXA-396,fosA,catB7,blaPAO" 99 Good 1 1 1 1 1 1 1 0 0 0 0 1

Pseudomonas aeruginosa strain PAC42A 287 WGS PAC42A RWRQ01000000 32 6628616 Nasopharynx Canada "Human, Homo sapiens" Resistant Computational Prediction "crpP,aph(3')-IIb,blaOXA-396,fosA,catB7,blaPAO" 99 Good 1 1 1 1 1 1 1 0 0 0 0 1

Pseudomonas aeruginosa strain PAC56B 287 WGS PAC56B RWSK01000000 37 6307452 Sputum Canada "Human, Homo sapiens" Resistant Computational Prediction "aph(3')-IIb,blaOXA-486,fosA,catB7,blaPAO" 98.8 Good 1 1 0 1 1 1 1 0 0 0 0 0

Pseudomonas aeruginosa AZPAE14989 287 WGS AZPAE14989 JTPF00000000 76 6549370 itra-abdominal tract infection China "Human, Homo sapiens" Resistant AMR Panel "aph(3')-IIb,blaOXA-488,blaOXA-485,blaOXA-50,fosA,catB7,blaPAO" 99.2 100 Good 1 1 0 1 1 1 1 0 0 0 0 0

Pseudomonas aeruginosa strain PAC61A 287 WGS PAC61A RWTK01000000 24 6501365 Sputum Canada "Human, Homo sapiens" Resistant Computational Prediction "crpP,aph(3')-IIb,blaOXA-396,fosA,catB7,blaPAO,blaOXA-494" 98.4 Good 1 1 1 1 1 1 1 0 0 0 0 1

Pseudomonas aeruginosa strain 320S290711BSL_PA2 287 WGS 320S290711BSL_PA2 RWTT01000000 71 6939580 Sputum Canada "Human, Homo sapiens" Resistant Computational Prediction "crpP,aph(3')-IIb,blaOXA-396,fosA,catB7,blaPAO" 98.7 Good 1 1 1 1 1 1 1 0 0 0 0 1

Pseudomonas aeruginosa strain 293S080611BSL_PA2 287 WGS 293S080611BSL_PA2 RWUB01000000 42 6426421 Sputum Canada "Human, Homo sapiens" Resistant Computational Prediction "crpP,aph(3')-IIb,blaOXA-395,blaOXA-488,blaOXA-485,blaOXA-50,fosA,catB7,blaPAO" 98.7 Good 1 1 1 1 1 1 1 0 0 0 0 1

Pseudomonas aeruginosa strain 295s071211BSL_PA1 287 WGS 295s071211BSL_PA1 RWUA01000000 49 6423108 Sputum Canada "Human, Homo sapiens" Resistant Computational Prediction "crpP,aph(3')-IIb,blaOXA-395,blaOXA-488,blaOXA-485,blaOXA-50,fosA,catB7,blaPAO" 98.7 Good 1 1 1 1 1 1 1 0 0 0 0 1

Pseudomonas aeruginosa strain PAC46A 287 WGS PAC46A RWTL01000000 27 6504440 Sputum Canada "Human, Homo sapiens" Resistant Computational Prediction "crpP,aph(3')-IIb,blaOXA-396,fosA,catB7,blaPAO,blaOXA-494" 98.3 Good 1 1 1 1 1 1 1 0 0 0 0 1

Pseudomonas aeruginosa strain PAC5B 287 WGS PAC5B RWTM01000000 22 6503267 Sputum Canada "Human, Homo sapiens" Resistant Computational Prediction "crpP,aph(3')-IIb,blaOXA-396,fosA,catB7,blaPAO,blaOXA-494" 98.3 Good 1 1 1 1 1 1 1 0 0 0 0 1

Pseudomonas aeruginosa AZPAE14987 287 WGS AZPAE14987 JTPH00000000 121 6855155 respiratory tract infection Germany "Human, Homo sapiens" Resistant AMR Panel "aph(3')-IIb,blaOXA-488,blaOXA-485,blaOXA-50,fosA,catB7,blaPAO" 98.4 99.7 Good 1 1 0 1 1 1 1 0 0 0 0 0

Pseudomonas aeruginosa strain PAC5A 287 WGS PAC5A RWTN01000000 31 6545027 Sputum Canada "Human, Homo sapiens" Resistant Computational Prediction "crpP,aph(3')-IIb,blaOXA-396,fosA,catB7,blaPAO,blaOXA-494" 97.7 Good 1 1 1 1 1 1 1 0 0 0 0 1

Pseudomonas aeruginosa strain 320S290711BSL_PA1 287 WGS 320S290711BSL_PA1 RWTU01000000 62 6954916 Sputum Canada "Human, Homo sapiens" Resistant Computational Prediction "crpP,aph(3')-IIb,blaOXA-396,fosA,catB7,blaPAO" 98.8 Good 1 1 1 1 1 1 1 0 0 0 0 1

Pseudomonas aeruginosa strain 313s141011BSL_PA1 287 WGS 313s141011BSL_PA1 RWTX01000000 35 6384539 Sputum Canada "Human, Homo sapiens" Resistant Computational Prediction "aph(3')-IIb,blaOXA-396,fosA,catB7,blaPAO" 98.7 Good 1 1 1 1 1 1 1 0 0 0 0 0

Pseudomonas aeruginosa strain 293S080611BSL_PA1 287 WGS 293S080611BSL_PA1 RWUC01000000 34 6337735 Sputum Canada "Human, Homo sapiens" Resistant Computational Prediction "aph(3')-IIb,blaOXA-395,blaOXA-488,blaOXA-485,blaOXA-50,fosA,catB7,blaPAO" 99 Good 1 1 1 1 1 1 1 0 0 0 0 0

Pseudomonas aeruginosa strain 201s070911bsl_PA2 287 WGS 201s070911bsl_PA2 RWUL01000000 51 6814164 Sputum Canada "Human, Homo sapiens" Resistant Computational Prediction "crpP,aph(3')-IIb,blaOXA-50,fosA,catB7,blaPAO" 98.2 Good 1 1 0 1 1 1 1 0 0 0 0 1

Pseudomonas aeruginosa strain 197S020911BSL_PA4 287 WGS 197S020911BSL_PA4 RWUN01000000 39 6228253 Sputum Canada "Human, Homo sapiens" Resistant Computational Prediction "aph(3')-IIb,blaOXA-486,fosA,catB7,blaPAO" 99 Good 1 1 0 1 1 1 1 0 0 0 0 0

Pseudomonas aeruginosa strain 197S020911BSL_PA2 287 WGS 197S020911BSL_PA2 RWUP01000000 29 6307339 Sputum Canada "Human, Homo sapiens" Resistant Computational Prediction "aph(3')-IIb,blaOXA-486,fosA,catB7,blaPAO" 99.4 Good 1 1 0 1 1 1 1 0 0 0 0 0

Pseudomonas aeruginosa strain 197S020911BSL_PA1 287 WGS 197S020911BSL_PA1 RWUQ01000000 28 6307533 Sputum Canada "Human, Homo sapiens" Resistant Computational Prediction "aph(3')-IIb,blaOXA-486,fosA,catB7,blaPAO" 99.4 Good 1 1 0 1 1 1 1 0 0 0 0 0

Pseudomonas aeruginosa strain 192S190811BSL_PA3 287 WGS 192S190811BSL_PA3 RWUR01000000 38 6302235 Sputum Canada "Human, Homo sapiens" Resistant Computational Prediction "aph(3')-IIb,blaOXA-50,fosA,catB7,blaPAO" 99 Good 1 1 0 1 1 1 1 0 0 0 0 0

Pseudomonas aeruginosa strain 192S190811BSL_PA2 287 WGS 192S190811BSL_PA2 RWUS01000000 35 6301321 Sputum Canada "Human, Homo sapiens" Resistant Computational Prediction "aph(3')-IIb,blaOXA-50,fosA,catB7,blaPAO" 99.2 Good 1 1 0 1 1 1 1 0 0 0 0 0

Pseudomonas aeruginosa strain 66S100212BSL _PA1 287 WGS 66S100212BSL _PA1 RWVD01000000 57 6650818 Sputum Canada "Human, Homo sapiens" Resistant Computational Prediction "aadA2b,aph(3'')-Ib,aph(6)-Id,ant(2'')-Ia,aph(3')-Iib,fosA,crpP,sul1,catB7,blaPAO,cmlA1,blaOXA-396,blaOXA-494,qacE" 98.5 Good 1 1 1 1 1 1 1 0 0 1 0 1

Pseudomonas aeruginosa strain 201s070911bsl_PA1 287 WGS 201s070911bsl_PA1 RWUM01000000 56 6811669 Sputum Canada "Human, Homo sapiens" Resistant Computational Prediction "crpP,aph(3')-IIb,blaOXA-50,fosA,catB7,blaPAO" 98.1 Good 1 1 0 1 1 1 1 0 0 0 0 1

Pseudomonas aeruginosa strain 192S190811BSL_PA1 287 WGS 192S190811BSL_PA1 RWUT01000000 32 6299581 Sputum Canada "Human, Homo sapiens" Resistant Computational Prediction "aph(3')-IIb,blaOXA-50,fosA,catB7,blaPAO" 99.2 Good 1 1 0 1 1 1 1 0 0 0 0 0

Pseudomonas aeruginosa AZPAE14984 287 WGS AZPAE14984 JTPK00000000 84 6809111 urinary tract infection France "Human, Homo sapiens" Resistant AMR Panel "crpP,aph(3')-IIb,blaOXA-396,fosA,catB7,blaPAO" 99 100 Good 1 1 1 1 1 1 1 0 0 0 0 1

Pseudomonas aeruginosa strain 4094345290 287 WGS 4094345290 RWVJ01000000 29 7077392 Sputum Canada "Human, Homo sapiens" Resistant Computational Prediction "crpP,aph(3')-IIb,blaOXA-396,fosA,catB7,blaPAO" 98.8 Good 1 1 1 1 1 1 1 0 0 0 0 1

Pseudomonas aeruginosa strain PAC31A 287 WGS PAC31A RWRS01000000 29 6486658 Sputum Canada "Human, Homo sapiens" Resistant Computational Prediction "aph(3')-IIb,blaOXA-396,fosA,catB7,blaPAO" 99.2 Good 1 1 1 1 1 1 1 0 0 0 0 0

Pseudomonas aeruginosa strain F815101 287 WGS F815101 SRHR01000000 304 7572594 fluid Canada "Human, Homo sapiens" Resistant Computational Prediction "fosA,aac(6')-33,aac(6')-Ib-cr,aac(6')-Ib3,aph(3')-Iib,crpP,sul1,blaGES-16,blaPAO,blaOXA-486,blaGES-5,qacE,catB7," 98.1 Good 1 1 0 1 1 1 1 0 0 0 0 1

Pseudomonas aeruginosa strain MMK2018 287 WGS MMK2018 SRKE01000000 108 7009277 wound Myanmar "Human, Homo sapiens" Resistant Computational Prediction "fosA,aadA1,aph(6)-Id,aac(6')-Ib-cr,aph(3'')-Ib,aph(3')-Iib,aac(6')-Ib-Hangzhou,ant(2'')-Ia,qnrVC1,crpP,dfrA14,dfrA10,tet(G),ARR-2,blaVEB-1,blaPAO,blaNDM-1,blaOXA-486,blaOXA-10,qacE,cmx,cmlA1" 98.4 Good 1 1 1 1 1 1 1 0 1 1 0 1

Pseudomonas aeruginosa strain CCBH3462 287 WGS CCBH3462 SRPM01000000 131 6656481 Brazil "Human, Homo sapiens" Resistant Computational Prediction "aadA7,aac(6')-Ib-cr,aph(3')-Iib,aac(6')-Ib3,fosA,crpP,sul1,blaPAO,blaoXA-56,blaOXA-494,blaSPM-1,blaOXA-396,cmx,catB7" 98.7 Good 1 1 1 1 1 1 1 0 0 0 0 1

Pseudomonas aeruginosa strain CCBH276 287 WGS CCBH276 SRPL01000000 112 6640220 Brazil "Human, Homo sapiens" Resistant Computational Prediction "fosA,aadA7,aph(3')-Iib,aac(6')-Ib3,aac(6')-Ib-cr,crpP,sul1,blaOXA-56,blaPAO,cmx,blaOXA-494,blaOXA-396,catB7,qacE" 98.7 Good 1 1 1 1 1 1 1 0 0 0 0 1

Pseudomonas aeruginosa strain NUBRI-P 287 WGS NUBRI-P SMZF01000000 99 6452072 Wound swab Sudan "Human, Homo sapiens" Resistant Computational Prediction "crpP,aph(3')-IIb,blaOXA-395,fosA,catB7,blaPAO" 99.2 Good 1 1 1 1 1 1 1 0 0 0 0 1

Pseudomonas aeruginosa strain 19-032124 287 WGS 19-032124 SSDC01000000 130 7089704 USA "Human, Homo sapiens" Resistant Computational Prediction "fosA,aph(3')-Iib,aac(6')-29a,crpP,sul1,blaPAO,blaOXA-395,blaVIM-2,qacE,catB7" 98.8 Good 1 1 1 1 1 1 1 0 0 0 0 1

Pseudomonas aeruginosa strain 19-026975 287 WGS 19-026975 SSDE01000000 194 7205149 USA "Human, Homo sapiens" Resistant Computational Prediction "fosA,aph(3')-Iib,crpP,sul1,blaPAO,blaOXA-395,blaVIM-2,qacE,catB7" 98.7 Good 1 1 1 1 1 1 1 0 0 0 0 1

Pseudomonas aeruginosa strain 19-028480 287 WGS 19-028480 SSDD01000000 124 7226690 USA "Human, Homo sapiens" Resistant Computational Prediction "fosA,aph(3')-Iib,crpP,sul1,blaPAO,blaOXA-395,blaVIM-2,qacE,catB7" 98.8 Good 1 1 1 1 1 1 1 0 0 0 0 1

Pseudomonas aeruginosa strain BWH047 287 WGS BWH047 SUPJ01000000 138 6742224 bronchoalveolar lavage USA "Human, Homo sapiens" Resistant Computational Prediction "fosA,ant(2'')-Ia,aph(3')-Iib,crpP,sul1,dfrA10,blaOXA-488,qacE,catB7" 98.7 Good 0 0 0 0 1 0 1 0 0 1 0 1

Pseudomonas aeruginosa strain PA298 287 Complete PA298 "CP040127,CP040126" 2 6935668 feces China "Human, Homo sapiens" Resistant Computational Prediction "fosA,aac(6')-Ib-cr,aph(3')-Iib,aac(6')-Ib3,crpP,sul1,blaOXA-1,blaOXA-494,blaOXA-396,blaPAO,blaVIM-1,blaVIM-45,qacE,catB3,catB7" 98.5 Good 1 1 1 1 1 1 1 0 0 0 0 1

Pseudomonas aeruginosa strain MED02 287 WGS MED02 VAUY01000000 250 7251609 stool France "Human, Homo sapiens" Resistant Computational Prediction "fosA,aac(6')-Ib-cr,aph(3')-Iib,aac(6')-Ib3,crpP,sul1,blaPAO,blaOXA-9,blaOXA-395,qacE,catB7" 97.6 Good 1 1 1 1 1 1 1 0 0 0 0 1

Pseudomonas aeruginosa strain MED03 287 WGS MED03 VAUZ01000000 186 7258187 wound France "Human, Homo sapiens" Resistant Computational Prediction "fosA,aac(6')-Ib-cr,aph(3')-Iib,aac(6')-Ib3,crpP,sul1,blaPAO,blaOXA-9,blaOXA-395,qacE,catB7" 98.4 Good 1 1 1 1 1 1 1 0 0 0 0 1

Pseudomonas aeruginosa strain MED01 287 WGS MED01 VAUX01000000 178 7252711 bile France "Human, Homo sapiens" Resistant Computational Prediction "fosA,aac(6')-Ib-cr,aph(3')-Iib,aac(6')-Ib3,crpP,sul1,blaPAO,blaOXA-9,blaOXA-395,qacE,catB7" 98.5 Good 1 1 1 1 1 1 1 0 0 0 0 1

Pseudomonas aeruginosa strain MED04 287 WGS MED04 VAVA01000000 188 7259254 wound France "Human, Homo sapiens" Resistant Computational Prediction "fosA,aac(6')-Ib-cr,aph(3')-Iib,aac(6')-Ib3,crpP,sul1,blaPAO,blaOXA-9,blaOXA-395,qacE,catB7" 98.2 Good 1 1 1 1 1 1 1 0 0 0 0 1

Pseudomonas aeruginosa strain MED06 287 WGS MED06 VAVC01000000 190 7263170 blood France "Human, Homo sapiens" Resistant Computational Prediction "fosA,aac(6')-Ib-cr,aph(3')-Iib,aac(6')-Ib3,crpP,sul1,blaPAO,blaOXA-9,blaOXA-395,qacE,catB7" 98.7 Good 1 1 1 1 1 1 1 0 0 0 0 1

Pseudomonas aeruginosa strain MED07 287 WGS MED07 VAVD01000000 112 7246584 blood France "Human, Homo sapiens" Resistant Computational Prediction "fosA,aph(3')-Iib,aac(6')-Ib3,crpP,sul1,blaPAO,blaOXA-9,blaOXA-395,qacE,catB7" 98.6 Good 1 1 1 1 1 1 1 0 0 0 0 1

Pseudomonas aeruginosa strain MED05 287 WGS MED05 VAVB01000000 209 7258432 bile France "Human, Homo sapiens" Resistant Computational Prediction "fosA,aac(6')-Ib-cr,aph(3')-Iib,aac(6')-Ib3,crpP,sul1,blaPAO,blaOXA-9,blaOXA-395,qacE,catB7" 98.2 Good 1 1 1 1 1 1 1 0 0 0 0 1

Pseudomonas aeruginosa strain MED08 287 WGS MED08 VAVE01000000 217 7258464 blood France "Human, Homo sapiens" Resistant Computational Prediction "fosA,aac(6')-Ib-cr,aph(3')-Iib,aac(6')-Ib3,crpP,sul1,blaPAO,blaOXA-9,blaOXA-395,qacE,catB7" 97.9 Good 1 1 1 1 1 1 1 0 0 0 0 1

Pseudomonas aeruginosa strain MED09 287 WGS MED09 VAVF01000000 204 7257001 blood France "Human, Homo sapiens" Resistant Computational Prediction "fosA,aac(6')-Ib-cr,aph(3')-Iib,aac(6')-Ib3,crpP,sul1,blaPAO,blaOXA-9,blaOXA-395,qacE,catB7" 98.4 Good 1 1 1 1 1 1 1 0 0 0 0 1

Pseudomonas aeruginosa strain MED10 287 WGS MED10 VAVG01000000 213 7256979 blood France "Human, Homo sapiens" Resistant Computational Prediction "fosA,aac(6')-Ib-cr,aph(3')-Iib,aac(6')-Ib3,crpP,sul1,blaPAO,blaOXA-9,blaOXA-395,qacE,catB7" 98.1 Good 1 1 1 1 1 1 1 0 0 0 0 1

Pseudomonas aeruginosa strain MED13 287 WGS MED13 VAVJ01000000 215 7259036 blood France "Human, Homo sapiens" Resistant Computational Prediction "fosA,aac(6')-Ib-cr,aph(3')-Iib,aac(6')-Ib3,crpP,sul1,blaPAO,blaOXA-9,blaOXA-395,qacE,catB7" 98.1 Good 1 1 1 1 1 1 1 0 0 0 0 1

Pseudomonas aeruginosa strain MED14 287 WGS MED14 VAVK01000000 225 7217167 blood France "Human, Homo sapiens" Resistant Computational Prediction "fosA,aac(6')-Ib-cr,aph(3')-Iib,aac(6')-Ib3,crpP,sul1,blaPAO,blaOXA-9,blaOXA-395,qacE,catB7" 98.2 Good 1 1 1 1 1 1 1 0 0 0 0 1

Pseudomonas aeruginosa strain MED12 287 WGS MED12 VAVI01000000 202 7256123 stool France "Human, Homo sapiens" Resistant Computational Prediction "fosA,aac(6')-Ib-cr,aph(3')-Iib,aac(6')-Ib3,crpP,sul1,blaPAO,blaOXA-9,blaOXA-395,qacE,catB7" 98.5 Good 1 1 1 1 1 1 1 0 0 0 0 1

Pseudomonas aeruginosa strain MED11 287 WGS MED11 VAVH01000000 105 7253899 blood France "Human, Homo sapiens" Resistant Computational Prediction "fosA,aph(3')-Iib,aac(6')-Ib3,crpP,sul1,blaPAO,blaOXA-9,blaOXA-395,qacE,catB7" 98.5 Good 1 1 1 1 1 1 1 0 0 0 0 1

Pseudomonas aeruginosa strain MED15 287 WGS MED15 VAVL01000000 210 7214008 Pulmonary France "Human, Homo sapiens" Resistant Computational Prediction "fosA,aac(6')-Ib-cr,aph(3')-Iib,aac(6')-Ib3,crpP,sul1,blaPAO,blaOXA-9,blaOXA-395,qacE,catB7" 98.7 Good 1 1 1 1 1 1 1 0 0 0 0 1

Pseudomonas aeruginosa strain MED16 287 WGS MED16 VAVM01000000 108 7254954 blood France "Human, Homo sapiens" Resistant Computational Prediction "fosA,aac(6')-Ib-cr,aph(3')-Iib,aac(6')-Ib3,crpP,sul1,blaPAO,blaOXA-9,blaOXA-395,qacE,catB7" 98.6 Good 1 1 1 1 1 1 1 0 0 0 0 1

Pseudomonas aeruginosa strain MED18 287 WGS MED18 VAVO01000000 109 7248172 blood France "Human, Homo sapiens" Resistant Computational Prediction "fosA,aac(6')-Ib-cr,aph(3')-Iib,aac(6')-Ib3,crpP,sul1,blaPAO,blaOXA-9,blaOXA-395,qacE,catB7" 98.6 Good 1 1 1 1 1 1 1 0 0 0 0 1

Pseudomonas aeruginosa strain MED17 287 WGS MED17 VAVN01000000 98 7256676 blood France "Human, Homo sapiens" Resistant Computational Prediction "fosA,aac(6')-Ib-cr,aph(3')-Iib,aac(6')-Ib3,crpP,sul1,blaPAO,blaOXA-9,blaOXA-395,qacE,catB7" 98.7 Good 1 1 1 1 1 1 1 0 0 0 0 1

Pseudomonas aeruginosa strain MED19 287 WGS MED19 VAVP01000000 260 7274952 blood France "Human, Homo sapiens" Resistant Computational Prediction "fosA,aac(6')-Ib-cr,aph(3')-Iib,aac(6')-Ib3,crpP,sul1,blaPAO,blaOXA-9,blaOXA-395,qacE,catB7" 98.3 Good 1 1 1 1 1 1 1 0 0 0 0 1

Pseudomonas aeruginosa strain MED21 287 WGS MED21 VAVR01000000 106 7253747 blood France "Human, Homo sapiens" Resistant Computational Prediction "fosA,aph(3')-Iib,aac(6')-Ib3,crpP,sul1,blaPAO,blaOXA-9,blaOXA-395,qacE,catB7" 98.5 Good 1 1 1 1 1 1 1 0 0 0 0 1

Pseudomonas aeruginosa strain MED20 287 WGS MED20 VAVQ01000000 222 7263437 blood France "Human, Homo sapiens" Resistant Computational Prediction "fosA,aac(6')-Ib-cr,aph(3')-Iib,aac(6')-Ib3,crpP,sul1,blaPAO,blaOXA-9,blaOXA-395,qacE,catB7" 98.3 Good 1 1 1 1 1 1 1 0 0 0 0 1

Pseudomonas aeruginosa strain MED22 287 WGS MED22 VAVS01000000 240 7262266 blood France "Human, Homo sapiens" Resistant Computational Prediction "fosA,aac(6')-Ib-cr,aph(3')-Iib,aac(6')-Ib3,crpP,sul1,blaPAO,blaOXA-9,blaOXA-395,qacE,catB7" 98.3 Good 1 1 1 1 1 1 1 0 0 0 0 1

Pseudomonas aeruginosa strain MED24 287 WGS MED24 VAVU01000000 238 7268478 stool France "Human, Homo sapiens" Resistant Computational Prediction "fosA,aac(6')-Ib-cr,aph(3')-Iib,aac(6')-Ib3,crpP,sul1,blaPAO,blaOXA-9,blaOXA-395,qacE,catB7" 98.3 Good 1 1 1 1 1 1 1 0 0 0 0 1

Pseudomonas aeruginosa strain MED23 287 WGS MED23 VAVT01000000 220 7256118 blood France "Human, Homo sapiens" Resistant Computational Prediction "fosA,aac(6')-Ib-cr,aph(3')-Iib,aac(6')-Ib3,crpP,sul1,blaPAO,blaOXA-9,blaOXA-395,qacE,catB7" 98.5 Good 1 1 1 1 1 1 1 0 0 0 0 1

Pseudomonas aeruginosa strain MED25 287 WGS MED25 VAVV01000000 268 7263297 blood France "Human, Homo sapiens" Resistant Computational Prediction "fosA,aac(6')-Ib-cr,aph(3')-Iib,aac(6')-Ib3,crpP,sul1,blaPAO,blaOXA-9,blaOXA-395,qacE,catB7" 98 Good 1 1 1 1 1 1 1 0 0 0 0 1

Pseudomonas aeruginosa strain MED26 287 WGS MED26 VAVW01000000 229 7263039 stool France "Human, Homo sapiens" Resistant Computational Prediction "fosA,aac(6')-Ib-cr,aph(3')-Iib,aac(6')-Ib3,crpP,sul1,blaPAO,blaOXA-9,blaOXA-395,qacE,catB7" 98.4 Good 1 1 1 1 1 1 1 0 0 0 0 1

Pseudomonas aeruginosa strain MED27 287 WGS MED27 VAVX01000000 250 7262164 blood France "Human, Homo sapiens" Resistant Computational Prediction "fosA,aac(6')-Ib-cr,aph(3')-Iib,aac(6')-Ib3,crpP,sul1,blaPAO,blaOXA-9,blaOXA-395,qacE,catB7" 98.4 Good 1 1 1 1 1 1 1 0 0 0 0 1

Pseudomonas aeruginosa strain NCTC13717 287 WGS NCTC13717 UAUC01000000 26 7020415 blood United Kingdom "Human, Homo sapiens" Resistant Computational Prediction "aadA2,fosA,aac(6')-Il,aac(3)-Id,aph(3')-Iib,crpP,sul1,dfrB5,tet(G),blaPAO,cmlA1,catB7,blaOXA-4,blaOXA-486,blaVIM-2,qacE" 98.1 Good 1 1 1 1 1 1 1 0 1 1 0 1

Pseudomonas aeruginosa strain NCTC10727 287 WGS NCTC10727 UAUH01000000 62 6823435 not available: to be reported later Resistant Computational Prediction "crpP,aph(3')-IIb,blaOXA-488,fosA,catB7,blaPAO" 98.4 Good 1 1 0 1 1 1 1 0 0 1 0 1

Pseudomonas aeruginosa strain NCTC13716 287 WGS NCTC13716 UAUB01000000 56 7043294 sputum United Kingdom "Human, Homo sapiens" Resistant Computational Prediction "fosA,aac(6')-29a,aac(6')-29b,aph(3')-Iib,crpP,sul1,blaPAO,blaOXA-395,blaVIM-2,qacE,catB7" 98.2 Good 1 1 1 1 1 1 1 0 0 0 0 1

Pseudomonas aeruginosa strain NCTC13921 287 WGS NCTC13921 UAUD01000000 24 6902612 throat United Kingdom "Human, Homo sapiens" Resistant Computational Prediction "fosA,aadA7,rmtD,aac(6')-Ib-cr,aac(6')-Ib3,aph(3')-Iib,sul1,crpP,blaPAO,blaOXA-494,blaOXA-396,blaOXA-56,qacE,cmx,catB7" 97.9 Good 1 1 1 1 1 1 1 0 0 1 0 1

Pseudomonas aeruginosa strain 1 287 Complete 1 "LS998783,LS998784" 2 7231910 Resistant Computational Prediction "fosA,aadA1b,aac(6')-Ib-cr,aph(3')-Iib,aac(6')-Ib3,sul1,crpP,blaPAO,blaGIM-1,blaOXA-395,blaOXA-2,qacE,catB7" 98.8 Good 1 1 1 1 1 1 1 0 0 0 0 1

Pseudomonas aeruginosa strain XDR-PA 287 WGS XDR-PA "CAADJN010000001,CAADJN010000002,CAADJN010000003,CAADJN010000004,CAADJN010000005,CAADJN010000006,CAADJN010000007" 853 6693171 Spain "Human, Homo sapiens" Resistant Computational Prediction "crpP,aph(3')-IIb,blaOXA-494,blaOXA-396,fosA,catB7,blaPAO" 92.6 Good 1 1 1 1 1 1 1 0 0 0 0 1

Pseudomonas aeruginosa strain NCTC13437 287 WGS NCTC13437 "CAADJS010000001,CAADJS010000002,CAADJS010000003" 3 7156099 not available: not collected not available: not collected Resistant Computational Prediction "fosA,aadA1,aph(3')-Iib,ant(2'')-Ia,aac(6')-Il,crpP,sul1,dfrB2,tet(A),blaOXA-50,blaOXA-10,blaVIM-2,blaVEB-1,blapAO,catB7" 94.5 Good 1 1 1 1 1 1 1 0 1 1 0 1

Pseudomonas aeruginosa strain XDR-PA 287 WGS XDR-PA "CAADLR010000001,CAADLR010000002,CAADLR010000003,CAADLR010000004,CAADLR010000005,CAADLR010000006,CAADLR010000007" 803 6176443 Spain "Human, Homo sapiens" Resistant Computational Prediction "aph(3')-IIb,blaOXA-50,fosA,catB7,blaPAO" 93.6 Good 1 1 0 1 1 1 1 0 0 0 0 0

Pseudomonas aeruginosa strain XDR-PA 287 WGS XDR-PA "CAADLS010000001,CAADLS010000002,CAADLS010000003,CAADLS010000004,CAADLS010000005,CAADLS010000006,CAADLS010000007" 961 6831938 Spain "Human, Homo sapiens" Resistant Computational Prediction "fosA,aph(6)-Id,aph(3'')-Ib,aac(6')-Ib-cr,aph(3')-Iib,aac(6')-Ib3,crpP,sul1,blaOXA-488,blaPAO,qacE,catB7" 93 Good 1 1 0 1 1 1 1 0 0 1 0 1

Pseudomonas aeruginosa strain XDR-PA 287 WGS XDR-PA "CAADLT010000001,CAADLT010000002,CAADLT010000003,CAADLT010000004,CAADLT010000005,CAADLT010000006,CAADLT010000007" 546 6354481 Spain "Human, Homo sapiens" Resistant Computational Prediction "crpP,aph(3')-IIb,blaOXA-494,blaOXA-396,fosA,catB7,blaPAO" 96.4 Good 1 1 1 1 1 1 1 0 0 0 0 1

Pseudomonas aeruginosa strain XDR-PA 287 WGS XDR-PA "CAADMA010000001,CAADMA010000002,CAADMA010000003,CAADMA010000004,CAADMA010000005,CAADMA010000006,CAADMA010000007" 972 6998541 Spain "Human, Homo sapiens" Resistant Computational Prediction "fosA,aph(3'')-Ib,aac(6')-Il,aph(3')-Iib,aph(3')-Vib,aph(6)-Id,tet(Y),blapAO,blaPER-1,blaOXA-2,blaOXA-488,qacE,cmlA1,catB7" 93.3 Good 1 1 0 1 1 1 1 0 1 1 1 0

Pseudomonas aeruginosa strain XDR-PA 287 WGS XDR-PA "CAADME010000001,CAADME010000002,CAADME010000003,CAADME010000004,CAADME010000005,CAADME010000006,CAADME010000007" 902 6861986 Spain "Human, Homo sapiens" Resistant Computational Prediction "qacE,aph(3')-Iib,ant(2'')-Ia,blaOXA-50,catB7,blaPAO,fosA" 94.6 Good 1 1 0 1 1 1 1 0 0 1 0 0

Pseudomonas aeruginosa strain XDR-PA 287 WGS XDR-PA "CAADMK010000001,CAADMK010000002,CAADMK010000003,CAADMK010000004,CAADMK010000005,CAADMK010000006,CAADMK010000007" 965 6912434 Spain "Human, Homo sapiens" Resistant Computational Prediction "fosA,aac(6')-Ib-cr,aac(6')-Ib3,aph(3')-Iib,crpP,blaPAO,blaOXA-50,qacE,catB7," 93.5 Good 1 1 0 1 1 1 1 0 0 0 0 1

Pseudomonas aeruginosa strain XDR-PA 287 WGS XDR-PA "CAADMM010000001,CAADMM010000002,CAADMM010000003,CAADMM010000004,CAADMM010000005,CAADMM010000006,CAADMM010000007" 880 6891404 Spain "Human, Homo sapiens" Resistant Computational Prediction "aadA2b,aac(6')-Ib-cr,aac(3)-Ia,aph(3')-Iib,aac(6')-Ib3,crpP,blaPAO,blaOXA-395,blaCARB-2,fosA" 94.2 Good 1 1 1 1 1 1 0 0 0 1 0 1

Pseudomonas aeruginosa strain XDR-PA 287 WGS XDR-PA "CAADMN010000001,CAADMN010000002,CAADMN010000003,CAADMN010000004,CAADMN010000005,CAADMN010000006,CAADMN010000007" 931 6799465 Spain "Human, Homo sapiens" Resistant Computational Prediction "fosA,qacE,blaPAO,blaOXA-50,aph(3')-Iib,ant(2'')-Ia,aac(3)-Ia,crpP" 93.3 Good 1 1 0 1 1 1 0 0 0 1 0 1

Pseudomonas aeruginosa strain XDR-PA 287 WGS XDR-PA "CAADMQ010000001,CAADMQ010000002,CAADMQ010000003,CAADMQ010000004,CAADMQ010000005,CAADMQ010000006,CAADMQ010000007" 938 6785862 Spain "Human, Homo sapiens" Resistant Computational Prediction "fosA,qacE,blaPAO,blaOXA-50,aph(3')-Iib,ant(2'')-Ia,catB7,crpP" 93.7 Good 1 1 0 1 1 1 1 0 0 1 0 1

Pseudomonas aeruginosa strain XDR-PA 287 WGS XDR-PA "CAADMT010000001,CAADMT010000002,CAADMT010000003,CAADMT010000004,CAADMT010000005,CAADMT010000006,CAADMT010000007" 861 6734139 Spain "Human, Homo sapiens" Resistant Computational Prediction "fosA,qacE,blaPAO,blaOXA-50,aph(3')-Iib,ant(2'')-Ia,catB7,crpP" 94.4 Good 1 1 0 1 1 1 1 0 0 1 0 1

Pseudomonas aeruginosa strain XDR-PA 287 WGS XDR-PA "CAADMZ010000001,CAADMZ010000002,CAADMZ010000003,CAADMZ010000004,CAADMZ010000005,CAADMZ010000006,CAADMZ010000007" 1000 6861121 Spain "Human, Homo sapiens" Resistant Computational Prediction "fosA,qacE,blaOXA-50,aph(3')-Iib,ant(2'')-Ia,catB7,crpP" 93.3 Good 1 1 0 1 1 1 1 0 0 1 0 1

Pseudomonas aeruginosa strain XDR-PA 287 WGS XDR-PA "CAADNB010000001,CAADNB010000002,CAADNB010000003,CAADNB010000004,CAADNB010000005,CAADNB010000006,CAADNB010000007" 704 6681658 Spain "Human, Homo sapiens" Resistant Computational Prediction "fosA,aadA6,aph(6)-Id,ant(2'')-Ia,aph(3')-Iib,aac(6')-Il,sul1,tet©,blaPAO,blaOXA-2,blaOXA-488,qacE,catB7" 94.4 Good 1 1 0 1 1 1 1 0 1 1 0 0

Pseudomonas aeruginosa AZPAE14983 287 WGS AZPAE14983 JTPL00000000 96 7082650 respiratory tract infection Croatia "Human, Homo sapiens" Resistant AMR Panel "fosA,aadA2b,aac(6')-Ib-cr,aph(3')-Iib,aac(6')-Ib3,crpP,sul1,blaPAO,blaCARB-2,blaOXA-395,qacE,catB7" 99 100 Good 1 1 1 1 1 1 1 0 0 0 0 1

Pseudomonas aeruginosa strain XDR-PA 287 WGS XDR-PA "CAADNS010000001,CAADNS010000002,CAADNS010000003,CAADNS010000004,CAADNS010000005,CAADNS010000006,CAADNS010000007" 929 6651466 Spain "Human, Homo sapiens" Resistant Computational Prediction "crpP,aph(3')-IIb,blaOXA-488,fosA,catB7,blaPAO" 93.9 Good 1 1 0 1 1 1 1 0 0 0 0 1

Pseudomonas aeruginosa strain XDR-PA 287 WGS XDR-PA "CAADNG010000001,CAADNG010000002,CAADNG010000003,CAADNG010000004,CAADNG010000005,CAADNG010000006,CAADNG010000007" 871 6766723 Spain "Human, Homo sapiens" Resistant Computational Prediction "ant(2'')-Ia,aph(3')-IIb,blaOXA-50,fosA,catB7,blaPAO,qacE" 94.5 Good 1 1 0 1 1 1 1 0 0 1 0 0

Pseudomonas aeruginosa strain XDR-PA 287 WGS XDR-PA "CAADNP010000001,CAADNP010000002,CAADNP010000003,CAADNP010000004,CAADNP010000005,CAADNP010000006,CAADNP010000007" 779 6908630 Spain "Human, Homo sapiens" Resistant Computational Prediction "crpP,ant(2'')-Ia,aph(3')-IIb,blaOXA-50,fosA,catB7,blaPAO,qacE" 95.3 Good 1 1 0 1 1 1 1 0 0 1 0 1

Pseudomonas aeruginosa strain XDR-PA 287 WGS XDR-PA "CAADNX010000001,CAADNX010000002,CAADNX010000003,CAADNX010000004,CAADNX010000005,CAADNX010000006,CAADNX010000007" 866 6637487 Spain "Human, Homo sapiens" Resistant Computational Prediction "fosA,aadA6,aph(3')-Iib,sul1,crpP,blaPAO,blaOXA-488,qacE,catB7" 94 Good 1 1 0 1 1 1 1 0 0 1 0 1

Pseudomonas aeruginosa strain XDR-PA 287 WGS XDR-PA "CAADOA010000001,CAADOA010000002,CAADOA010000003,CAADOA010000004,CAADOA010000005,CAADOA010000006,CAADOA010000007" 826 6487244 Spain "Human, Homo sapiens" Resistant Computational Prediction "fosA,aac(3)-Ic,aph(3')-Iib,sul1,blaOXA-488,blaPAO,qacE,catB7,cmlA1" 93.4 Good 1 1 0 1 1 1 1 0 0 1 0 0

Pseudomonas aeruginosa strain XDR-PA 287 WGS XDR-PA "CAADNZ010000001,CAADNZ010000002,CAADNZ010000003,CAADNZ010000004,CAADNZ010000005,CAADNZ010000006,CAADNZ010000007" 831 6598134 Spain "Human, Homo sapiens" Resistant Computational Prediction "fosA,aac(6')-Ib-cr,aph(3'')-Ib,aac(6')-Ib-Hangzhou,aph(6)-Id,aph(3')-Iib,sul1,blaPAO,blaOXA-488,qacE,catB7" 94.7 Good 1 1 0 1 1 1 1 0 0 1 0 1

Pseudomonas aeruginosa strain XDR-PA 287 WGS XDR-PA "CAADOB010000001,CAADOB010000002,CAADOB010000003,CAADOB010000004,CAADOB010000005,CAADOB010000006,CAADOB010000007" 657 6327434 Spain "Human, Homo sapiens" Resistant Computational Prediction "fosA,aadA13,aph(3')-VI,ant(2'')-Ia,aph(3')-Iib,crpP,blaPAO,blaVIM-20,blaOXA-210,blaOXA-50,qacE,catB7" 94.3 Good 1 1 1 1 1 1 1 0 0 1 0 1

Pseudomonas aeruginosa strain XDR-PA 287 WGS XDR-PA "CAADOE010000001,CAADOE010000002,CAADOE010000003,CAADOE010000004,CAADOE010000005,CAADOE010000006,CAADOE010000007" 917 6725522 Spain "Human, Homo sapiens" Resistant Computational Prediction "fosA,ant(2'')-Ia,aph(3')-VI,aac(6')-Ib-cr,aac(6')-Ib3,aph(3')-Iib,crpP,blaPAO,blaOXA-50,blaOXA-2,blaVIM-20,qacE,catB7" 93.3 Good 1 1 1 1 1 1 1 0 0 1 0 1

Pseudomonas aeruginosa AZPAE14980 287 WGS AZPAE14980 JTPO00000000 83 6768315 itra-abdominal tract infection United States "Human, Homo sapiens" Resistant;Susceptible AMR Panel "fosA,aac(6')-Ib3,aph(3')-VI,aac(6')-Ib-cr,aadA13,aph(3')-Iib,ant(2'')-Ia,crpP,blaPAO,blaOXA-2,blaOXA-50,blaVIM-20,qacE,catB7" 98.8 100 0.6 Good 1 1 1 1 1 1 1 0 0 1 0 1

Pseudomonas aeruginosa strain XDR-PA 287 WGS XDR-PA "CAADOC010000001,CAADOC010000002,CAADOC010000003,CAADOC010000004,CAADOC010000005,CAADOC010000006,CAADOC010000007" 981 6722030 Spain "Human, Homo sapiens" Resistant Computational Prediction "crpP,aph(3')-IIb,blaOXA-494,blaOXA-396,aac(3)-IIIa,fosA,catB7,blaPAO" 93.1 Good 1 1 1 1 1 1 1 0 0 1 0 1

Pseudomonas aeruginosa strain XDR-PA 287 WGS XDR-PA "CAADOD010000001,CAADOD010000002,CAADOD010000003,CAADOD010000004,CAADOD010000005,CAADOD010000006,CAADOD010000007" 869 6554316 Spain "Human, Homo sapiens" Resistant Computational Prediction "crpP,aph(3')-IIb,blaOXA-494,blaoXA-396,fosA,catB7,blaPAO" 93.5 Good 1 1 1 1 1 1 1 0 0 0 0 1

Pseudomonas aeruginosa AZPAE14979 287 WGS AZPAE14979 JTPP00000000 125 6751299 urinary tract infection United States "Human, Homo sapiens" Resistant AMR Panel "aadA6,aph(3')-Iib,fosA,crpP,tet(G),sul1,blaPAO,blaOXA-396,qacE,catB7" 98.1 100 0.3 Good 1 1 0 1 1 1 1 0 1 1 0 1

Pseudomonas aeruginosa strain XDR-PA 287 WGS XDR-PA "CAADOR010000001,CAADOR010000002,CAADOR010000003,CAADOR010000004,CAADOR010000005,CAADOR010000006,CAADOR010000007" 632 6554839 Spain "Human, Homo sapiens" Resistant Computational Prediction "fosA,aadA6,aph(3')-Iib,sul1,crpP,blaPAO,blaOXA-488,qacE,catB7" 96.2 Good 1 1 0 1 1 1 1 0 0 1 0 0

Pseudomonas aeruginosa strain XDR-PA 287 WGS XDR-PA "CAADOS010000001,CAADOS010000002,CAADOS010000003,CAADOS010000004,CAADOS010000005,CAADOS010000006,CAADOS010000007" 814 6888336 Spain "Human, Homo sapiens" Resistant Computational Prediction "crpP,aph(3')-IIb,blaOXA-494,blaoXA-396,fosA,catB7,blaPAO" 95 Good 1 1 1 1 1 1 1 0 0 0 0 1

Pseudomonas aeruginosa strain XDR-PA 287 WGS XDR-PA "CAADOV010000001,CAADOV010000002,CAADOV010000003,CAADOV010000004,CAADOV010000005,CAADOV010000006,CAADOV010000007" 591 6261712 Spain "Human, Homo sapiens" Resistant Computational Prediction "aph(3')-IIb,blaOXA-494,blaOXA-50,blaoXA-396,fosA,catB7,blaPAO" 95.9 Good 1 1 1 1 1 1 1 0 0 0 0 0

Pseudomonas aeruginosa AZPAE14978 287 WGS AZPAE14978 JTPQ00000000 132 7130480 urinary tract infection United States "Human, Homo sapiens" Resistant AMR Panel "fosA,catB7,blaOXA-486,aph(3')-Iib" 98.3 99.7 0.3 Good 0 0 0 0 1 0 1 0 0 0 0 0

Pseudomonas aeruginosa strain XDR-PA 287 WGS XDR-PA "CAADPA010000001,CAADPA010000002,CAADPA010000003,CAADPA010000004,CAADPA010000005,CAADPA010000006,CAADPA010000007" 938 6420472 Spain "Human, Homo sapiens" Resistant Computational Prediction "aac(6')-Ib3,aac(6')-Ib-cr,blaCARB-3,cmlA1,catB7,crpP,fosA,sul1,aph(3')-Iib,catB7,blaOXA-488,blaPAO,qacE" 92.8 Good 1 1 0 1 1 1 1 0 0 0 0 1

Pseudomonas aeruginosa strain XDR-PA 287 WGS XDR-PA "CAADPB010000001,CAADPB010000002,CAADPB010000003,CAADPB010000004,CAADPB010000005,CAADPB010000006,CAADPB010000007" 969 6455119 Spain "Human, Homo sapiens" Resistant Computational Prediction "aac(6')-Ib3,aac(6')-Ib-cr,cmlA1,catB7,crpP,fosA,sul1,aph(3')-Iib,catB7,blaOXA-488,blaPAO,qacE" 91.5 Good 1 1 0 1 1 1 1 0 0 0 0 1

Pseudomonas aeruginosa strain XDR-PA 287 WGS XDR-PA "CAADPE010000001,CAADPE010000002,CAADPE010000003,CAADPE010000004,CAADPE010000005,CAADPE010000006,CAADPE010000007" 758 6719663 Spain "Human, Homo sapiens" Resistant Computational Prediction "fosA,aph(3')-Iib,ant(2'')-Ia,crpP,blaPAO,catB7,blaOXA-50,qacE," 95.3 Good 1 1 0 1 1 1 1 0 0 1 0 1

Pseudomonas aeruginosa strain XDR-PA 287 WGS XDR-PA "CAADPG010000001,CAADPG010000002,CAADPG010000003,CAADPG010000004,CAADPG010000005,CAADPG010000006,CAADPG010000007" 947 6717811 Spain "Human, Homo sapiens" Resistant Computational Prediction "fosA,aph(6)-Id,aph(3')-Iib,aph(3'')-Ib,aac(6')-Ib3,crpP,sul1,blaPAO,tet©,blaOXA-488,catA2,catB7," 93.6 Good 1 1 0 1 1 1 1 0 1 1 0 1

Pseudomonas aeruginosa strain XDR-PA 287 WGS XDR-PA "CAADPJ010000001,CAADPJ010000002,CAADPJ010000003,CAADPJ010000004,CAADPJ010000005,CAADPJ010000006,CAADPJ010000007" 911 6877727 Spain "Human, Homo sapiens" Resistant Computational Prediction "fosA,aph(3')-Iib,ant(2'')-Ia,crpP,blaPAO,catB7,blaOXA-50,qacE," 94 Good 1 1 0 1 1 1 1 0 0 1 0 1

Pseudomonas aeruginosa strain XDR-PA 287 WGS XDR-PA "CAADPL010000001,CAADPL010000002,CAADPL010000003,CAADPL010000004,CAADPL010000005,CAADPL010000006,CAADPL010000007" 899 6887254 Spain "Human, Homo sapiens" Resistant Computational Prediction "fosA,aac(6')-Ib-cr,catB7,blaGES-5,blaPAO,blaOXA-50,ant(2'')-Ia,aph(3')-Iib,aac(6')-Ib3" 94.8 Good 1 1 0 1 1 1 1 0 0 1 0 1

Pseudomonas aeruginosa AZPAE14976 287 WGS AZPAE14976 JTPS00000000 104 6631621 respiratory tract infection China "Human, Homo sapiens" Resistant;Susceptible AMR Panel "fosA,aph(3')-Iia,aph(3')-Iib,aadA1b,aph(6)-Ic,aac(6')-33,aac(6')-Ib3,aac(6')-Ib-cr,sul1,blaPAO,blaGES-20,blaOXA-2,blaOXA-488qacE,catB7" 98.8 99.7 Good 1 1 0 1 1 1 1 0 0 1 0 1

Pseudomonas aeruginosa strain XDR-PA 287 WGS XDR-PA "CAADPT010000001,CAADPT010000002,CAADPT010000003,CAADPT010000004,CAADPT010000005,CAADPT010000006,CAADPT010000007" 972 6543170 Spain "Human, Homo sapiens" Resistant Computational Prediction "fosA,aac(6')-Iia,aph(3')-Iib,ant(2'')-Ia,crpP,sul1,blaPAO,blaCARB-2,blaOXA-486,qacE,catB7" 92.2 Good 1 1 0 1 1 1 1 0 0 1 0 0

Pseudomonas aeruginosa strain XDR-PA 287 WGS XDR-PA "CAADQF010000001,CAADQF010000002,CAADQF010000003,CAADQF010000004,CAADQF010000005,CAADQF010000006,CAADQF010000007" 799 6784540 Spain "Human, Homo sapiens" Resistant Computational Prediction "fosA,aph(3')-Iib,sul1,aac(6')-Il,crpP,aac(3)-I,blaPAO,blaIMP-8,blaOXA-396,sul1,qacE,catB7" 94.5 Good 1 1 1 1 1 1 1 0 0 1 0 1

Pseudomonas aeruginosa strain XDR-PA 287 WGS XDR-PA "CAADQS010000001,CAADQS010000002,CAADQS010000003,CAADQS010000004,CAADQS010000005,CAADQS010000006,CAADQS010000007" 718 6753924 Spain "Human, Homo sapiens" Resistant Computational Prediction "fosA,aph(3')-Iib,ant(2'')-Ia,crpP,blaPAO,catB7,blaOXA-50,qacE," 95.1 Good 1 1 0 1 1 1 1 0 0 1 0 1

Pseudomonas aeruginosa strain XDR-PA 287 WGS XDR-PA "CAADQW010000001,CAADQW010000002,CAADQW010000003,CAADQW010000004,CAADQW010000005,CAADQW010000006,CAADQW010000007" 909 6730132 Spain "Human, Homo sapiens" Resistant Computational Prediction "fosA,aac(3)-I,aac(6')-31,aph(3')-Iib,crpP,sul1,blaPAO,blaOXA-488,blaOXA-2,qacE,catB7" 93.6 Good 1 1 0 1 1 1 1 0 0 1 0 1

Pseudomonas aeruginosa AZPAE14969 287 WGS AZPAE14969 JTPZ00000000 179 6832241 itra-abdominal tract infection United States "Human, Homo sapiens" Resistant AMR Panel "crpP,fosA,catB7,blaOXA-488,aph(3')-Iib" 97.6 98.5 Good 1 1 0 1 1 1 1 0 0 1 0 1

Pseudomonas aeruginosa AZPAE14963 287 WGS AZPAE14963 JTQE00000000 50 6380431 respiratory tract infection Spain "Human, Homo sapiens" Resistant;Susceptible AMR Panel "crpP,fosA,catB7,blaOXA-396,blaOXA-494,aph(3')-Iib" 98.9 100 Good 1 1 1 1 1 1 1 0 0 0 0 1

Pseudomonas aeruginosa AZPAE14961 287 WGS AZPAE14961 JTQG00000000 81 6441305 respiratory tract infection Spain "Human, Homo sapiens" Resistant AMR Panel "crpP,fosA,catB7,blaOXA-486,aph(3')-Iib" 99 99.7 Good 1 1 0 1 1 1 1 0 0 0 0 1

Pseudomonas aeruginosa AZPAE14959 287 WGS AZPAE14959 JTQI00000000 90 6743682 itra-abdominal tract infection India "Human, Homo sapiens" Resistant;Susceptible AMR Panel "aadA1,aph(3')-Iib,aac(6')-Ib-cr,aac(6')-Ib3,fosA,dfrB2,sul1,ARR-2,blaPAO,blaVIM-2,blaOXA-10,blaOXA-395,cmlA1,catB7" 99 100 0.3 Good 1 1 1 1 1 1 1 0 0 1 0 1

Pseudomonas aeruginosa AZPAE14958 287 WGS AZPAE14958 JTQJ00000000 132 7130390 itra-abdominal tract infection India "Human, Homo sapiens" Resistant;Susceptible AMR Panel "crpP,fosA,catB7,blaOXA-396,blaOXA-494,aph(3')-Iib" 98.3 99.7 0.3 Good 1 1 1 1 1 1 1 0 0 0 0 1

Pseudomonas aeruginosa AZPAE14956 287 WGS AZPAE14956 JTQL00000000 455 7110106 itra-abdominal tract infection Germany "Human, Homo sapiens" Resistant;Intermediate;Susceptible AMR Panel "aadA1,aph(3')-Iib,aph(3'')-Ib,ant(2'')-Ia,aph(6)-Id,ant(4')-Iib,ARR-2,crpP,tet(G),tet(A),blaVEB-1,blaOXA-10,blaOXA-486,qacE" 96.1 96.7 0.3 Good 1 1 0 1 1 1 1 0 1 1 0 1

Pseudomonas aeruginosa strain PA-NM-088 287 WGS PA-NM-088 VIGZ01000000 222 7201493 blood USA "Human, Homo sapiens" Resistant Computational Prediction "aadA10,ant(2'')-Ia,aph(3')-Iib,fosA,crpP,sul1,blaPAO,blaOXA-395,blaOXA-10,qacE,catB7" Good 1 1 1 1 1 1 1 0 0 1 0 1

Pseudomonas aeruginosa strain PA-NM-079 287 WGS PA-NM-079 VIHA01000000 217 7199132 BAL USA "Human, Homo sapiens" Resistant Computational Prediction "aadA10,ant(2'')-Ia,aph(3')-Iib,fosA,crpP,sul1,blaPAO,blaOXA-395,blaOXA-10,qacE,catB7" 97.7 Good 1 1 1 1 1 1 1 0 0 1 0 1

Pseudomonas aeruginosa strain PA-NM-069 287 WGS PA-NM-069 VIHC01000000 229 7204989 BAL USA "Human, Homo sapiens" Resistant Computational Prediction "aadA10,ant(2'')-Ia,aph(3')-Iib,fosA,crpP,sul1,blaPAO,blaOXA-395,blaOXA-10,qacE,catB7" 97.7 Good 1 1 1 1 1 1 1 0 0 1 0 1

Pseudomonas aeruginosa strain PS1948 287 WGS PS1948 VIHH01000000 162 6881180 urine USA "Human, Homo sapiens" Resistant Computational Prediction "crpP,fosA,catB7,blaOXA-395,aph(3')-Iib" 98.4 Good 1 1 1 1 1 1 1 0 0 1 0 1

Pseudomonas aeruginosa strain PS2027 287 WGS PS2027 VIHE01000000 214 6813452 blood USA "Human, Homo sapiens" Resistant Computational Prediction "crpP,fosA,catB7,blaOXA-395,aph(3')-Iib" 98.4 Good 1 1 1 1 1 1 1 0 0 1 0 1

Pseudomonas aeruginosa strain PS1955 287 WGS PS1955 VIHG01000000 229 6820851 blood USA "Human, Homo sapiens" Resistant Computational Prediction "crpP,fosA,catB7,blaOXA-395,aph(3')-Iib" 98.4 Good 1 1 1 1 1 1 1 0 0 1 0 1

Pseudomonas aeruginosa strain PS1946 287 WGS PS1946 VIHI01000000 163 6881492 blood USA "Human, Homo sapiens" Resistant Computational Prediction "crpP,fosA,catB7,blaOXA-395,aph(3')-Iib" 98.5 Good 1 1 1 1 1 1 1 0 0 1 0 1

Pseudomonas aeruginosa strain PS1900 287 WGS PS1900 VIHK01000000 219 7227545 fluid USA "Human, Homo sapiens" Resistant Computational Prediction "aadA10,ant(2'')-Ia,aph(3')-Iib,fosA,crpP,sul1,blaPAO,blaOXA-395,blaOXA-10,qacE,catB7" 97.8 Good 1 1 1 1 1 1 1 0 0 1 0 1

Pseudomonas aeruginosa strain PS1893 287 WGS PS1893 VIHL01000000 241 7221184 fluid urethra USA "Human, Homo sapiens" Resistant Computational Prediction "aadA10,ant(2'')-Ia,aph(3')-Iib,fosA,crpP,sul1,blaPAO,blaOXA-395,blaOXA-10,qacE,catB7" 97.8 Good 1 1 1 1 1 1 1 0 0 1 0 1

Pseudomonas aeruginosa AZPAE14954 287 WGS AZPAE14954 JTQN00000000 71 6368200 itra-abdominal tract infection France "Human, Homo sapiens" Resistant;Susceptible AMR Panel "crpP,fosA,catB7,blaOXA-395,aph(3')-Iib" 99 100 Good 1 1 1 1 1 1 1 0 0 1 0 1

Pseudomonas aeruginosa strain PS1934 287 WGS PS1934 VIHJ01000000 236 7215523 urine USA "Human, Homo sapiens" Resistant Computational Prediction "aadA10,ant(2'')-Ia,aph(3')-Iib,fosA,crpP,sul1,blaPAO,blaOXA-395,blaOXA-10,qacE,catB7" 97.7 Good 1 1 1 1 1 1 1 0 0 1 0 1

Pseudomonas aeruginosa strain PS1882 287 WGS PS1882 VIHN01000000 209 7225094 blood USA "Human, Homo sapiens" Resistant Computational Prediction "aadA10,ant(2'')-Ia,aph(3')-Iib,fosA,crpP,sul1,blaPAO,blaOXA-395,blaOXA-10,qacE,catB7" 97.8 Good 1 1 1 1 1 1 1 0 0 1 0 1

Pseudomonas aeruginosa strain PS1875 287 WGS PS1875 VIHO01000000 225 7071125 blood USA "Human, Homo sapiens" Resistant Computational Prediction "aadA10,ant(2'')-Ia,aph(3')-Iib,fosA,crpP,sul1,blaPAO,blaOXA-395,blaOXA-10,qacE,catB7" 97.9 Good 1 1 1 1 1 1 1 0 0 1 0 1

Pseudomonas aeruginosa strain BWH031 287 WGS BWH031 VIIA01000000 101 7035660 Abd Wound USA "Human, Homo sapiens" Resistant Computational Prediction "crpP,fosA,catB7,blaOXA-395,aph(3')-Iib" 98.4 Good 1 1 1 1 1 1 1 0 0 1 0 1

Pseudomonas aeruginosa strain BWH011 287 WGS BWH011 VIIB01000000 125 7075697 BAL USA "Human, Homo sapiens" Resistant Computational Prediction "crpP,fosA,catB7,blaOXA-395,aph(3')-Iib" 98.5 Good 1 1 1 1 1 1 1 0 0 1 0 1

Pseudomonas aeruginosa AZPAE14951 287 WGS AZPAE14951 JTQQ00000000 141 6876017 urinary tract infection Argentina "Human, Homo sapiens" Resistant;Susceptible AMR Panel "crpP,fosA,catB7,blaOXA-395,aph(3')-Iib" 99 99.1 Good 1 1 1 1 1 1 1 0 0 1 0 1

Pseudomonas aeruginosa strain 174319 287 WGS 174319 SISM01000000 160 6940236 "Urine, blood, lung" France "Human, Homo sapiens" Resistant Computational Prediction "rmtF,aac(6')-Ib-Hangzhou,aac(6')-Ib-cr,aph(3')-Iib,fosA,crpP,sul1,tet(G),blaPAO,blaOXA-50,blaOXA-10,blaOXA-4,blaOXA-1,catB7,qacE" 99.2 Good 1 1 0 1 1 1 1 0 1 1 0 1

Pseudomonas aeruginosa AZPAE14950 287 WGS AZPAE14950 JTQR00000000 149 6332797 urinary tract infection Argentina "Human, Homo sapiens" Susceptible;Resistant AMR Panel "crpP,fosA,catB7,blaOXA-486,aph(3')-Iib" 98.5 99.1 Good 1 1 0 1 1 1 1 0 0 0 0 1

Pseudomonas aeruginosa strain GO78 287 WGS GO78 VJNJ01000000 66 6553583 Duke University Hospital USA Resistant Computational Prediction "crpP,fosA,catB7,blaOXA-395,aph(3')-Iib" 99.3 Good 1 1 1 1 1 1 1 0 0 0 0 1

Pseudomonas aeruginosa strain GO72 287 WGS GO72 VJMW01000000 60 6743815 Duke University Hospital USA Resistant Computational Prediction "crpP,fosA,catB7,blaOXA-50,aph(3')-Iib" 98.8 Good 1 1 0 1 1 1 1 0 0 0 0 1

Pseudomonas aeruginosa strain GO73 287 WGS GO73 VJNL01000000 83 6734343 Duke University Hospital USA Resistant Computational Prediction "crpP,fosA,catB7,blaOXA-486,aph(3')-Iib" 99.1 Good 1 1 0 1 1 1 1 0 0 0 0 1

Pseudomonas aeruginosa strain NSC1791 287 WGS NSC1791 VKLB01000000 155 7234569 urine Peru "Human, Homo sapiens" Resistant Computational Prediction "crpP,aac(6')-29a,aac(6')-29b,aac(6')-Ib-Hangzhou,aac(6')-Ib-cr,aph(3')-Iib,sul1,blaVIM-2,blapAO,blaOXA-395,blaIMP-18,blaOXA-2,qacE,catB7" 97.9 Good 1 1 1 1 1 1 1 0 0 0 0 1

Pseudomonas aeruginosa strain NSC1664 287 WGS NSC1664 VKLA01000000 256 6709954 peritoneal fluid Peru "Human, Homo sapiens" Resistant Computational Prediction "fosA,ant(2'')-Ia,aph(3')-Iib,crpP,sul1,blaPAO,blaIMP-74,blaIMP-16,blaOXA-50cmlA1,qacE,catB7" 97.3 Good 1 1 1 1 1 1 1 0 0 1 0 1

Pseudomonas aeruginosa strain UY1PSABAL 287 WGS UY1PSABAL VLHS01000000 136 7029327 broncho alveolar lavage Cameroon "Human, Homo sapiens" Resistant Computational Prediction "rmtB,aph(3')-Iib,aph(6)-Id,aph(3'')-Ib,fosA,sul1,crpP,tet(G),blaOXA-395,blaPAO,catB7" 98.2 Good 1 1 1 1 1 1 1 0 1 1 0 1

Pseudomonas aeruginosa strain 60503 287 Complete 60503 CP041774 1 6809062 sputum specimen China "Human, Homo sapiens" Resistant Computational Prediction "crpP,fosA,catB7,blaOXA-395,aph(3')-Iib" 99.3 Good 1 1 1 1 1 1 0 0 0 1 0 1

Pseudomonas aeruginosa strain A681 287 Complete A681 CP041771 1 6616247 sputum China "Human, Homo sapiens" Resistant Computational Prediction "crpP,fosA,catB7,blaOXA-486,aph(3')-Iib" 99.3 Good 1 1 0 1 1 1 1 0 0 0 0 1
[truncated: 11,816 more chars]
